# Synthesis of New Phenothiazine/3-cyanoquinoline and Phenothiazine/3-aminothieno[2,3-b]pyridine(-quinoline) Heterodimers

**DOI:** 10.3390/ijms26199798

**Published:** 2025-10-08

**Authors:** Victor V. Dotsenko, Vladislav K. Kindop, Vyacheslav K. Kindop, Eva S. Daus, Igor V. Yudaev, Yuliia V. Daus, Alexander V. Bespalov, Dmitrii S. Buryi, Darya Yu. Lukina, Nicolai A. Aksenov, Inna V. Aksenova

**Affiliations:** 1 Department of Organic Chemistry and Technologies, Kuban State University, 149 Stavropolskaya St., 350040 Krasnodar, Russia; vlad.kindop@mail.ru (V.K.K.); slavakindop@mail.ru (V.K.K.); bespalov-alex@mail.ru (A.V.B.); buryy.ds@gmail.com (D.S.B.); dashakosylina@rambler.ru (D.Y.L.); 2 Department of Chemistry, North Caucasus Federal University, 1a Pushkin St., 355017 Stavropol, Russia; radioanimation@rambler.ru (N.A.A.); 3 Faculty of Energetics, Kuban State Agrarian University, 13 Kalinina St., 350044 Krasnodar, Russia; etsh1965@mail.ru (I.V.Y.); zirochka2505@gmail.com (Y.V.D.)

**Keywords:** phenothiazine heterodimers, thieno[2,3-b]pyridines, thieno[2,3-b]quinolines, quinoline-3-carbonitriles, Thorpe–Ziegler cyclization, α-thiocyanatoacetamides, 2-iminothiazolidine-4-ones, Gruner–Gewald rearrangement1

## Abstract

The aim of this work was to prepare new heterodimeric molecules containing pharmacophoric fragments of 3-cyanoquinoline/3-aminothieno[2,3-b]pyridine/3-aminothieno[2,3-b]quinoline on one side and phenothiazine on the other. The products were synthesized via selective S-alkylation of readily available 2-thioxo-3-cyanopyridines or -quinolines with N-(chloroacetyl)phenothiazines, followed by base-promoted Thorpe–Ziegler isomerization of the resulting N-[(3-cyanopyridin-2-ylthio)acetyl]phenothiazines. We found that both the S-alkylation and the Thorpe–Ziegler cyclization reactions, when conducted with KOH under heating, were accompanied to a significant extent by a side reaction involving the elimination of phenothiazine. Optimization of the conditions (0–5 °C, anhydrous N,N-dimethylacetamide and NaH or *t-*BuONa as non-nucleophilic bases) minimized the side reaction and increased the yields of the target heterodimers. The structures of the products were confirmed by IR spectroscopy, ^1^H, and ^13^C DEPTQ NMR studies. It was demonstrated that the synthesized 3-aminothieno[2,3-b]pyridines can be acylated with chloroacetyl chloride in hot chloroform. The resulting chloroacetamide derivative reacts with potassium thiocyanate in DMF to form the corresponding 2-iminothiazolidin-4-one; in this process, phenothiazine elimination does not occur, and the Gruner–Gewald rearrangement product was not observed. The structural features and spectral characteristics of the synthesized 2-iminothiazolidin-4-one derivative were investigated by quantum chemical methods at the B3LYP-D4/def2-TZVP level. A range of drug-relevant properties was also evaluated using in silico methods, and ADMET parameters were calculated. A molecular docking study identified a number of potential protein targets for the new heterodimers, indicating the promise of these compounds for the development of novel antitumor agents.

## 1. Introduction

In recent years, the concept of molecular hybridization—combining two or more pharmacophore scaffolds into a single molecule—has been employed to develop pharmacological drugs with enhanced efficacy [1,2,3,4]. This approach operates on the principle that a hybrid molecule incorporates structural features from two (in the case of heterodimers) or more parent pharmacophores, each of which independently and selectively acts on distinct pharmacological targets. The inclusion of multiple pharmacophore subunits in a conjugate often leads to a synergistic effect that surpasses the combined impact of the individual compounds. These pharmacophore subunits may function independently, with different fragments binding to separate targets, or they may act simultaneously by interacting with different regions of the same target protein.

Recently, molecular hybrids have shown promise as therapeutic agents for a variety of conditions, including cancer [5,6,7,8,9,10,11,12,13,14,15], Alzheimer’s disease [16,17,18,19,20,21,22,23,24,25,26,27], malaria [28,29,30,31,32,33,34], tuberculosis [35,36,37,38,39,40,41,42], HIV [37,43,44,45,46,47,48,49], and SARS-CoV-2 [50]. They have also demonstrated efficacy against bacterial and fungal infections [51,52,53,54,55,56,57,58,59,60,61], as well as potential applications as antidiabetic drugs [62,63,64,65,66,67,68,69,70,71], anticoagulants and platelet aggregation inhibitors [72,73,74,75], analgesics [76,77,78,79,80,81], photopharmacological compounds [82], and anti-inflammatory agents [83,84,85]. Furthermore, molecular hybrids demonstrate significant potential for creating novel materials possessing photochromic and luminescent characteristics. They also show utility as reagents in fine organic synthesis, serve as high-energy-density materials, and find purpose in various other applications [86,87,88,89,90,91,92,93].

In continuation of our research on condensed S,N-heterocyclic systems [94,95,96,97,98,99,100,101,102], we aimed to investigate the possibility of obtaining new molecular hybrids combining a phenothiazine fragment on one side with either nicotinonitrile or 3-aminothieno[2,3-b]pyridine on the other.

**Figure 1:**
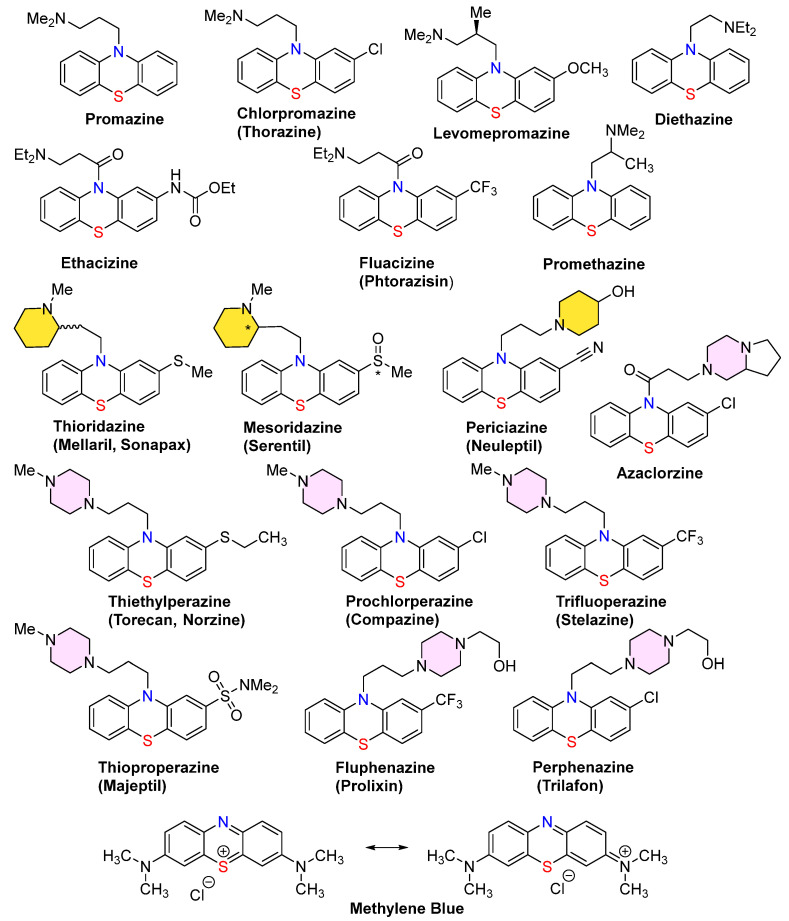
Biologically active phenothiazines.

The choice of starting components for the synthesis of these molecular hybrids was guided by several considerations. First, phenothiazine derivatives are readily available and well-known for their broad range of practical applications. In medical practice, phenothiazine-based neuroleptics, antiemetics, and antipsychotics—such as promazine and chlorpromazine (Thorazine)—are widely used to treat mental disorders, Parkinson’s disease, motion sickness, and rheumatism (Figure 1) [103,104,105]. It is noteworthy that several established drugs are, in fact, hybrid heterodimers of phenothiazine with piperidine—examples include thioridazine (Mellaril, Sonapax), periciazine (Neuleptil), and Mesoridazine (Serentil)—or with piperazine, such as fluphenazine (Prolixin), perphenazine (Trilafon), prochlorperazine (Compazine), and trifluoperazine (Stelazine) (Figure 1).

The dye methylene blue (Figure 1) is actively used in medicine, photography, analytical chemistry, and the textile industry as a blue dye. In medicine, methylene blue is used as an antiseptic for treating oral and urogenital tract infections, as an antidote for cyanide, carbon monoxide, and hydrogen sulfide poisoning, is effective in treating Alzheimer’s disease, and serves in the photodynamic therapy of cancer as a potent photosensitizer that promotes photo-induced destruction of tumor cells [106,107,108,109,110,111].

Next, phenothiazines are also of interest as compounds exhibiting anticancer [112,113], antiprotozoal [113,114], fungicidal [114], and other effects. They also function as antioxidants [115], dyes and fluorophores for optoelectronics [108,116,117,118], chemosensors for the analytical determination of cations and anions [119,120,121], molecular generators of NO [122], DNA sensors [123], photocatalysts [124,125,126], among other applications.

Heterodimeric molecules based on phenothiazine have also found diverse applications (for a recent review, see [127]). Recently, hybrid molecules containing a phenothiazine fragment have been proposed as multifunctional agents for the potential treatment of neurodegenerative disease [128], cholinesterase modulators [129], acetylcholinesterase/butyrylcholinesterase inhibitors [130], antioxidants [131,132], and antitumor agents [133,134,135,136].

On the other hand, nicotinonitriles (3-cyanopyridines) and their close structural analogs, such as 3-cyanoquinolines, were recognized as readily available reagents for organic synthesis and exhibit a promising profile of biological activity (for reviews, see [137,138,139,140,141,142,143,144,145,146]). In recent years, hybrid molecules containing a nicotinonitrile fragment have been reported as acetylcholinesterase inhibitors [147], anticancer agents [148,149], chromophores [150], hypolipidemic and hepatoprotective agents [151,152,153], analgesics [154,155,156,157], plant growth regulators and herbicide safeners [158,159], and EGFR/BRAFV600E inhibitors [160].

**Figure 2:**
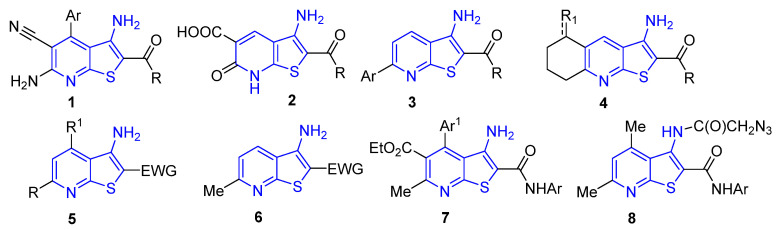
Biologically active 3-aminothieno[2,3-b]pyridines.

Among 3-aminothieno[2,3-b]pyridines and -quinolines (for reviews, see [95,161,162,163,164,165]), numerous active molecules have also been identified. Notable examples include 3,6-diaminothieno[2,3-b]pyridines **1**, recognized as dual inhibitors of Hsp90 and B-Raf [166], antiplasmodial agents [167,168,169,170], and adenosine receptor agonists [171,172]. Thienopyridine-5-carboxylic acids **2** [173] act as HIV-1 integrase inhibitors [174], IKKβ inhibitors [175], and ubiquitin C-terminal hydrolase-L1 (UCH-L1) inhibitors [176] (Figure 2).

6-Aryl-3-aminothieno[2,3-b]pyridines **3** exhibit a broad spectrum of biological activity, ranging from antimicrobial [177] to antitumor [178,179]. Structurally related thieno[2,3-b]quinolines **4** demonstrate antitumor activity [180,181,182,183] and also inhibit platelet aggregation [184]. 4,6-Disubstituted thienopyridines **5** have been described as potent IκB kinase β inhibitors [185], Pim-1 inhibitors [186], and non-competitive Epac1 inhibitors [187], while compounds **6** are active against the HIV-1 virus [188].

The potential of thienopyridines in agrochemistry is illustrated by compounds **7**, which exhibit insecticidal activity against *Aonidiella aurantii* [189], and by azidoacetamides **8**, which show a herbicide safening effect against 2,4-D in sunflower seedlings [190] (Figure 2).

Hybrid molecules bearing a thienopyridine fragment remain relatively unexplored. Reported examples include thienopyridine/coumarin heterodimers with antiacetylcholinesterase activity [191], thiazolidine/thienopyridine fungicides [192], and thieno[2,3-b]quinoline/procaine hybrid molecules that are allosteric SHP-1 activators evolved from PTP1B inhibitors [193].

According to recent reviews on the chemistry of phenothiazine heterodimers [127] and thienopyridines/thienoquinolines [164,165], along with the results of our literature search, hybrid molecules combining phenothiazine with nicotinonitrile or thienopyridine(quinoline) fragments have not been previously described.

This work presents the synthesis of such hybrid molecules and an investigation into some of their properties.

## 2. Results and Discussion

### 2.1. Synthesis

**Scheme 1:**
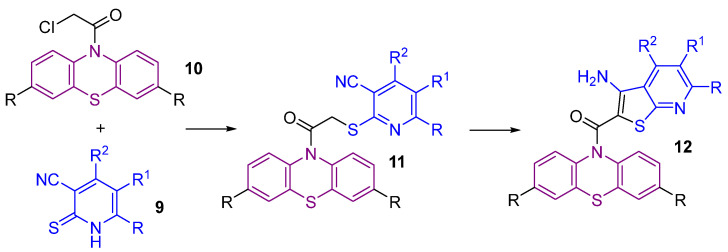
Synthesis strategy for nicotinonitrile–phenothiazine and thieno[2,3-b]pyridine–phenothiazine heterodimers.

To prepare the target heterodimers, we selected a synthetic strategy involving the S-alkylation of readily available 3-cyanopyridine-2(1H)-thiones **9** with N-(chloroacetyl)phenothiazines **10**. The resulting sulfides **11** can subsequently undergo Thorpe–Ziegler cyclization under basic conditions, enabling a one-pot conversion into the thienopyridine products **12** (Scheme 1).

**Scheme 2:**
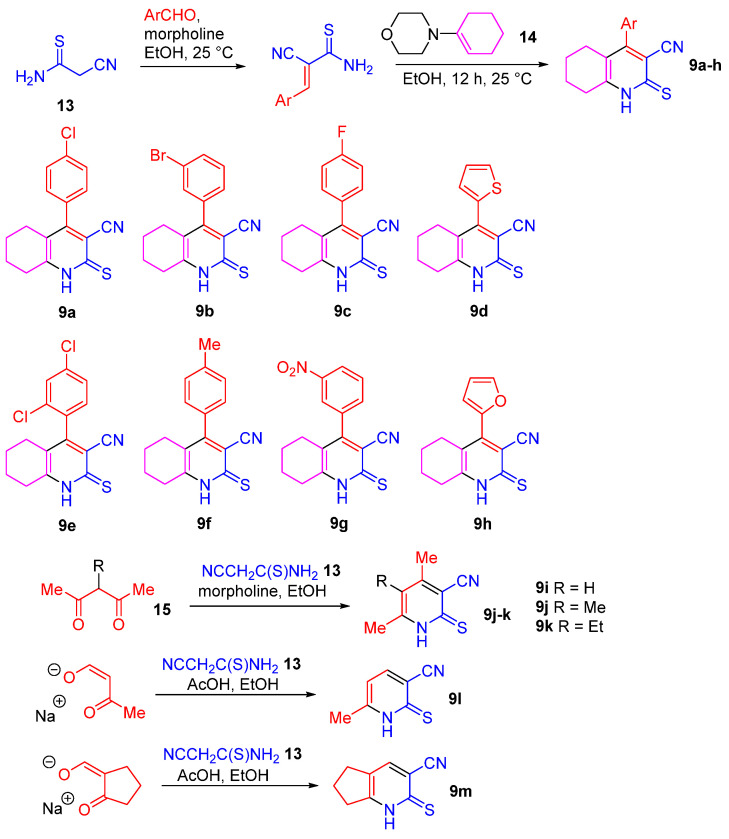
Preparation of starting 2-thioxoquinolines and 2-thioxopyridines **9a-m**.

Starting 2-thioxoazines **9** were prepared through several routes (Scheme 2). Thus, 4-aryl-2-thioxoquinolines **9a-h** were synthesized by the sequential treatment of cyanothioacetamide **13** (for review, see [194]) with aromatic aldehydes and enamine **14** [195,196,197,198]. It should be noted that quinolines **9b** (Ar = 3-BrC_6_H_4_) and **9e** (Ar = 2,4-Cl_2_C_6_H_3_) have not been previously described in the literature.

Next, 2-thioxonicotinonitriles **9i-k** were prepared by reacting thioamide **13** with 1,3-diketones **15** via the Guareschi–Thorpe reaction according to known procedures [199,200]. Starting pyridines **9l** [201] and **9m** [202] were prepared by the reaction of cyanothioacetamide **13** with sodium enolates of α-formyl ketones in the presence of AcOH (Scheme 2).

**Scheme 3:**
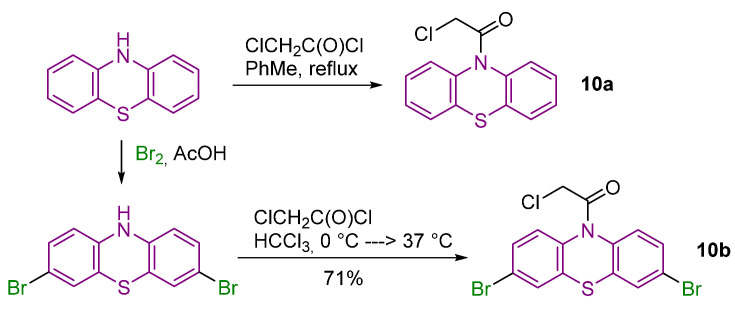
Synthesis of starting chloroacetyl phenothiazines **10a,b**.

**Figure 3:**
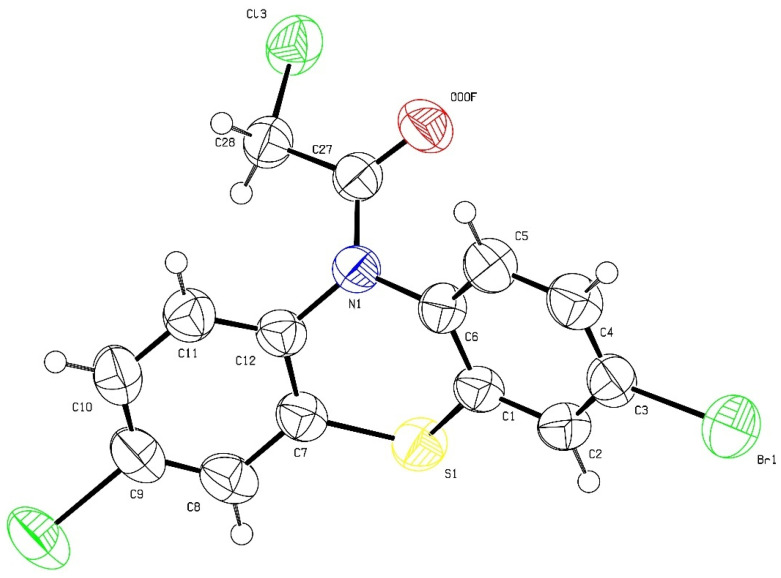
ORTEP drawing of X-ray structure for 3,7-dibromo-10-(chloroacetyl)phenothiazine **10b** with numbering system (not the IUPAC standard) and ellipsoids with 50% probability (CCDC deposition number 2478604).

Synthesis of N-(chloroacetyl)phenothiazine **10a** was achieved by acylating phenothiazine with chloroacetyl chloride [203]. A similar reaction with 3,7-dibromophenothiazine [204,205] afforded chloroacetamide **10b** (Scheme 3). The structure of 3,7-dibromo-10-(chloroacetyl)phenothiazine **10b** was studied by single-crystal X-ray diffraction analysis (Figure 3).

**Scheme 4:**
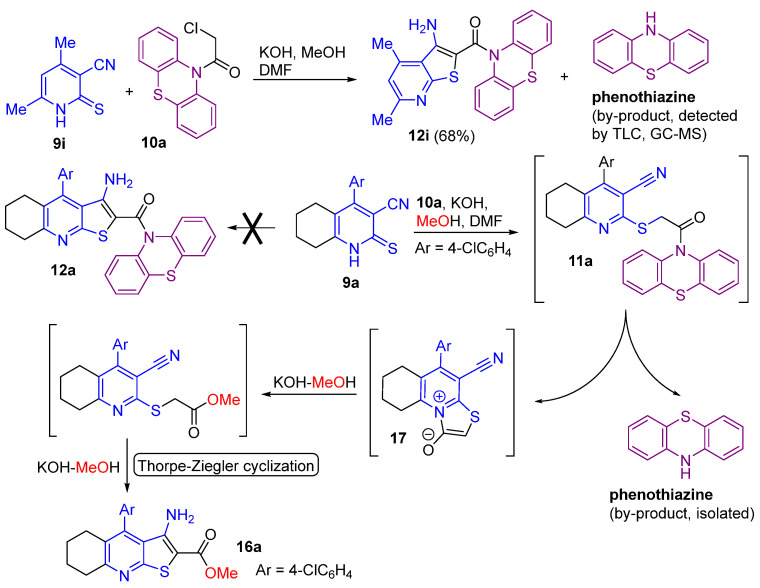
KOH/MeOH-promoted Thorpe–Ziegler reaction with 10-(chloroacetyl)phenothiazines **10**.

Traditionally, the reaction of 3-cyanopyridine-2(1H)-thiones **9** with alkylating agents, followed by Thorpe–Ziegler isomerization into 3-aminothieno[2,3-b]pyridines, is carried out in the presence of strong bases such as KOH, NaOH, EtONa, etc., most commonly under heating [95,161,162,163,164,165]. However, when we attempted to synthesize the Thorpe–Ziegler product from thioxopyridine **9i** and N-(chloroacetyl)phenothiazine **10a** using KOH in MeOH, we observed an unusual behavior in this reaction. Although the expected product **12i** was still isolated in a 68% yield, TLC and GC-MS analysis confirmed the formation of a significant amount of unsubstituted phenothiazine (Scheme 4).

Surprisingly, when 2-thioxoquinoline **9a** was reacted with chloride **10a** in the presence of KOH in MeOH, the expected heterodimer **12a** was not obtained at all. Instead, the methyl ester **16a** was formed in a 61% yield [206] (Scheme 4).

We propose that the probable cause is the specific nature of the N-acyl phenothiazine fragment, in which the phenothiazine moiety acts in an uncharacteristic role as a labile leaving group. This leads to the formation of a mesoionic intermediate **17**, which, under the action of methanolic KOH, undergoes cleavage to form the corresponding methyl ester. Subsequent Thorpe–Ziegler cyclization led to the formation of the aforementioned ester **16**. More experimental details are provided in our paper [206].

Undoubtedly, this remarkably facile elimination of the phenothiazine moiety through the formation of mesoionic species is of significant theoretical and practical interest. However, a detailed investigation of the scope and limitations of this reaction falls outside the scope of the present work and will be the subject of further studies.

Given the aforementioned challenges in synthesizing phenothiazine heterodimers **11** and **12**, we opted to avoid heating as well as strongly nucleophilic solvents and bases in favor of milder reaction conditions.

**Scheme 5:**
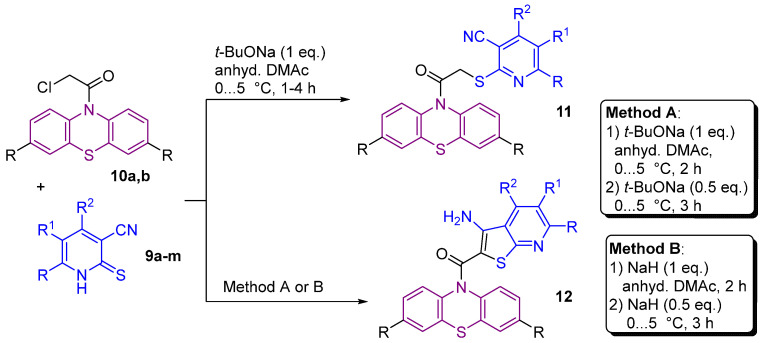
Synthesis of nicotinonitrile–phenothiazine heterodimers **11** and thieno[2,3-b]pyridine–phenothiazine heterodimers **12**.

**Figure 4:**
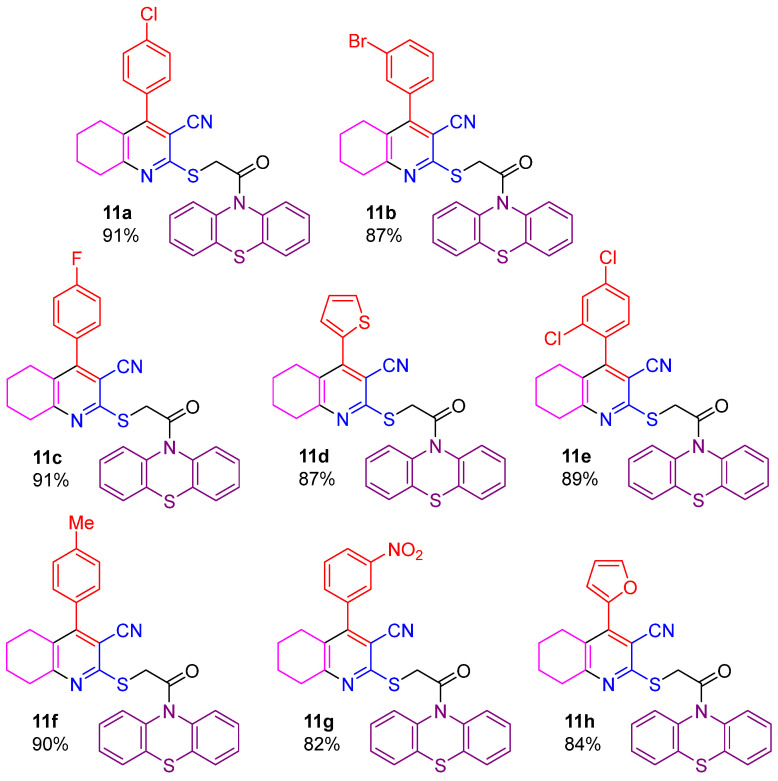
Structure and yields of nicotinonitrile–phenothiazine heterodimers **11a-h**.

**Figure 5:**
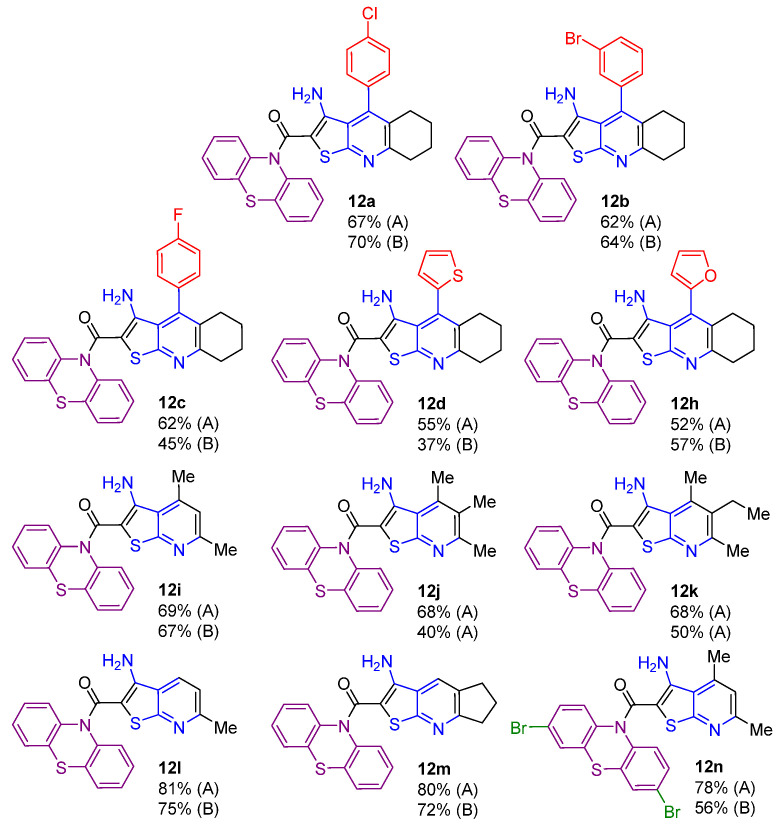
Structure and yields of thieno[2,3-b]pyridine(thieno[2,3-b]quinoline)–phenothiazine heterodimers **12** (the method of preparation—**A** or **B**—is indicated in parentheses).

We decided to conduct the S-alkylation reaction and subsequent Thorpe–Ziegler cyclization under cooling, while also employing non-nucleophilic basic catalysts and solvents. Success was achieved using the systems *t*-BuONa–anhydrous N,N-dimethylacetamide (DMAc) (Method A) and NaH–DMAc (Method B, Scheme 5, Figure 4 and Figure 5). The use of *t*-BuONa as a base appears preferable as it affords somewhat higher yields (Figure 5).

Compounds **11** are beige or pale yellow powders, typically exhibiting poor solubility in benzene and alcohols, and limited solubility in EtOAc, acetone, methylene chloride, as well as in CDCl_3_ or (CD_3_)_2_SO at 25 °C. The IR spectra of heterodimers **11a–h** display an intense absorption band for the conjugated cyano group at ν = 2218–2222 cm^−1^, along with an absorption band for the amide C=O group at ν = 1682–1686 cm^−1^.

Characteristic signals in the ^1^H NMR spectra of cyanoquinoline–phenothiazine heterodimers **11** include multiplets for the protons of four methylene groups in the range δ 1.56–1.69, 1.72–1.80, 2.09–2.65, and 2.80–2.86 ppm, and a singlet for the SCH_2_ protons at δ 4.39–4.42 ppm.

In the ^13^C NMR spectra of compounds **11**, the characteristic signals of the four methylene carbons at δ 21.5–21.6, 21.5–21.8, 25.6–26.5, and 32.8–33.1 ppm, SCH_2_ (δ 33.5–33.7 ppm), quinoline C-3 (δ 101.2–104.5 ppm), C≡N carbon (δ 114.5–115.5 ppm), quinoline C-4a (δ 126.3–128.0 ppm), and the carbonyl group signals (δ 166.0–166.1 ppm) are observed. Phenothiazine fragment shows a characteristic set of signals for CH carbons (δ 127.25–127.28, 127.34–127.39, and 128.0 ppm), as well as signals for the C–S–C carbons at δ 132.2–132.3 ppm and C–N–C carbons at δ 138.0 ppm.

To our surprise, heterodimers **11** proved to be relatively unstable compounds. When heated above 70–100 °C for drying or melting point determination, compounds **11** undergo noticeable decomposition to form free phenothiazine (detected spectroscopically and by TLC) and products exhibiting orange fluorescence under UV light, for which we propose a mesoionic structure **17**. The likely reason is the previously mentioned specific behavior of the N-(acyl)phenothiazine fragment, which acts as a mild acylating agent with phenothiazine serving as the leaving group. A detailed examination of this transformation will be the subject of further investigation.

The elimination of phenothiazine is observed to some extent even under the mild synthesis conditions we selected. For instance, the NMR spectra of the crude heterodimers **11** consistently show signals corresponding to unsubstituted phenothiazine [^1^H NMR-δ 8.57–8.60 ppm (NH), δ 6.66–6.99 ppm (CH Ar); ^13^C NMR-δ 142.1 (C–N–C), 127.6 (CH), 126.3 (CH), 121.8 (CH), 116.3 (C–S–C), 114.4 (CH)] (for example, see Figures S42 and S43 in Supplementary Materials file).

Compounds **12** are yellow or yellow-brown powders, typically insoluble in benzene, alcohols, and sparingly soluble in DMSO, chloroform, or DMAc.

FTIR spectra of heterodimers **12** lack the absorption bands for a conjugated C≡N group. Instead, two absorption bands corresponding to the asymmetric and symmetric vibrations of the N–H bond of a primary amino group appeared at ν 3508–3429 cm^−1^ and 3356–3306 cm^−1^. Notably, due to conjugation, the absorption band of the amide C=O group undergoes a bathochromic shift and is observed in the range of ν 1620–1597 cm^−1^.

The ^1^H NMR spectra of 3-aminothienopyridines **12** reveal a signal for primary amino group protons as a broad singlet at δ 5.86–7.64 ppm. Characteristic signals in the ^13^C NMR spectra of compounds **12** include the carbons of the thienopyridine system: C-2 (δ 94.3–96.6 ppm), C-3a (δ 119.2–122.7 ppm), C-3 (δ 138.4–150.6 ppm), as well as amide C=O carbon (δ 164.2–164.9 ppm). The signals of the phenothiazine fragment appear as four CH carbon peaks at δ 126.9–127.1, δ 127.1–127.3, δ 127.4–127.5, and δ 127.8–127.9 ppm, along with two signals for quaternary carbons: C–S–C at δ 132.3–132.4 ppm and C–N–C at δ 138.9–139.2 ppm.

As with heterodimers **11**, the NMR spectra of crude thienopyridines **12** contain signals of unsubstituted phenothiazine (see, for example, Figures S45 and S46 in the Supplementary Materials file). Thienopyridines **12** can be purified by recrystallization from a large volume of a low-boiling non-nucleophilic solvent (e.g., acetone or CH_2_Cl_2_) or by re-precipitation from a solution using light petroleum.

**Scheme 6:**
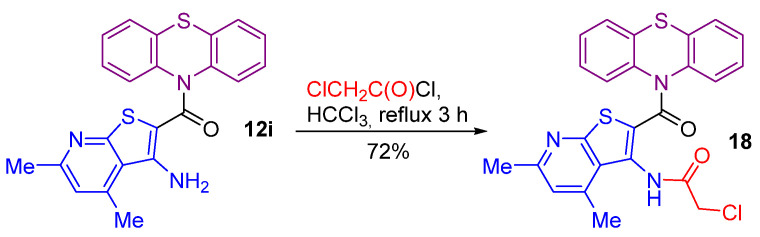
Synthesis of chloroacetamide **18**.

We investigated some reactions of the new compounds. Thus, acylation of the model thienopyridine **12j** with chloroacetyl chloride in hot chloroform yielded the expected α-chloroacetamide **18** in 72% yield (Scheme 6).

**Scheme 7:**
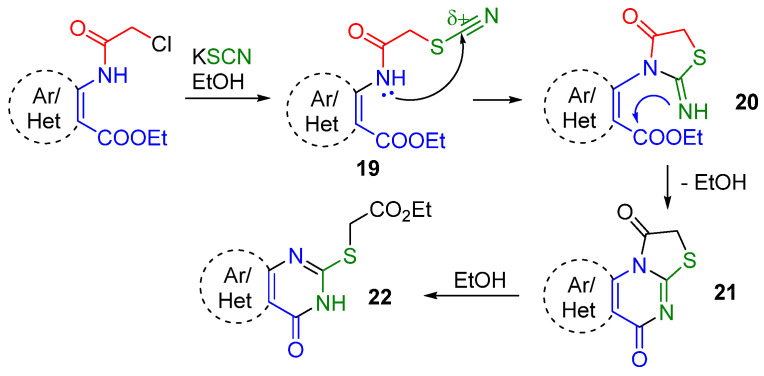
The Gruner–Gewald rearrangement of *ortho*-(chloroacetamido) carboxylates to ring-fused pyrimidines **22** [207].

The reaction of chloroacetamide **18** with potassium thiocyanate is of particular interest. In 2000, Margit Gruner, Karl Gewald, and co-workers reported [207] a cascade rearrangement of aromatic carboxylic acid esters bearing α-chloroacetamide substituent at *ortho-*position upon treatment with potassium thiocyanate in boiling alcohol (Scheme 7).

This rearrangement provides an elegant approach towards functionalized condensed pyrimidines through a sequence of steps: initial formation of nucleophilic substitution products, α-thiocyanatoacetamides **19**, which undergo in situ cyclization to give pseudothiohydantoins (2-iminothiazolidin-4-ones) **20**. These 2-iminothiazolidine species then undergo intramolecular cyclocondensation to form thiazolo[3,2-a]pyrimidines intermediates **21**, which are then nucleophilically attacked by an EtOH molecule, resulting in thiazole ring opening and the formation of ethyl (pyrimidin-2-yl)thioacetates **22** (Scheme 7).

**Scheme 8:**
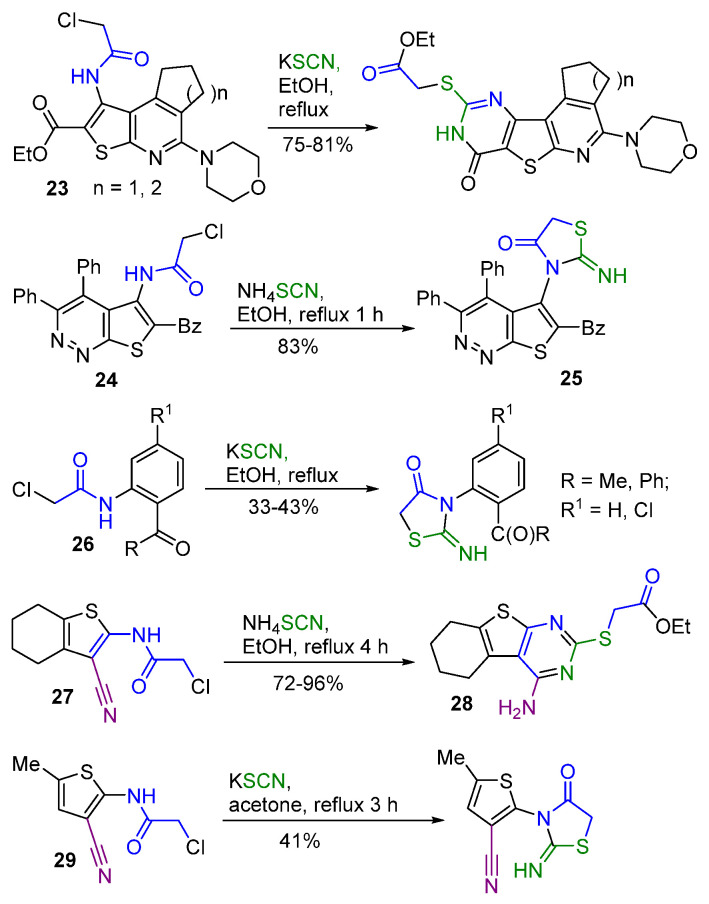
Different pathways for the reaction of *ortho-*substituted α-chloroacetamides with thiocyanates.

Later, other *ortho-*(α-chloroacetamido)-substituted esters of thieno[2,3-b]pyridine series **23** were successfully introduced into this reaction [208,209]. The structure of the electron-withdrawing substituent in the *ortho*-position relative to the α-chloroacetamide fragment plays a crucial and often ambiguous role. For instance, it was noted [210] that in the case of 2-benzoyl-3-(chloroacetamido)thieno[2,3-c]pyridazine **24,** the reaction with thiocyanate terminated at an earlier stage to afford the 2-iminothiazolidine **25** (Scheme 8).

Similarly, *ortho-*acyl-α-chloroacetanilides **26** react analogously: even under prolonged heating, the reaction progressed only to the intermediate 2-iminothiazolidin-4-ones [207]. The behavior of *ortho*-(chloroacetamido) carbonitriles is also ambiguous. While nitrile **27** reacts smoothly with ammonium thiocyanate in ethanol or an ethanol–dioxane mixture to form the corresponding thienopyrimidine **28** [211,212], the reaction of the structurally related thiophene-3-carbonitrile **29** in boiling acetone stops at the 2-iminothiazoline stage [213] (Scheme 8).

**Scheme 9:**
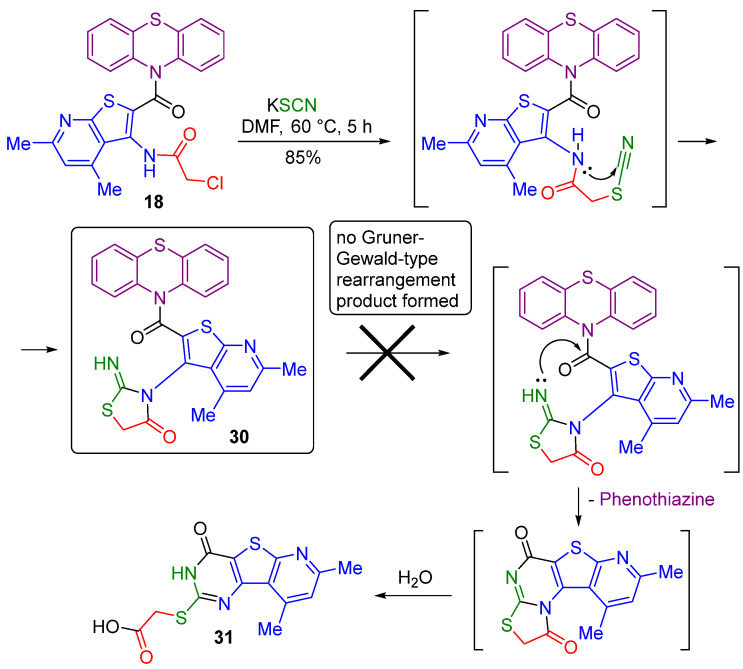
The reaction of chloroacetamide **18** with potassium thiocyanate.

We found that the reaction of chloroacetamide **18** with potassium thiocyanate proceeds under prolonged heating (60–70 °C, 5 h) in DMF to give 2-iminothiazolidin-4-one **30** in a high yield (Scheme 9). Notably, we did not observe the formation of any Gruner–Gewald rearrangement products such as **31**. The likely reason for the failure of the cyclization/rearrangement is the bulkiness of the phenothiazine substituent and the relatively mild cyclization conditions.

An interesting feature of the FTIR spectrum of compound **30** is the strong hypsochromic shift of the thiazolidinone C=O band (1720 cm^−1^), which is unusual for typical amide groups. For this reason, we performed quantum chemical calculations of the molecular geometry and vibrational frequencies of the IR spectrum for molecule **30** using the hybrid functional B3LYP with D4 dispersion correction in the def2-TZVP basis set.

**Figure 6:**
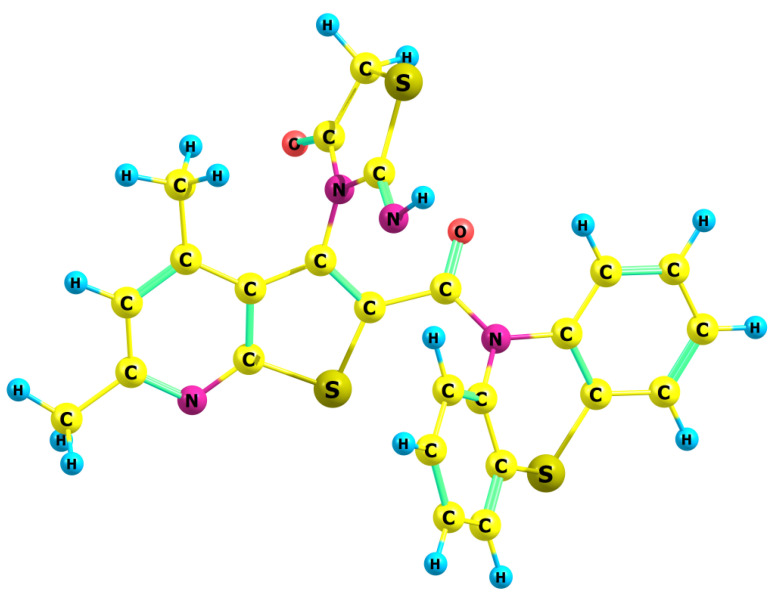
The optimized molecular structure of compound **30** calculated at the B3LYP-D4/def2-TZVP level of theory.

The molecular structure of compound **30** is presented in Figure 6. As we can see, the 2-iminothiazolidinone ring is oriented nearly perpendicular to the thieno[2,3-b]pyridine fragment, with a torsion angle C=C–N–C=O of 96.3° between them.

A comparison of the calculated vibrational frequencies with experimental results is shown in Table 1. The use of correction factors [214] significantly improves the agreement between the calculated frequencies and experimental values, reducing the mean absolute percentage error (MAPE) from 2.83% (without correction) to just 0.77% (with correction factors). Analysis of the results confirmed that the C=O stretching vibration band in the 2-iminothiazolidin-4-one fragment is indeed shifted to a higher wavenumber compared to standard values for amide groups. This shift is likely due to both the incorporation of the amide group into a five-membered ring and the strong conjugation of the amide nitrogen atom with the C=N double bond.

### 2.2. Drug-Relevant Properties, ADMET, and Docking Studies

In the context of studies on a potential biological activity of the new heterodimeric molecules, we performed in silico calculations of bioavailability parameters for structures **11**, **12**, **18**, and **30**. A preliminary analysis of the structures for compliance with C. Lipinski’s “Rule of Five” (c*Log*P ≤ 5.0, molecular weight (MW) ≤ 500, TPSA ≤ 140 Å^2^) [215,216] was performed using the OSIRIS Property Explorer service [217]. The evaluated parameters included c*Log*P (the calculated logarithm of n-octanol/water partition coefficient, log(c_octanol_/c_water_)), solubility (*log*S), Topological Polar Surface Area (TPSA), toxicological parameters—risks of side effects (mutagenic, tumorigenic, reproductive effects), drug-likeness, and the overall drug score. The obtained calculated data are presented in Table S8 (Supplementary Materials file).

As we can see, the c*Log*P value exceeds 5.0 in all cases, with the least substituted thienopyridine heterodimers **12l** and **12i** showing the best values (c*Log*P 5.18 and 5.53, respectively). The *log*S value indicates low predicted solubility for the heterodimeric molecules (*log*S ranging from −8.66 to −10.87 for nitriles **11** and *log*S = −7.89 to −11.22 for thienopyridines **12**), which is in agreement with experimental observations of their low solubility. At the same time, the new molecules predominantly exhibit acceptable TPSA values (<140 Å^2^), suggesting a likely ability to permeate cell membranes or the blood–brain barrier (BBB).

The calculated drug-likeness parameter values are relatively low for heterodimers **11** (ranging from −4.13 to −10.74), while for thieno[2,3-b]pyridines **12**, **18**, and **30**, they vary between −3.67 and 5.77, depending on the nature of substituents in the thienopyridine component. The overall drug score values are also modest, not exceeding 0.10–0.13 for **11** and 0.10–0.39 for thieno[2,3-b]pyridines **12**, **18**, and **30**. For all synthesized molecules, a risk of reproductive effects is predicted.

ADMET parameters were predicted using the admetSAR software package [218]. The results are summarized in Table S9 (Supplementary Materials file). High human gastrointestinal absorption (HIA) and BBB permeability are predicted for all compounds, along with inhibitory effects on cytochrome P450 isoforms CYP1A2, CYP2C19, and CYP2C9. Evaluation of potential mutagenic effects using the Ames test suggests a probable absence of such activity.

Overall, despite low solubility (*log*S) and c*Log*P values falling outside typical bioavailability criteria, the synthesized molecules may be considered promising candidates for further screening.

To identify potential protein targets for the synthesized compounds, we performed molecular docking studies using the novel protein–ligand docking protocol GalaxySagittarius [219] on the GalaxyWeb server [220]. Initially, the 3D structures of the new compounds were optimized using molecular mechanics with the MM2 force field to refine their geometry and minimize energy. Molecular docking was conducted in “Binding compatibility prediction” and “Re-ranking using docking” modes.

Table S10 (Supplementary Materials file) presents the results of the molecular docking studies (top 10 of the best docked poses for each compound **11**, **12**, **18**, and **30**), listing the protein–ligand complexes with the lowest binding free energy ΔG_bind_ and the highest protein–ligand interaction scores. Predicted protein targets were specified using Protein Data Bank (PDB) IDs and UniProt database identifiers. As shown in Table S10, common probable protein targets for most compounds, with ΔG_bind_ values ranging from approximately −17 to −31 kcal/mol, are human peroxisome proliferator-activated receptor (PPAR) (PDB IDs 2zno, 8hum, 8hup), nuclear receptor ROR-gamma (PDB ID 7qp4), and the prosurvival BCL-2 family protein BCL-X(L) (PDB ID 3zln). Thus, the likely activity profile of the new heterodimers involves inhibiting the proliferation of certain cancer cell types.

**Figure 7:**
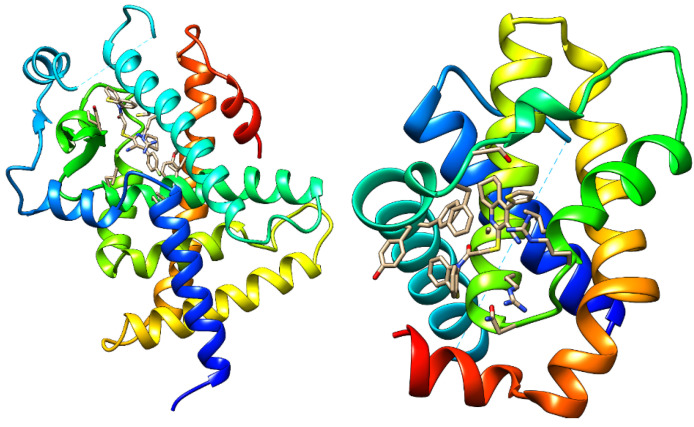
Predicted structure of the protein–ligand complex between compound **11c** and PPAR (PDB ID 2zno) (**left**), and predicted structure of the protein–ligand complex between compound **11d** and the BCL-2 family protein BCL-X(L) (PDB ID 3zln) (**right**).

**Figure 8:**
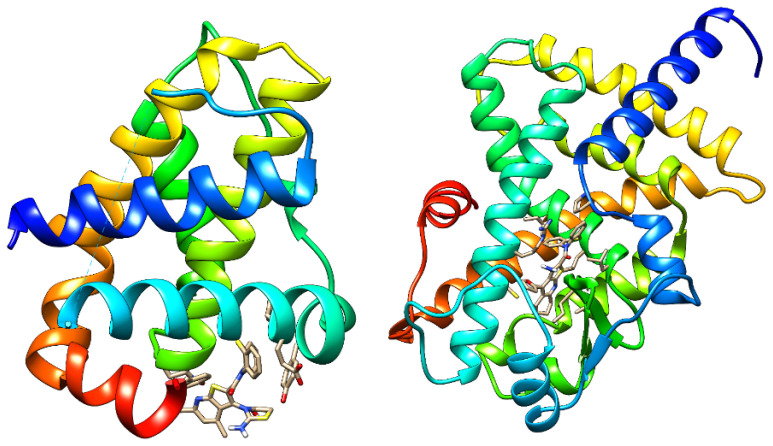
Predicted structure of the protein–ligand complex between compound **12h** and the PPAR-γ receptor (PDB ID 8hup) (**left**), and predicted structure of the protein–ligand complex between compound **30** and the BCL-2 protein (PDB ID 3zln) (**right**).

Three-dimensional visualization of the molecular docking results was generated using the UCSF Chimera software package [221,222] and is presented in Figure 7 and Figure 8.

## 3. Materials and Methods

^1^H, ^13^C, and ^13^C DEPTQ NMR spectra were recorded in solutions of DMSO-d_6_ on a Bruker AVANCE-III HD instrument (Göttingen, Germany) (at 400.40 MHz for ^1^H or 100.61 MHz for ^13^C nuclei) and a JEOL JNM-ECA-400 instrument (JEOL, Tokyo, Japan) (at 399.78 MHz for ^1^H or 100.53 MHz for ^13^C nuclei). Residual solvent signals were used as internal standards, in DMSO-d_6_—2.49 ppm for ^1^H and 39.50 ppm for ^13^C nuclei. FTIR spectra were recorded on a Bruker Vertex 70 instrument (Ettlingen, Germany) equipped with an ATR sampling module or an Infraspec FSM2201 instrument (Saint-Petersburg, Russia) in KBr pellets or in Nujol mulls. Elemental analyses were carried out using a Carlo Erba 1106 Elemental Analyzer (Milan, Italy). Single-crystal X-ray diffraction analyses were performed on an Agilent SuperNova, Dual, Cu at home/near, Atlas diffractometer (Santa Clara, CA, USA). See Electronic Supplementary Material file for NMR, FTIR spectral charts, and X-ray analysis data. Reaction progress and purity of isolated compounds were controlled by TLC on Sorbfil-A plates (produced by Imid Ltd., Krasnodar, Russia), eluents—acetone–hexane 2:1, HCCl_3_–toluene 2:1, or ethyl acetate–light petroleum. Developed TLC plates were stained with UV-light and iodine vapors. The reagents and solvents were purchased from the commercial vendor (BioInLabs, Rostov-on-Don, Russia) and used as received.

**4-Aryl-2-thioxo-1,2,5,6,7,8-hexahydroquinoline-3-carbonitriles** (**9a-h**) (Scheme 2) were prepared according to the modified reported procedures [195,196,197,198] as follows: a mixture of cyanothioacetamide **13** [223] (2.0 g, 0.02 mol) and 0.02 mol of the corresponding aromatic aldehyde in 25 mL EtOH was stirred in the presence of catalytic amounts of morpholine (2 drops; in the case of furfural and thiophen-2-carbaldehyde we used trace amounts of triethylamine) at 25 °C for 1 h. The formation of a yellow-orange precipitate of the Knoevenagel condensation products, 3-aryl-2-cyanothioacrylamide was observed. To a resulted suspension, freshly distilled 4-(cyclohex-1-en-1-yl)morpholine **14** (3.5 mL, 0.021 mol) was added, and the mixture was stirred at 25 °C for 12 h. Then a reaction mixture was treated with AcOH to adjust the pH to ~ 7. After 5 h, a yellow precipitate of the corresponding quinoline **9** was filtered off, washed with EtOH and petroleum ether, and dried at 60 °C. The resulted 3-cyanoquinoline-2(1H)-thiones **9a-h** are sufficiently pure for analytical purposes and were further used without any purification.

*4-(4-Chlorophenyl)-2-thioxo-1,2,5,6,7,8-hexahydroquinoline-3-carbonitrile* **9a** was isolated as yellow powder in 34% yield. Spectral data were identical to those reported in [198].

*4-(3-Bromophenyl)-2-thioxo-1,2,5,6,7,8-hexahydroquinoline-3-carbonitrile* **9b**. Yellow solid, yield was 4.41 g (64%).

FTIR, ν_max_, cm^−1^: 3175 (N-H); 2222 (C≡N). ^1^H NMR (400 MHz, DMSO-*d*_6_): 1.56–1.60 (m, 2H, C(6)H_2_), 1.65–1.69 (m, 2H, C(7)H_2_), 2.04–2.12 (m, 2H, C(8)H_2_), 2.75–2.78 (m, 2H, C(5)H_2_), 7.35 (dd, ^3^*J* = 7.8 Hz, ^4^*J* = 1.1 Hz, 1H, H Ar), 7.46–7.50 (m, 1H, H-5 Ar), 7.60–7.61 (m, 1H, H-2 Ar), 7.68–7.71 (m, 1H, H Ar), 13.45 (very br. s, 1H, NH). ^13^C DEPTQ NMR (101 MHz, DMSO-*d*_6_): 20.3 (C-7), 21.3 (C-6), 25.2 (C-8), 27.3 (C-5), 113.8 (C-3), 116.3 (C≡N), 120.3 (C-4a), 121.9 (C-Br), 126.7 * (CH Ar), 130.0 * (CH Ar), 131.0 * (CH Ar), 132.1 * (CH Ar), 137.3 (C-1 Ar), 152.7 (C-8a), 156.0 (C-4), 175.3 (C=S). * Negatively phased signals. Elemental Analysis: found, %: C, 55.57; H, 4.07; N, 8.28. C_16_H_13_BrN_2_S (M 345.26). Calculated, %: C, 55.66; H, 3.80; N, 8.11.

*4-(4-Fluorophenyl)-2-thioxo-1,2,5,6,7,8-hexahydroquinoline-3-carbonitrile* **9c** was isolated as yellow powder in 32% yield. Spectral data were identical to those reported in [197].

*4-(2-Thienyl)-2-thioxo-1,2,5,6,7,8-hexahydroquinoline-3-carbonitrile* **9d** was isolated as yellow-orange crystals in 39% yield. Spectral data were identical to those reported in [198].

*4-(2,4-Dichlorophenyl)-2-thioxo-1,2,5,6,7,8-hexahydroquinoline-3-carbonitrile* **9e**. Yellow solid, yield was 2.08 g (31%). FTIR, ν_max_, cm^−1^: 3173 (N-H); 2222 (C≡N). ^1^H NMR (400 MHz, DMSO-*d*_6_): 1.57–1.61 (m, 2H, C(6)H_2_), 1.65–1.70 (m, 2H, C(7)H_2_), 1.92–2.06 (m, 2H, C(8)H_2_), 2.74–2.85 (m, 2H, C(5)H_2_), 7.45 (d, ^3^*J* = 8.3 Hz, 1H, H-6 Ar), 7.63 (dd, ^3^*J* = 8.3 Hz, ^4^*J* = 2.0 Hz, 1H, H-5 Ar), 7.88 (d, ^4^*J* = 2.0 Hz, 1H, H-3 Ar), 14.16 (very br. s, 1H, NH). ^13^C DEPTQ NMR (101 MHz, DMSO-*d*_6_): 20.3 (C-7), 21.0 (C-6), 24.6 (C-8), 27.2 (C-5), 114.1 (C-3), 115.7 (C≡N), 120.5 (C-4a), 128.4 * (CH Ar), 129.4 * (CH Ar), 130.8 * (CH Ar), 131.6 (C–Cl), 132.9 (C–Cl), 135.1 (C-1 Ar), 153.3 (C-8a), 154.0 (C-4), 175.3 (C=S). * Negatively phased signals. Elemental Analysis: found, %: C, 57.27; H, 3.74; N, 8.30. C_16_H_12_Cl_2_N_2_S (M 335.25). Calculated, %: C, 57.32; H, 3.61; N, 8.36.

*4-(4-Methylphenyl)-2-thioxo-1,2,5,6,7,8-hexahydroquinoline-3-carbonitrile* **9f** was isolated as yellow powder in 54% yield. Spectral data were identical to those reported in [224].

*4-(3-Nitrophenyl)-2-thioxo-1,2,5,6,7,8-hexahydroquinoline-3-carbonitrile* **9g** was isolated as light-yellow powder in 43% yield. Spectral data were identical to those reported in [198].

*4-(2-Furyl)-2-thioxo-1,2,5,6,7,8-hexahydroquinoline-3-carbonitrile* **9h** was isolated as dark yellow powder in 42% yield. Spectral data were identical to those reported in [195].

*4,6-Dimethyl-2-thioxo-1,2-dihydropyridine-3-carbonitrile* **9i** was prepared in 94% yield according to the known method [199].

*4,5,6-Trimethyl-2-thioxo-1,2-dihydropyridine-3-carbonitrile* **9j** was prepared in 83% yield according to the known method [200].

*5-Ethyl-4,6-dimethyl-2-thioxo-1,2-dihydropyridine-3-carbonitrile* **9k** was prepared in 75% yield according to the known method [200].

*6-Methyl-2-thioxo-1,2-dihydropyridine-3-carbonitrile* **9l** was prepared in 66% yield according to the known method [201].

*2-Thioxo-2,5,6,7-tetrahydro-1H-cyclopenta[b]pyridine-3-carbonitrile* **9m** was prepared in 50% yield according to the known method [202].

*10-Chloroacetyl-10H-phenothiazine* **10a** was prepared in 76% yield according to the known method [203].

*3,7-Dibromo-10-(chloroacetyl)phenothiazine* **10b** was prepared as follows: a solution of 3.1 g (8.74 mmol) of 3,7-dibromo-10H-phenothiazine (synthesized in 82% yield by bromination of phenothiazine in AcOH according to [205]) in 40 mL of chloroform was placed in a round-bottom flask, and the mixture was cooled to 0 °C. An excess of chloroacetyl chloride (2.0 mL, 25.1 mmol) was then added dropwise, and the mixture was stirred at 37 °C for 12 h. The reaction mixture was allowed to cool to ambient temperature, and chloroform was removed using a rotary evaporator. The resulting residue was treated with 50 mL of water and extracted with dichloromethane (2 × 20 mL). The organic layer was separated and dried over anhydrous calcium chloride. The extract was purified by column chromatography (eluent–light petroleum/ethyl acetate mixture, 1:3). The solvent was then removed under reduced pressure to give yellow crystals of **10b**. The yield was 2.70 g (71%). FTIR, ν_max_, cm^−1^: 3082 (N-H); 3008, 2951 (C–H); 1690 (C=O); 1592, 1466 (Ar). ^1^H NMR (400 MHz, DMSO-*d*_6_): 4.53 (s, 2H, ClCH_2_), 7.60–7.62 (m, 4H, H Ar), 7.86 (br s, 2H, H-4, H-6 Ar). ^13^C DEPTQ NMR (101 MHz, DMSO-*d*_6_): 42.8 (ClCH_2_), 120.0 (2C, C-Br), 128.5 * (2 CH Ar), 130.4 * (2 CH Ar), 130.6 * (2 CH Ar), 134.2 (2 C-S), 136.6 (2 C-N), 165.0 (C=O). * Negatively phased signals. Elemental Analysis: found, %: C, 38.77; H, 1.94; N, 3.30. C_14_H_8_Br_2_ClNOS (M 433.54). Calculated, %: C, 38.79; H, 1.86; N, 3.23.

*X-Ray studies of single crystals of 3,7-dibromo-10-(chloroacetyl)phenothiazine* **10b**.

Single crystals of **10b** were grown from EtOAc–light petroleum (3:1). A suitable crystal was analyzed on a SuperNova, Dual, Cu at home/near, AtlasS2 diffractometer. The crystal was kept at 293(2) K during data collection. Using Olex2 [225], the structure was solved with the SHELXT structure solution program [226] using Intrinsic Phasing and refined with the SHELXL refinement package [227] using Least Squares minimization. The crystals of **10b** (C_14_H_8_Br_2_ClNOS, M = 433.54 g/mol) are monoclinic, space group P2_1_/c (no. 14), *a* = 8.14000(10) Å, *b* = 13.3616(2) Å, *c* = 41.2690(5) Å, *β* = 90.0560(10)°, *V* = 4488.56(10) Å^3^, *Z* = 12, *T* = 293(2) K, μ(Cu Kα) = 9.772 mm^−1^, *D*_calc_ = 1.925 g/cm^3^, 23,363 reflections measured (6.954° ≤ 2Θ ≤ 134.146°), 7904 unique (*R*_int_ = 0.0302, *R*_sigma_ = 0.0301), which were used in all calculations. The final *R*_1_ was 0.0468 (*I* > 2σ(I)), and *wR*_2_ was 0.1275 (all data). A full set of crystallographic data has been deposited in the Cambridge Crystallographic Data Center (CCDC Deposition Number 2478604).

**Preparation of 4-aryl-2-{[2-oxo-2-(10H-phenothiazin-10-yl)ethyl]thio}-5,6,7,8-tetra- hydroquinoline-3-carbonitriles 11a-h (Scheme 5, Figure 4). General procedure.** A round-bottom flask was charged with 1.75 mmol of the corresponding 3-cyanoquinoline-2(1H)-thione **9a**–**h** and 15 mL of anhydrous N,N-dimethylacetamide (DMAc, dried over CaH_2_) under vigorous stirring. To the solution formed, 170 mg (1.75 mmol) of sodium *tert*-butoxide was added. The reaction mixture was stirred at room temperature under protection from atmospheric moisture (calcium chloride tube) for 1 h. The resulted solution of sodium salt of **9** was then cooled to 0–5 °C and treated with 485 mg (1.75 mmol) of 10-(chloroacetyl)-10H-phenothiazine **10a**. Stirring was continued for 1–4 h under protection from moisture (monitored by TLC, eluent: chloroform–toluene 2:1). After the reaction was complete, the reaction mixture was poured into cold water under vigorous stirring. The precipitated solid was filtered off, dried under vacuum at room temperature, and if necessary, purified by dissolution in a large volume of CH_2_Cl_2_ at room temperature followed by re-precipitation using light petroleum.

*4-(4-Chlorophenyl)-2-{[2-oxo-2-(10H-phenothiazin-10-yl)ethyl]thio}-5,6,7,8-tetrahydroquinoline-3-carbonitrile* **11a**. Off-white solid, yield was 860 mg (91%). FTIR, ν_max_, cm^−1^: 2222 (C≡N); 1682 (C=O). ^1^H NMR (400 MHz, DMSO-*d*_6_): 1.58–1.62 (m, 2H, C(6)H_2_), 1.72–1.78 (m, 2H, C(7)H_2_), 2.27–2.30 (m, 2H, C(5)H_2_), 2.80–2.83 (m, 2H, C(8)H_2_), 4.41 (br. s, 2H, SCH_2_), 7.31–7.44 (m, 6H, H Ar), 7.58–7.60 (m, 4H, H Ar), 7.70–7.74 (m, 2H, H Ar). ^13^C DEPTQ NMR (101 MHz, DMSO-*d*_6_): 21.6 (CH_2_), 21.7 (CH_2_), 26.1 (CH_2_), 32.9 (CH_2_), 33.6 (SCH_2_), 103.7 (C–C≡N), 115.0 (C≡N), 126.9 (C-4a Quin **), 127.25 * (2 CH PhTz **), 127.34 * (4 CH PhTz), 128.0 * (2 CH PhTz), 128.9 * (2 CH Ar), 130.1 * (2 CH Ar), 132.2 (2C-S PhTz), 133.7 (C Ar), 134.0 (C Ar), 138.0 (2C-N PhTz), 152.4 (C-4 Quin), 156.4 (C-8a Quin), 161.6 (C-2 Quin), 166.1 (C=O). * Negatively phased signals. ** Here and throughout the paper, Quin = quinoline, PhTz = Phenothiazine. Elemental Analysis: found, %: C, 66.47; H, 3.92; N, 7.60. C_30_H_22_ClN_3_OS_2_ (M 540.10). Calculated, %: C, 66.72; H, 4.11; N, 7.78.

*4-(3-Bromophenyl)-2-{[2-oxo-2-(10H-phenothiazin-10-yl)ethyl]thio}-5,6,7,8-tetrahydroquinoline-3-carbonitrile* **11b**. Off-white solid, yield was 890 mg (87%). FTIR, ν_max_, cm^−1^: 2218 (C≡N); 1682 (C=O). ^1^H NMR (400 MHz, DMSO-*d*_6_): 1.58–1.64 (m, 2H, C(6)H_2_), 1.73–1.79 (m, 2H, C(7)H_2_), 2.27–2.32 (m, 2H, C(5)H_2_), 2.81–2.84 (m, 2H, C(8)H_2_), 4.41 (br. s, 2H, SCH_2_), 7.32–7.35 (m, 3H, H Ar), 7.41–7.50 (m, 3H, H Ar), 7.59–7.61 (m, 3H, H Ar), 7.69–7.74 (m, 3H, H Ar). ^13^C DEPTQ NMR (101 MHz, DMSO-*d*_6_): 21.5 (CH_2_), 21.7 (CH_2_), 26.1 (CH_2_), 32.9 (CH_2_), 33.6 (SCH_2_), 103.6 (C–C≡N), 115.0 (C≡N), 121.9 (C-Br), 126.9 (C-4a Quin), 127.27 * (2 CH PhTz), 127.32 * (4 CH PhTz), 127.4 * (CH Ar), 128.0 * (2 CH PhTz), 130.6 * (CH Ar), 131.0 * (CH Ar), 132.1 * (CH Ar), 132.3 (2C-S PhTz), 137.2 (C-1 Ar), 138.0 (2C-N PhTz), 151.9 (C-4 Quin), 156.4 (C-8a Quin), 161.6 (C-2 Quin), 166.0 (C=O). * Negatively phased signals. Elemental Analysis: found, %: C, 61.60; H, 3.84; N, 7.13. C_30_H_22_BrN_3_OS_2_ (M 584.55). Calculated, %: C, 61.64; H, 3.79; N, 7.19.

*4-(4-Fluorophenyl)-2-{[2-oxo-2-(10H-phenothiazin-10-yl)ethyl]thio}-5,6,7,8-tetrahydroquinoline-3-carbonitrile* **11c**. White solid, yield was 834 mg (91%). FTIR, ν_max_, cm^−1^: 2222 (C≡N); 1686 (C=O). ^1^H NMR (400 MHz, DMSO-*d*_6_): 1.57–1.63 (m, 2H, C(6)H_2_), 1.72–1.78 (m, 2H, C(7)H_2_), 2.28–2.31 (m, 2H, C(5)H_2_), 2.80–2.84 (m, 2H, C(8)H_2_), 4.40 (br. s, 2H, SCH_2_), 7.31–7.36 (m, 3H, H Ar), 7.38–7.44 (m, 5H, H Ar), 7.59 (dd, ^3^*J* = 7.8 Hz, ^4^*J* = 1.2 Hz, 2H, H Ar), 7.68–7.74 (m, 2H, H Ar). ^13^C DEPTQ NMR (101 MHz, DMSO-*d*_6_): 21.6 (CH_2_), 21.8 (CH_2_), 26.1 (CH_2_), 32.9 (CH_2_), 33.5 (SCH_2_), 103.9 (C–C≡N), 115.1 (C≡N), 115.8 * (d, ^2^*J*_C-F_ = 21.6 Hz, CH-3, CH-5 Ar), 127.1 (C-4a Quin), 127.25 * (2 CH PhTz), 127.34 * (4 CH PhTz), 128.0 * (2 CH PhTz), 130.6 * (d, ^3^*J*_C-F_ = 8.4 Hz, CH-2, CH-6 Ar), 131.2 (d, ^4^*J*_C-F_ = 3.3 Hz, C-1 Ar), 132.2 (2C-S PhTz), 138.0 (2C-N PhTz), 152.7 (C-4 Quin), 156.4 (C-8a Quin), 161.5 (C-2 Quin), 162.4 (d, ^1^*J*_C-F_ = −246.1 Hz, C-F), 166.1 (C=O). * Negatively phased signals. Elemental Analysis: found, %: C, 68.74; H, 4.32; N, 8.10. C_30_H_22_FN_3_OS_2_ (M 523.64). Calculated, %: C, 68.81; H, 4.23; N, 8.02.

*4-(2-Thienyl)-2-{[2-oxo-2-(10H-phenothiazin-10-yl)ethyl]thio}-5,6,7,8-tetrahydroquinoline-3-carbonitrile* **11d**. Off-white solid, yield was 780 mg (87%). FTIR, ν_max_, cm^−1^: 2218 (C≡N); 1686 (C=O). ^1^H NMR (400 MHz, DMSO-*d*_6_): 1.60–1.66 (m, 2H, C(6)H_2_), 1.73–1.77 (m, 2H, C(7)H_2_), 2.45–2.49 (m, 2H, C(5)H_2_), 2.80–2.83 (m, 2H, C(8)H_2_), 4.40 (br. s, 2H, SCH_2_), 7.23 (dd, ^3^*J* = 5.0 Hz, ^3^*J* = 3.7 Hz, 1H, H-4 thienyl), 7.26 (dd, ^3^*J* = 3.7 Hz, ^4^*J* = 1.4 Hz, 1H, H-3 thienyl), 7.30–7.34 (m, 2H, PhTz), 7.40–7.44 (m, 2H, PhTz), 7.59 (dd, ^3^*J* = 7.7 Hz, ^4^*J* = 1.2 Hz, 2H, PhTz), 7.68–7.73 (m, 2H, PhTz), 7.85 (dd, ^3^*J* = 5.0 Hz, ^4^*J* = 1.4 Hz, 1H, H-5 thienyl). ^13^C DEPTQ NMR (101 MHz, DMSO-*d*_6_): 21.5 (CH_2_), 21.7 (CH_2_), 26.4 (CH_2_), 32.9 (CH_2_), 33.7 (SCH_2_), 104.5 (C–C≡N), 115.0 (C≡N), 127.25 * (2 CH PhTz), 127.34 * (2 CH PhTz), 127.38 * (2 CH PhTz), 127.8 * (CH thienyl), 128.0 * (2 CH PhTz), 128.0 (C-4a Quin), 129.2 * (CH thienyl), 129.7 * (CH thienyl), 132.2 (2C-S PhTz), 133.6 (C-2 thienyl), 138.0 (2C-N PhTz), 146.6 (C-4 Quin), 156.8 (C-8a Quin), 161.7 (C-2 Quin), 166.0 (C=O). * Negatively phased signals. Elemental Analysis: found, %: C, 65.66; H, 4.35; N, 8.20. C_28_H_21_N_3_OS_3_ (M 511.68). Calculated, %: C, 65.73; H, 4.14; N, 8.21.

*4-(2,4-Dichlorophenyl)-2-{[2-oxo-2-(10H-phenothiazin-10-yl)ethyl]thio}-5,6,7,8-tetrahydroquinoline-3-carbonitrile* **11e**. White solid, yield was 895 mg (89%). FTIR, ν_max_, cm^−1^: 2222 (C≡N); 1682 (C=O). ^1^H NMR (400 MHz, DMSO-*d*_6_): 1.59–1.66 (m, 2H, C(6)H_2_), 1.72–1.78 (m, 2H, C(7)H_2_), 2.09–2.28 (m, 2H, C(5)H_2_), 2.82–2.86 (m, 2H, C(8)H_2_), 4.42 (br. s, 2H, SCH_2_), 7.31–7.35 (m, 2H, H Ar), 7.41–7.44 (m, 3H, H Ar), 7.58–7.63 (m, 3H, H Ar), 7.68–7.78 (m, 2H, H Ar), 7.88 (d, ^4^*J* = 2.0 Hz, 1H, H-3 Ar). ^13^C DEPTQ NMR (101 MHz, DMSO-*d*_6_): 21.48 (CH_2_), 21.54 (CH_2_), 25.6 (CH_2_), 32.8 (CH_2_), 33.6 (SCH_2_), 103.6 (C–C≡N), 114.5 (C≡N), 127.2 (C-4a Quin), 127.28 * (2 CH PhTz), 127.37 * (2 CH PhTz), 127.39 * (2 CH PhTz), 128.0 * (2 CH PhTz), 128.3 * (CH Ar), 129.4 * (CH Ar), 131.4 * (CH Ar), 132.2 (C–Cl, 2C-S PhTz), 132.6 (C–Cl), 135.1 (C-1 Ar), 138.0 (2C-N PhTz), 149.9 (C-4 Quin), 156.5 (C-8a Quin), 162.1 (C-2 Quin), 166.0 (C=O). * Negatively phased signals. Elemental Analysis: found, %: C, 62.50; H, 3.90; N, 7.54. C_30_H_21_Cl_2_N_3_OS_2_ (M 574.54). Calculated, %: C, 62.72; H, 3.68; N, 7.31.

*4-(4-Methylphenyl)-2-{[2-oxo-2-(10H-phenothiazin-10-yl)ethyl]thio}-5,6,7,8-tetrahydroquinoline-3-carbonitrile* **11f**. White solid, yield was 818 mg (90%). FTIR, ν_max_, cm^−1^: 2222 (C≡N); 1682 (C=O). ^1^H NMR (400 MHz, DMSO-*d*_6_): 1.56–1.62 (m, 2H, C(6)H_2_), 1.72–1.78 (m, 2H, C(7)H_2_), 2.29–2.32 (m, 2H, C(5)H_2_), 2.36 (s, 3H, Me), 2.80–2.83 (m, 2H, C(8)H_2_), 4.40 (br. s, 2H, SCH_2_), 7.19 (d, ^3^*J* = 8.0 Hz, 2H, H-3 H-5 Ar), 7.30–7.35 (m, 4H, H Ar), 7.40–7.44 (m, 2H, PhTz), 7.59 (dd, ^3^*J* = 7.7 Hz, ^4^*J* = 1.1 Hz, 2H, PhTz), 7.70–7.75 (m, 2H, PhTz). ^13^C DEPTQ NMR (101 MHz, DMSO-*d*_6_): 20.9 * (Me), 21.6 (CH_2_), 21.8 (CH_2_), 26.2 (CH_2_), 32.9 (CH_2_), 33.5 (SCH_2_), 103.8 (C–C≡N), 115.2 (C≡N), 127.0 (C-4a Quin), 127.25 * (2 CH PhTz **), 127.36 * (4 CH PhTz), 128.0 * (2 CH PhTz, 2CH Ar), 129.3 * (2 CH Ar), 132.0 (C-Me), 132.1 (2C-S PhTz), 138.0 (2C-N PhTz), 138.7 (C-1 Ar), 152.8 (C-4 Quin), 156.3 (C-8a Quin), 161.3 (C-2 Quin), 166.1 (C=O). * Negatively phased signals. Elemental Analysis: found, %: C, 71.74; H, 4.97; N, 8.11. C_31_H_25_N_3_OS_2_ (M 519.68). Calculated, %: C, 71.65; H, 4.85; N, 8.09.

*4-(3-Nitrophenyl)-2-{[2-oxo-2-(10H-phenothiazin-10-yl)ethyl]thio}-5,6,7,8-tetrahydroquinoline-3-carbonitrile* **11g**. Yellowish solid, yield was 790 mg (82%). FTIR, ν_max_, cm^−1^: 2222 (C≡N); 1686 (C=O), 1531 (NO_2 as_), 1350 (NO_2 symm_). ^1^H NMR (400 MHz, DMSO-*d*_6_): 1.60–1.63 (m, 2H, C(6)H_2_), 1.73–1.79 (m, 2H, C(7)H_2_), 2.23–2.36 (m, 2H, C(5)H_2_), 2.82-2.85 (m, 2H, C(8)H_2_), 4.42 (br. s, 2H, SCH_2_), 7.32-7.36 (m, 2H, PhTz), 7.40-7.45 (m, 2H, PhTz), 7.60 (dd, ^3^*J* = 7.7 Hz, ^4^*J* = 1.0 Hz, 2H, PhTz), 7.72-7.74 (m, 2H, PhTz), 7.82-7.84 (m, 2H, H Ar), 8.26-8.27 (m, 1H, H Ar), 8.33-8.37 (m, 1H. H Ar). ^13^C DEPTQ NMR (101 MHz, DMSO-*d*_6_): 21.5 (CH_2_), 21.7 (CH_2_), 26.1 (CH_2_), 32.9 (CH_2_), 33.6 (SCH_2_), 103.7 (C–C≡N), 115.0 (C≡N), 123.3 * (CH Ar), 124.1 * (CH Ar), 127.0 (C-4a Quin), 127.28 * (2 CH PhTz **), 127.34 * (2 CH PhTz), 127.39 * (2 CH PhTz), 128.0 * (2 CH PhTz, 2CH Ar), 130.7 * (CH Ar), 132.3 (2C-S PhTz), 135.1 * (CH Ar), 136.4 (C-1 Ar), 138.0 (2C-N PhTz), 147.9 (C-NO_2_), 151.1 (C-4 Quin), 156.5 (C-8a Quin), 161.8 (C-2 Quin), 166.0 (C=O). * Negatively phased signals. Elemental Analysis: found, %: C, 65.30; H, 4.24; N, 10.32. C_30_H_22_N_4_O_3_S_2_ (M 550.65). Calculated, %: C, 65.44; H, 4.03; N, 10.17.

*4-(2-Furyl)-2-{[2-oxo-2-(10H-phenothiazin-10-yl)ethyl]thio}-5,6,7,8-tetrahydroquinoline-3-carbonitrile* **11h**. Beige solid, yield was 729 mg (84%). FTIR, ν_max_, cm^−1^: 2218 (C≡N); 1686 (C=O). ^1^H NMR (400 MHz, DMSO-*d*_6_): 1.63–1.69 (m, 2H, C(6)H_2_), 1.74–1.80 (m, 2H, C(7)H_2_), 2.62–2.65 (m, 2H, C(5)H_2_), 2.80–2.83 (m, 2H, C(8)H_2_), 4.39 (br. s, 2H, SCH_2_), 6.75 (dd, ^3^*J* = 3.4 Hz, ^3^*J* = 1.8 Hz, 1H, H-4 furyl), 7.04 (br. d, ^3^*J* = 3.4 Hz, 1H, H-3 furyl), 7.30–7.34 (m, 2H, PhTz), 7.39–7.43 (m, 2H, PhTz), 7.58 (dd, ^3^*J* = 7.7 Hz, ^4^*J* = 1.1 Hz, 2H, PhTz), 7.70–7.74 (m, 2H, PhTz), 7.99 (br. d, ^3^*J* = 1.8 Hz, 1H, H-5 furyl). ^13^C DEPTQ NMR (101 MHz, DMSO-*d*_6_): 21.5 (CH_2_), 21.8 (CH_2_), 26.5(CH_2_), 33.1 (CH_2_), 33.7 (SCH_2_), 101.2 (C–C≡N), 112.1 * (CH-3 furyl), 115.4 * (CH-4 furyl), 115.5 (C≡N), 126.3 (C-4a Quin), 127.25 * (2 CH PhTz), 127.35 * (4 CH PhTz), 128.0 * (2 CH PhTz), 132.3 (2C-S PhTz), 138.0 (2C-N PhTz), 140.7 (C-2 furyl), 145.4 * (CH-5 furyl), 145.9 (C-4 Quin), 157.5 (C-8a Quin), 161.9 (C-2 Quin), 166.0 (C=O). * Negatively phased signals. Elemental Analysis: found, %: C, 67.61; H, 4.43; N, 8.65. C_28_H_21_N_3_O_2_S_2_ (M 495.62). Calculated, %: C, 67.86; H, 4.27; N, 8.48.

***t*-BuONa-promoted synthesis of phenothiazine/thieno[2,3-b]pyridine heterodimers 12 (Method A, Scheme 5** **, Figure 5** **). General procedure.**

A 50 mL round-bottom flask was charged with 1.2 mmol of the corresponding thione **9,** which was then dissolved in 10 mL of anhydrous DMAc under vigorous stirring. To the solution formed, 115 mg (1.2 mmol) of sodium *tert*-butoxide was added. The reaction mixture was stirred at room temperature under protection from atmospheric moisture (calcium chloride tube) for 1 h. The resulted solution of sodium salt of thione **9** was then cooled to 0–5 °C, and 1.2 mmol of the corresponding 10-(chloroacetyl)-10H-phenothiazine **10a,b** was added. Stirring was continued for 2 h under protection from moisture. Next, an additional 60 mg (~0.5 equiv.) of sodium *tert*-butoxide was added at 0–5 °C, and the reaction mixture was stirred for another 3 h (monitored by TLC, eluent: chloroform–toluene 2:1). Upon the reaction was completed, the mixture was poured into cold water under vigorous stirring. The precipitated yellow solid of product **12** was filtered off, dried under vacuum at room temperature, and purified if necessary by recrystallization or re-precipitation from a suitable solvent (DMAc, acetone, CH_2_Cl_2_).

**Na** **H** **-promoted synthesis of phenothiazine/thieno [2,3-b]pyridine heterodimers 12 (Method B, Scheme 4** **). General procedure.**

A 50 mL round-bottom flask was charged with 1.2 mmol of the corresponding thione **9,** which was then dissolved in 10 mL of anhydrous DMAc under vigorous stirring. To the solution formed, 48 mg (1.2 mmol) of sodium hydride (as 60% suspension in mineral oil) was added. The reaction mixture was stirred at room temperature under protection from atmospheric moisture (calcium chloride tube) for 1 h. The resulted solution of sodium salt of thione **9** was then cooled to 0–5 °C, and 1.2 mmol of the corresponding 10-(chloroacetyl)-10H-phenothiazine **10a,b** was added. Stirring was continued for 2 h under protection from moisture. Next, an additional 24 mg (~0.5 equiv.) of 60% NaH suspension was added at 0–5 °C, and the reaction mixture was stirred for another 3 h (monitored by TLC, eluent: chloroform–toluene 2:1). Upon the reaction was completed, the mixture was poured into cold water under vigorous stirring. The precipitated yellow solid of thienopyridine **12** was filtered off, dried under vacuum at room temperature, and purified, if necessary, by recrystallization or re-precipitation from a suitable solvent (DMAc, acetone, CH_2_Cl_2_).

*(3-Amino-4-(4-chlorophenyl)-5,6,7,8-tetrahydrothieno[2,3-b]quinolin-2-yl)(10H-phenothiazin-10-yl)methanone* **12a**. Yellow solid, yield was 435 mg (67%, method A) and 453 mg (70%, method B). FTIR, ν_max_, cm^−1^: 3483, 3333 (NH_2_); 1616 (C=O). ^1^H NMR (400 MHz, DMSO-*d*_6_): 1.58–1.64 (m, 2H, C(6)H_2_), 1.71–1.76 (m, 2H, C(7)H_2_), 2.23–2.26 (m, 2H, C(5)H_2_), 2.85–2.87 (m, 2H, C(8)H_2_), 5.91 (br. s, 2H, NH_2_), 7.30–7.34 (m, 4H, PhTz), 7.38 (d, ^3^*J* = 8.0 Hz, 2H, H Ar), 7.56–7.58 (m, 2H, PhTz), 7.64 (d, ^3^*J* = 8.0 Hz, 2H, H Ar), 7.67–7.69 (m, 2H, PhTz). ^13^C DEPTQ NMR (101 MHz, DMSO-*d*_6_): 21.9 (CH_2_), 22.2 (CH_2_), 26.2 (CH_2_), 33.0 (CH_2_), 95.7 (C-2 ThQ **), 119.2 (C-3a ThQ), 126.7 (C-4a ThQ), 127.0 * (2 CH PhTz), 127.2 * (2 CH PhTz), 127.4 * (2 CH PhTz), 127.8 * (2 CH PhTz), 129.3 * (2 CH Ar), 130.1 * (2 CH Ar), 132.4 (2C-S PhTz), 133.7 (C Ar), 133.8 (C Ar), 139.0 (2C-N PhTz), 144.4 (C-3 ThQ), 150.0 (C-4 ThQ), 157.8 (C-8a ThQ), 159.4 (C-9a ThQ), 164.3 (C=O). * Negatively phased signals. ** Here and throughout the paper, ThQ = thieno[2,3-b]quinoline. Elemental Analysis: found, %: C, 66.90; H, 4.20; N, 7.69. C_30_H_22_ClN_3_OS_2_ (M 540.10). Calculated, %: C, 66.72; H, 4.11; N, 7.78.

*(3-Amino-4-(3-bromophenyl)-5,6,7,8-tetrahydrothieno[2,3-b]quinolin-2-yl)(10H-phenothiazin-10-yl)methanone* **12b**. Yellow solid, yield was 435 mg (62%, method A) and 449 mg (64%, method B). FTIR, ν_max_, cm^−1^: 3483, 3329 (NH_2_); 1620 (C=O). ^1^H NMR (400 MHz, DMSO-*d*_6_): 1.58–1.64 (m, 2H, C(6)H_2_), 1.71–1.77 (m, 2H, C(7)H_2_), 2.21–2.32 (m, 2H, C(5)H_2_), 2.85–2.88 (m, 2H, C(8)H_2_), 5.86 (br. s, 2H, NH_2_), 7.29–7.39 (m, 5H, H Ar), 7.52–7.59 (m, 3H, H Ar), 7.62–7.63 (m, 1H, H Ar), 7.67–7.70 (m, 2H, H Ar), 7.77 (br.d, ^3^*J* = 8.1 Hz, 1H, H Ar). ^13^C DEPTQ NMR (101 MHz, DMSO-*d*_6_): 21.9 (CH_2_), 22.1 (CH_2_), 26.2 (CH_2_), 33.0 (CH_2_), 95.8 (C-2 ThQ), 119.1 (C-3a ThQ), 122.5 (C-Br), 126.6 (C-4a ThQ), 127.0 * (2 CH PhTz), 127.2 * (2 CH PhTz, CH Ar), 127.4 * (2 CH PhTz), 127.8 * (2 CH PhTz), 130.7 * (CH Ar), 131.3 * (CH Ar), 132.0 * (CH Ar), 132.3 (2C-S PhTz), 137.2 (C-1 Ar), 139.0 (2C-N PhTz), 143.9 (C-3 ThQ), 149.8 (C-4 ThQ), 157.8 (C-8a ThQ), 159.4 (C-9a ThQ), 164.2 (C=O). * Negatively phased signals. Elemental Analysis: found, %: C, 61.72; H, 3.96; N, 7.35. C_30_H_22_BrN_3_OS_2_ (M 584.55). Calculated, %: C, 61.64; H, 3.79; N, 7.19.

*(3-Amino-4-(4-fluorophenyl)-5,6,7,8-tetrahydrothieno[2,3-b]quinolin-2-yl)(10H-phenothiazin-10-yl)methanone* **12c**. Yellow solid, yield was 390 mg (62%, method A) and 283 mg (45%, method B). FTIR, ν_max_, cm^−1^: 3485, 3350 (NH_2_); 1616 (C=O). ^1^H NMR (400 MHz, DMSO-*d*_6_): 1.55–1.61 (m, 2H, C(6)H_2_), 1.68–1.74 (m, 2H, C(7)H_2_), 2.21–2.25 (m, 2H, C(5)H_2_), 2.81–2.86 (m, 2H, C(8)H_2_), 5.89 (br. s, 2H, NH_2_), 7.29–7.39 (m, 8H, H Ar), 7.53–7.56 (m, 2H, H Ar), 7.65–7.68 (m, 2H, H Ar). ^13^C NMR (101 MHz, DMSO-*d*_6_): 21.4 (CH_2_), 21.9 (CH_2_), 26.2 (CH_2_), 33.0 (CH_2_), 95.5 (C-2 ThQ), 116.3 (d, ^2^*J*_C-F_ = 21.1 Hz, CH-3, CH-5 Ar), 119.4 (C-3a ThQ), 126.9 (C-4a ThQ), 127.0 (2 CH PhTz), 127.2 (2 CH PhTz), 127.4 (2 CH PhTz), 127.8 (2 CH PhTz), 130.3 (d, ^3^*J*_C-F_ = 7.7 Hz, CH-2, CH-6 Ar), 131.1 (C-1 Ar), 132.4 (2C-S PhTz), 139.0 (2C-N PhTz), 144.7 (C-3 ThQ), 150.2 (C-4 ThQ), 157.8 (C-8a ThQ), 159.4 (C-9a ThQ), 162.2 (d, ^1^*J*_C-F_ = -244.4 Hz, C-F), 164.3 (C=O). Elemental Analysis: found, %: C, 68.94; H, 4.40; N, 8.13. C_30_H_22_FN_3_OS_2_ (M 523.64). Calculated, %: C, 68.81; H, 4.23; N, 8.02.

*(3-Amino-4-(2-thienyl)-5,6,7,8-tetrahydrothieno[2,3-b]quinolin-2-yl)(10H-phenothiazin-10-yl)methanone* **12d**. Yellow solid, yield was 338 mg (55%, method A) and 227 mg (37%, method B). FTIR, ν_max_, cm^−1^: 3472, 3337 (NH_2_); 1616 (C=O). ^1^H NMR (400 MHz, DMSO-*d*_6_): 1.61–1.68 (m, 2H, C(6)H_2_), 1.72–1.78 (m, 2H, C(7)H_2_), 2.40–2.44 (m, 2H, C(5)H_2_), 2.85-2.88 (m, 2H, C(8)H_2_), 6.11 (br. s, 2H, NH_2_), 7.21 (br. d, ^3^*J* = 2.7 Hz, 1H, H-4 thienyl), 7.29–7.35 (m, 5H, PhTz, H-3 thienyl), 7.56–7.59 (m, 2H, PhTz), 7.68–7.70 (m, 2H, PhTz), 7.92 (br. d, ^3^*J* = 4.7 Hz, 1H, H-5 thienyl). ^13^C DEPTQ NMR (101 MHz, DMSO-*d*_6_): 21.9 (CH_2_), 22.0 (CH_2_), 25.9 (CH_2_), 32.9 (CH_2_), 95.9 (C-2 ThQ), 120.3 (C-3a ThQ), 127.1 * (2 CH PhTz), 127.3 * (2 CH PhTz), 127.4 * (2 CH PhTz), 127.8 * (2 CH PhTz), 128.1 * (CH thienyl), 128.5 * (CH thienyl), 129.0 (C-4a ThQ), 129.1 * (CH thienyl), 132.4 (2C-S PhTz), 133.9 (C-2 thienyl), 138.4 (C-3 ThQ), 138.9 (2C-N PhTz), 149.9 (C-4 ThQ), 157.7 (C-8a ThQ), 159.4 (C-9a ThQ), 164.2 (C=O). * Negatively phased signals. Elemental Analysis: found, %: C, 65.68; H, 4.30; N, 8.37. C_28_H_21_N_3_OS_3_ (M 511.68). Calculated, %: C, 65.73; H, 4.14; N, 8.21.

*(3-Amino-4-(2-furyl)-5,6,7,8-tetrahydrothieno[2,3-b]quinolin-2-yl)(10H-phenothiazin-10-yl)methanone* **12h**. Yellow-brown solid, yield was 309 mg (52%, method A) and 339 mg (57%, method B). FTIR, ν_max_, cm^−1^: 3477, 3356 (NH_2_); 1620 (C=O). ^1^H NMR (400 MHz, DMSO-*d*_6_): 1.63–1.67 (m, 2H, C(6)H_2_), 1.74–1.78 (m, 2H, C(7)H_2_), 2.46–2.49 (m, 2H, C(5)H_2_), 2.86–2.89 (m, 2H, C(8)H_2_), 6.18 (br. s, 2H, NH_2_), 6.77–6.78 (m, 1H, H-4 furyl), 6.81 (br. d, ^3^*J* = 3.2 Hz, 1H, H-3 furyl), 7.29–7.35 (m, 4H, PhTz), 7.57–7.59 (m, 2H, PhTz), 7.69–7.72 (m, 2H, PhTz), 7.99–8.00 (m, 1H, H-5 furyl). ^13^C NMR (101 MHz, DMSO-*d*_6_): 21.9 (CH_2_), 22.0 (CH_2_), 26.0 (CH_2_), 32.9 (CH_2_), 96.6 (C-2 ThQ), 111.6 (CH-3 furyl), 112.4 (CH-4 furyl), 120.1 (C-3a ThQ), 127.1 * (2 CH PhTz), 127.3 * (2 CH PhTz), 127.4 * (2 CH PhTz), 127.8 * (2 CH PhTz), 129.0 (C-4a ThQ), 132.4 (2C-S PhTz), 133.9 (C-2 furyl), 138.9 (2C-N PhTz), 144.8 (C-3), 145.3 (CH-5 furyl), 149.4 (C-4 ThQ), 157.8 (C-8a ThQ), 159.7 (C-9a ThQ), 164.2 (C=O). Elemental Analysis: found, %: C, 67.80; H, 4.49; N, 8.62. C_28_H_21_N_3_O_2_S_2_ (M 495.62). Calculated, %: C, 67.86; H, 4.27; N, 8.48.

*(3-Amino-4,6-dimethylthieno[2,3-b]pyridin-2-yl)(10H-phenothiazin-10-yl)methanone* **12i**. Yellow solid, yield was 334 mg (69%, method A) and 324 mg (67%, method B). FTIR, ν_max_, cm^−1^: 3483, 3356 (NH_2_); 1597 (C=O). ^1^H NMR (400 MHz, DMSO-*d*_6_): 2.40 (s, 3H, CH_3–_6), 2.70 (s, 3H, CH_3–_4), 6.97 (s, 1H, H-5 ThPy **), 7.16 (br. s, 2H, NH_2_), 7.29–7.36 (m, 4H, PhTz), 7.57–7.59 (m, 2H, PhTz), 7.72–7.74 (m, 2H, PhTz). ^13^C DEPTQ NMR (101 MHz, DMSO-*d*_6_): 20.0 * (CH_3–_4), 23.8 * (CH_3–_6), 95.6 (C-2 ThPy), 121.3 (C-3a ThPy), 121.7 * (CH-5 ThPy), 127.0 * (2 CH PhTz), 127.2 * (2 CH PhTz), 127.4 * (2 CH PhTz), 127.8 * (2 CH PhTz), 132.3 (2C-S PhTz), 139.1 (2C-N PhTz), 144.8 (C-3 ThPy), 152.3 (C-4 ThPy), 159.6 (C-6 ThPy), 160.5 (C-7a ThPy), 164.6 (C=O). * Negatively phased signals. **Here and throughout the paper, ThPy = thieno[2,3-b]pyridine. Elemental Analysis: found, %: C, 65.56; H, 4.32; N, 10.36. C_22_H_17_N_3_OS_2_ (M 403.52). Calculated, %: C, 65.48; H, 4.25; N, 10.41.

*(3-Amino-4,5,6-trimethylthieno[2,3-b]pyridin-2-yl)(10H-phenothiazin-10-yl)methanone* **12j**. Yellow solid, yield was 341 mg (68%, method A) and 200 mg (40%, method B). FTIR, ν_max_, cm^−1^: 3508, 3342 (NH_2_); 1610 (C=O). ^1^H NMR (400 MHz, DMSO-*d*_6_): 2.17 (s, 3H, CH_3–_5), 2.41 (s, 3H, CH_3–_6), 2.64 (s, 3H, CH_3–_4), 7.23 (br. s, 2H, NH_2_), 7.27–7.35 (m, 4H, PhTz), 7.55–7.58 (m, 2H, PhTz), 7.71–7.73 (m, 2H, PhTz). ^13^C DEPTQ NMR (101 MHz, DMSO-*d*_6_): 14.4 * (CH_3–_5), 15.9 * (CH_3_-4), 24.0 * (CH_3_-6), 96.0 (C-2 ThPy), 121.8 (C-3a ThPy), 126.3 (C-5 ThPy), 126.9 * (2 CH PhTz), 127.1 * (2 CH PhTz), 127.4 * (2 CH PhTz), 127.8 * (2 CH PhTz), 132.3 (2C-S PhTz), 139.2 (2C-N PhTz), 142.6 (C-3 ThPy), 152.7 (C-4 ThPy), 157.4 (C-6 ThPy), 158.7 (C-7a ThPy), 164.8 (C=O). * Negatively phased signals. Elemental Analysis: found, %: C, 66.33; H, 4.64; N, 9.88. C_23_H_19_N_3_OS_2_ (M 417.55). Calculated, %: C, 66.16; H, 4.59; N, 10.06.

*(3-Amino-5-ethyl-4,6-dimethylthieno[2,3-b]pyridin-2-yl)(10H-phenothiazin-10-yl)methanone* **12k**. Yellow solid, yield was 352 mg (68%, method A) and 259 mg (50%, method B). FTIR, ν_max_, cm^−1^: 3497, 3325 (NH_2_); 1597 (C=O). ^1^H NMR (400 MHz, DMSO-*d*_6_): 1.02 (t, ^3^*J* = 7.3 Hz, 3H, CH_2_CH_3_), 2.45 (s, 3H, CH_3_-6), 2.65–2.69 (m, 5H, CH_2_CH_3_ and CH_3_-4 overlapped), 7.22 (br. s, 2H, NH_2_), 7.27–7.33 (m, 4H, PhTz), 7.55–7.58 (m, 2H, PhTz), 7.70–7.73 (m, 2H, PhTz). ^13^C DEPTQ NMR (101 MHz, DMSO-*d*_6_): 13.6 * (CH_3_CH_2_), 15.3 (CH_3_CH_2_), 21.2 * (CH_3_-4), 23.2 * (CH_3_-6), 96.0 (C-2 ThPy), 121.4 (C-3a ThPy), 122.2 (C-5 ThPy), 127.0 * (2 CH PhTz), 127.2 * (2 CH PhTz), 127.5 * (2 CH PhTz), 127.9 * (2 CH PhTz), 132.4 (2C-S PhTz), 139.2 (2C-N PhTz), 142.5 (C-3 ThPy), 152.8 (C-4 ThPy), 157.7 (C-6 ThPy), 158.5 (C-7a ThPy), 164.9 (C=O). * Negatively phased signals. Elemental Analysis: found, %: C, 66.70; H, 4.98; N, 9.63. C_24_H_21_N_3_OS_2_ (M 431.57). Calculated, %: C, 66.79; H, 4.90; N, 9.74.

*(3-Amino-6-methylthieno[2,3-b]pyridin-2-yl)(10H-phenothiazin-10-yl)methanone***12l**. Yellow-brown solid, yield was 378 mg (81%, method A) and 351 mg (75%, method B). FTIR, ν_max_, cm^−1^: 3449, 3306 (NH_2_); 1612 (C=O). ^1^H NMR (400 MHz, DMSO-*d*_6_): 2.48 (s, 3H, CH_3_-6), 7.24 (d, ^3^*J* = 8.3 Hz, 1H, H-5 ThPy), 7.29–7.37 (m, 4H, PhTz), 7.56–7.59 (m, 2H, PhTz), 7.64 (br. s, 2H, NH_2_), 7.72–7.75 (m, 2H, PhTz), 8.34 (d, ^3^*J* = 8.3 Hz, 1H, H-4 ThPy). ^13^C DEPTQ NMR (101 MHz, DMSO-*d*_6_): 24.2 * (CH_3_-6), 94.3 (C-2 ThPy), 119.3 * (CH-5 ThPy), 122.3 (C-3a ThPy), 127.0 * (2 CH PhTz), 127.3 * (2 CH PhTz), 127.4 * (2 CH PhTz), 127.8 * (2 CH PhTz), 131.1 * (CH-4 ThPy), 132.4 (2C-S PhTz), 139.1 (2C-N PhTz), 150.6 (C-3 ThPy), 159.8 (C-6 ThPy), 160.2 (C-7a ThPy), 164.3 (C=O). * Negatively phased signals. Elemental Analysis: found, %: C, 64.93; H, 4.03; N, 10.72. C_21_H_15_N_3_OS_2_ (M 389.49). Calculated, %: C, 64.76; H, 3.88; N, 10.79.

*(3-Amino-6,7-dihydro-5H-cyclopenta[b]thieno[3,2-e]pyridin-2-yl)(10H-phenothiazin-10-yl)methanone* **12m**. Yellow-brown solid, yield was 399 mg (80%, method A) and 359 mg (72%, method B). FTIR, ν_max_, cm^−1^: 3429, 3310 (NH_2_); 1601 (C=O). ^1^H NMR (400 MHz, DMSO-*d*_6_): 2.05–2.08 (m, 2H, C(6)H_2_), 2.89–2.92 (m, 4H, C(5)H_2_, C(7)H_2_), 7.29–7.35 (m, 4H, PhTz), 7.55–7.59 (m, 4H, PhTz, NH_2_), 7.72–7.74 (m, 2H, PhTz), 8.24 (s, 1H, H-4 ThPy). ^13^C DEPTQ NMR (101 MHz, DMSO-*d*_6_): 23.2 (CH_2_-6), 29.6 (CH_2_-5), 33.6 (CH_2_-7), 94.6 (C-2 ThPy), 122.7 (C-3a ThPy), 126.2 * (CH-4 ThPy), 126.9 * (2 CH PhTz), 127.3 * (2 CH PhTz), 127.4 * (2 CH PhTz), 127.8 * (2 CH PhTz), 132.3 (2C-S PhTz), 133.1 (C-4a ThPy), 139.1 (2C-N PhTz), 150.5 (C-3 ThPy), 158.7 (C-8a ThPy), 164.4 (C=O), 168.4 (C-7a ThPy). * Negatively phased signals. Elemental Analysis: found, %: C, 66.42; H, 4.23; N, 10.06. C_23_H_17_N_3_OS_2_ (M 415.53). Calculated, %: C, 66.48; H, 4.12; N, 10.11.

*(3-Amino-4,6-dimethylthieno[2,3-b]pyridin-2-yl)(3,7-dibromo-10H-phenothiazin-10-yl)methanone* **12n**. Yellow solid, yield was 525 mg (78%, method A) and 377 mg (56%, method B). FTIR, ν_max_, cm^−1^: 3452, 3337 (NH_2_); 1601 (C=O). ^1^H NMR (400 MHz, DMSO-*d*_6_): 2.41 (s, 3H, CH_3_-6), 2.69 (s, 3H, CH_3_-4), 6.98 (s, 1H, H-5 ThPy), 7.24 (br. s, 2H, NH_2_), 7.52 (dd, ^3^*J* = 8.7 Hz, ^3^*J* = 2.0 Hz, 2H, H-2 H-8 PhTz), 7.65 (d, ^3^*J* = 8.7 Hz, 2H, H-1 H-9 PhTz), 7.83 (d, ^3^*J* = 2.0 Hz, 2H, H-4 H-6 PhTz). ^13^C NMR (101 MHz, DMSO-*d*_6_): 20.1 (CH_3_-4), 23.8 (CH_3_-6), 94.7 (C-2 ThPy), 119.3 (2C, C-Br), 121.3 (C-3a ThPy), 121.9 (CH-5 ThPy), 128.5 (2 CH PhTz), 130.2 (2 CH PhTz), 130.5 (2 CH PhTz), 134.0 (2C-S PhTz), 138.3 (2C-N PhTz), 145.1 (C-3 ThPy), 152.9 (C-4 ThPy), 159.9 (C-6 ThPy), 160.5 (C-7a ThPy), 164.4 (C=O). Elemental Analysis: found, %: C, 47.30; H, 3.01; N, 7.69. C_22_H_15_Br_2_N_3_OS_2_ (M 561.31). Calculated, %: C, 47.08; H, 2.69; N, 7.49.

*2-Chloro-N-[4,6-dimethyl-2-(10H-phenothiazine-10-carbonyl)thieno[2,3-b]pyridin-3-yl]acetamide* **18**. A 50 mL round-bottom flask equipped with a reflux condenser and a calcium chloride tube was charged with 340 mg (0.84 mmol) of (3-amino-4,6-dimethylthieno[2,3- b]pyridin-2-yl)(10H-phenothiazin-10-yl)methanone **12i** and 20 mL of anhydrous chloroform. Then 0.1 mL (1.26 mmol) of chloroacetyl chloride was added to the resulting heterogeneous mixture. The mixture was then stirred under reflux for 3 h. Then chloroform was partially evaporated under vacuum, and the residue was treated with 20 mL of light petroleum. The solid was subsequently treated with an aqueous solution of NaHCO_3_, filtered off, washed with aqueous EtOH and petroleum ether, and dried under vacuum at ambient temperature. Off-white solid, yield was 290 mg (72%). FTIR, ν_max_, cm^−1^: 3143 (N–H); 1709, 1659 (2 C=O). ^1^H NMR (400 MHz, DMSO-*d*_6_): 2.46 (s, 3H, CH_3_-6), 2.65 (s, 3H, CH_3_-4), 4.43 (s, 2H, ClCH_2_), 7.11 (s, 1H, H-5 ThPy), 7.28–7.30 (m, 4H, PhTz), 7.59–7.61 (m, 2H, PhTz), 7.80–7.82 (m, 2H, PhTz), 10.47 (br. s, 1H, C(O)NH). ^13^C DEPTQ NMR (101 MHz, DMSO-*d*_6_): 18.9 * (CH_3_-4), 23.7 * (CH_3_-6), 42.9 (CH_2_Cl), 122.9 * (CH-5 ThPy), 124.8 (C-3a ThPy), 125.4 (C-2 ThPy), 127.1 * (2 CH PhTz), 127.3 * (4 CH PhTz), 127.7 * (2 CH PhTz), 130.5 (C-3 ThPy), 131.7 (2C-S PhTz), 137.9 (2C-N PhTz), 144.1 (C-4 ThPy), 158.1 (C-6 ThPy), 158.3 (C-7a ThPy), 160.3 (C=O), 166.0 (C=O). * Negatively phased signals. Elemental Analysis: found, %: C, 60.00; H, 3.89; N, 8.81. C_24_H_18_ClN_3_O_2_S_2_ (M 480.00). Calculated, %: C, 60.06; H, 3.78; N, 8.75.

*3-{4,6-Dimethyl-2-(10H-phenothiazine-10-carbonyl)thieno[2,3-b]pyridin-3-yl}-2-iminothiazolidin-4-one* **30**. A vial was charged with 240 mg (0.5 mmol) of chloroacetamide **18**, anhydrous DMF (5 mL), and an excess (100 mg, 1.03 mmol) of potassium thiocyanate. The resulted solution was vigorously stirred at 60 °C for 5 h. The reaction mixture was then cooled and diluted with cold water. The precipitate solid was filtered off after 12 h and dried under vacuum at 25 °C. Off-white solid, yield was 214 mg (85%). FTIR, ν_max_, cm^−1^: 3294 (N–H); 1720, 1659 (2 C=O), 1624 (C=N) (see also Table 1). ^1^H NMR (400 MHz, DMSO-*d*_6_): 2.42 (s, 3H, CH_3_), 2.49 (s, 3H, CH_3_, overlapped with the signal of DMSO), 4.42 (*AB*-pattern, ^2^*J* = 17.4 Hz, 2H, SCH_2_), 7.17 (s, 1H, H-5 ThPy), 7.30–7.34 (m, 4H, PhTz), 7.61–7.64 (m, 2H, PhTz), 7.75–7.77 (m, 2H, PhTz), 9.66 (s, 1H, C=NH). ^13^C DEPTQ NMR (101 MHz, DMSO-*d*_6_): 17.5 * (CH_3_-4), 23.8 * (CH_3_-6), 37.8 (CH_2_S), 123.1 * (CH-5 ThPy), 125.0 (C-2 ThPy), 127.0 * (2 CH PhTz), 127.5 * (2 CH PhTz), 127.7 * (2 CH PhTz), 127.9 * (2 CH PhTz), 129.3 (C-3a ThPy), 129.8 (C-3 ThPy), 131.8 (2C-S PhTz), 137.7 (2C-N PhTz), 143.2 (C-4 ThPy), 157.9 (C-6 ThPy), 158.3 (C-7a ThPy), 158.8 (C=NH), 159.0 (C=O), 171.9 (C=O thiazolidone). * Negatively phased signals. Elemental Analysis: found, %: C, 59.70; H, 3.71; N, 11.08. C_25_H_18_N_4_O_2_S_3_ (M 502.63). Calculated, %: C, 59.74; H, 3.61; N, 11.15.

**Quantum chemical studies**

Quantum chemical calculations of molecular geometry and vibrational frequencies were performed using the ORCA 6.0.1 software package [228,229]. These calculations employed the well-established hybrid functional B3LYP [230,231] with the D4 dispersion correction [232] in the def2-TZVP basis set [233]. A comparison of the calculated vibrational frequencies with experimental data was made using correction factors (0.9673 for high-frequency modes (>1800 cm^−1^) and 0.979 for lower-frequency modes (<1800 cm^−1^)) [214]. Molecular structures and vibrational frequencies were visualized using the ChemCraft 1.8 program. All calculations were performed following a preliminary search for the most stable conformers using the GOAT algorithm [234] with the semi-empirical GFN2-XTB method [235].

## 4. Conclusions

Thus, we have developed a method for the preparation of new heterodimeric molecules bearing the pharmacophoric fragments of 3-cyanoquinoline/3-aminothieno[2,3-b]pyridine (-quinoline) and phenothiazine. The proposed method is based on the S-alkylation reaction of readily available 2-thioxopyridine-3-carbonitriles or 2-thioxoquinoline-3-carbonitriles with N-(chloroacetyl)phenothiazines, followed by Thorpe–Ziegler cyclization.

We found that both reactions are accompanied by a previously unreported side reaction involving the elimination of the phenothiazine fragment. Under standard synthesis conditions (aqueous KOH, MeOH, or DMF), this side process reduces the yield and contaminates the products with unsubstituted phenothiazine. This phenothiazine elimination side reaction was minimized by carrying out the reaction under mild conditions (0–5 °C) and avoiding the use of nucleophilic bases and solvents (NaH or *tert-*BuONa, anhydrous DMAc).

We also demonstrated that the resulting heterodimeric 3-aminothienopyridines undergo acylation at the amino group. However, it was found that the reaction of the resulting (3-chloroacetamido-4,6-dimethylthieno[2,3-b]pyridin-2-yl)(10H-phenothiazin- 10-yl)methanone with potassium thiocyanate does not lead to phenothiazine elimination and the formation of the Gruner–Gewald rearrangement product. The structure and spectral data of the reaction product, 3-{4,6-dimethyl-2-(10H-phenothiazine-10-carbonyl) thieno[2,3-b]pyridin-3-yl}-2-iminothiazolidin-4-one, were studied using quantum chemical methods at the B3LYP-D4/def2-TZVP level of theory.

We also performed in silico studies of drug-relevant properties and ADMET parameters, which revealed that, in most cases, the prepared heterodimers do not meet the criteria for peroral bioavailability, primarily due to low solubility, which is consistent with experimental observations. Nevertheless, blind molecular docking of new compounds revealed the potential for further screening to identify new molecules with antitumor activity.

## Figures and Tables

**Table 1:** Comparison of experimental vibrational frequencies with the results of quantum chemical calculation for compound **30** (calculated at B3LYP-D4/def2-TZVP level of theory).

Vibrations	Experimental Bands, cm^−1^	Calculated Vibrational Frequencies, cm^−1^	
		No Correction Factors	With Correction Factors *
ν N-H	3294	3503	3388
ν C–H(Ar)	3063	3196	3091
ν_as_ CH_3_	2978	3113	3011
ν_s_ CH_3_	2935	3037	2938
ν C=O thiazolidinone	1720	1805	1746
ν C=O (amide I)	1659	1701	1665
ν C=N	1624	1672	1637
ν C–C(Ar) ThPy	1585	1622	1588
ν C–C(Ar) ThPy	1555	1596	1562
ν C–C(Ar) ThPy	1524	1558	1525
ν C–C(Ar) + δ C–H(Ar) PhTz	1458	1502	1470
ν C-N + δ CH_3_	1396	1423	1393
Skeletal	1331	1367	1338
Skeletal	1261	1299	1272
Skeletal	1211	1233	1207
δ N-H + δ C–H(Ar)	1126	1169	1144
δ C–H(Ar)	1030	1060	1038
Skeletal	918	938	918
Skeletal	895	907	888
ν C-S PhTz	868	878	860
δ N-H	787	795	778
δ C–H(Ar)	764	777	761
Skeletal	725	738	723
Skeletal	613	633	620
MAPE **, %	-	2.83	0.77

* The correction factors were 0.9673 for high-frequency vibrations (>1800 cm^−1^) and 0.979 for low-frequency vibrations (<1800 cm^−1^) according to [214]. ** MAPE stands for Mean Absolute Percentage Error.

## Data Availability

File Electronic Supplementary Material.pdf containing X-ray data, ^1^H and ^13^C DEPTQ NMR, FTIR spectral charts (Figures S1–S70, Tables S1–S10).

## Supplementary Materials

The following supporting information can be downloaded at https://www.mdpi.com/article/10.3390/ijms26199798/s1: Figures S1–S70, Tables S1–S10 with FTIR, ^1^H, ^13^C DEPTQ NMR spectra of starting compounds and new phenothiazine heterodimers, predicted ADMET parameters and docking studies results as well as X-ray data for molecule **10b**.
