# Supplementary material for: Synthesis of New Phenothiazine/3-cyanoquinoline and Phenothiazine/3-aminothieno[2,3-b]pyridine(-quinoline) Heterodimers
Source: Int J Mol Sci. 2025 Oct 8;26(19):9798. doi: 10.3390/ijms26199798 (PMC12524799; doi:10.3390/ijms26199798)
Supplement: Supplementary file 1 [file ijms-26-09798-s001.zip › ijms-3903977-supplementary.pdf]

Electronic Supplementary Material file

Synthesis of new phenothiazine/3-cyanoquinoline and phenothiazine/3-aminothieno[2,3-b]pyridine (-quinoline) heterodimers

Victor V. Dotsenko[1,2,]*, Vladislav K. Kindop[1], Vyacheslav K. Kindop[1], Eva S. Daus[1], Igor V. Yudaev[3], Yulia V. Daus[3], Alexander V. Bespalov[1], Dmitrii S. Buryi[1], Darya Yu. Lukina[1], Nicolai A. Aksenov[2] and Inna V. Aksenova [2]

[1] Department of Organic Chemistry and Technologies, Kuban State University, 149 Stavropolskaya St., 350040 Krasnodar, Russia; victor_dotsenko_@mail.ru
[2] Department of Chemistry, North Caucasus Federal University, 1 Pushkin St., 355017 Stavropol, Russia
[3] Faculty of Energetics, Kuban State Agrarian University, 13 Kalinina St., 350044 Krasnodar, Russia
*****Correspondence: victor_dotsenko_@mail.ru (V.V.D.).

Table of contents (selected spectral and X-ray data)

Figure S1. FTIR spectrum of N-(chloroacetyl)phenothiazine 10a 5
Figure S2. ^{1}H NMR spectrum of N-(chloroacetyl)phenothiazine 10a, DMSO-d_6 (400 MHz).......... 5
Figure S3. ^{13}C DEPTQ NMR spectrum of N-(chloroacetyl)phenothiazine 10a, DMSO-d_6 (101 MHz) 6
Figure S4. ^{1}H NMR spectrum of 3,7-dibromo-N-(chloroacetyl)phenothiazine 10b, DMSO-d_6 (400 MHz) 6
Figure S5. ^{13}C DEPTQ NMR spectrum of 3,7-dibromo-N-(chloroacetyl)phenothiazine 10b, DMSO-d_6 (101 MHz) 7
Figure S6. ORTEP drawing of X-ray structure for 3,7-dibromo-10-(chloroacetyl)phenothiazine 10b with 50% probability (CCDC deposition number 2478604). 7
Table S1 Crystal data and structure refinement for 3,7-dibromo-10-(chloroacetyl)phenothiazine 10b.......... 8
Table S2 Fractional Atomic Coordinates ($\times10^4$) and Equivalent Isotropic Displacement Parameters ($Å^2\times10^3$) for 3,7-dibromo-10-(chloroacetyl)phenothiazine 10b 9
Table S3 Anisotropic Displacement Parameters ($Å^2\times10^3$) for 3,7-dibromo-10-(chloroacetyl)phenothiazine 10b.......... 11
Table S4 Bond Lengths for 3,7-dibromo-10-(chloroacetyl)phenothiazine 10b.......... 13
Table S5 Bond Angles for 3,7-dibromo-10-(chloroacetyl)phenothiazine 10b. 14
Table S6 Torsion Angles for 3,7-dibromo-10-(chloroacetyl)phenothiazine 10b. 15
Table S7 Hydrogen Atom Coordinates ($Å\times10^4$) and Isotropic Displacement Parameters ($Å^2\times10^3$) for 3,7-dibromo-10-(chloroacetyl)phenothiazine 10b.......... 16
Figure S7. FTIR spectrum of 4-(4-chlorophenyl)-2-thioxo-1,2,5,6,7,8-hexahydroquinoline-3-carbonitrile 9b 17
Figure S8. ^{1}H NMR spectrum (400 MHz, DMSO-d_6) of 4-(4-chlorophenyl)-2-thioxo-1,2,5,6,7,8-hexahydroquinoline-3-carbonitrile 9b.......... 18
Figure S9. ^{13}C DEPTQ NMR spectrum (101 MHz, DMSO-d_6) of 4-(4-chlorophenyl)-2-thioxo-1,2,5,6,7,8-hexahydroquinoline-3-carbonitrile 9b 18
Figure S10. ^{1}H NMR spectrum (400 MHz, DMSO-d_6) of 4-(3-bromophenyl)-2-thioxo-1,2,5,6,7,8-hexahydroquinoline-3-carbonitrile 9c.......... 19

Figure S11. ^{13}C DEPTQ NMR spectrum (101 MHz, DMSO-d_6) of 4-(3-bromophenyl)-2-thioxo-1,2,5,6,7,8-hexahydroquinoline-3-carbonitrile 9c 19
Figure S12. ^{1}H NMR spectrum (400 MHz, DMSO-d_6) of 4-(4-fluorophenyl)-2-thioxo-1,2,5,6,7,8-hexahydroquinoline-3-carbonitrile 9d 20
Figure S13. ^{13}C DEPTQ NMR spectrum (101 MHz, DMSO-d_6) of 4-(4-fluorophenyl)-2-thioxo-1,2,5,6,7,8-hexahydroquinoline-3-carbonitrile 9d 20
Figure S14. FTIR spectrum of 4-(2-thienyl)-2-thioxo-1,2,5,6,7,8-hexahydroquinoline-3-carbonitrile 9e 21
Figure S15. ^{1}H NMR spectrum (400 MHz, DMSO-d_6) of 4-(2-thienyl)-2-thioxo-1,2,5,6,7,8-hexahydroquinoline-3-carbonitrile 9e 21
Figure S16. ^{13}C DEPTQ NMR spectrum (101 MHz, DMSO-d_6) of 4-(2-thienyl)-2-thioxo-1,2,5,6,7,8-hexahydroquinoline-3-carbonitrile 9e 22
Figure S17. ^{1}H NMR spectrum (400 MHz, DMSO-d_6) of 4-(2,4-dichlorophenyl)-2-thioxo-1,2,5,6,7,8-hexahydroquinoline-3-carbonitrile 9f 22
Figure S18. ^{13}C DEPTQ NMR spectrum (101 MHz, DMSO-d_6) of 4-(2,4-dichlorophenyl)-2-thioxo-1,2,5,6,7,8-hexahydroquinoline-3-carbonitrile 9f 23
Figure S19. FTIR spectrum of 4-(3-nitrophenyl)-2-thioxo-1,2,5,6,7,8-hexahydroquinoline-3-carbonitrile 9h 23
Figure S20. ^{1}H NMR spectrum (400 MHz, DMSO-d_6) of 4-(3-nitrophenyl)-2-thioxo-1,2,5,6,7,8-hexahydroquinoline-3-carbonitrile 9h 24
Figure S21. ^{13}C DEPTQ NMR spectrum (101 MHz, DMSO-d_6) of 4-(3-nitrophenyl)-2-thioxo-1,2,5,6,7,8-hexahydroquinoline-3-carbonitrile 9h 24
Figure S22. ^{1}H NMR spectrum (400 MHz, DMSO-d_6) of 4-(4-chlorophenyl)-2-{[2-oxo-2-(10H-phenothiazin-10-yl)ethyl]thio}-5,6,7,8-tetrahydroquinoline-3-carbonitrile 11a 25
Figure S23. ^{13}C DEPTQ NMR spectrum (101 MHz, DMSO-d_6) of 4-(4-chlorophenyl)-2-{[2-oxo-2-(10H-phenothiazin-10-yl)ethyl]thio}-5,6,7,8-tetrahydroquinoline-3-carbonitrile 11a 25
Figure S24. FTIR spectrum of 4-(3-bromophenyl)-2-{[2-oxo-2-(10H-phenothiazin-10-yl)ethyl]thio}-5,6,7,8-tetrahydroquinoline-3-carbonitrile 11b 26
Figure S25. ^{1}H NMR spectrum (400 MHz, DMSO-d_6) of 4-(3-bromophenyl)-2-{[2-oxo-2-(10H-phenothiazin-10-yl)ethyl]thio}-5,6,7,8-tetrahydroquinoline-3-carbonitrile 11b 26
Figure S26. ^{13}C DEPTQ NMR spectrum (101 MHz, DMSO-d_6) of 4-(3-bromophenyl)-2-{[2-oxo-2-(10H-phenothiazin-10-yl)ethyl]thio}-5,6,7,8-tetrahydroquinoline-3-carbonitrile 11b 27
Figure S27. ^{1}H NMR spectrum (400 MHz, DMSO-d_6) of 4-(4-fluorophenyl)-2-{[2-oxo-2-(10H-phenothiazin-10-yl)ethyl]thio}-5,6,7,8-tetrahydroquinoline-3-carbonitrile 11c 27
Figure S28. ^{13}C DEPTQ NMR spectrum (101 MHz, DMSO-d_6) of 4-(4-fluorophenyl)-2-{[2-oxo-2-(10H-phenothiazin-10-yl)ethyl]thio}-5,6,7,8-tetrahydroquinoline-3-carbonitrile 11c 28
Figure S29. FTIR spectrum of 4-(2-thienyl)-2-{[2-oxo-2-(10H-phenothiazin-10-yl)ethyl]thio}-5,6,7,8-tetrahydroquinoline-3-carbonitrile 11d 28
Figure S30. ^{1}H NMR spectrum (400 MHz, DMSO-d_6) of 4-(2-thienyl)-2-{[2-oxo-2-(10H-phenothiazin-10-yl)ethyl]thio}-5,6,7,8-tetrahydroquinoline-3-carbonitrile 11d 29
Figure S31. ^{13}C DEPTQ NMR spectrum (101 MHz, DMSO-d_6) of 4-(2-thienyl)-2-{[2-oxo-2-(10H-phenothiazin-10-yl)ethyl]thio}-5,6,7,8-tetrahydroquinoline-3-carbonitrile 11d 29
Figure S32. FTIR spectrum of 4-(2,4-dichlorophenyl)-2-{[2-oxo-2-(10H-phenothiazin-10-yl)ethyl]thio}-5,6,7,8-tetrahydroquinoline-3-carbonitrile 11e 30
Figure S33. ^{1}H NMR spectrum (400 MHz, DMSO-d_6) of 4-(2,4-dichlorophenyl)-2-{[2-oxo-2-(10H-phenothiazin-10-yl)ethyl]thio}-5,6,7,8-tetrahydroquinoline-3-carbonitrile 11e 30
Figure S34. ^{13}C DEPTQ NMR spectrum (101 MHz, DMSO-d_6) of 4-(2,4-dichlorophenyl)-2-{[2-oxo-2-(10H-phenothiazin-10-yl)ethyl]thio}-5,6,7,8-tetrahydroquinoline-3-carbonitrile 11e 31
Figure S35. ^{1}H NMR spectrum (400 MHz, DMSO-d_6) of 4-(4-methylphenyl)-2-{[2-oxo-2-(10H-phenothiazin-10-yl)ethyl]thio}-5,6,7,8-tetrahydroquinoline-3-carbonitrile 11f 31
Figure S36. ^{13}C DEPTQ NMR spectrum (101 MHz, DMSO-d_6) of 4-(4-methylphenyl)-2-{[2-oxo-2-(10H-phenothiazin-10-yl)ethyl]thio}-5,6,7,8-tetrahydroquinoline-3-carbonitrile 11f 32

Figure S37. ^{1}H NMR spectrum (400 MHz, DMSO-d_6) of 4-(3-nitrophenyl)-2-{[2-oxo-2-(10H-phenothiazin-10-yl)ethyl]thio}-5,6,7,8-tetrahydroquinoline-3-carbonitrile 11g 32
Figure S38. ^{13}C DEPTQ NMR spectrum (101 MHz, DMSO-d_6) of 4-(3-nitrophenyl)-2-{[2-oxo-2-(10H-phenothiazin-10-yl)ethyl]thio}-5,6,7,8-tetrahydroquinoline-3-carbonitrile 11g..... 33
Figure S39. FTIR spectrum of 4-(2-furyl)-2-{[2-oxo-2-(10H-phenothiazin-10-yl)ethyl]thio}-5,6,7,8-tetrahydroquinoline-3-carbonitrile 11h..... 33
Figure S40. ^{1}H NMR spectrum (400 MHz, DMSO-d_6) of 4-(2-furyl)-2-{[2-oxo-2-(10H-phenothiazin-10-yl)ethyl]thio}-5,6,7,8-tetrahydroquinoline-3-carbonitrile 11h 34
Figure S41. ^{13}C DEPTQ NMR spectrum (101 MHz, DMSO-d_6) of 4-(2-furyl)-2-{[2-oxo-2-(10H-phenothiazin-10-yl)ethyl]thio}-5,6,7,8-tetrahydroquinoline-3-carbonitrile 11h 34
Figure S42. ^{1}H NMR spectrum (400 MHz, DMSO-d_6) of phenothiazine (PhTz) 35
Figure S43. ^{13}C DEPTQ NMR spectrum (101 MHz, DMSO-d_6) of phenothiazine(PhTz) 35
Figure S44. FTIR spectrum of (3-amino-4-(4-chlorophenyl)-5,6,7,8-tetrahydrothieno[2,3-b]quinolin-2-yl)(10H-phenothiazin-10-yl)methanone 12a 36
Figure S45. ^{1}H NMR spectrum (400 MHz, DMSO-d_6) of crude (3-amino-4-(4-chlorophenyl)-5,6,7,8-tetrahydrothieno[2,3-b]quinolin-2-yl)(10H-phenothiazin-10-yl)methanone 12a..... 36
Figure S46. ^{13}C DEPTQ NMR spectrum (101 MHz, DMSO-d_6) of crude (3-amino-4-(4-chlorophenyl)-5,6,7,8-tetrahydrothieno[2,3-b]quinolin-2-yl)(10H-phenothiazin-10-yl)methanone 12a..... 37
Figure S47. FTIR spectrum of (3-amino-4-(3-bromophenyl)-5,6,7,8-tetrahydrothieno[2,3-b]quinolin-2-yl)(10H-phenothiazin-10-yl)methanone 12b 37
Figure S48. ^{1}H NMR spectrum (400 MHz, DMSO-d_6) of crude (3-amino-4-(3-bromophenyl)-5,6,7,8-tetrahydrothieno[2,3-b]quinolin-2-yl)(10H-phenothiazin-10-yl)methanone 12b..... 38
Figure S49. ^{13}C DEPTQ NMR spectrum (101 MHz, DMSO-d_6) of crude (3-amino-4-(3-bromophenyl)-5,6,7,8-tetrahydrothieno[2,3-b]quinolin-2-yl)(10H-phenothiazin-10-yl)methanone 12b..... 38
Figure S50. FTIR spectrum of (3-amino-4-(2-thienyl)-5,6,7,8-tetrahydrothieno[2,3-b]quinolin-2-yl)(10H-phenothiazin-10-yl)methanone 12d 39
Figure S51. ^{1}H NMR spectrum (400 MHz, DMSO-d_6) of crude (3-amino-4-(2-thienyl)-5,6,7,8-tetrahydrothieno[2,3-b]quinolin-2-yl)(10H-phenothiazin-10-yl)methanone 12d..... 39
Figure S52. ^{13}C DEPTQ NMR spectrum (101 MHz, DMSO-d_6) of crude (3-amino-4-(2-thienyl)-5,6,7,8-tetrahydrothieno[2,3-b]quinolin-2-yl)(10H-phenothiazin-10-yl)methanone 12d..... 40
Figure S53. FTIR spectrum of (3-amino-4,6-dimethylthieno[2,3-b]pyridin-2-yl)(10H-phenothiazin-10-yl)methanone 12i. 40
Figure S54. ^{1}H NMR spectrum (400 MHz, DMSO-d_6) of (3-amino-4,6-dimethylthieno[2,3-b]pyridin-2-yl)(10H-phenothiazin-10-yl)methanone 12i..... 41
Figure S55. ^{13}C DEPTQ NMR spectrum (101 MHz, DMSO-d_6) of (3-amino-4,6-dimethylthieno[2,3-b]pyridin-2-yl)(10H-phenothiazin-10-yl)methanone 12i..... 41
Figure S56. FTIR spectrum (nujol) of (3-amino-6-methylthieno[2,3-b]pyridin-2-yl)(10H-phenothiazin-10-yl)methanone 12k 42
Figure S57. ^{1}H NMR spectrum (400 MHz, DMSO-d_6) of (3-amino-6-methylthieno[2,3-b]pyridin-2-yl)(10H-phenothiazin-10-yl)methanone 12k 42
Figure S58. ^{13}C DEPTQ NMR spectrum (101 MHz, DMSO-d_6) of (3-amino-6-methylthieno[2,3-b]pyridin-2-yl)(10H-phenothiazin-10-yl)methanone 12k..... 43
Figure S59. FTIR spectrum (nujol) of (3-amino-6-methylthieno[2,3-b]pyridin-2-yl)(10H-phenothiazin-10-yl)methanone 12m 43
Figure S60. ^{1}H NMR spectrum (400 MHz, DMSO-d_6) of (3-amino-6-methylthieno[2,3-b]pyridin-2-yl)(10H-phenothiazin-10-yl)methanone 12m 44
Figure S61. ^{13}C DEPTQ NMR spectrum (101 MHz, DMSO-d_6) of (3-amino-6-methylthieno[2,3-b]pyridin-2-yl)(10H-phenothiazin-10-yl)methanone 12m..... 44
Figure S62. FTIR spectrum (nujol) of (3-amino-4,6-dimethylthieno[2,3-b]pyridin-2-yl)(3,7-dibromo-10H-phenothiazin-10-yl)methanone 12n 45

Figure S63. ^{1}H NMR spectrum (400 MHz, DMSO-d_6) of (3-amino-4,6-dimethylthieno[2,3-b]pyridin-2-yl)(3,7-dibromo-10H-phenothiazin-10-yl)methanone 12n 45
Figure S64. ^{13}C NMR spectrum (101 MHz, DMSO-d_6) of (3-amino-4,6-dimethylthieno[2,3-b]pyridin-2-yl)(3,7-dibromo-10H-phenothiazin-10-yl)methanone 12n 46
Figure S65. ^{1}H NMR spectrum (400 MHz, DMSO-d_6) of 2-chloro-N-[4,6-dimethyl-2-(10H-phenothiazine-10-carbonyl)thieno[2,3-b]pyridin-3-yl]acetamide 18 46
Figure S66. ^{13}C DEPTQ NMR spectrum (101 MHz, DMSO-d_6) of 2-chloro-N-[4,6-dimethyl-2-(10H-phenothiazine-10-carbonyl)thieno[2,3-b]pyridin-3-yl]acetamide 18 47
Figure S67. FTIR spectrum (nujol) of 3-{4,6-dimethyl-2-(10H-phenothiazine-10-carbonyl)thieno[2,3-b]pyridin-3-yl}-2-iminothiazolidin-4-one 30 47
Figure S68. ^{1}H NMR spectrum (400 MHz, DMSO-d_6) of 3-{4,6-dimethyl-2-(10H-phenothiazine-10-carbonyl)thieno[2,3-b]pyridin-3-yl}-2-iminothiazolidin-4-one 30 48
Figure S69. ^{13}C DEPTQ NMR spectrum (101 MHz, DMSO-d_6) of 3-{4,6-dimethyl-2-(10H-phenothiazine-10-carbonyl)thieno[2,3-b]pyridin-3-yl}-2-iminothiazolidin-4-one 30 48
Figure S70. Comparison of the IR spectra of compound 30: experimental (KBr pellets) (top) and the IR spectrum calculated at the B3LYP-D4/def2-TZVP level (bottom). 49
Table S9. Predicted ADMET parameters for molecules 11a-h, 12a-n, 18 and 30 53
Table S10. Prediction of the protein-ligand interaction for molecules 11a-h, 12a-n, 18, and 30 using the GalaxyWeb Sagittarius molecular docking protocol 54

Figure S1. FTIR spectrum of N-(chloroacetyl)phenothiazine 10a

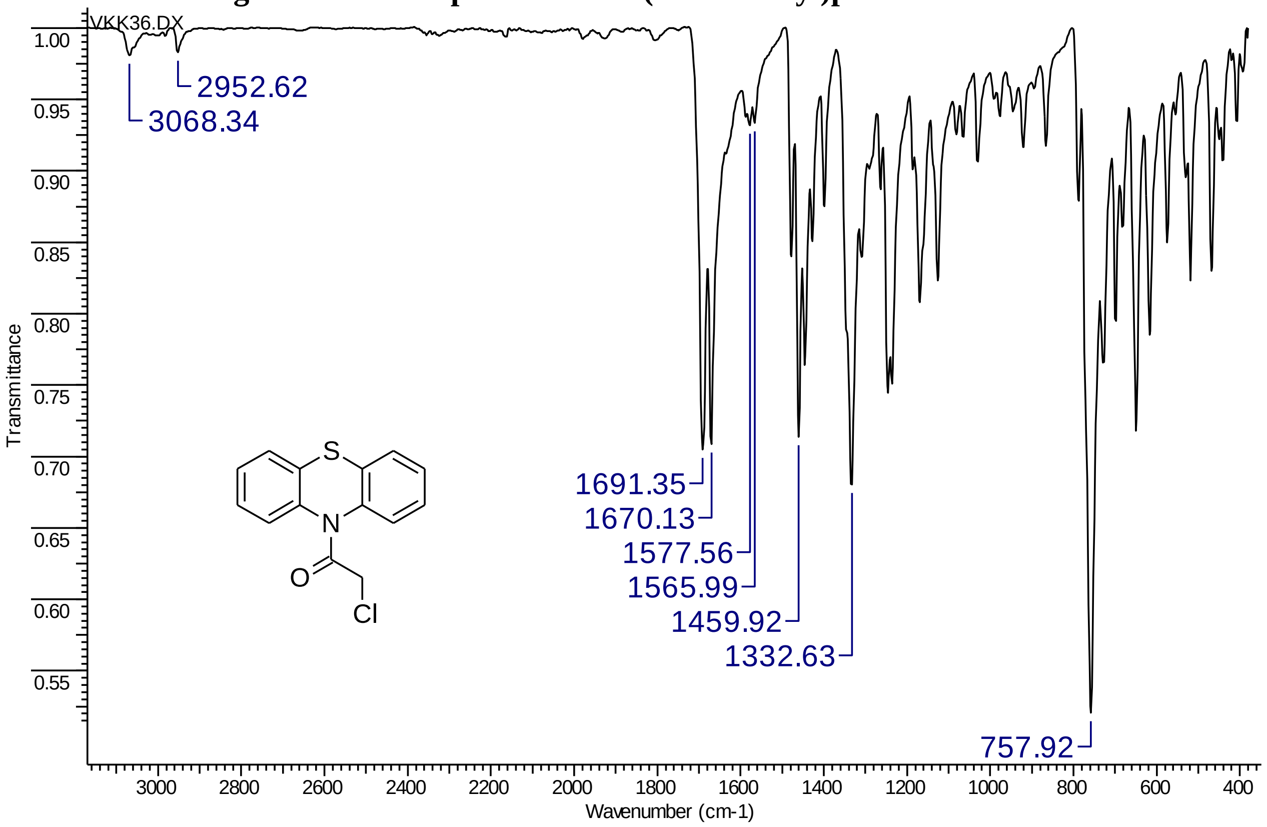

Figure S2. ^{1}H NMR spectrum of N-(chloroacetyl)phenothiazine 10a, DMSO-d_6 (400 MHz)

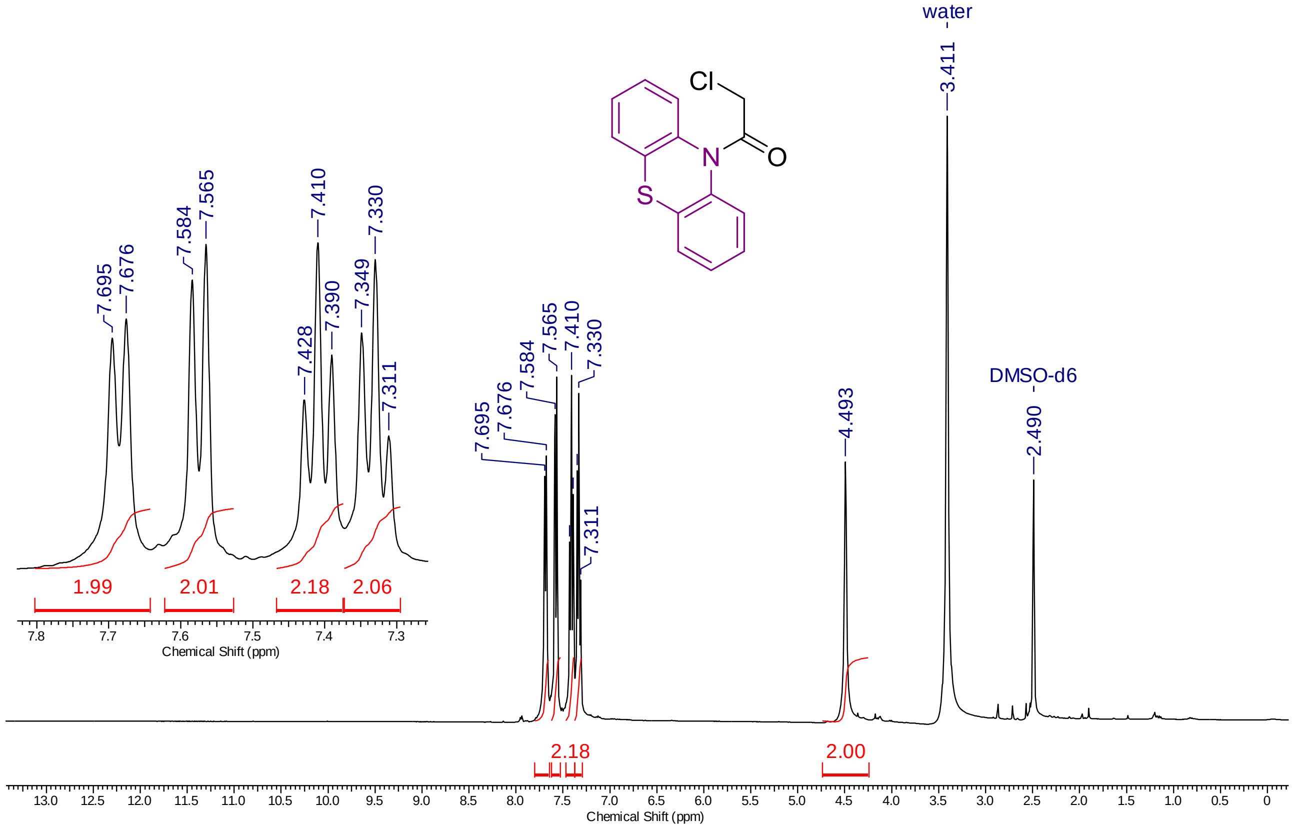

Figure S3. ^{13}**C DEPTQ NMR spectrum of N-(chloroacetyl)phenothiazine 10a, DMSO-d$_6$ (101 MHz)**

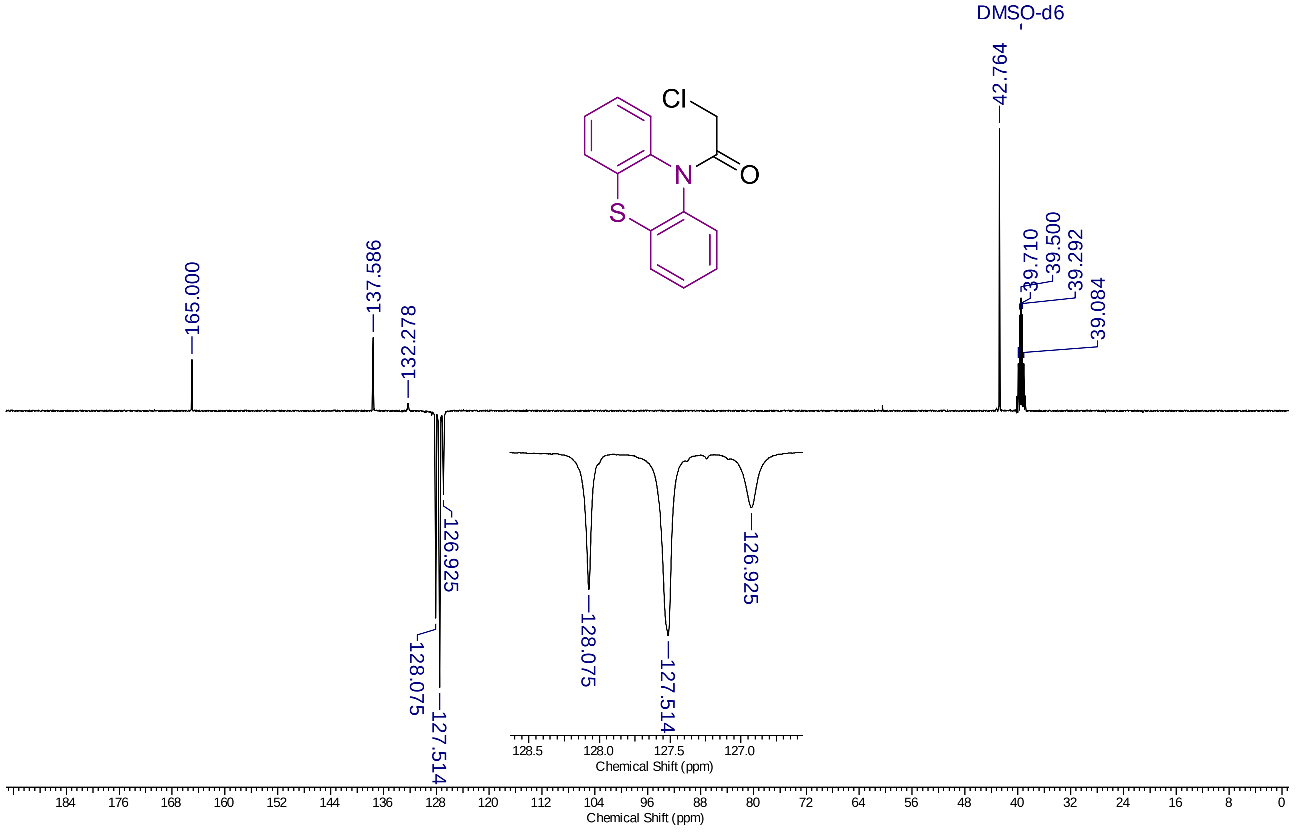

Figure S4. ^{1}**H NMR spectrum of 3,7-dibromo-N-(chloroacetyl)phenothiazine 10b, DMSO-d$_6$ (400 MHz)**

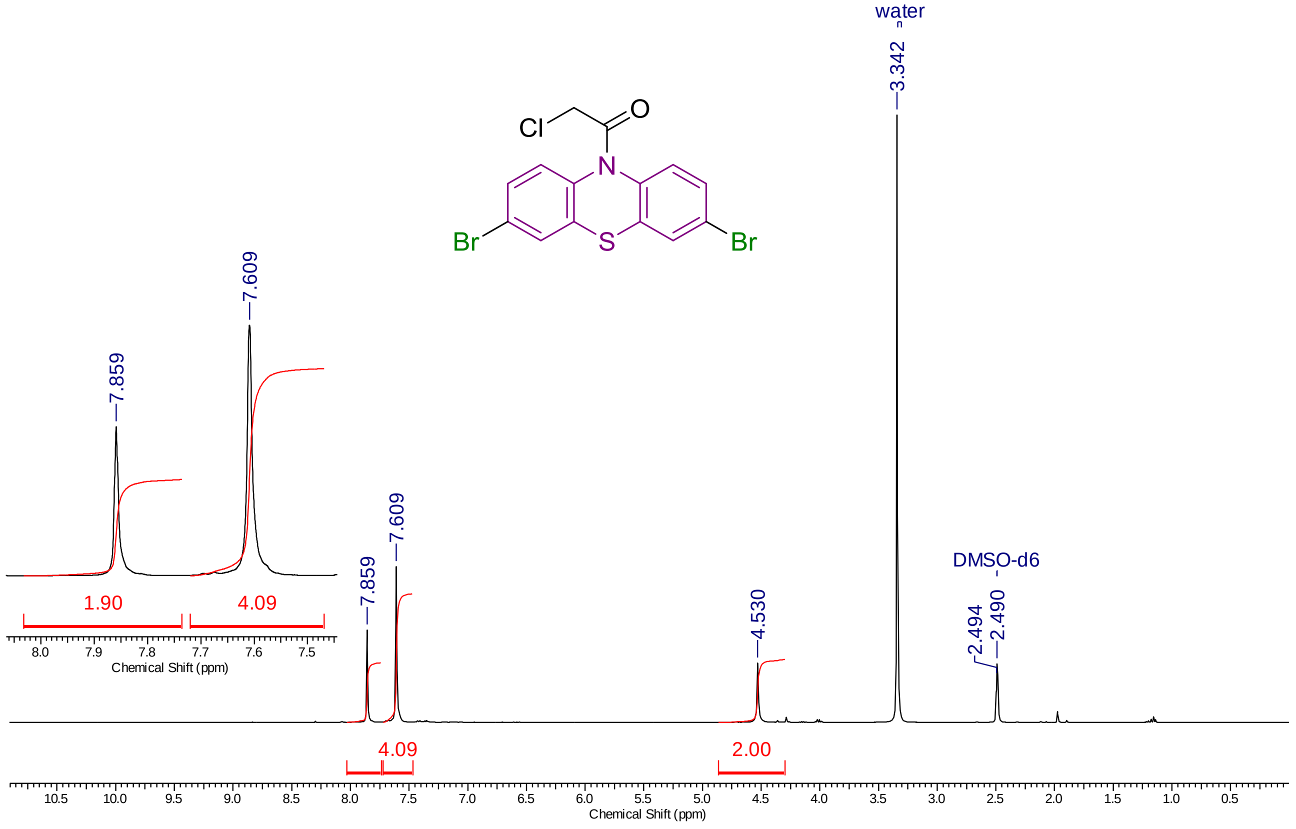

Figure S5. ^{13}C DEPTQ NMR spectrum of 3,7-dibromo-N-(chloroacetyl)phenothiazine 10b, DMSO-d$_6$ (101 MHz)

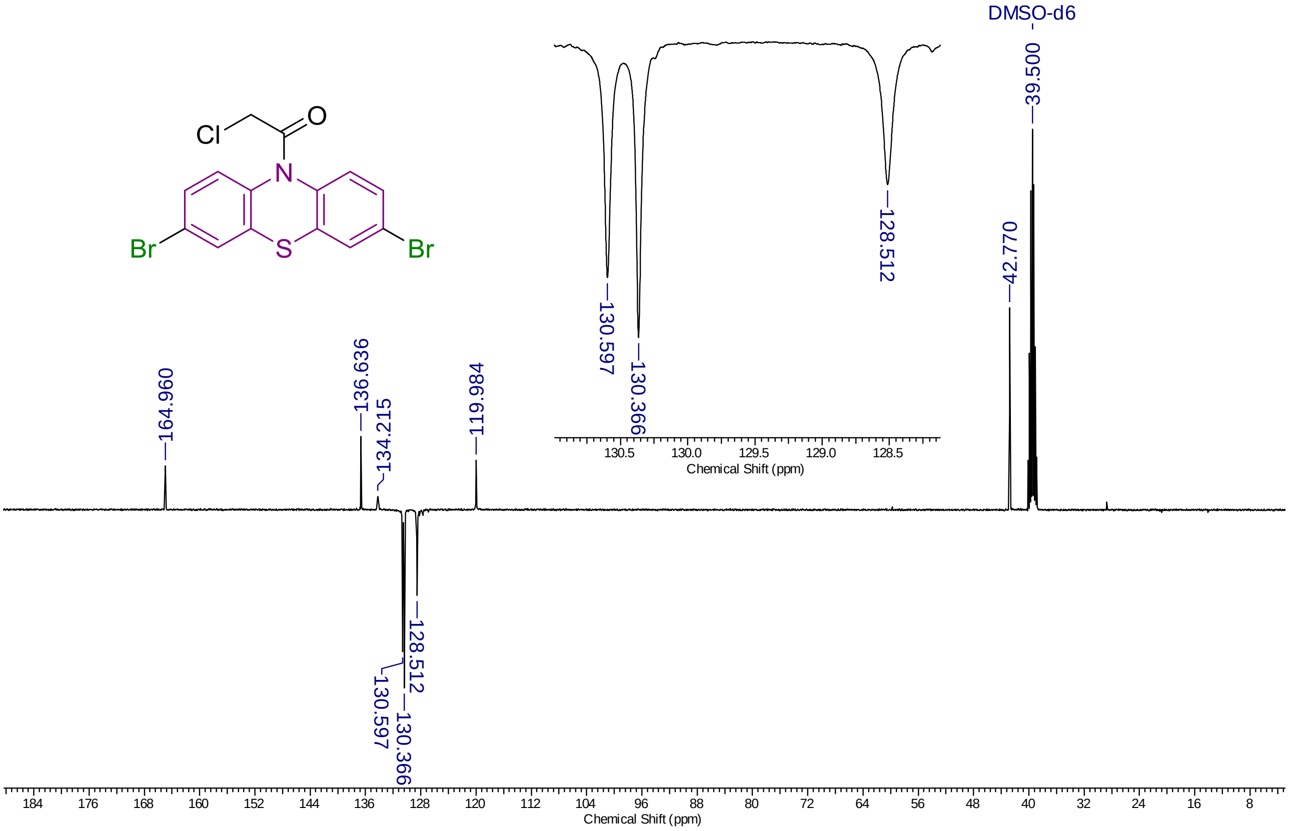

Figure S6. ORTEP drawing of X-ray structure for 3,7-dibromo-10-(chloroacetyl)phenothiazine 10b with 50% probability (CCDC deposition number 2478604).

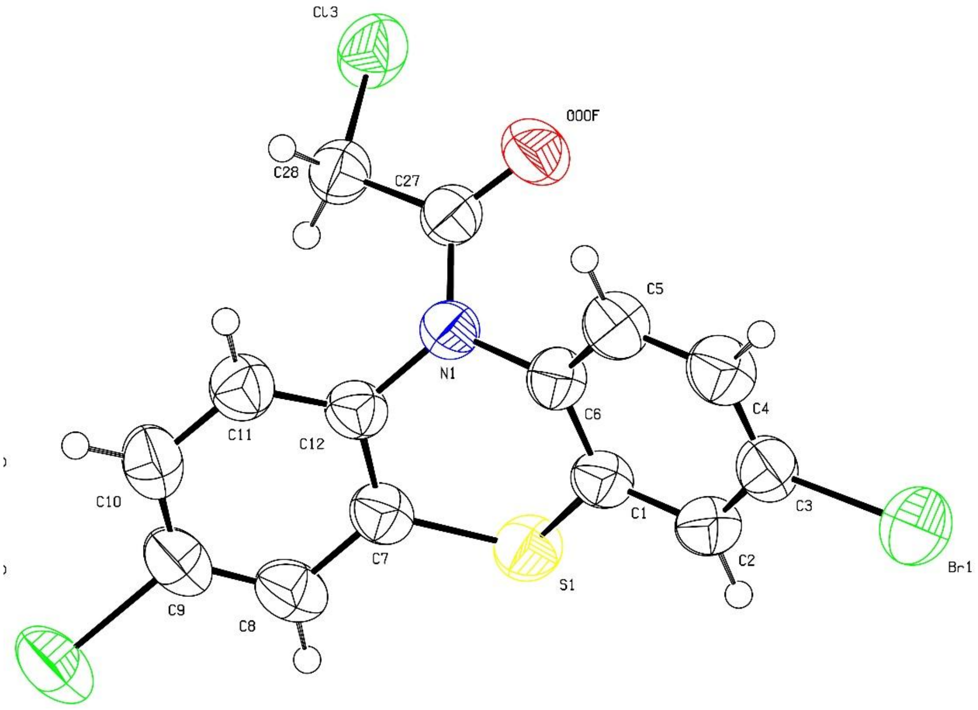

Table S1 Crystal data and structure refinement for 3,7-dibromo-10-(chloroacetyl)phenothiazine 10b.

Identification code	**10b**
Empirical formula	$C_{14}H_8Br_2ClNOS$
Formula weight	433.54
Temperature/K	293(2)
Crystal system	monoclinic
Space group	$P2_1/c$
a/Å	8.14000(10)
b/Å	13.3616(2)
c/Å	41.2690(5)
α/°	90
β/°	90.0560(10)
γ/°	90
Volume/Å^3	4488.56(10)
Z	12
ρ_{calc}g/cm^3	1.925
μ/mm^{-1}	9.772
F(000)	2520.0
Crystal size/mm^3	0.325 × 0.245 × 0.151
Radiation	Cu Kα (λ = 1.54184)
2Θ range for data collection/°	6.954 to 134.146
Index ranges	$-8 \le h \le 9$, $-15 \le k \le 15$, $-49 \le l \le 49$
Reflections collected	23363
Independent reflections	7904 [R_{int} = 0.0302, R_{sigma} = 0.0301]
Data/restraints/parameters	7904/0/541
Goodness-of-fit on F^2	1.086
Final R indexes [I>=2σ (I)]	R_1 = 0.0468, wR_2 = 0.1232
Final R indexes [all data]	R_1 = 0.0521, wR_2 = 0.1275
Largest diff. peak/hole / e Å^{-3}	0.93/-1.02

Table S2 Fractional Atomic Coordinates (×10^4) and Equivalent Isotropic Displacement Parameters (Å^2×10^3) for 3,7-dibromo-10-(chloroacetyl)phenothiazine 10b. U_{eq} is defined as 1/3 of of the trace of the orthogonalised U_{IJ} tensor.

Atom	*x*	*y*	*z*	U(eq)
Br6	9986.1(8)	4131.4(5)	1805.3(2)	75.85(18)
Br3	3448.2(8)	3341.2(4)	5402.4(2)	72.06(17)
Br2	2720.4(8)	10952.0(4)	5114.8(2)	77.59(19)
Br7	6524.1(9)	8973.1(6)	1896.9(2)	86.1(2)
Br4	11070.4(9)	951.2(6)	4053.4(2)	91.4(2)
Br1	5793.6(11)	12591.3(5)	4150.3(2)	92.0(2)
S2	7887.2(15)	2486.2(9)	2969.9(3)	55.8(3)
S3	490.8(14)	7032.1(9)	5211.3(3)	54.5(3)
S1	3939.1(17)	10716.2(9)	3004.5(3)	60.8(3)
Cl1	-1610.4(16)	7415.0(11)	3828.2(3)	70.3(3)
Cl3	1786.7(16)	6601.2(10)	3376.1(4)	71.8(3)
Cl2	5057.9(18)	5504.8(13)	3796.1(3)	79.0(4)
O00D	306(4)	5878(3)	4158.9(8)	60.1(8)
O5	6948(5)	5787(3)	3197.9(8)	63.8(9)
O00F	3553(5)	8114(3)	3742.1(8)	69.1(9)
N2	8650(5)	4462(3)	3249.9(8)	48.0(8)
N1	4983(5)	8837(3)	3333.9(9)	50.0(8)
N3	1607(5)	6832(3)	4533.5(8)	49.0(8)
C14	8961(5)	4415(3)	2907.5(10)	46.4(9)
C15	8694(5)	3509(3)	2751.7(10)	46.5(9)
C40	2031(5)	5985(3)	4728.4(10)	47.7(9)
C19	9292(5)	3649(3)	3436.7(10)	47.4(9)
C41	649(5)	6697(4)	4261.4(10)	50.6(10)
C35	1605(5)	6011(3)	5053.3(10)	46.1(9)
C17	9556(6)	4252(4)	2255.1(10)	53.4(10)
C16	9018(6)	3419(4)	2421.7(11)	52.0(10)
C29	1958(5)	7792(3)	4669.5(11)	48.2(9)
C6	5262(5)	9721(3)	3523.1(11)	49.6(9)
C12	5428(6)	8873(3)	3000.4(11)	50.6(10)
C7	4965(6)	9705(4)	2818.7(11)	52.4(10)
C39	2880(6)	5172(4)	4605.8(11)	53.6(10)
C36	2014(5)	5213(3)	5254.7(11)	51.2(10)
C25	7522(6)	5133(3)	3366.3(10)	49.0(9)
C33	1685(5)	8924(3)	5120.9(11)	52.0(10)
C34	1453(5)	7987(3)	4985.2(10)	47.1(9)
C1	4814(5)	10639(3)	3396.0(11)	49.3(9)
C24	8977(5)	2681(3)	3334.7(10)	49.9(10)
C32	2443(5)	9651(3)	4939.4(12)	55.0(11)
C38	3284(6)	4371(4)	4802.7(12)	56.0(11)
C27	4013(6)	8088(3)	3463.9(11)	51.6(10)
C37	2848(6)	4412(3)	5127.8(12)	53.6(10)
C13	9541(6)	5229(4)	2736.1(11)	54.6(11)
C30	2816(6)	8517(4)	4501.8(11)	55.5(11)

Table S2 Fractional Atomic Coordinates (×10^4) and Equivalent Isotropic Displacement Parameters (Å^2×10^3) for 3,7-dibromo-10-(chloroacetyl)phenothiazine 10b. U_{eq} is defined as 1/3 of of the trace of the orthogonalised U_{IJ} tensor.

**Atom**	***x***	***y***	***z***	**U(eq)**
C31	3038(6)	9457(4)	4633.7(13)	58.1(11)
C2	5000(6)	11507(4)	3578.4(12)	56.9(11)
C8	5290(6)	9738(4)	2486.3(12)	60.7(12)
C20	10242(6)	3816(4)	3711.9(11)	56.3(11)
C18	9826(6)	5150(4)	2406.1(11)	59.4(12)
C23	9525(6)	1873(4)	3517.0(12)	59.1(11)
C22	10389(6)	2059(4)	3798.8(12)	62.3(13)
C5	5933(6)	9674(4)	3830.6(12)	58.7(11)
C11	6322(6)	8111(4)	2855.4(12)	59.7(11)
C26	7016(7)	4980(4)	3716.7(11)	62.0(12)
C42	33(6)	7663(4)	4100.7(12)	58.2(11)
C28	3580(6)	7217(4)	3239.0(12)	55.6(10)
C9	6087(6)	8939(4)	2347.7(12)	63.4(13)
C10	6629(6)	8135(4)	2529.0(13)	64.2(12)
C21	10790(6)	3016(5)	3895.8(11)	64.6(13)
C3	5614(6)	11429(4)	3887.2(12)	59.2(12)
C4	6108(6)	10524(4)	4012.8(12)	62.5(12)

Table S3 Anisotropic Displacement Parameters ($Å^2×10^3$) for 3,7-dibromo-10-(chloroacetyl)phenothiazine 10b. The Anisotropic displacement factor exponent takes the form: -$2\pi^2[h^2a^{*2}U_{11}+2hka^*b^*U_{12}+\ldots]$.

Atom	U_{11}	U_{22}	U_{33}	U_{23}	U_{13}	U_{12}
Br6	89.1(4)	98.2(4)	40.3(2)	-5.7(2)	5.0(2)	-3.0(3)
Br3	80.0(4)	58.0(3)	78.1(4)	13.8(3)	-10.2(3)	-6.6(3)
Br2	71.2(3)	50.8(3)	110.7(5)	-12.3(3)	-23.0(3)	-0.9(3)
Br7	91.8(4)	116.8(5)	49.8(3)	-6.1(3)	14.0(3)	-31.1(4)
Br4	86.7(4)	104.3(5)	83.2(4)	47.3(4)	9.2(3)	32.5(4)
Br1	127.2(6)	72.8(4)	76.0(4)	-20.7(3)	3.8(4)	-26.4(4)
S2	63.2(6)	49.3(6)	54.8(6)	1.2(5)	-4.6(5)	-6.4(5)
S3	59.1(6)	55.2(6)	49.3(5)	-5.5(5)	11.6(5)	-7.2(5)
S1	71.7(7)	58.0(7)	52.6(6)	4.5(5)	-11.6(5)	4.9(6)
Cl1	64.9(7)	81.4(8)	64.6(7)	10.3(6)	-13.9(6)	0.3(6)
Cl3	65.2(7)	63.3(7)	86.8(9)	-13.2(6)	4.5(6)	-12.8(6)
Cl2	76.8(8)	111.2(11)	49.0(6)	-1.7(7)	10.8(6)	23.8(8)
O00D	71(2)	57.7(19)	51.9(17)	-6.5(15)	-10.0(15)	-4.8(16)
O5	77(2)	60(2)	54.1(18)	10.2(16)	6.6(16)	19.0(17)
O00F	84(2)	74(2)	48.8(18)	-4.3(16)	7.6(17)	-19(2)
N2	61(2)	48.8(19)	33.8(16)	1.2(14)	-1.4(15)	5.0(17)
N1	59(2)	48(2)	42.7(18)	1.4(15)	-1.6(15)	-0.1(16)
N3	59(2)	50(2)	38.2(16)	-0.6(15)	-1.5(15)	-3.5(17)
C14	49(2)	55(2)	34.8(19)	-0.4(17)	-4.3(16)	1.1(19)
C15	45(2)	52(2)	43(2)	1.3(17)	-4.9(16)	2.5(18)
C40	51(2)	50(2)	42(2)	-1.6(17)	0.6(17)	-7.8(19)
C19	52(2)	52(2)	37.8(19)	5.9(17)	0.3(17)	6.5(19)
C41	53(2)	59(3)	40(2)	-3.3(19)	2.9(17)	-3(2)
C35	47(2)	50(2)	42(2)	-3.9(17)	0.5(16)	-8.4(18)
C17	53(2)	71(3)	36(2)	-0.4(19)	-1.0(17)	4(2)
C16	55(2)	58(3)	44(2)	-7.6(19)	-6.5(18)	3(2)
C29	52(2)	46(2)	47(2)	0.1(18)	-3.4(18)	-4.3(18)
C6	49(2)	51(2)	48(2)	-2.9(18)	-3.8(18)	-0.5(19)
C12	54(2)	52(2)	45(2)	1.5(18)	-2.2(18)	-6(2)
C7	53(2)	59(3)	45(2)	-0.2(19)	-3.2(18)	-8(2)
C39	59(2)	57(3)	46(2)	-2.6(19)	7.4(19)	-6(2)
C36	54(2)	54(3)	45(2)	-0.9(19)	1.0(18)	-14(2)
C25	57(2)	49(2)	40(2)	1.7(18)	-0.3(18)	-1(2)
C33	49(2)	53(3)	54(2)	-10(2)	-7.9(19)	-0.9(19)
C34	41.5(19)	53(2)	47(2)	-4.2(18)	-2.7(16)	-2.0(18)
C1	48(2)	52(2)	48(2)	3.1(18)	1.6(17)	-5.4(19)
C24	50(2)	55(2)	45(2)	6.2(18)	2.2(17)	8.6(19)
C32	47(2)	49(2)	69(3)	-4(2)	-18(2)	0.8(19)
C38	55(2)	50(2)	63(3)	-8(2)	4(2)	-2(2)
C27	58(2)	49(2)	48(2)	0.3(19)	-4.4(19)	0(2)
C37	55(2)	46(2)	60(3)	2(2)	-4(2)	-11(2)
C13	70(3)	52(2)	42(2)	0.0(18)	-4(2)	-10(2)
C30	59(3)	61(3)	46(2)	5(2)	-0.2(19)	-4(2)

Table S3 Anisotropic Displacement Parameters (Å^2×10^3) for 3,7-dibromo-10-(chloroacetyl)phenothiazine 10b. The Anisotropic displacement factor exponent takes the form: -2π^2[h^{2}a*^{2}U$_{11}$+2hka*b*U$_{12}$+…].

Atom	U_{11}	U_{22}	U_{33}	U_{23}	U_{13}	U_{12}
C31	53(2)	52(3)	69(3)	10(2)	-11(2)	-4(2)
C2	59(3)	51(3)	60(3)	4(2)	4(2)	-5(2)
C8	62(3)	73(3)	48(2)	7(2)	-3(2)	-13(2)
C20	63(3)	67(3)	39(2)	2(2)	-3.4(19)	3(2)
C18	69(3)	65(3)	44(2)	8(2)	-1(2)	-11(2)
C23	66(3)	53(3)	58(3)	9(2)	10(2)	8(2)
C22	56(3)	76(3)	54(3)	23(2)	9(2)	19(2)
C5	60(3)	65(3)	51(2)	0(2)	-9(2)	0(2)
C11	58(3)	60(3)	60(3)	-1(2)	2(2)	1(2)
C26	70(3)	72(3)	44(2)	4(2)	6(2)	13(3)
C42	63(3)	60(3)	52(2)	5(2)	-7(2)	-6(2)
C28	59(3)	52(2)	55(2)	-3(2)	-1(2)	1(2)
C9	60(3)	85(4)	46(2)	-5(2)	5(2)	-19(3)
C10	59(3)	74(3)	60(3)	-13(2)	9(2)	-3(2)
C21	61(3)	91(4)	43(2)	11(2)	-3(2)	14(3)
C3	60(3)	62(3)	55(3)	-11(2)	7(2)	-16(2)
C4	64(3)	73(3)	51(2)	-4(2)	-9(2)	-11(2)

Table S4 Bond Lengths for 3,7-dibromo-10-(chloroacetyl)phenothiazine 10b.

Atom	Atom	Length/Å	Atom	Atom	Length/Å
Br6	C17	1.896(4)	C19	C20	1.391(6)
Br3	C37	1.889(5)	C41	C42	1.536(7)
Br2	C32	1.896(5)	C35	C36	1.391(6)
Br7	C9	1.895(5)	C17	C16	1.380(7)
Br4	C22	1.898(5)	C17	C18	1.369(7)
Br1	C3	1.900(5)	C29	C34	1.391(6)
S2	C15	1.764(5)	C29	C30	1.380(6)
S2	C24	1.766(4)	C6	C1	1.383(6)
S3	C35	1.764(4)	C6	C5	1.383(6)
S3	C34	1.765(5)	C12	C7	1.393(7)
S1	C7	1.764(5)	C12	C11	1.387(7)
S1	C1	1.768(4)	C7	C8	1.398(7)
Cl1	C42	1.778(5)	C39	C38	1.383(7)
Cl3	C28	1.769(5)	C36	C37	1.372(7)
Cl2	C26	1.773(5)	C25	C26	1.517(6)
O00D	C41	1.205(6)	C33	C34	1.385(6)
O5	C25	1.211(5)	C33	C32	1.373(7)
O00F	C27	1.208(6)	C1	C2	1.391(7)
N2	C14	1.437(5)	C24	C23	1.389(6)
N2	C19	1.430(5)	C32	C31	1.376(7)
N2	C25	1.371(6)	C38	C37	1.389(7)
N1	C6	1.434(6)	C27	C28	1.530(6)
N1	C12	1.424(6)	C13	C18	1.386(6)
N1	C27	1.383(6)	C30	C31	1.381(7)
N3	C40	1.431(6)	C2	C3	1.373(7)
N3	C41	1.379(6)	C8	C9	1.375(8)
N3	C29	1.429(6)	C20	C21	1.385(7)
C14	C15	1.387(6)	C23	C22	1.381(7)
C14	C13	1.382(6)	C22	C21	1.379(8)
C15	C16	1.393(6)	C5	C4	1.370(7)
C40	C35	1.386(6)	C11	C10	1.371(7)
C40	C39	1.384(7)	C9	C10	1.381(8)
C19	C24	1.385(7)	C3	C4	1.376(8)

Table S5 Bond Angles for 3,7-dibromo-10-(chloroacetyl)phenothiazine 10b.

Atom	Atom	Atom	Angle/°	Atom	Atom	Atom	Angle/°
C15	S2	C24	97.7(2)	O5	C25	N2	122.0(4)
C35	S3	C34	97.8(2)	O5	C25	C26	122.7(4)
C7	S1	C1	99.3(2)	N2	C25	C26	115.4(4)
C19	N2	C14	115.6(3)	C32	C33	C34	118.7(4)
C25	N2	C14	119.5(4)	C29	C34	S3	119.5(3)
C25	N2	C19	123.5(4)	C33	C34	S3	120.0(3)
C12	N1	C6	117.3(4)	C33	C34	C29	120.5(4)
C27	N1	C6	118.3(4)	C6	C1	S1	120.3(3)
C27	N1	C12	123.0(4)	C6	C1	C2	120.4(4)
C41	N3	C40	119.3(4)	C2	C1	S1	119.3(4)
C41	N3	C29	123.4(4)	C19	C24	S2	119.3(3)
C29	N3	C40	116.2(3)	C19	C24	C23	120.2(4)
C15	C14	N2	117.8(4)	C23	C24	S2	120.5(4)
C13	C14	N2	122.0(4)	C33	C32	Br2	119.6(4)
C13	C14	C15	120.2(4)	C33	C32	C31	121.7(4)
C14	C15	S2	119.8(3)	C31	C32	Br2	118.7(4)
C14	C15	C16	119.9(4)	C39	C38	C37	118.4(4)
C16	C15	S2	120.2(4)	O00F	C27	N1	121.7(4)
C35	C40	N3	117.6(4)	O00F	C27	C28	121.8(4)
C39	C40	N3	122.4(4)	N1	C27	C28	116.5(4)
C39	C40	C35	120.0(4)	C36	C37	Br3	119.3(4)
C24	C19	N2	118.5(4)	C36	C37	C38	121.8(4)
C24	C19	C20	120.0(4)	C38	C37	Br3	118.9(4)
C20	C19	N2	121.4(4)	C14	C13	C18	120.1(4)
O00D	C41	N3	122.4(4)	C29	C30	C31	120.4(5)
O00D	C41	C42	122.4(4)	C32	C31	C30	119.1(5)
N3	C41	C42	115.2(4)	C3	C2	C1	118.6(5)
C40	C35	S3	120.4(3)	C9	C8	C7	118.3(5)
C40	C35	C36	119.9(4)	C21	C20	C19	120.1(5)
C36	C35	S3	119.6(3)	C17	C18	C13	119.2(5)
C16	C17	Br6	118.6(4)	C22	C23	C24	118.7(5)
C18	C17	Br6	119.4(4)	C23	C22	Br4	118.3(4)
C18	C17	C16	122.0(4)	C21	C22	Br4	119.6(4)
C17	C16	C15	118.6(4)	C21	C22	C23	122.1(4)
C34	C29	N3	118.5(4)	C4	C5	C6	120.5(5)
C30	C29	N3	122.3(4)	C10	C11	C12	120.2(5)
C30	C29	C34	119.3(4)	C25	C26	Cl2	111.6(3)
C1	C6	N1	118.9(4)	C41	C42	Cl1	111.2(3)
C5	C6	N1	121.6(4)	C27	C28	Cl3	110.5(3)
C5	C6	C1	119.5(4)	C8	C9	Br7	118.6(4)
C7	C12	N1	118.6(4)	C8	C9	C10	122.0(5)
C11	C12	N1	121.7(4)	C10	C9	Br7	119.4(4)
C11	C12	C7	119.7(4)	C11	C10	C9	119.5(5)
C12	C7	S1	120.4(4)	C22	C21	C20	118.7(5)

Table S5 Bond Angles for 3,7-dibromo-10-(chloroacetyl)phenothiazine 10b.

Atom	Atom	Atom	Angle/°	Atom	Atom	Atom	Angle/°
C12	C7	C8	120.2(5)	C2	C3	Br1	119.7(4)
C8	C7	S1	119.5(4)	C2	C3	C4	121.5(5)
C38	C39	C40	120.7(4)	C4	C3	Br1	118.7(4)
C37	C36	C35	119.2(4)	C5	C4	C3	119.5(5)

Table S6 Torsion Angles for 3,7-dibromo-10-(chloroacetyl)phenothiazine 10b.

A	B	C	D	Angle/°	A	B	C	D	Angle/°
Br6	C17	C16	C15	-179.6(3)	C29	N3	C40	C35	45.5(5)
Br6	C17	C18	C13	-178.7(4)	C29	N3	C40	C39	-132.5(4)
Br2	C32	C31	C30	-178.9(3)	C29	N3	C41	O00D	-176.5(4)
Br7	C9	C10	C11	179.6(4)	C29	N3	C41	C42	2.7(6)
Br4	C22	C21	C20	-178.2(4)	C29	C30	C31	C32	2.3(7)
Br1	C3	C4	C5	177.7(4)	C6	N1	C12	C7	-46.1(6)
S2	C15	C16	C17	176.2(3)	C6	N1	C12	C11	133.0(5)
S2	C24	C23	C22	179.3(4)	C6	N1	C27	O00F	-8.2(7)
S3	C35	C36	C37	178.5(3)	C6	N1	C27	C28	172.9(4)
S1	C7	C8	C9	-179.8(4)	C6	C1	C2	C3	-0.8(7)
S1	C1	C2	C3	177.2(4)	C6	C5	C4	C3	-0.2(8)
O00D	C41	C42	Cl1	15.4(6)	C12	N1	C6	C1	45.0(6)
O5	C25	C26	Cl2	22.2(7)	C12	N1	C6	C5	-136.3(5)
O00F	C27	C28	Cl3	23.4(6)	C12	N1	C27	O00F	-174.5(5)
N2	C14	C15	S2	3.5(5)	C12	N1	C27	C28	6.7(6)
N2	C14	C15	C16	-178.3(4)	C12	C7	C8	C9	-0.8(7)
N2	C14	C13	C18	180.0(4)	C12	C11	C10	C9	2.1(8)
N2	C19	C24	S2	3.0(6)	C7	S1	C1	C6	-32.2(4)
N2	C19	C24	C23	-177.4(4)	C7	S1	C1	C2	149.7(4)
N2	C19	C20	C21	177.3(4)	C7	C12	C11	C10	-5.6(7)
N2	C25	C26	Cl2	-156.2(4)	C7	C8	C9	Br7	179.7(4)
N1	C6	C1	S1	-0.9(6)	C7	C8	C9	C10	-2.8(7)
N1	C6	C1	C2	177.2(4)	C39	C40	C35	S3	-178.9(3)
N1	C6	C5	C4	-176.6(5)	C39	C40	C35	C36	-0.8(6)
N1	C12	C7	S1	3.1(6)	C39	C38	C37	Br3	-178.2(3)
N1	C12	C7	C8	-176.0(4)	C39	C38	C37	C36	1.1(7)
N1	C12	C11	C10	175.3(5)	C25	N2	C14	C15	-121.2(5)
N1	C27	C28	Cl3	-157.8(3)	C25	N2	C14	C13	60.2(6)
N3	C40	C35	S3	3.1(5)	C25	N2	C19	C24	116.8(5)
N3	C40	C35	C36	-178.9(4)	C25	N2	C19	C20	-64.4(6)
N3	C40	C39	C38	179.3(4)	C33	C32	C31	C30	2.3(7)
N3	C41	C42	Cl1	-163.9(3)	C34	S3	C35	C40	-38.0(4)
N3	C29	C34	S3	2.9(6)	C34	S3	C35	C36	144.0(3)
N3	C29	C34	C33	-176.4(4)	C34	C29	C30	C31	-6.1(7)
N3	C29	C30	C31	175.8(4)	C34	C33	C32	Br2	178.2(3)
C14	N2	C19	C24	-49.6(6)	C34	C33	C32	C31	-2.9(7)

Table S6 Torsion Angles for 3,7-dibromo-10-(chloroacetyl)phenothiazine 10b.

A	B	C	D	Angle/°	A	B	C	D	Angle/°
C14	N2	C19	C20	129.2(4)	C1	S1	C7	C12	31.1(4)
C14	N2	C25	O5	-10.9(7)	C1	S1	C7	C8	-149.8(4)
C14	N2	C25	C26	167.6(4)	C1	C6	C5	C4	2.1(7)
C14	C15	C16	C17	-2.0(6)	C1	C2	C3	Br1	-177.2(4)
C14	C13	C18	C17	-1.3(8)	C1	C2	C3	C4	2.7(7)
C15	S2	C24	C19	35.5(4)	C24	S2	C15	C14	-38.9(4)
C15	S2	C24	C23	-144.1(4)	C24	S2	C15	C16	142.9(4)
C15	C14	C13	C18	1.4(7)	C24	C19	C20	C21	-4.0(7)
C40	N3	C41	O00D	-8.8(7)	C24	C23	C22	Br4	178.0(4)
C40	N3	C41	C42	170.5(4)	C24	C23	C22	C21	-3.2(7)
C40	N3	C29	C34	-48.9(6)	C32	C33	C34	S3	179.8(3)
C40	N3	C29	C30	129.2(5)	C32	C33	C34	C29	-0.9(6)
C40	C35	C36	C37	0.5(6)	C27	N1	C6	C1	-122.1(5)
C40	C39	C38	C37	-1.4(7)	C27	N1	C6	C5	56.6(6)
C19	N2	C14	C15	45.8(5)	C27	N1	C12	C7	120.3(5)
C19	N2	C14	C13	-132.8(5)	C27	N1	C12	C11	-60.6(6)
C19	N2	C25	O5	-176.8(4)	C13	C14	C15	S2	-177.9(4)
C19	N2	C25	C26	1.7(6)	C13	C14	C15	C16	0.3(6)
C19	C24	C23	C22	-0.3(7)	C30	C29	C34	S3	-175.3(3)
C19	C20	C21	C22	0.6(7)	C30	C29	C34	C33	5.4(7)
C41	N3	C40	C35	-123.2(4)	C2	C3	C4	C5	-2.2(8)
C41	N3	C40	C39	58.9(6)	C8	C9	C10	C11	2.1(8)
C41	N3	C29	C34	119.2(5)	C20	C19	C24	S2	-175.8(4)
C41	N3	C29	C30	-62.7(6)	C20	C19	C24	C23	3.8(7)
C35	S3	C34	C29	34.6(4)	C18	C17	C16	C15	2.1(7)
C35	S3	C34	C33	-146.1(4)	C23	C22	C21	C20	3.0(8)
C35	C40	C39	C38	1.3(7)	C5	C6	C1	S1	-179.6(4)
C35	C36	C37	Br3	178.6(3)	C5	C6	C1	C2	-1.5(7)
C35	C36	C37	C38	-0.6(7)	C11	C12	C7	S1	-176.1(4)
C16	C17	C18	C13	-0.4(8)	C11	C12	C7	C8	4.9(7)

Table S7 Hydrogen Atom Coordinates (Å×10^4) and Isotropic Displacement Parameters (Å^2×10^3) for 3,7-dibromo-10-(chloroacetyl)phenothiazine 10b.

Atom	*x*	*y*	*z*	U(eq)
H16	8876.17	2810.75	2315.99	62
H39	3183.56	5163.54	4388.56	64
H36	1725.2	5223.57	5472.63	61
H33	1333.56	9058.79	5330.85	62
H38	3833.7	3817.88	4719.46	67
H13	9741.7	5830.77	2842.24	65
H30	3248.33	8370.48	4298.76	67
H31	3581.69	9952.49	4517.51	70
H2	4713.51	12127.46	3492.98	68

Table S7 Hydrogen Atom Coordinates (Å×10^{4}) and Isotropic Displacement Parameters (Å^{2}×10^{3}) for 3,7-dibromo-10-(chloroacetyl)phenothiazine 10b.

Atom	*x*	*y*	*z*	U(eq)
H8	4974.91	10286.73	2362.24	73
H20	10509.49	4466.77	3772.54	68
H18	10194.73	5699.67	2288.65	71
H23	9313.44	1220.59	3450.69	71
H5	6269.07	9060.74	3914.31	70
H11	6714.86	7582.8	2980.11	72
H26A	7824.72	5287.55	3857.83	74
H26B	6990.85	4268.87	3764.22	74
H42A	929.63	7976.7	3983.95	70
H42B	-335.67	8125.7	4266.63	70
H28A	4486.36	6745.81	3232.82	67
H28B	3403.06	7466.2	3021.02	67
H10	7196.85	7613.46	2430.43	77
H21	11416.76	3121.7	4081.23	78
H4	6556.78	10489.76	4219.86	75

Figure S7. FTIR spectrum of 4-(4-chlorophenyl)-2-thioxo-1,2,5,6,7,8-hexahydroquinoline-3-carbonitrile 9b

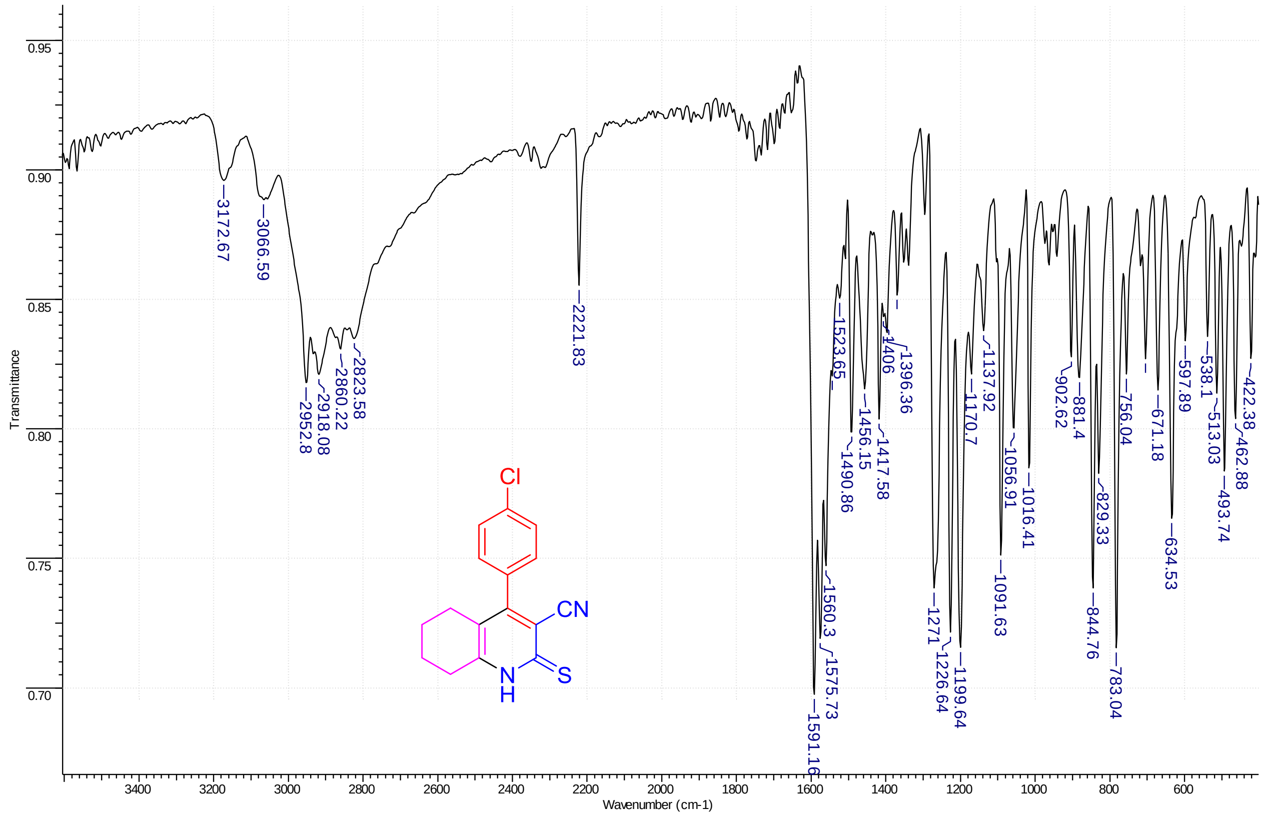

Figure S8. ^{1}H **NMR spectrum (400 MHz, DMSO-d$_6$) of 4-(4-chlorophenyl)-2-thioxo-1,2,5,6,7,8-hexahydroquinoline-3-carbonitrile 9b**

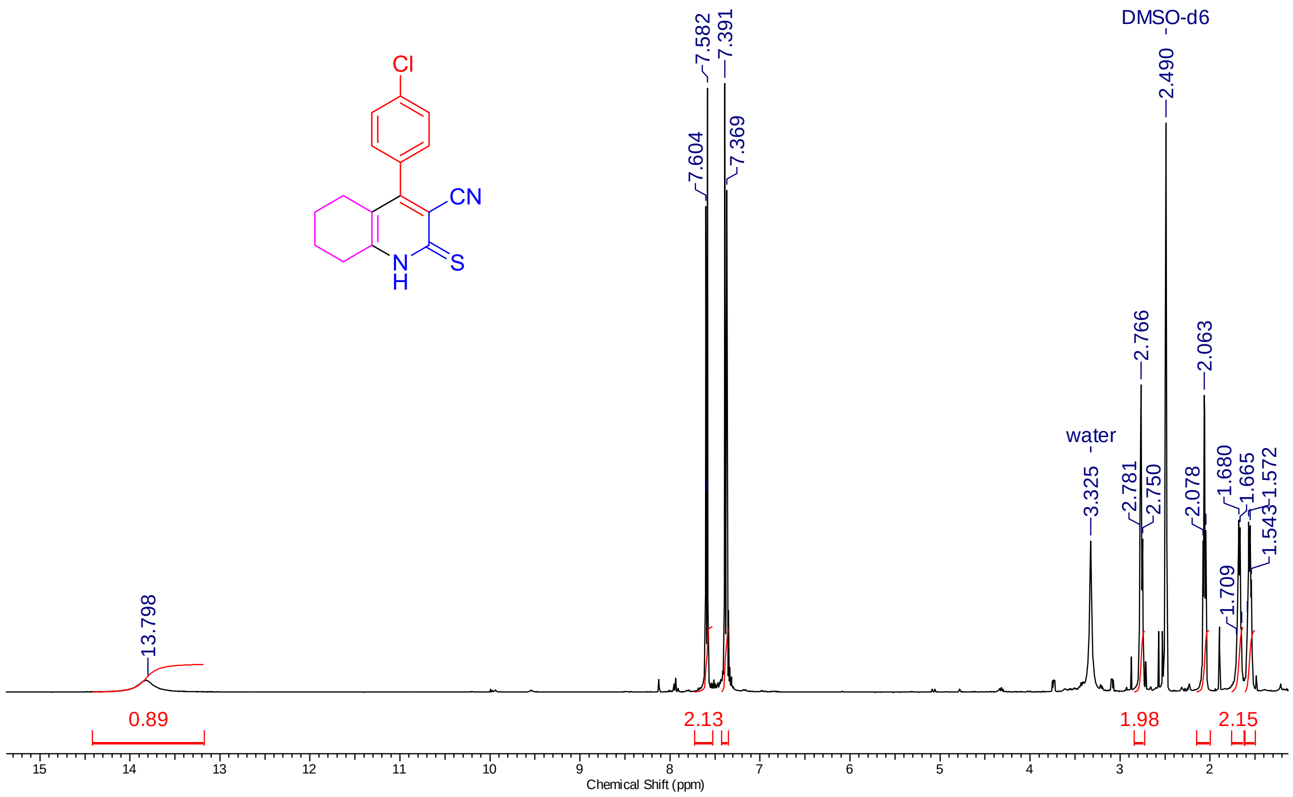

Figure S9. ^{13}C **DEPTQ NMR spectrum (101 MHz, DMSO-d$_6$) of 4-(4-chlorophenyl)-2-thioxo-1,2,5,6,7,8-hexahydroquinoline-3-carbonitrile 9b**

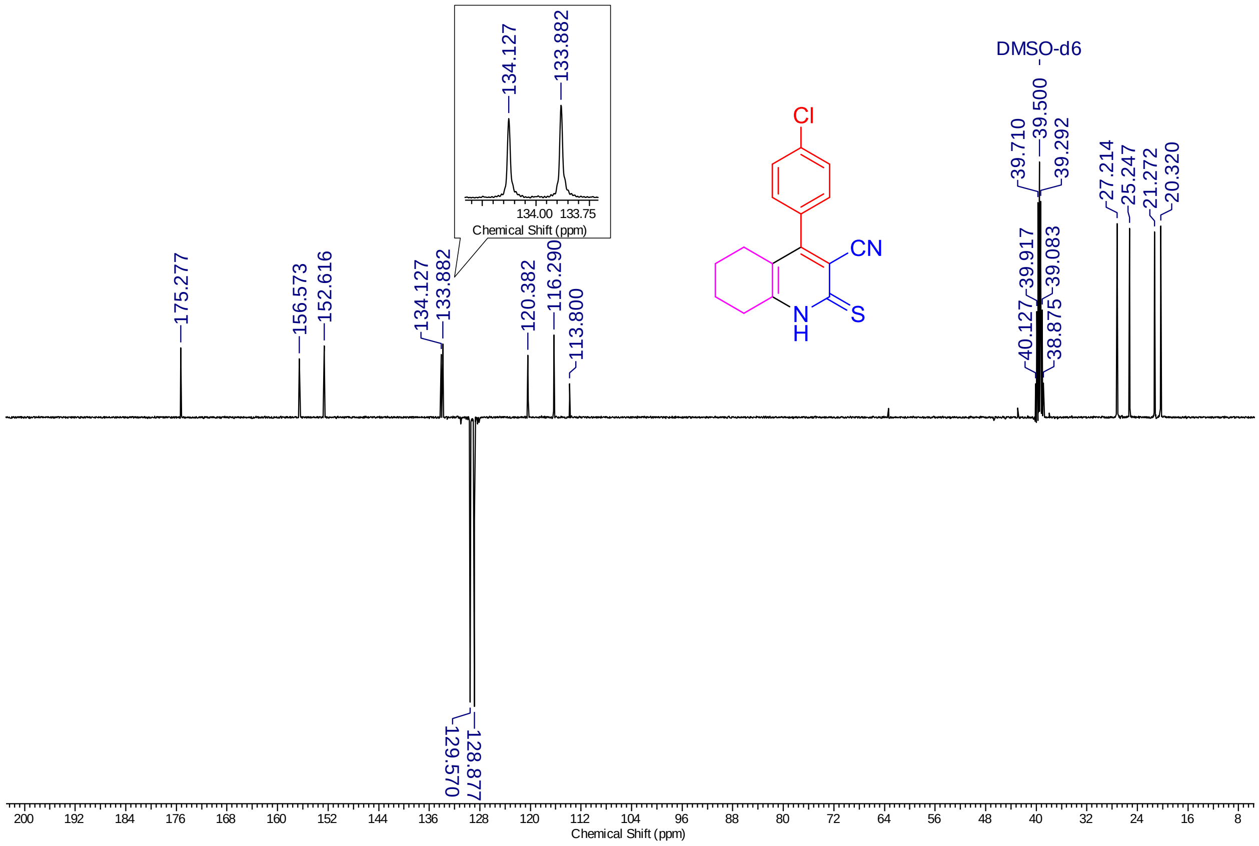

Figure S10. ^{1}H NMR spectrum (400 MHz, DMSO-d$_6$) of 4-(3-bromophenyl)-2-thioxo-1,2,5,6,7,8-hexahydroquinoline-3-carbonitrile 9c

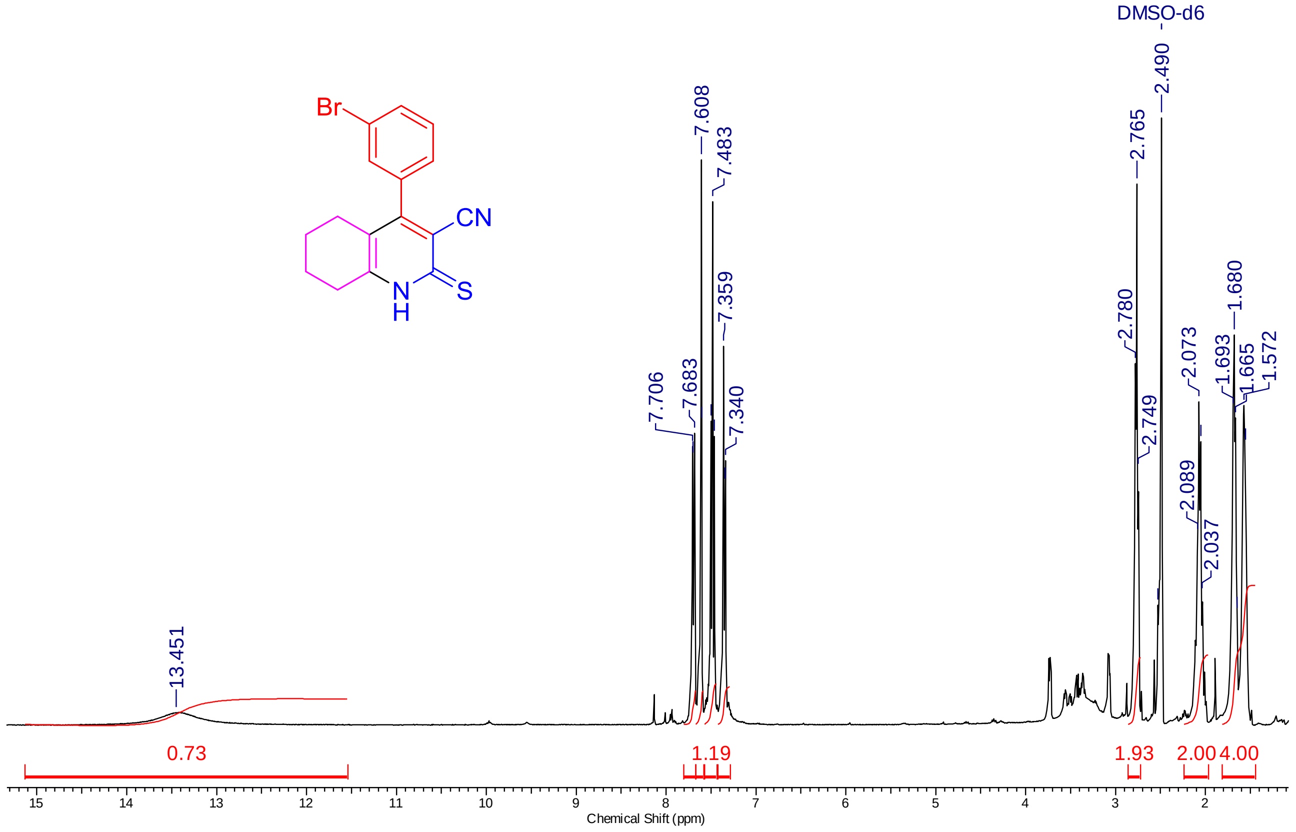

Figure S11. ^{13}C DEPTQ NMR spectrum (101 MHz, DMSO-d$_6$) of 4-(3-bromophenyl)-2-thioxo-1,2,5,6,7,8-hexahydroquinoline-3-carbonitrile 9c

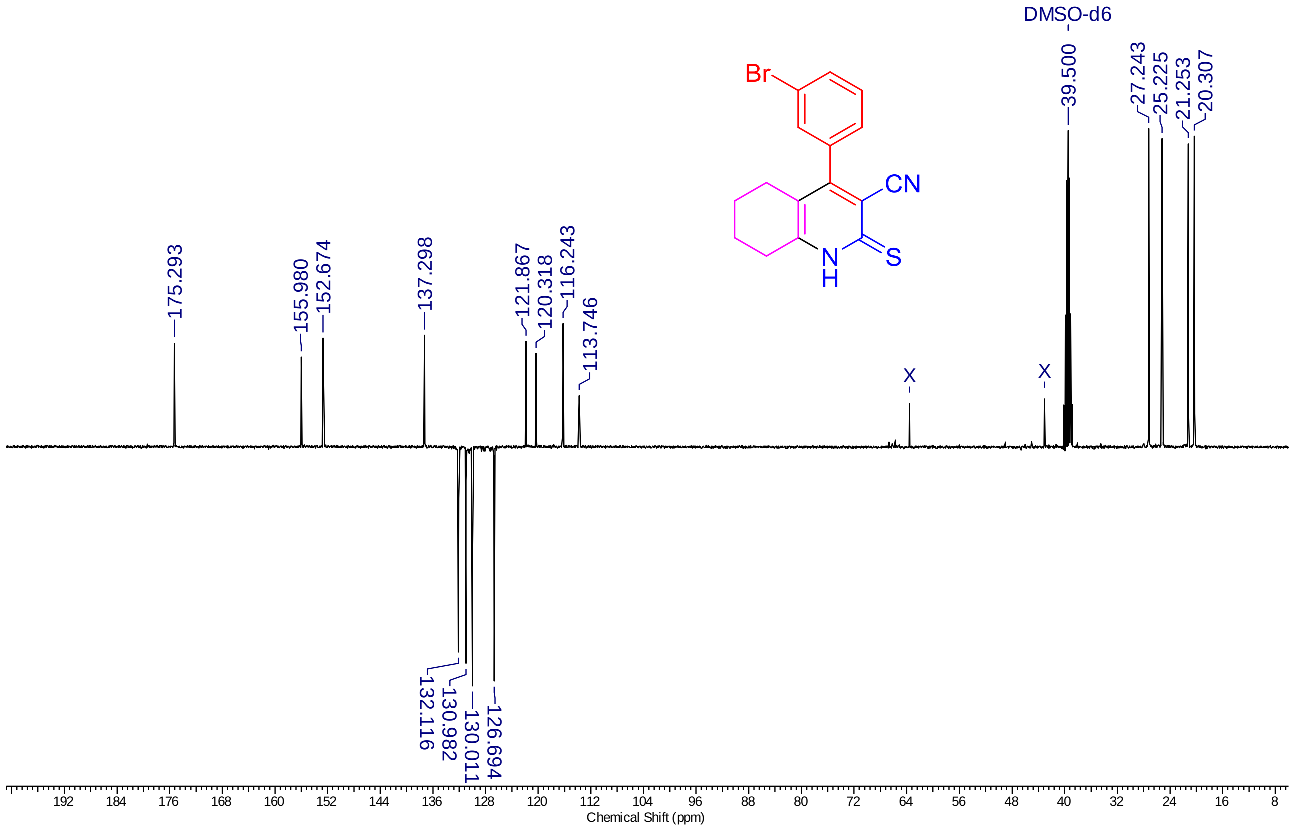

Figure S12. ^{1}H NMR spectrum (400 MHz, DMSO-d_6) of 4-(4-fluorophenyl)-2-thioxo-1,2,5,6,7,8-hexahydroquinoline-3-carbonitrile 9d

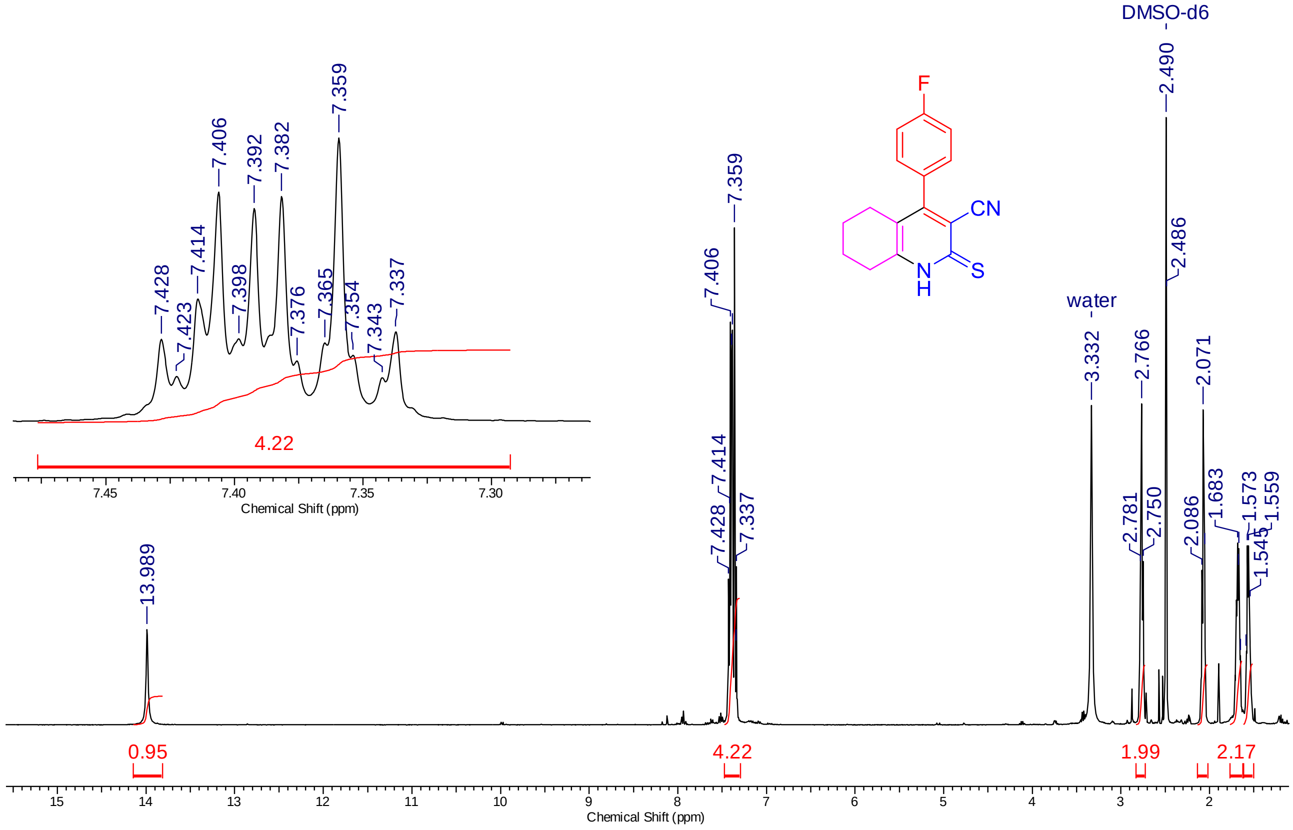

Figure S13. ^{13}C DEPTQ NMR spectrum (101 MHz, DMSO-d_6) of 4-(4-fluorophenyl)-2-thioxo-1,2,5,6,7,8-hexahydroquinoline-3-carbonitrile 9d

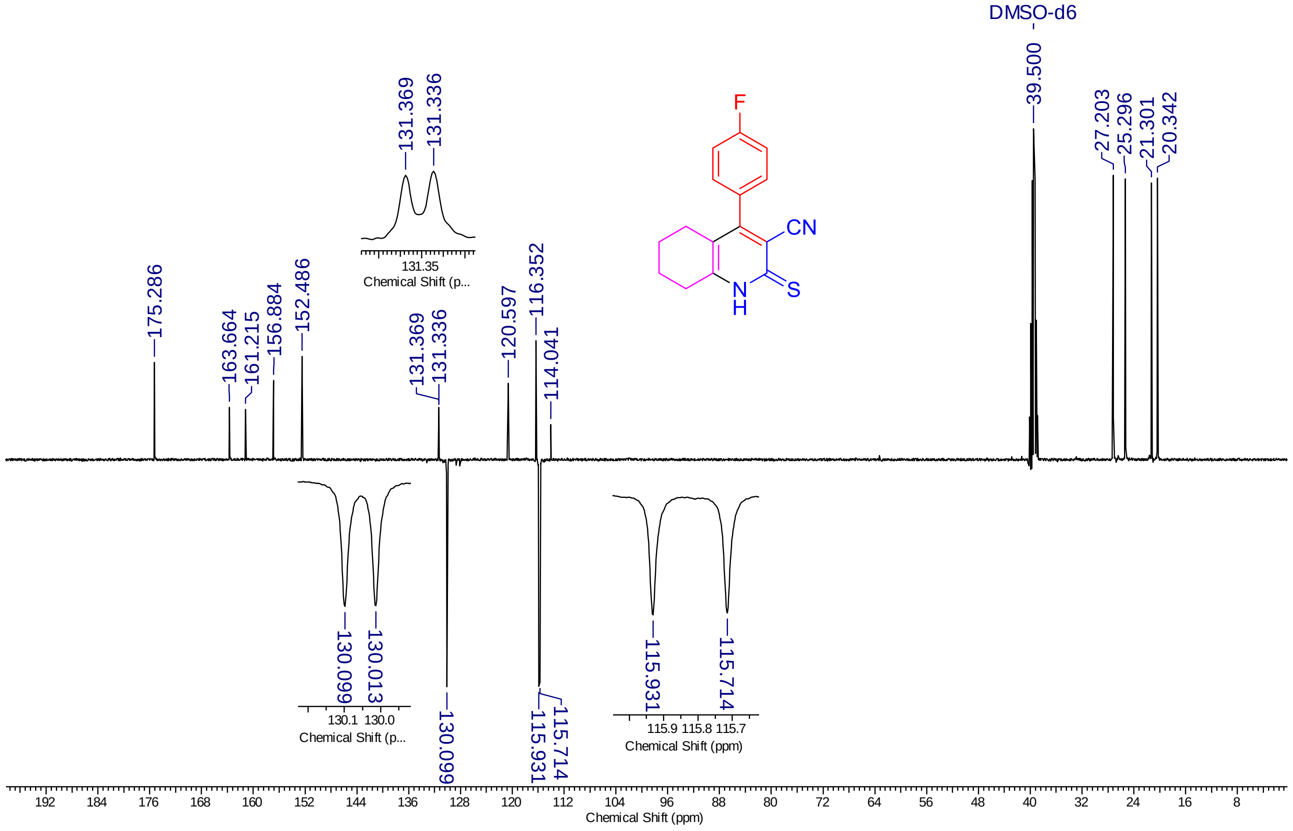

Figure S14. FTIR spectrum of 4-(2-thienyl)-2-thioxo-1,2,5,6,7,8-hexahydroquinoline-3-carbonitrile 9e

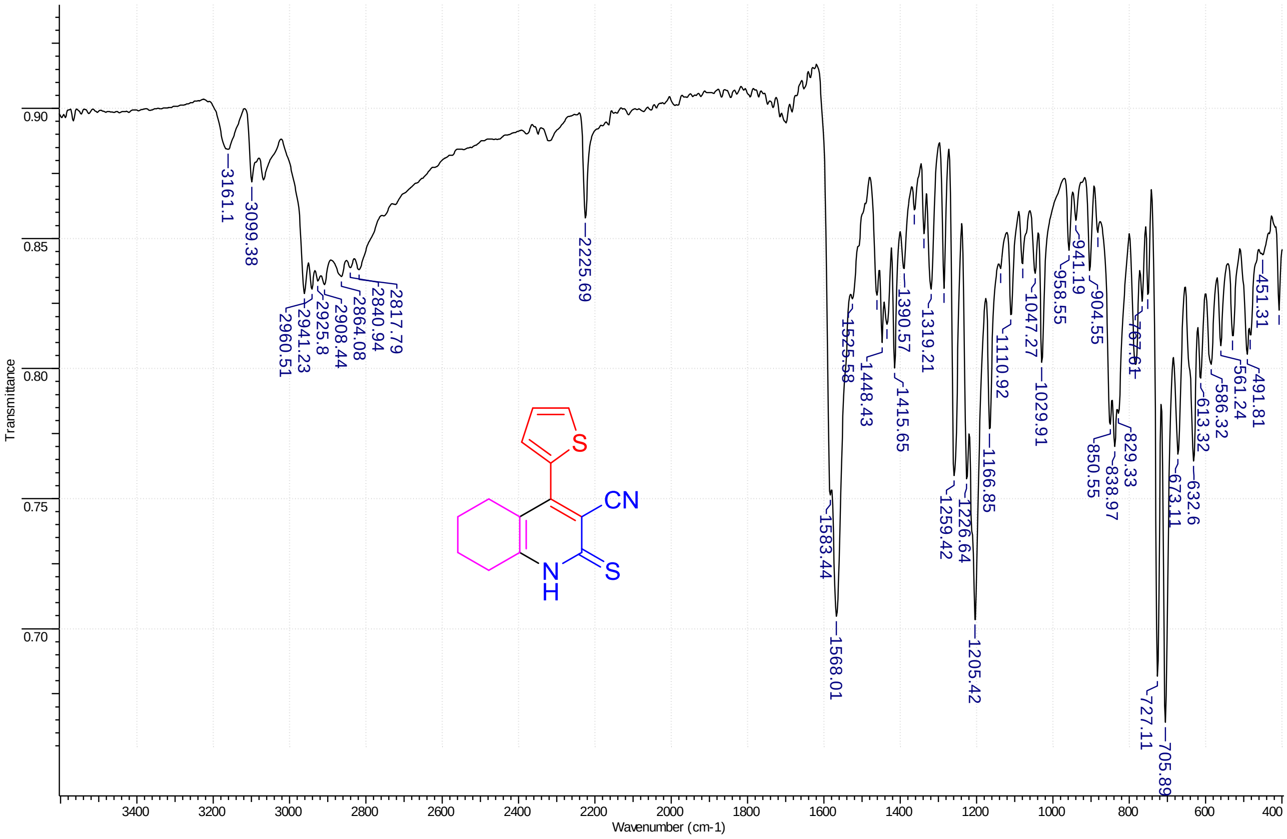

Figure S15. ^{1}H NMR spectrum (400 MHz, DMSO-d$_6$) of 4-(2-thienyl)-2-thioxo-1,2,5,6,7,8-hexahydroquinoline-3-carbonitrile 9e

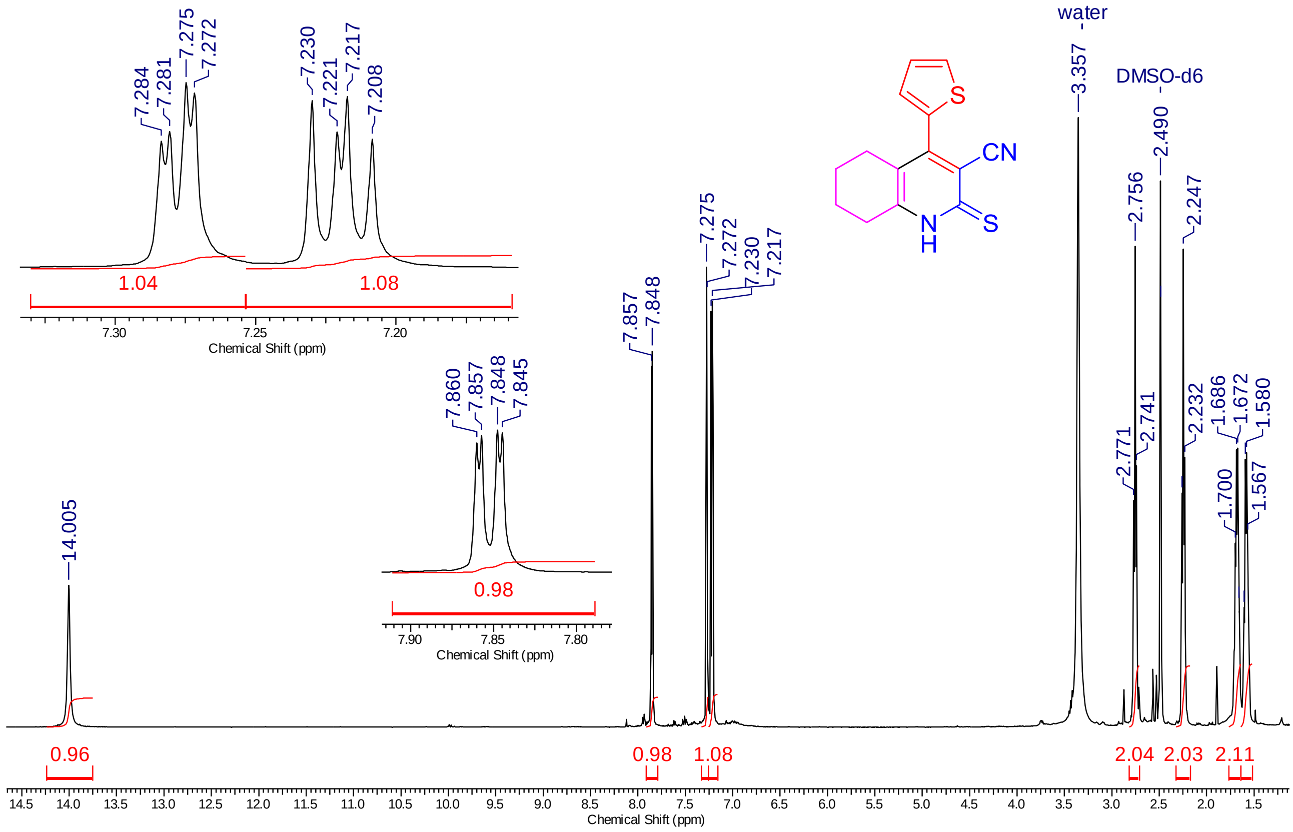

Figure S16. ^{13}C DEPTQ NMR spectrum (101 MHz, DMSO-d_6) of 4-(2-thienyl)-2-thioxo-1,2,5,6,7,8-hexahydroquinoline-3-carbonitrile 9e

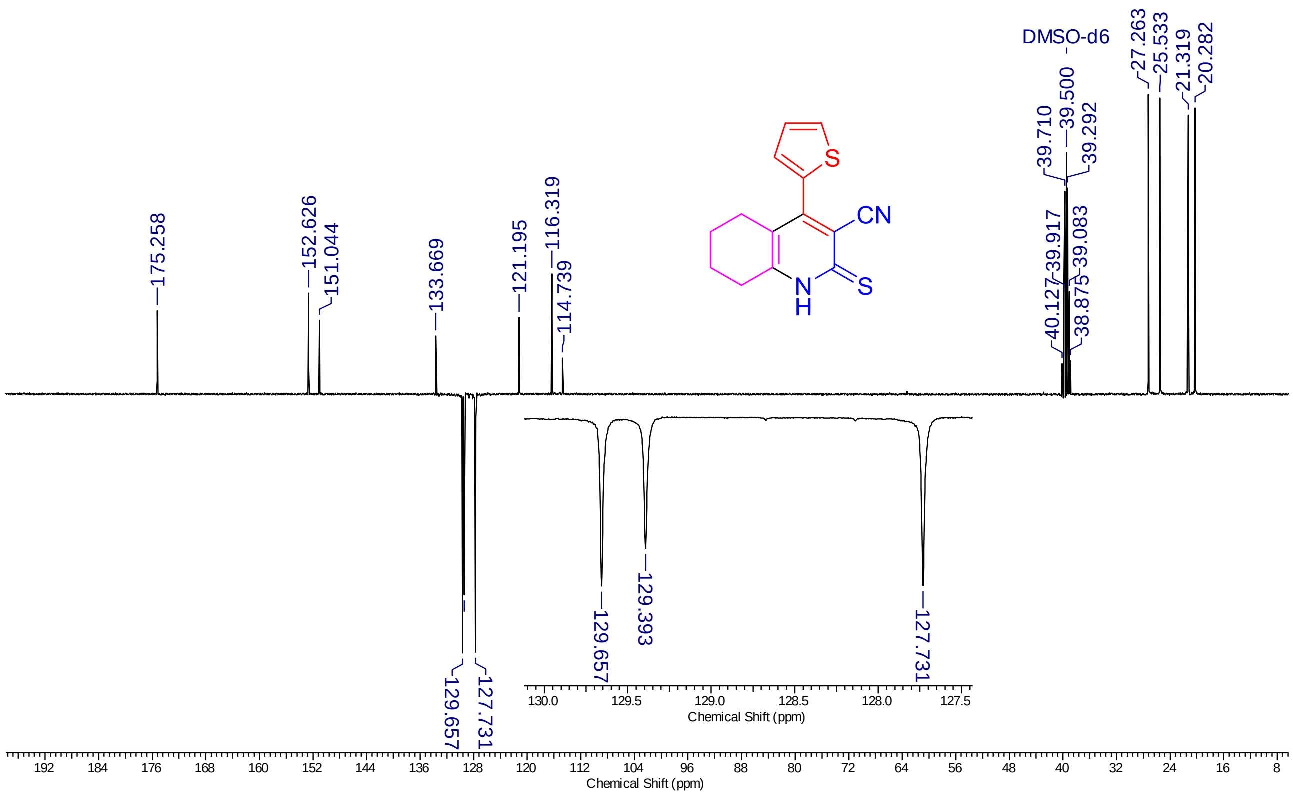

Figure S17. ^{1}H NMR spectrum (400 MHz, DMSO-d_6) of 4-(2,4-dichlorophenyl)-2-thioxo-1,2,5,6,7,8-hexahydroquinoline-3-carbonitrile 9f

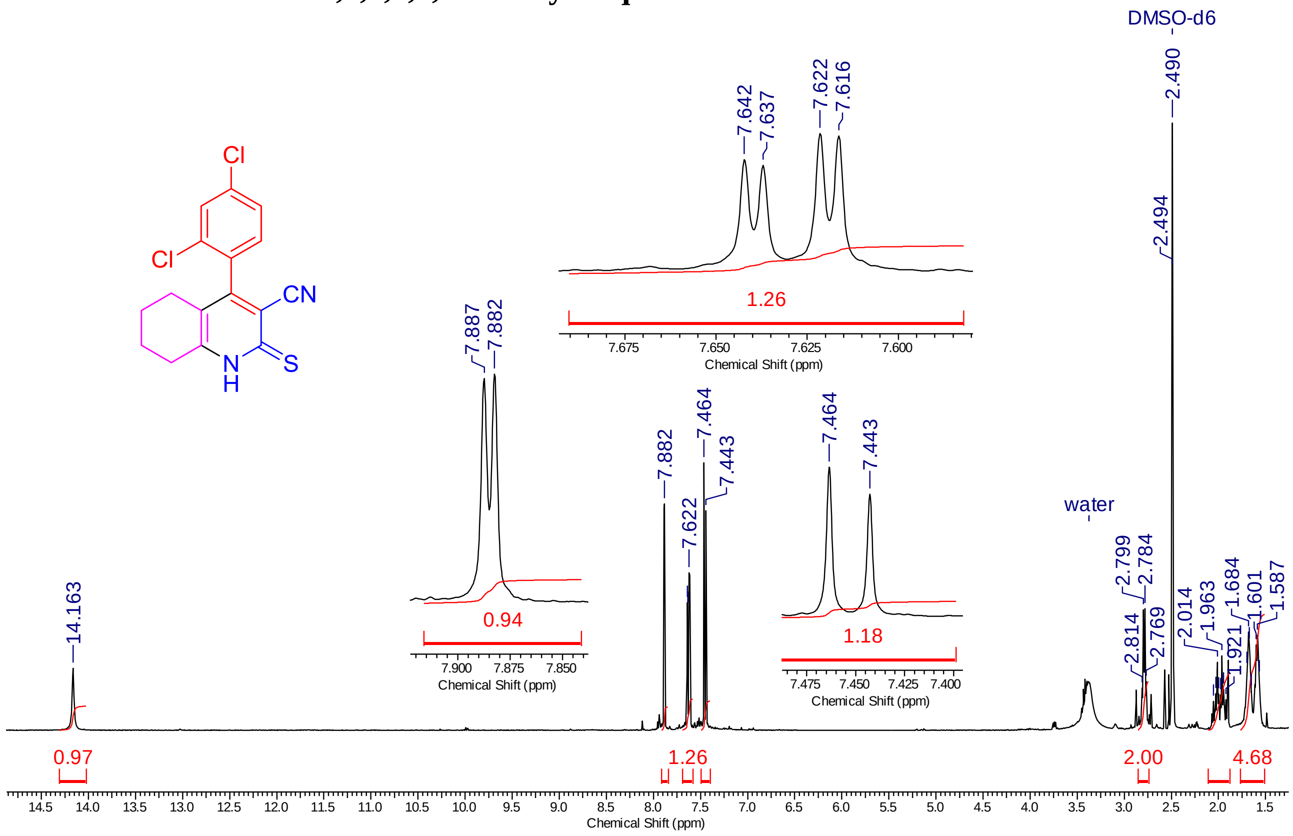

Figure S18. ^{13}C **DEPTQ NMR spectrum (101 MHz, DMSO-d_6) of 4-(2,4-dichlorophenyl)-2-thioxo-1,2,5,6,7,8-hexahydroquinoline-3-carbonitrile 9f**

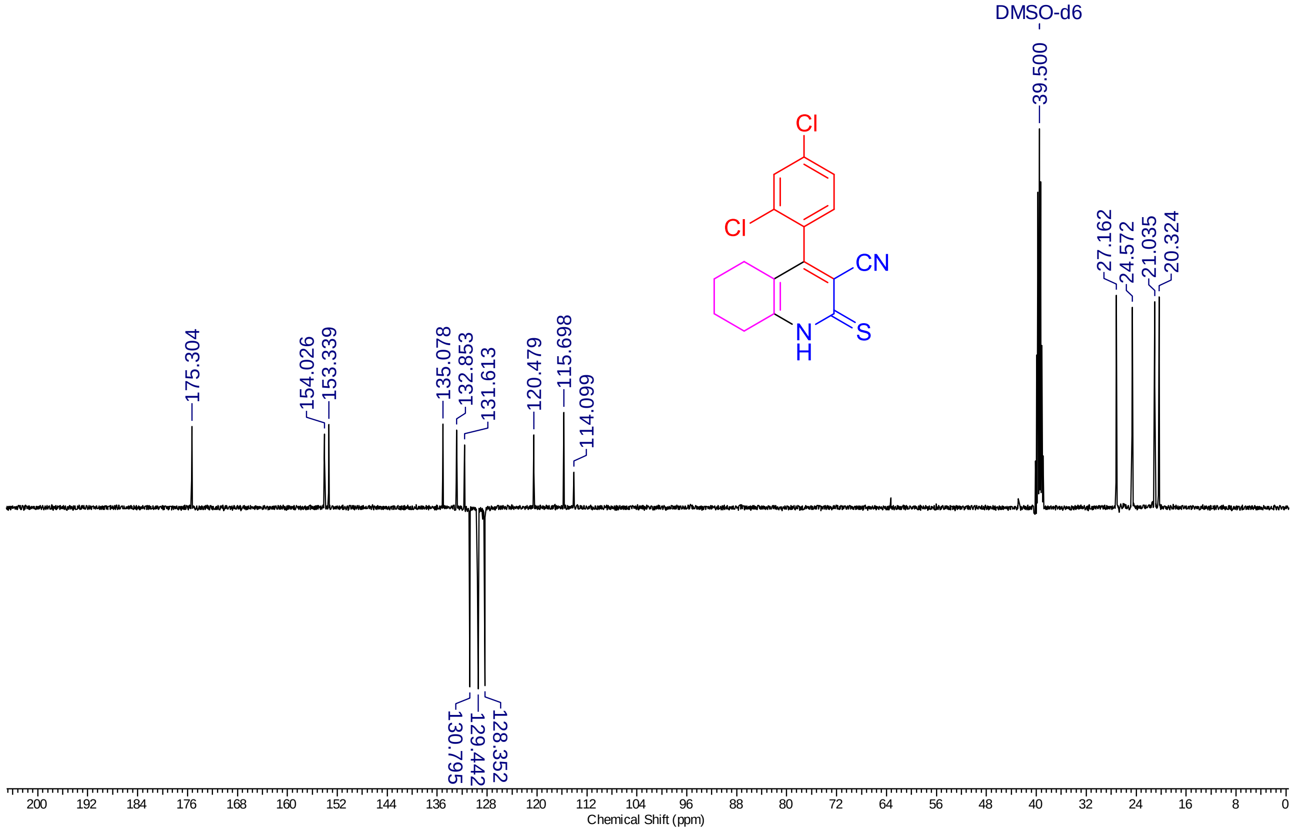

Figure S19. FTIR spectrum of 4-(3-nitrophenyl)-2-thioxo-1,2,5,6,7,8-hexahydroquinoline-3-carbonitrile 9h

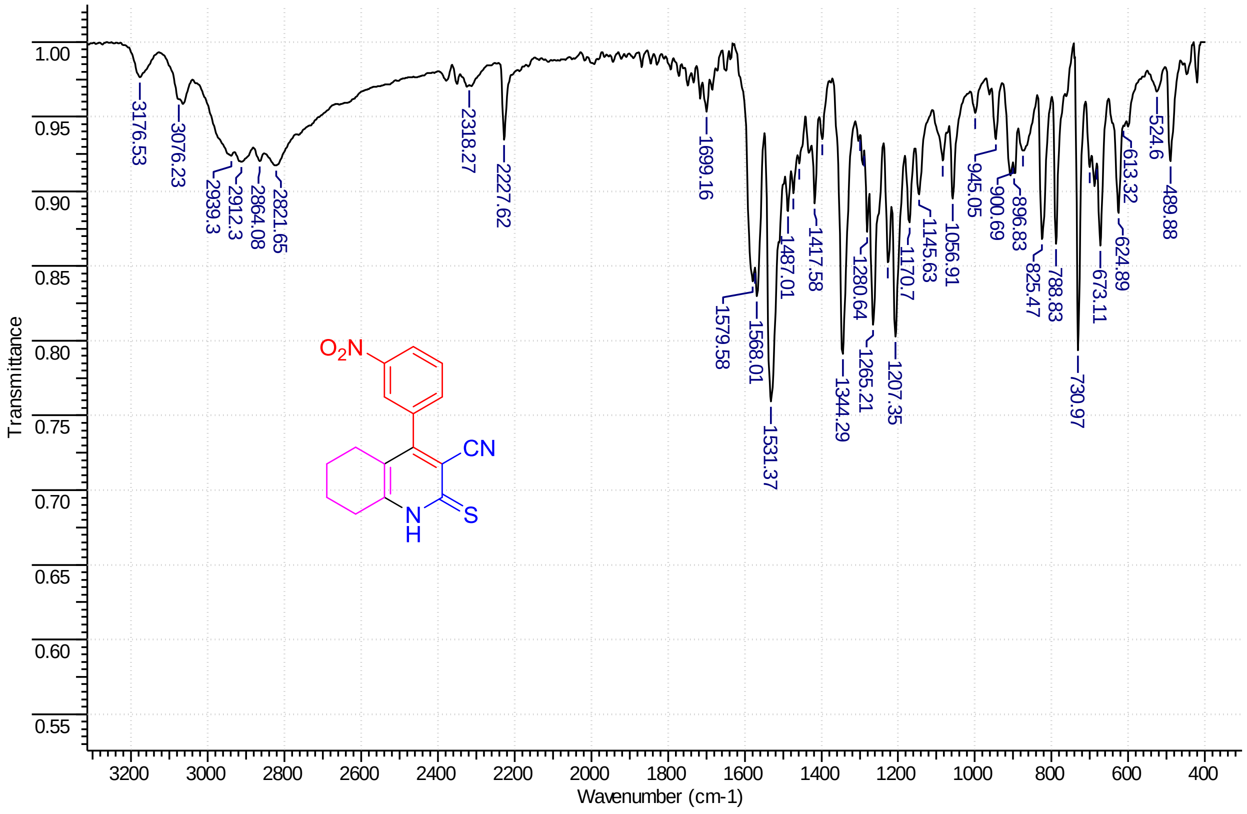

Figure S20. ^{1}H NMR spectrum (400 MHz, DMSO-d_6) of 4-(3-nitrophenyl)-2-thioxo-1,2,5,6,7,8-hexahydroquinoline-3-carbonitrile 9h

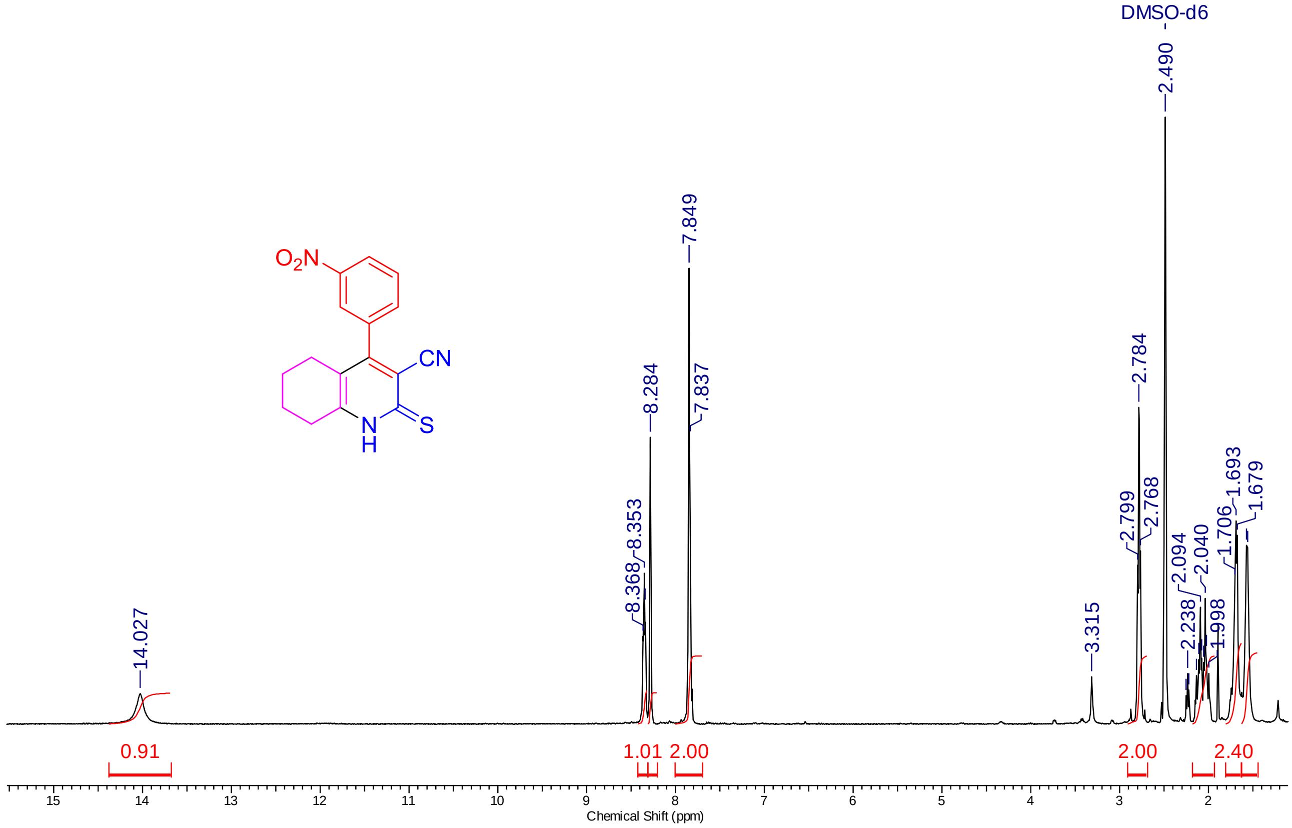

Figure S21. ^{13}C DEPTQ NMR spectrum (101 MHz, DMSO-d_6) of 4-(3-nitrophenyl)-2-thioxo-1,2,5,6,7,8-hexahydroquinoline-3-carbonitrile 9h

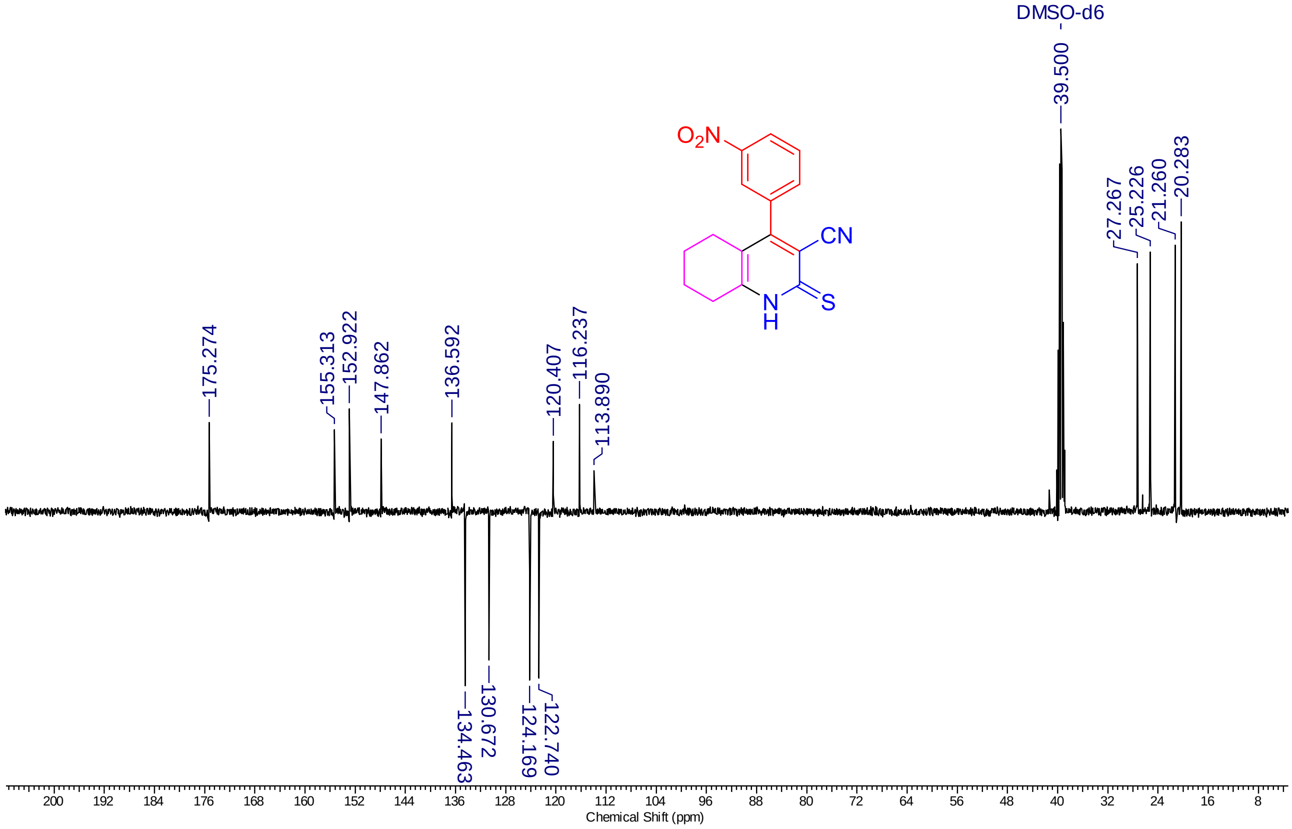

Figure S22. ^{1}H NMR spectrum (400 MHz, DMSO-d_6) of 4-(4-chlorophenyl)-2-{[2-oxo-2-(10H-phenothiazin-10-yl)ethyl]thio}-5,6,7,8-tetrahydroquinoline-3-carbonitrile 11a

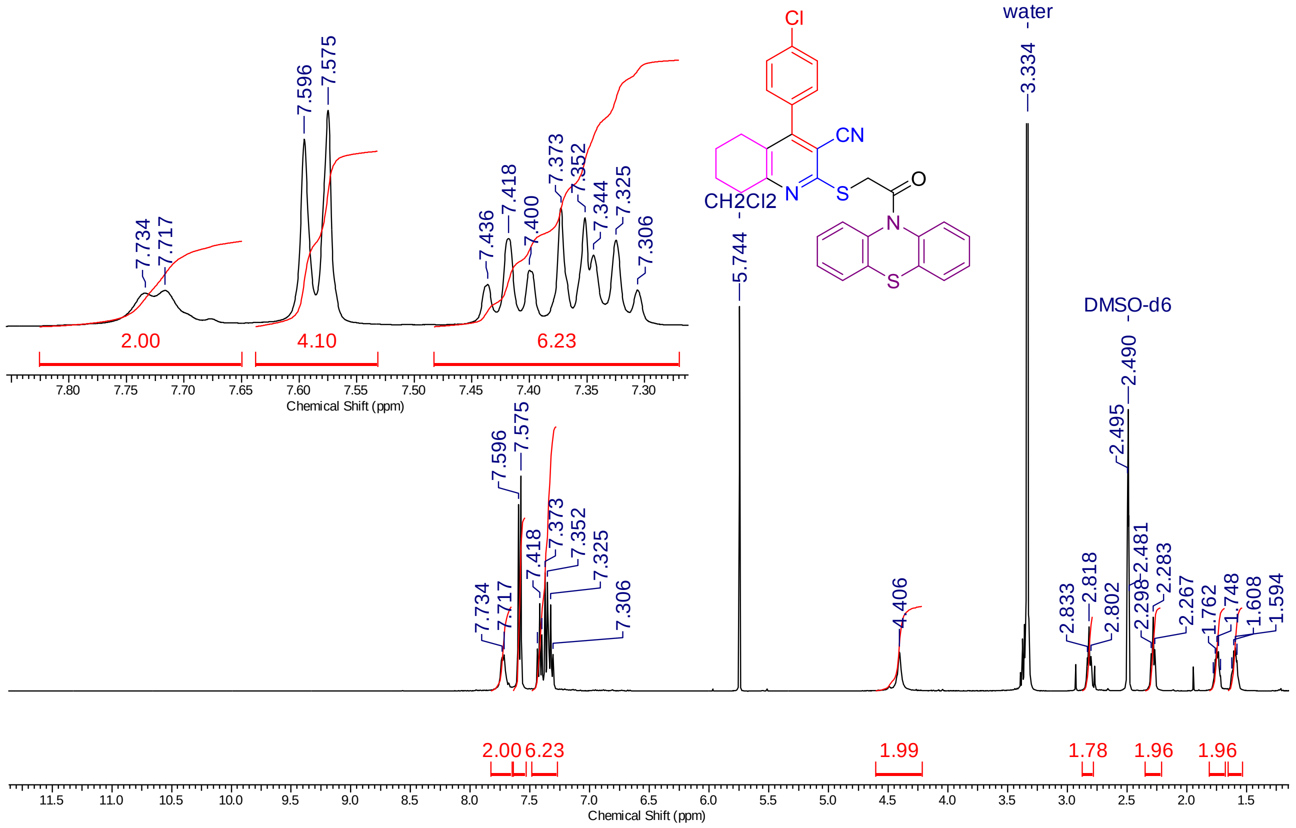

Figure S23. ^{13}C DEPTQ NMR spectrum (101 MHz, DMSO-d_6) of 4-(4-chlorophenyl)-2-{[2-oxo-2-(10H-phenothiazin-10-yl)ethyl]thio}-5,6,7,8-tetrahydroquinoline-3-carbonitrile 11a

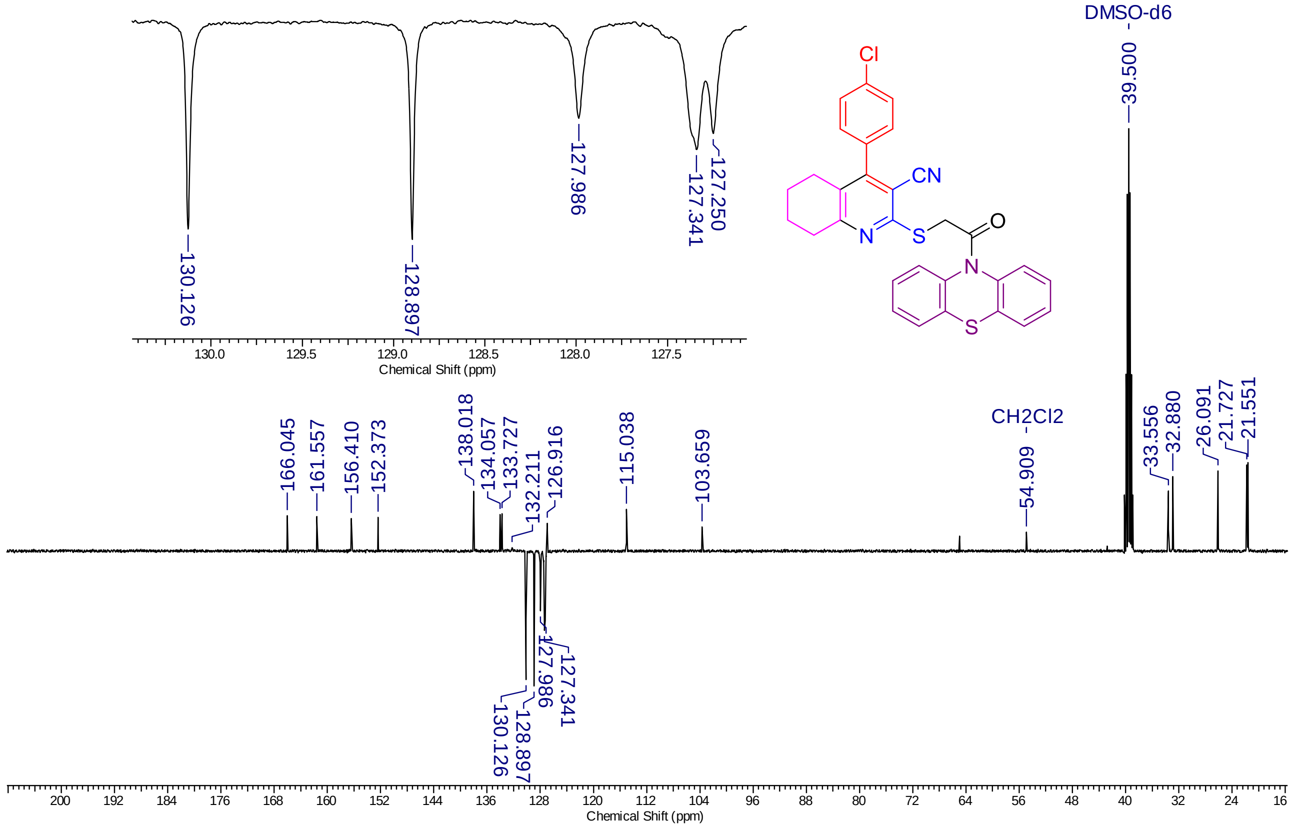

Figure S24. FTIR spectrum of 4-(3-bromophenyl)-2-{[2-oxo-2-(10H-phenothiazin-10-yl)ethyl]thio}-5,6,7,8-tetrahydroquinoline-3-carbonitrile 11b

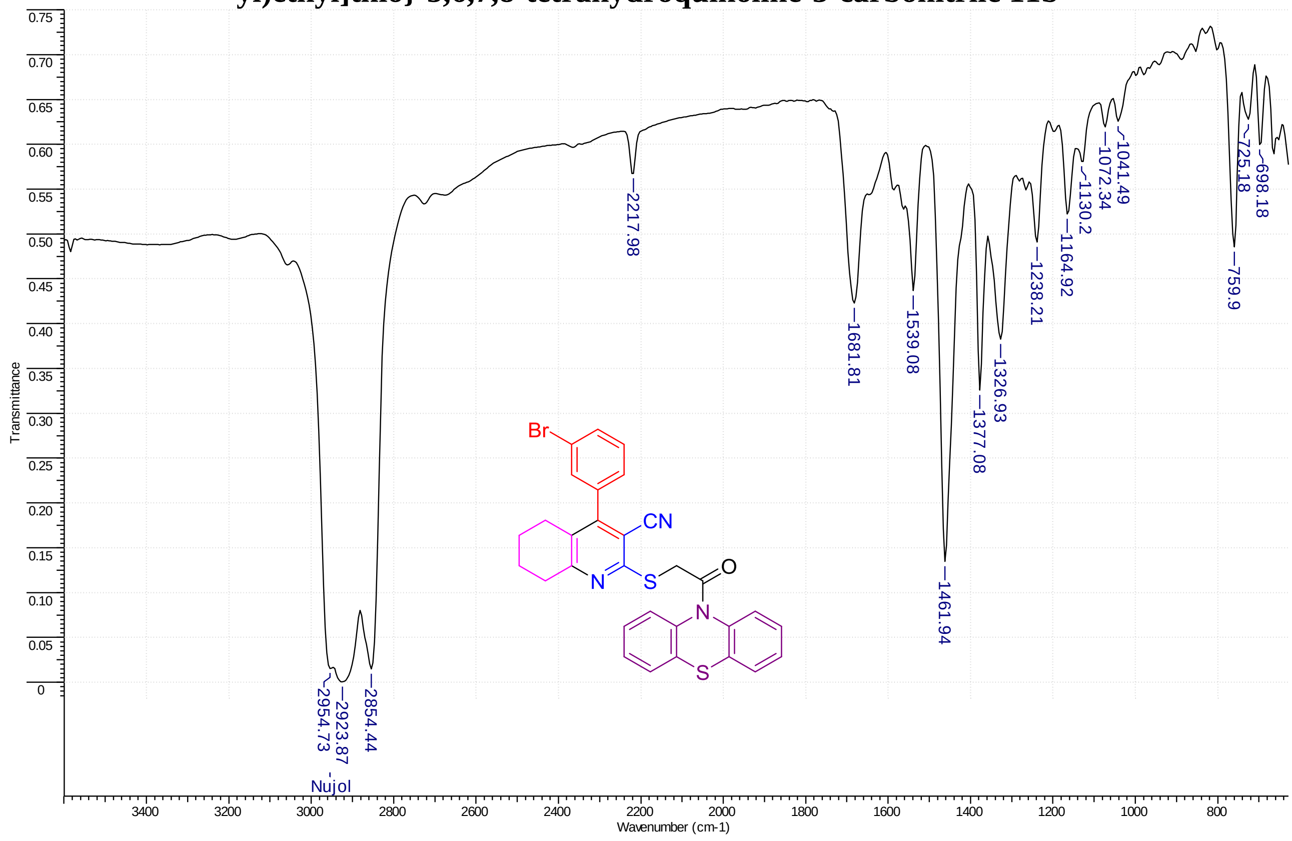

Figure S25. ^{1}H NMR spectrum (400 MHz, DMSO-d_6) of 4-(3-bromophenyl)-2-{[2-oxo-2-(10H-phenothiazin-10-yl)ethyl]thio}-5,6,7,8-tetrahydroquinoline-3-carbonitrile 11b

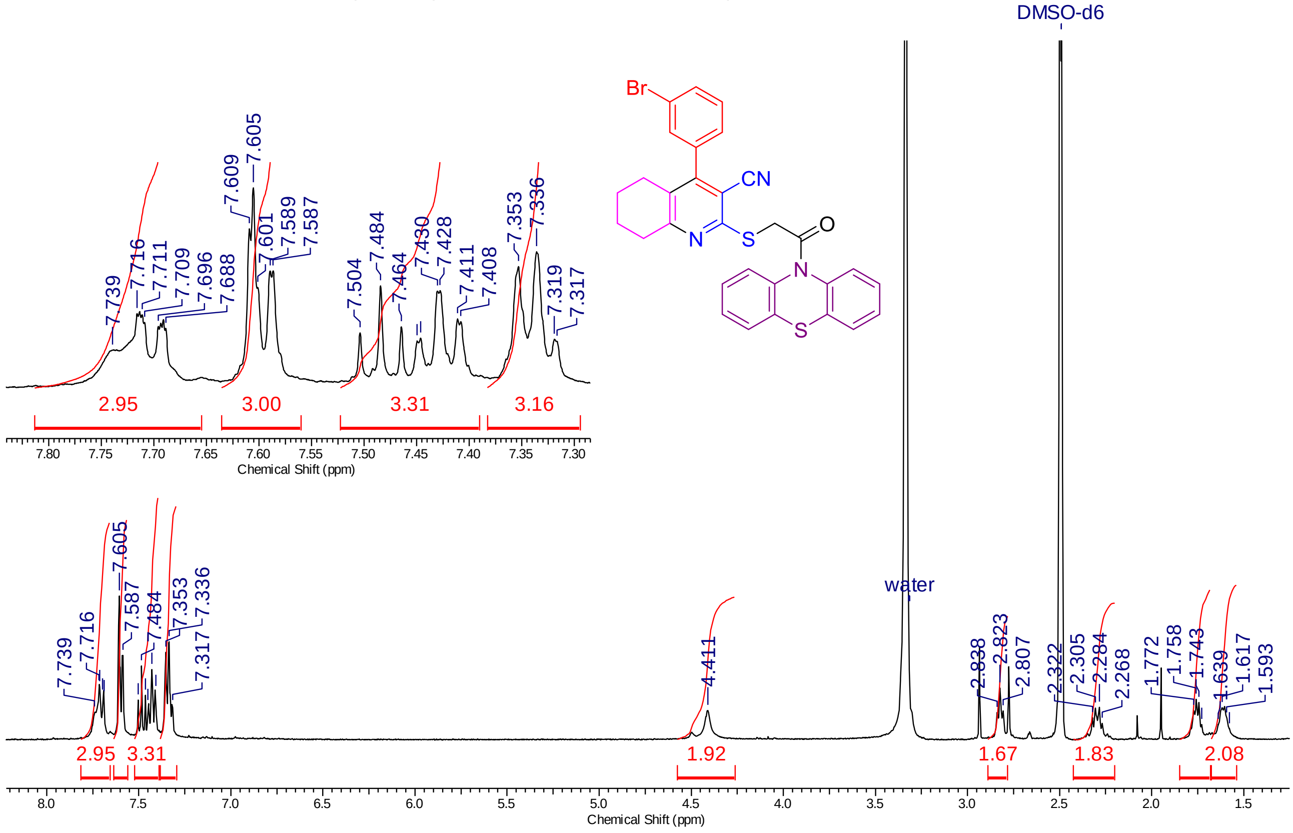

Figure S26. ^{13}C **DEPTQ NMR spectrum (101 MHz, DMSO-d_6) of 4-(3-bromophenyl)-2-{[2-oxo-2-(10H-phenothiazin-10-yl)ethyl]thio}-5,6,7,8-tetrahydroquinoline-3-carbonitrile 11b**

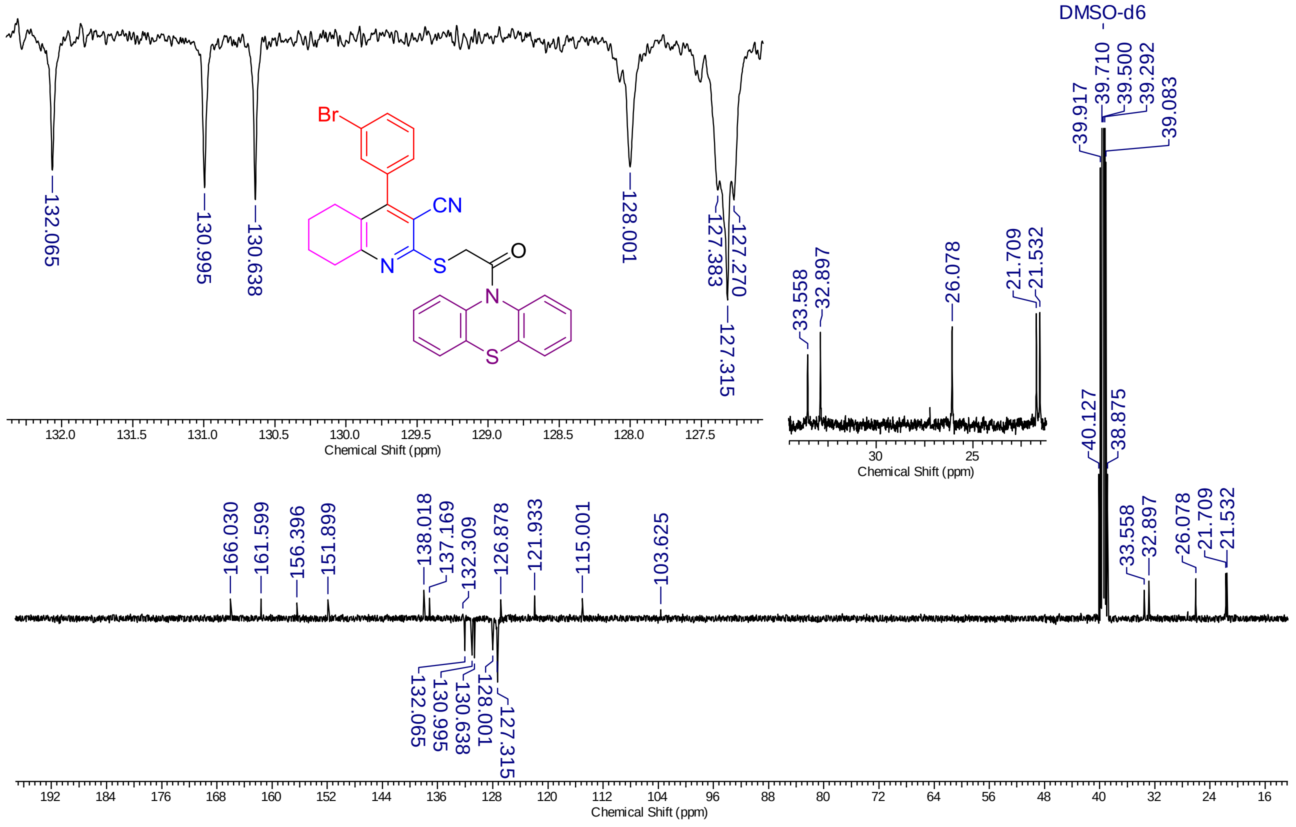

Figure S27. ^{1}H **NMR spectrum (400 MHz, DMSO-d_6) of 4-(4-fluorophenyl)-2-{[2-oxo-2-(10H-phenothiazin-10-yl)ethyl]thio}-5,6,7,8-tetrahydroquinoline-3-carbonitrile 11c**

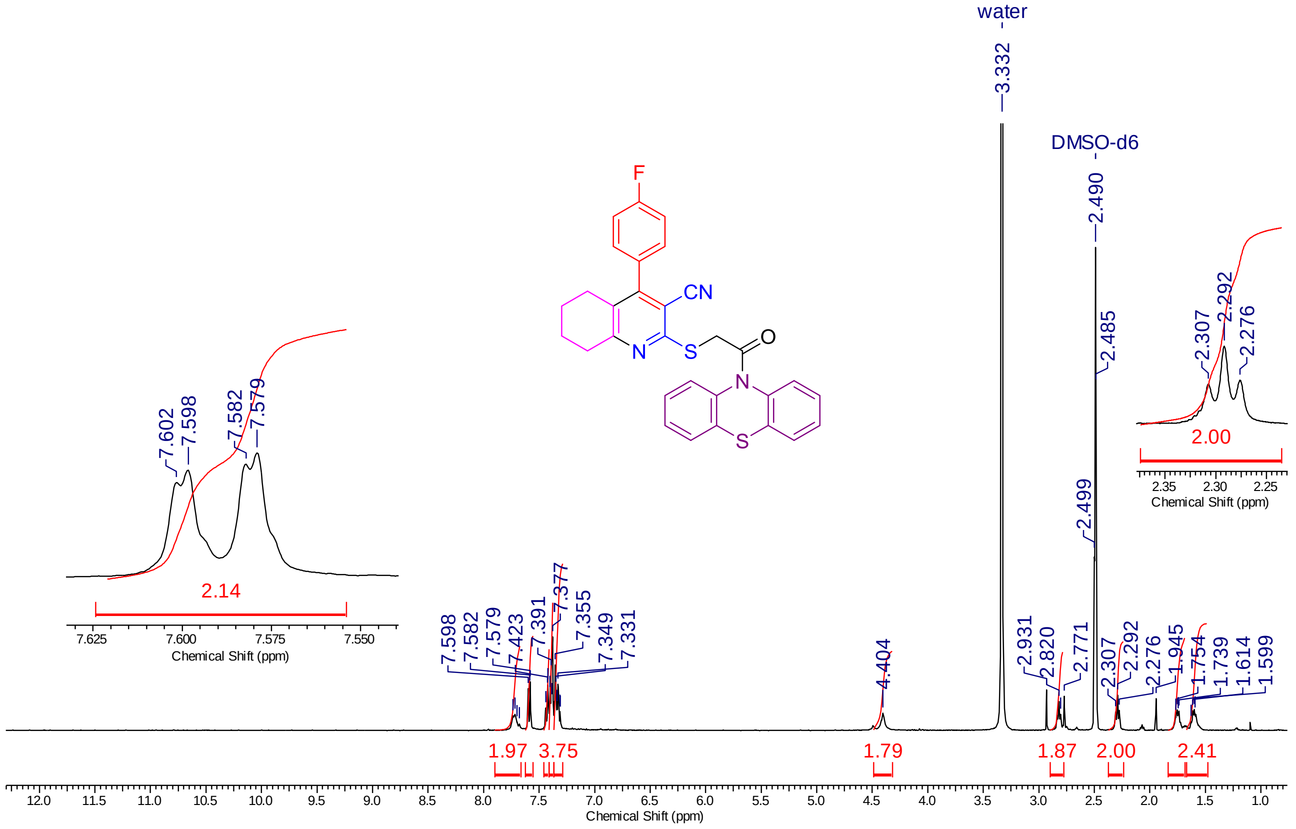

Figure S28. ^{13}C **DEPTQ NMR spectrum (101 MHz, DMSO-d$_6$) of 4-(4-fluorophenyl)-2-{[2-oxo-2-(10H-phenothiazin-10-yl)ethyl]thio}-5,6,7,8-tetrahydroquinoline-3-carbonitrile 11c**

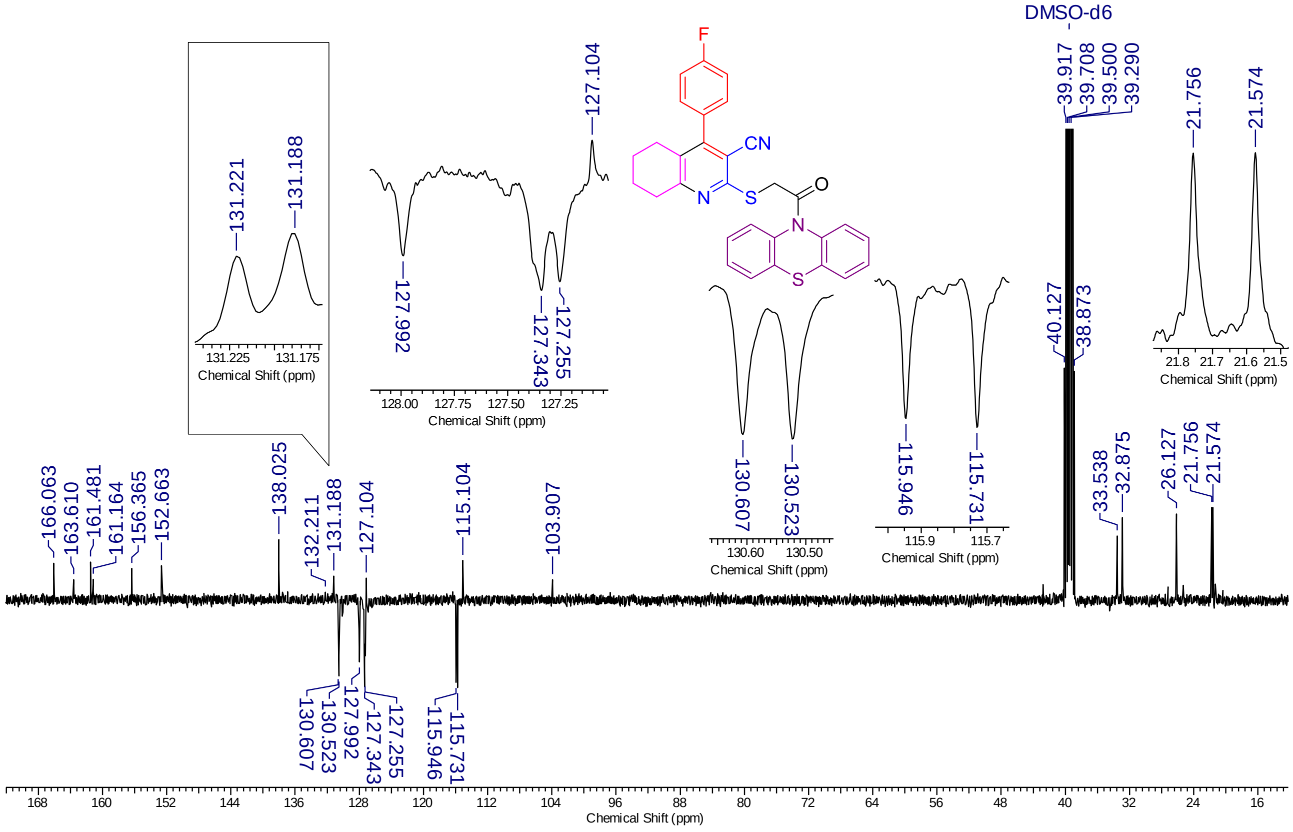

Figure S29. FTIR spectrum of 4-(2-thienyl)-2-{[2-oxo-2-(10H-phenothiazin-10-yl)ethyl]thio}-5,6,7,8-tetrahydroquinoline-3-carbonitrile 11d

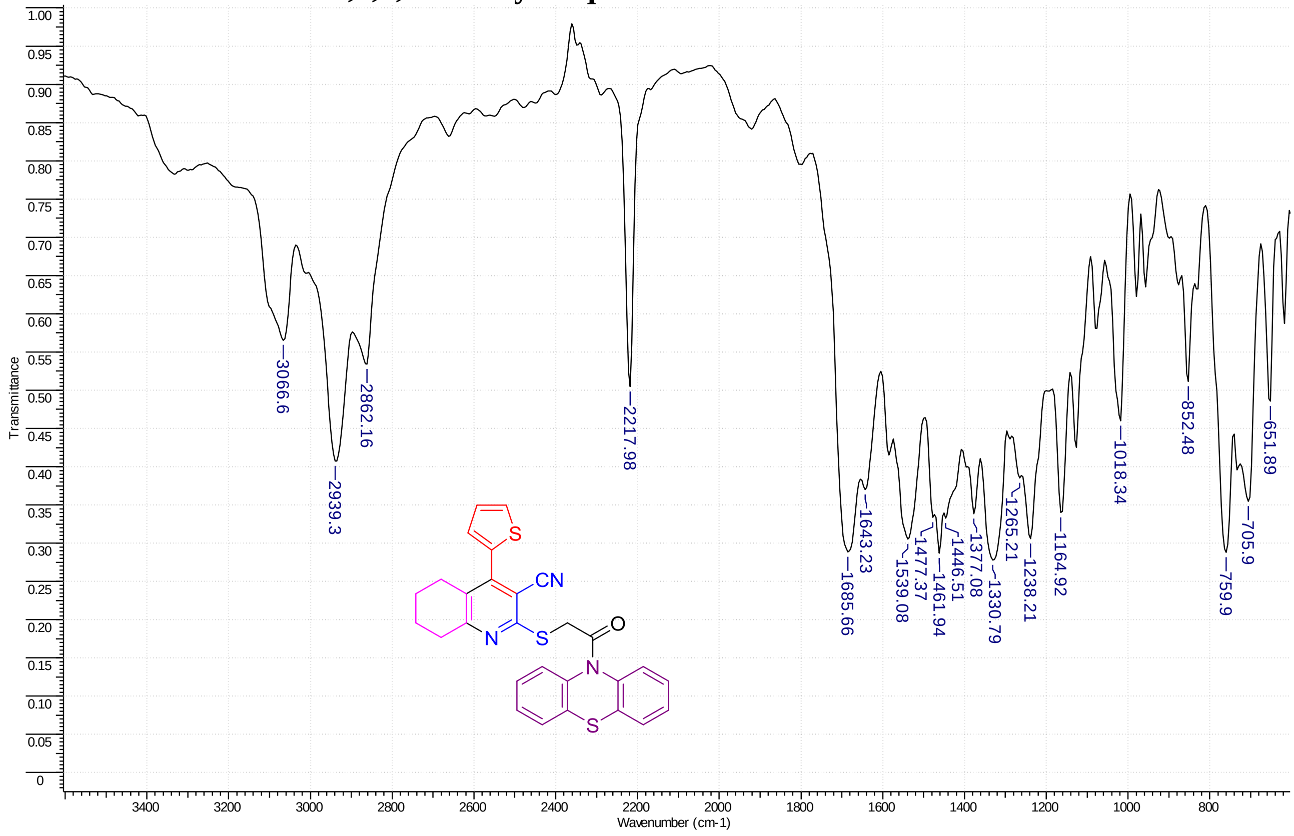

Figure S30. ^{1}**H NMR spectrum (400 MHz, DMSO-d$_6$) of 4-(2-thienyl)-2-{[2-oxo-2-(10H-phenothiazin-10-yl)ethyl]thio}-5,6,7,8-tetrahydroquinoline-3-carbonitrile 11d**

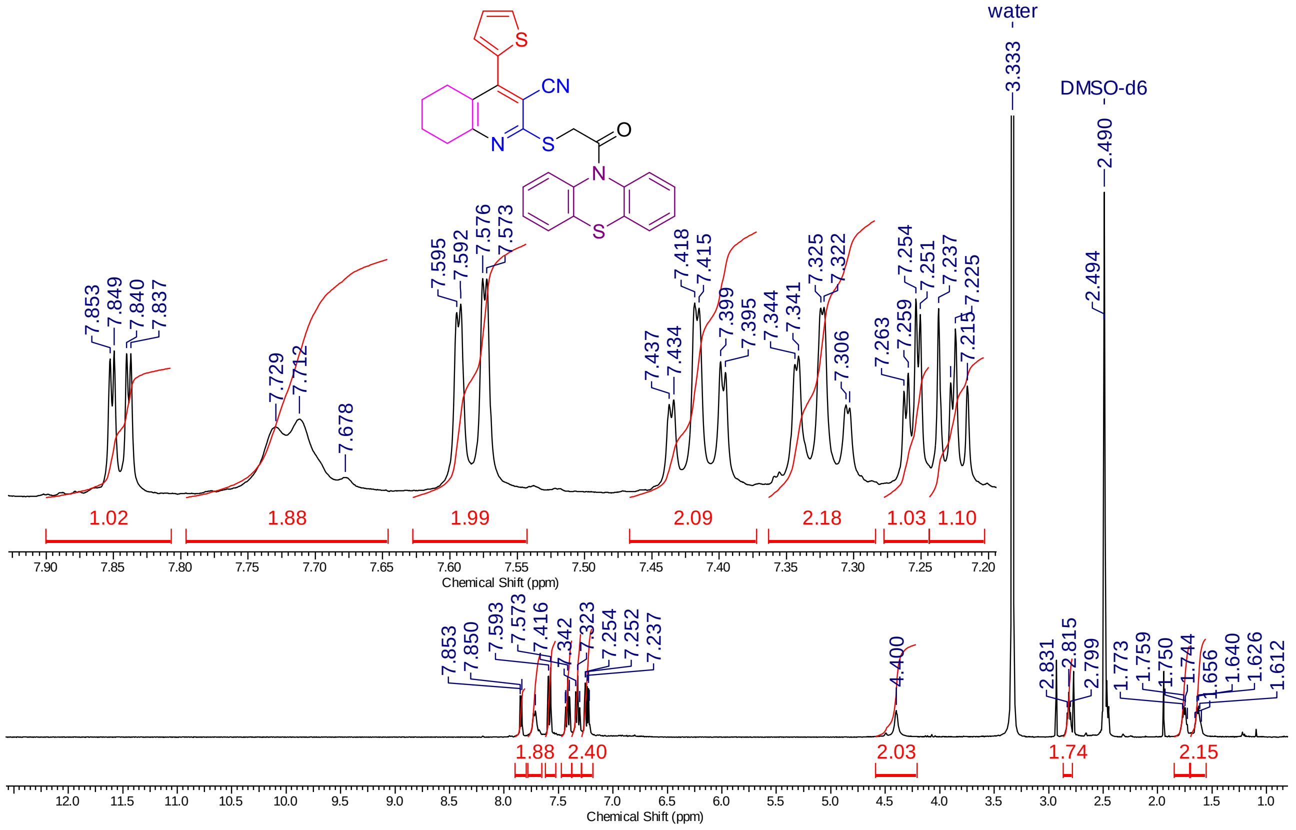

Figure S31. ^{13}**C DEPTQ NMR spectrum (101 MHz, DMSO-d$_6$) of 4-(2-thienyl)-2-{[2-oxo-2-(10H-phenothiazin-10-yl)ethyl]thio}-5,6,7,8-tetrahydroquinoline-3-carbonitrile 11d**

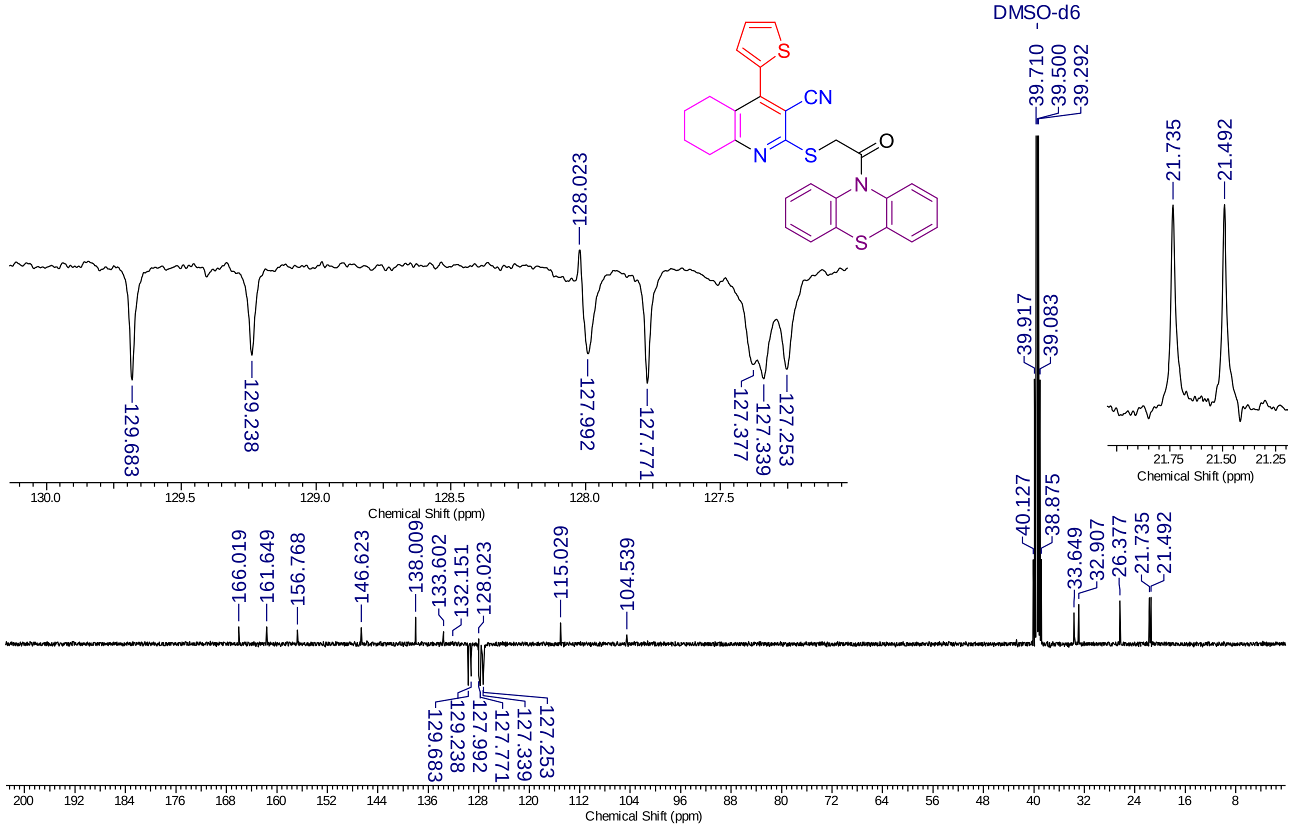

Figure S32. FTIR spectrum of 4-(2,4-dichlorophenyl)-2-{[2-oxo-2-(10H-phenothiazin-10-yl)ethyl]thio}-5,6,7,8-tetrahydroquinoline-3-carbonitrile 11e

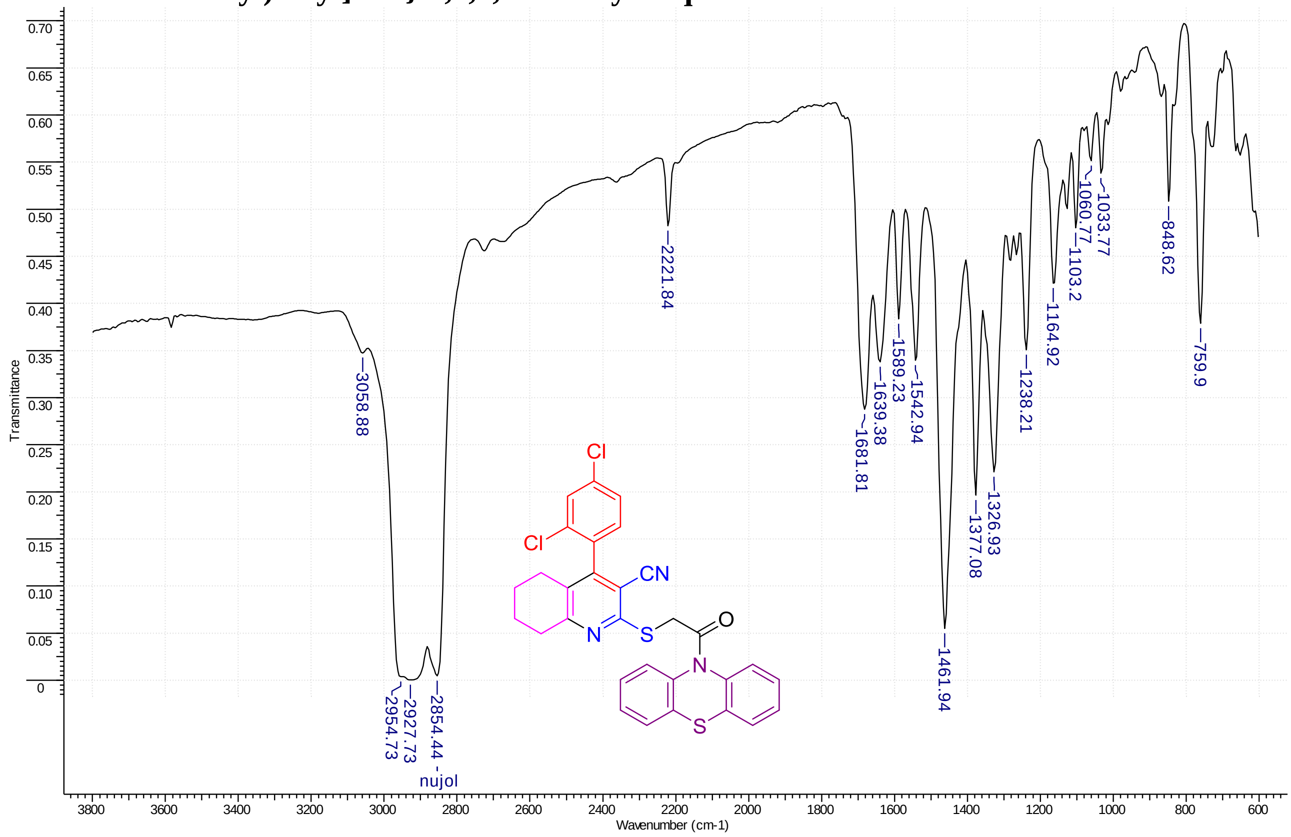

Figure S33. ^{1}H NMR spectrum (400 MHz, DMSO-d_6) of 4-(2,4-dichlorophenyl)-2-{[2-oxo-2-(10H-phenothiazin-10-yl)ethyl]thio}-5,6,7,8-tetrahydroquinoline-3-carbonitrile 11e

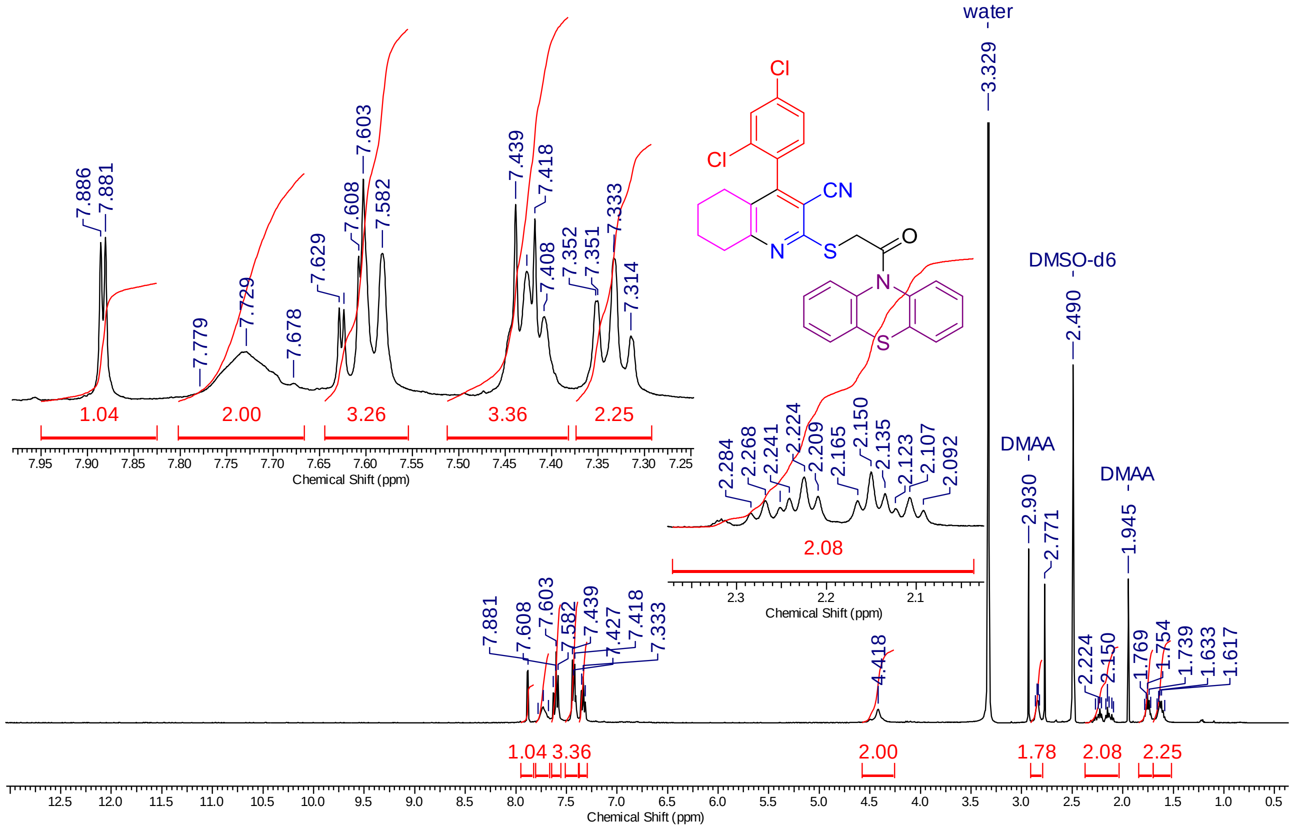

Figure S34. ^{13}C DEPTQ NMR spectrum (101 MHz, DMSO-d_6) of 4-(2,4-dichlorophenyl)-2-{[2-oxo-2-(10H-phenothiazin-10-yl)ethyl]thio}-5,6,7,8-tetrahydroquinoline-3-carbonitrile 11e

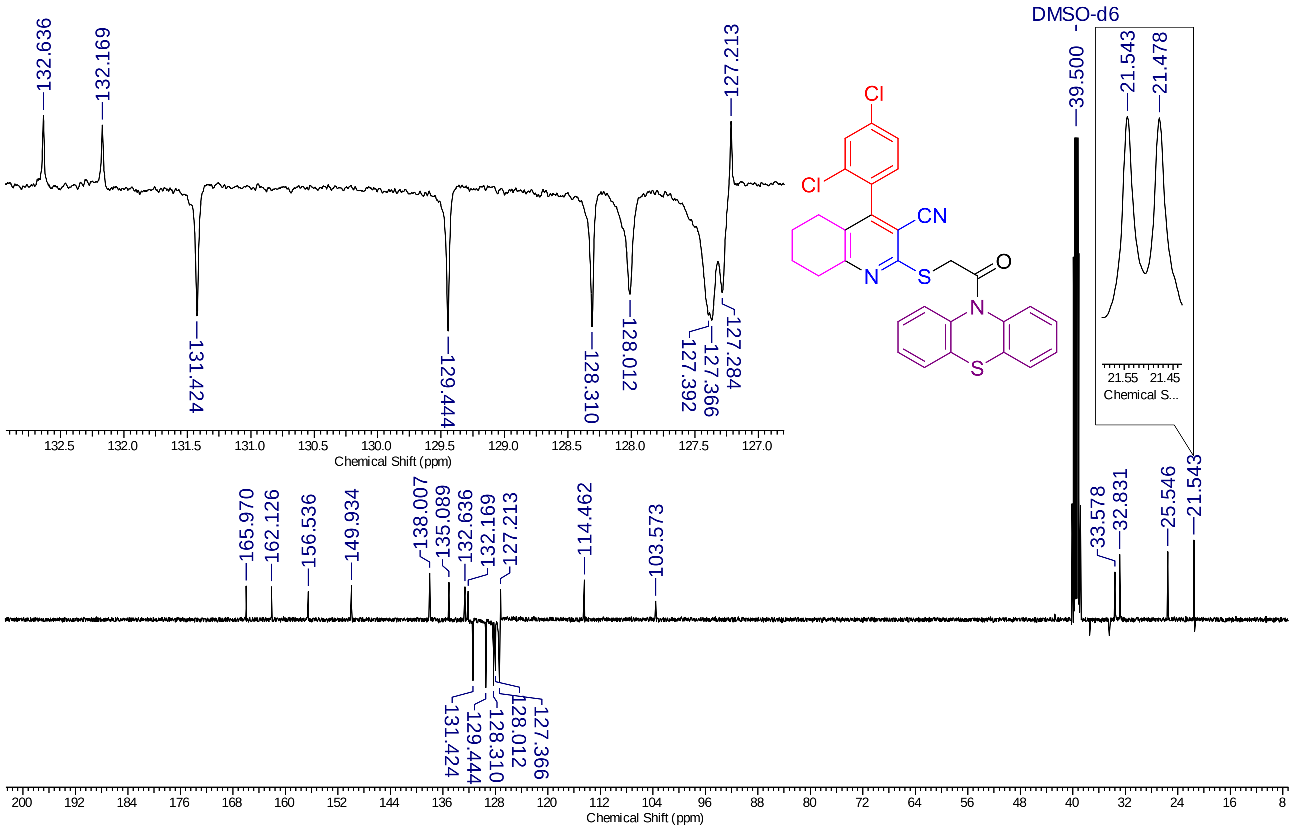

Figure S35. ^{1}H NMR spectrum (400 MHz, DMSO-d_6) of 4-(4-methylphenyl)-2-{[2-oxo-2-(10H-phenothiazin-10-yl)ethyl]thio}-5,6,7,8-tetrahydroquinoline-3-carbonitrile 11f

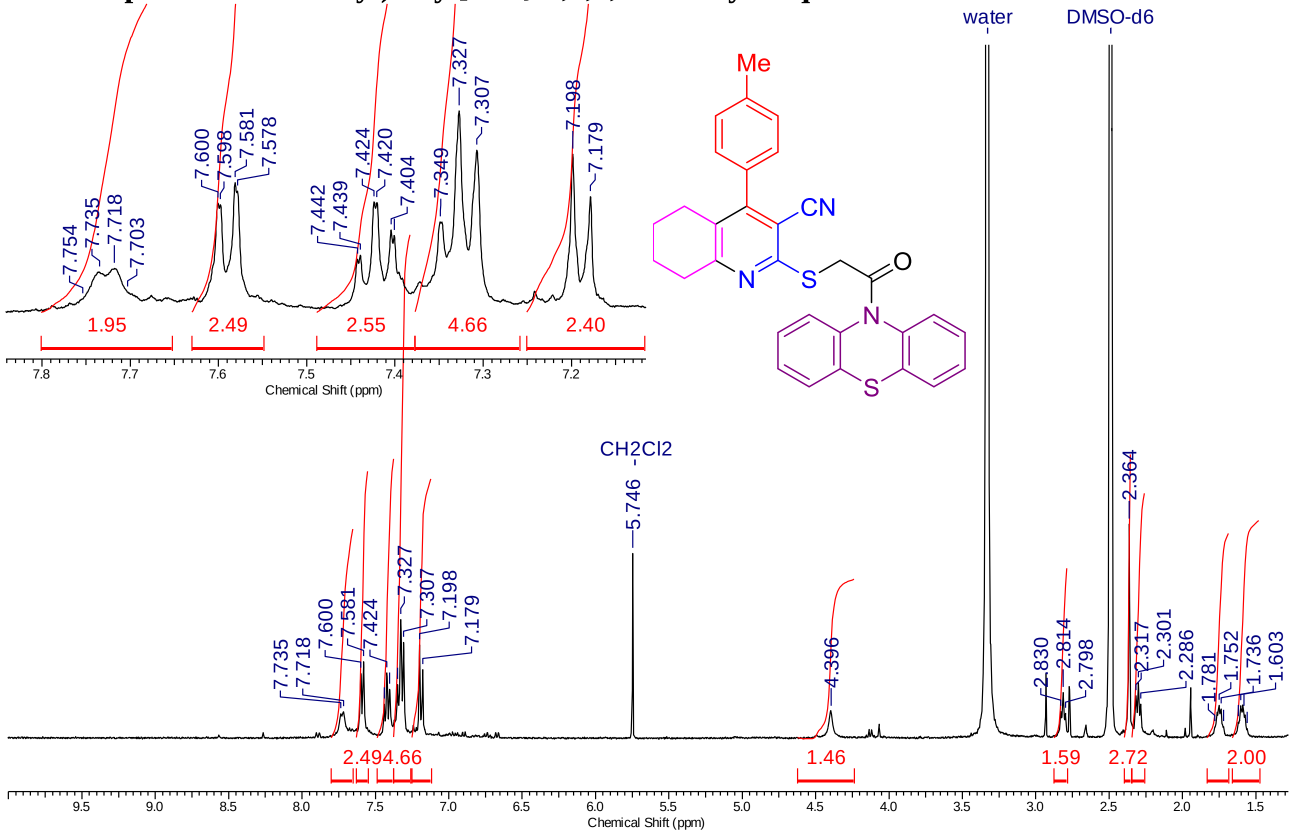

Figure S36. ^{13}C DEPTQ NMR spectrum (101 MHz, DMSO-d_6) of 4-(4-methylphenyl)-2-{[2-oxo-2-(10H-phenothiazin-10-yl)ethyl]thio}-5,6,7,8-tetrahydroquinoline-3-carbonitrile 11f

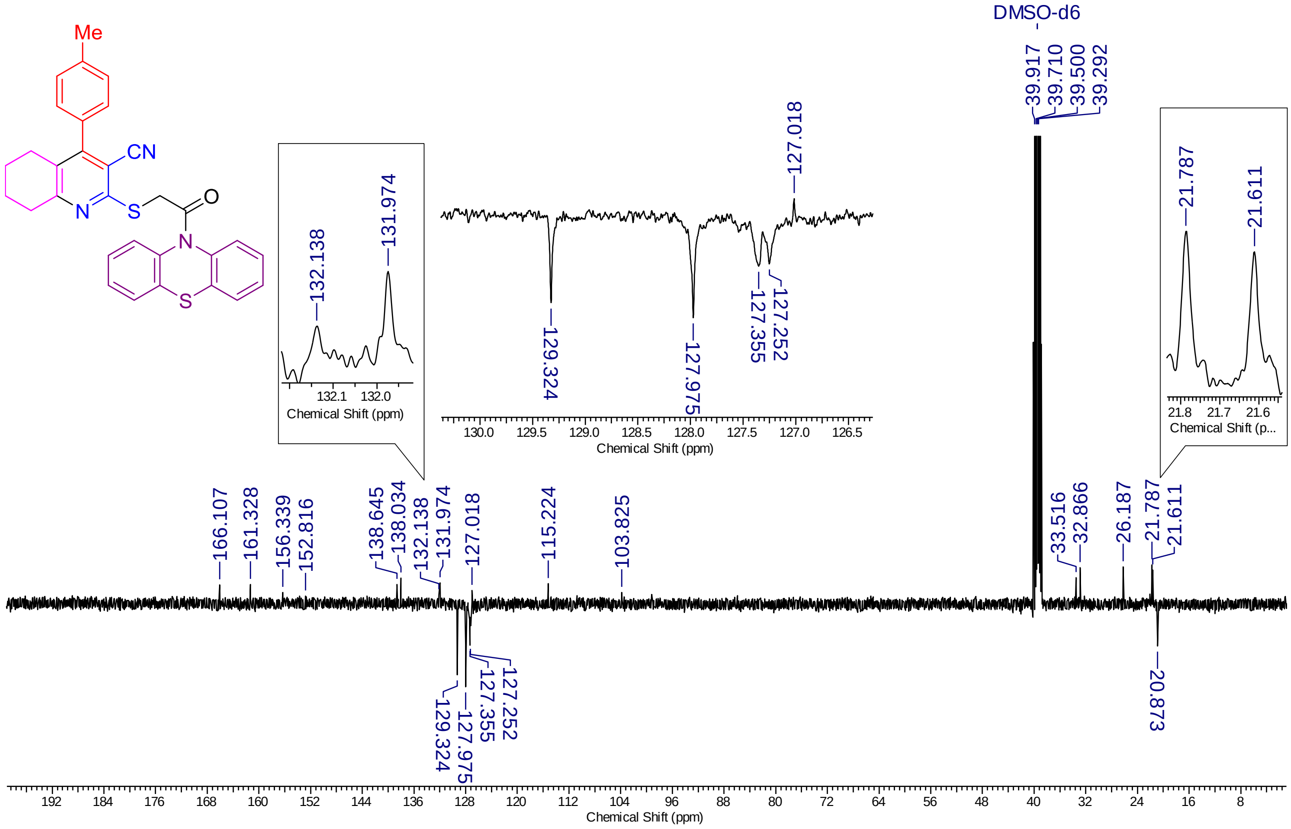

Figure S37. ^{1}H NMR spectrum (400 MHz, DMSO-d_6) of 4-(3-nitrophenyl)-2-{[2-oxo-2-(10H-phenothiazin-10-yl)ethyl]thio}-5,6,7,8-tetrahydroquinoline-3-carbonitrile 11g

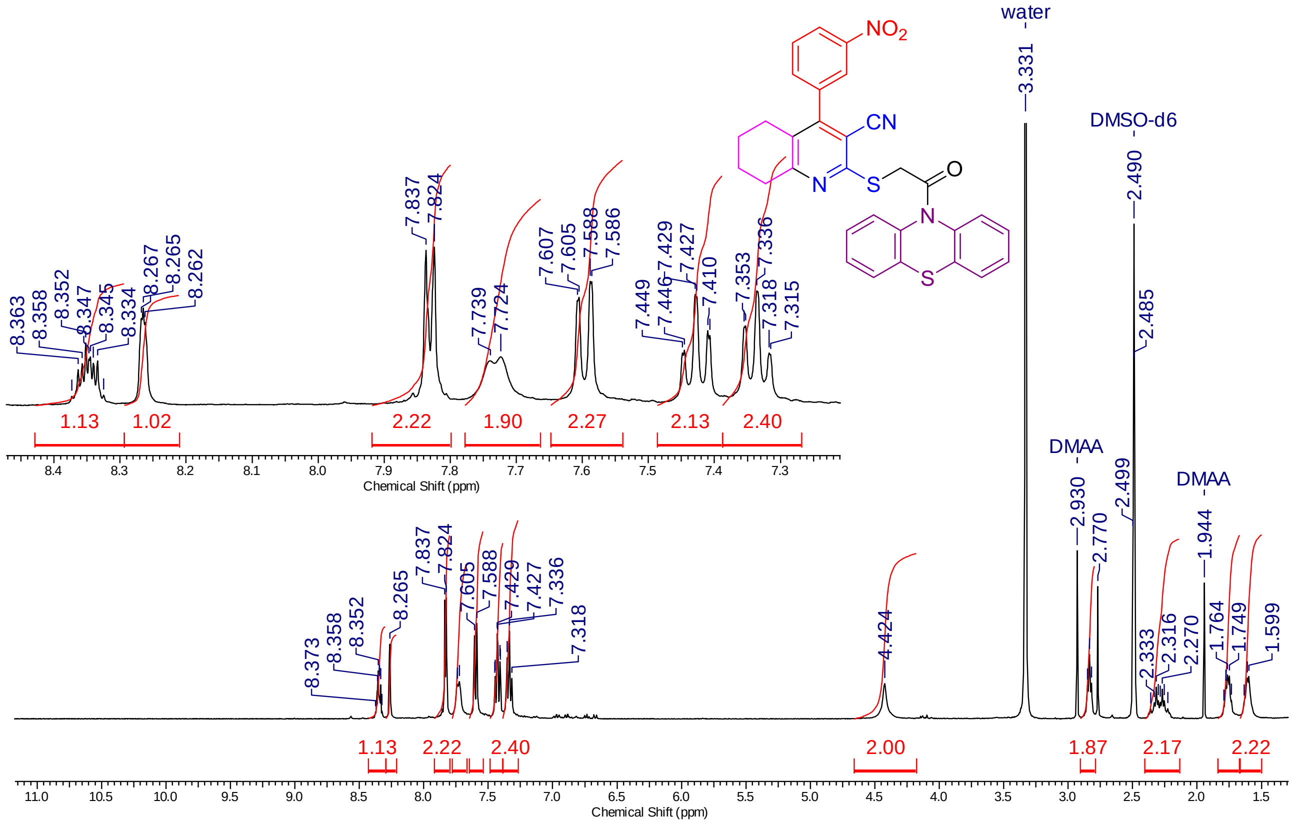

Figure S38. ^{13}C DEPTQ NMR spectrum (101 MHz, DMSO-d_6) of 4-(3-nitrophenyl)-2-{[2-oxo-2-(10H-phenothiazin-10-yl)ethyl]thio}-5,6,7,8-tetrahydroquinoline-3-carbonitrile 11g

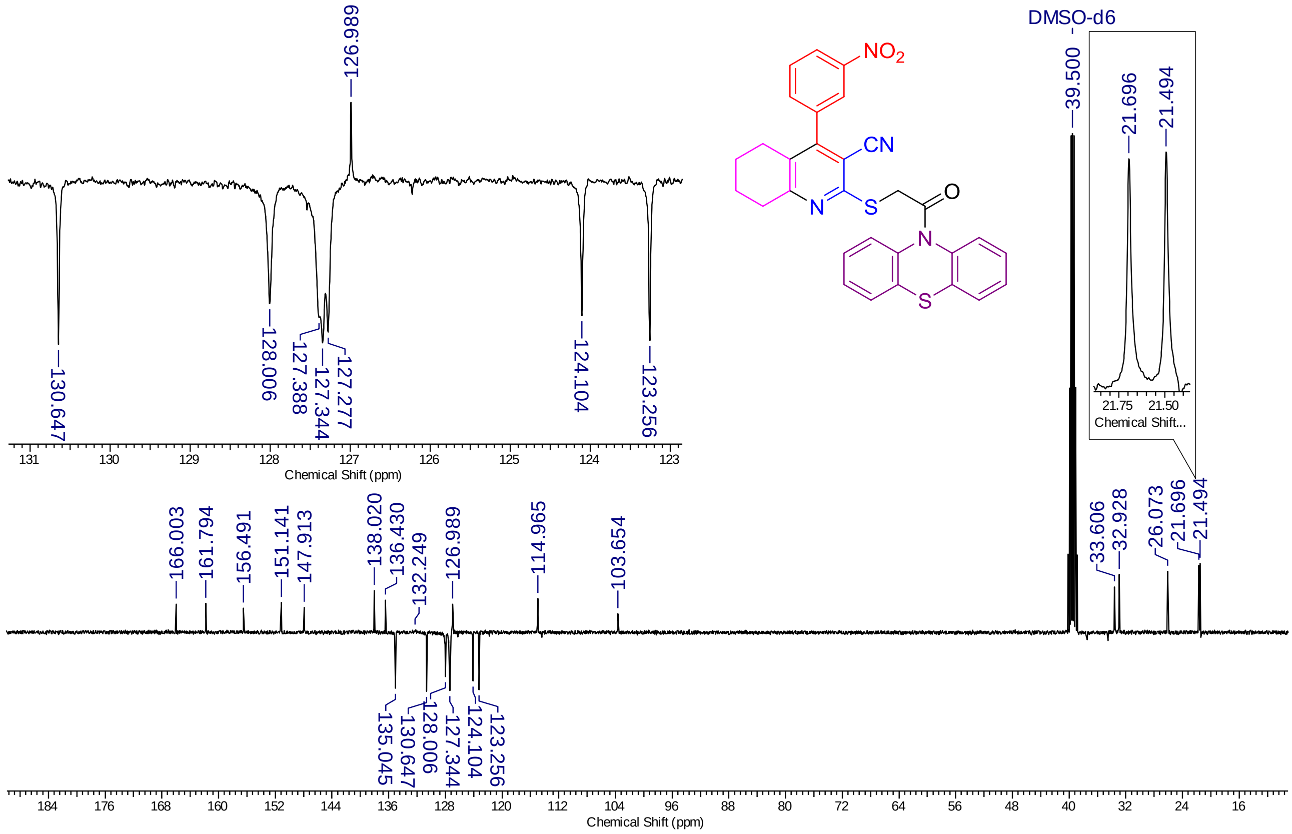

Figure S39. FTIR spectrum of 4-(2-furyl)-2-{[2-oxo-2-(10H-phenothiazin-10-yl)ethyl]thio}-5,6,7,8-tetrahydroquinoline-3-carbonitrile 11h

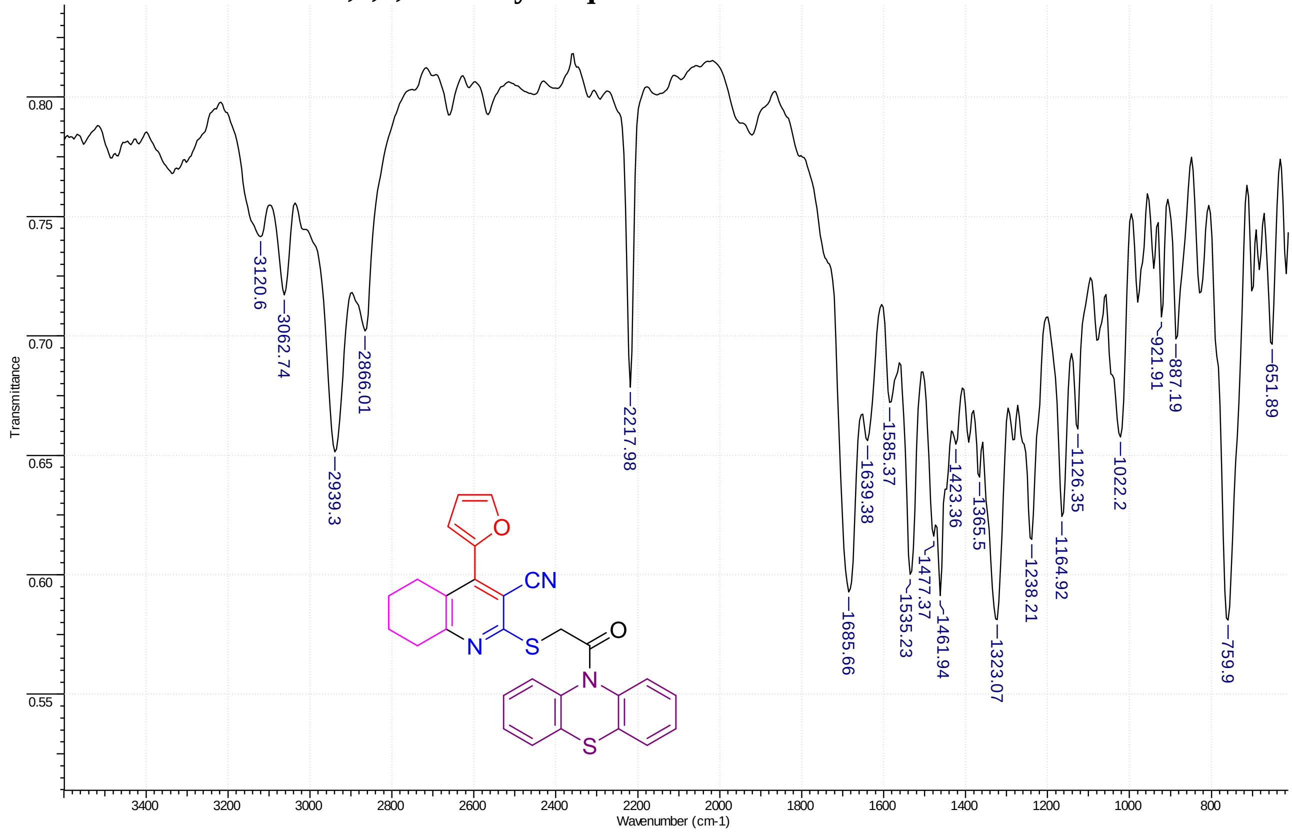

Figure S40. ^{1}H **NMR spectrum (400 MHz, DMSO-d_6) of 4-(2-furyl)-2-{[2-oxo-2-(10H-phenothiazin-10-yl)ethyl]thio}-5,6,7,8-tetrahydroquinoline-3-carbonitrile 11h**

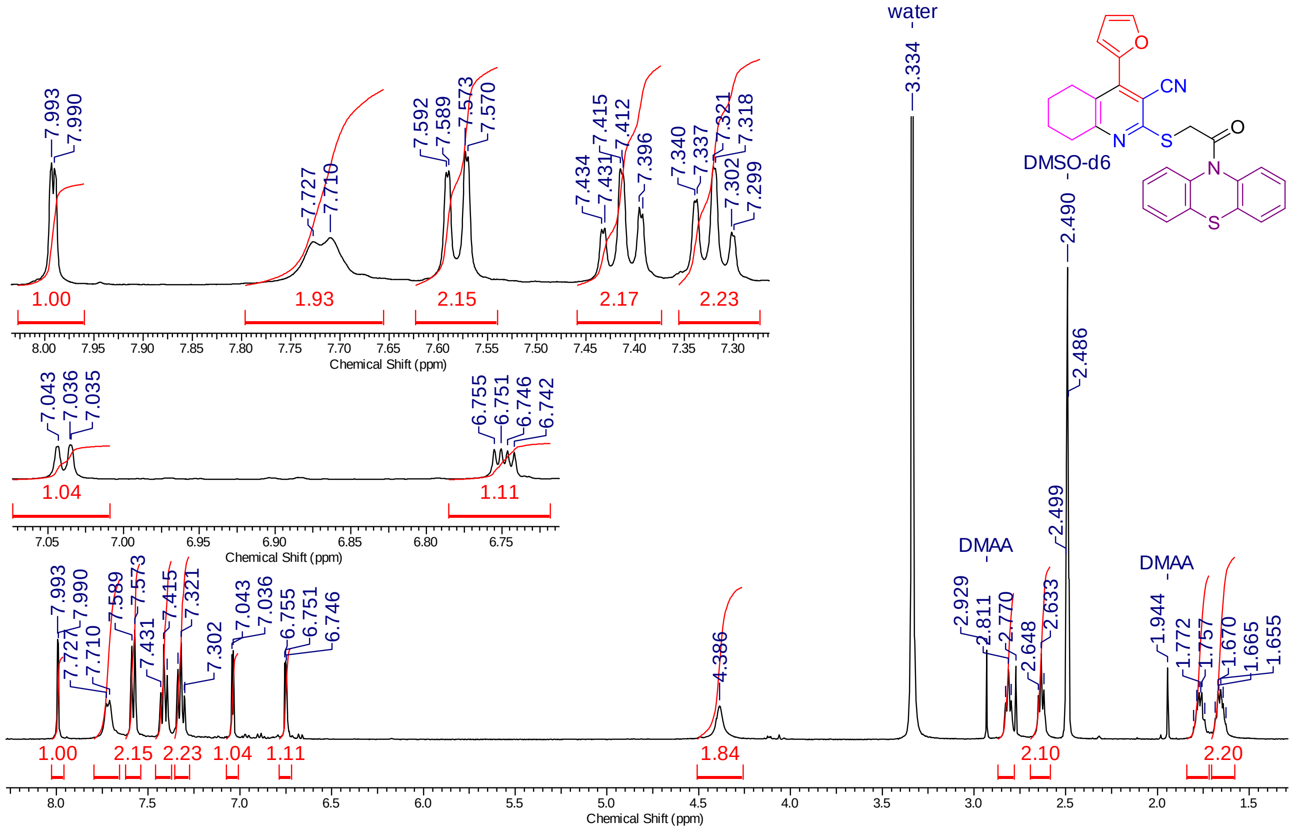

Figure S41. ^{13}C **DEPTQ NMR spectrum (101 MHz, DMSO-d_6) of 4-(2-furyl)-2-{[2-oxo-2-(10H-phenothiazin-10-yl)ethyl]thio}-5,6,7,8-tetrahydroquinoline-3-carbonitrile 11h**

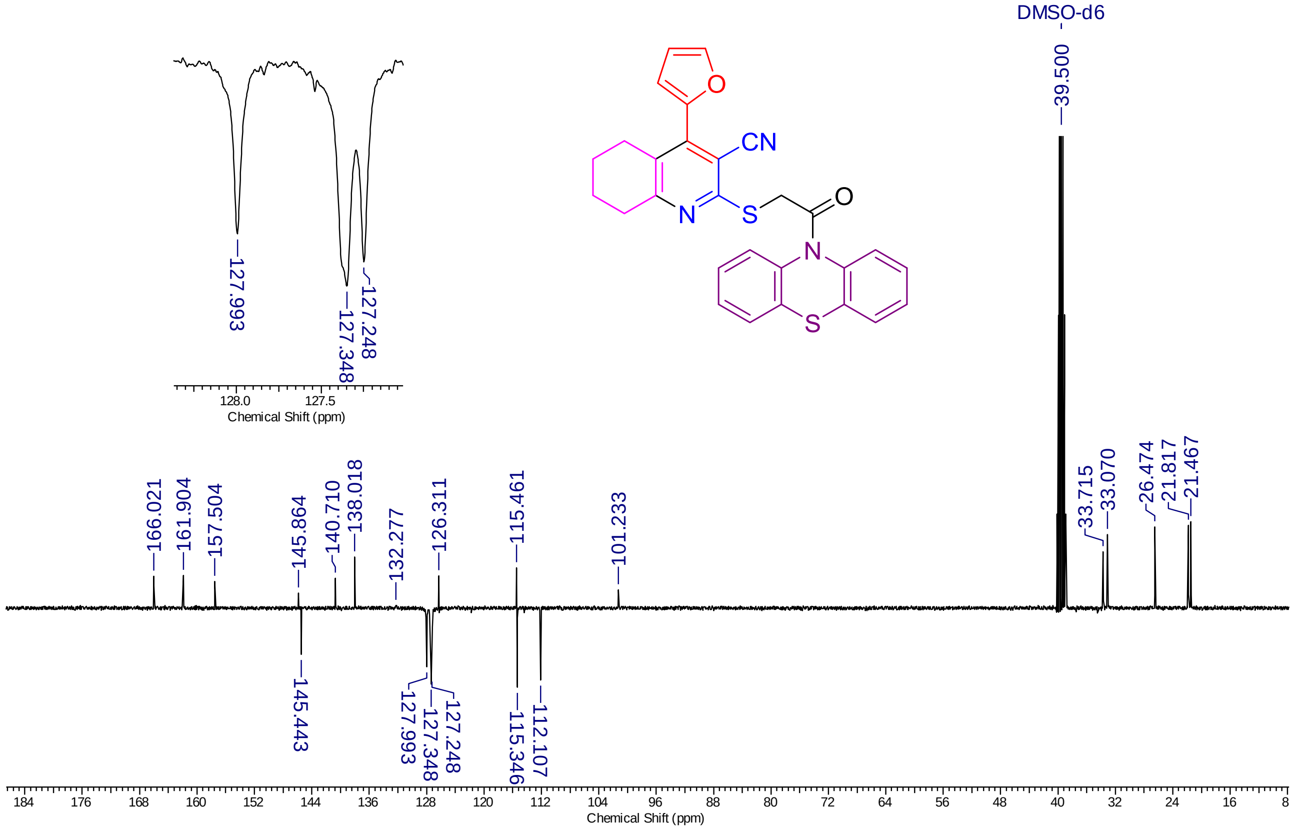

Figure S42. [1]**H NMR spectrum (400 MHz, DMSO-d$_6$) of phenothiazine (PhTz)**

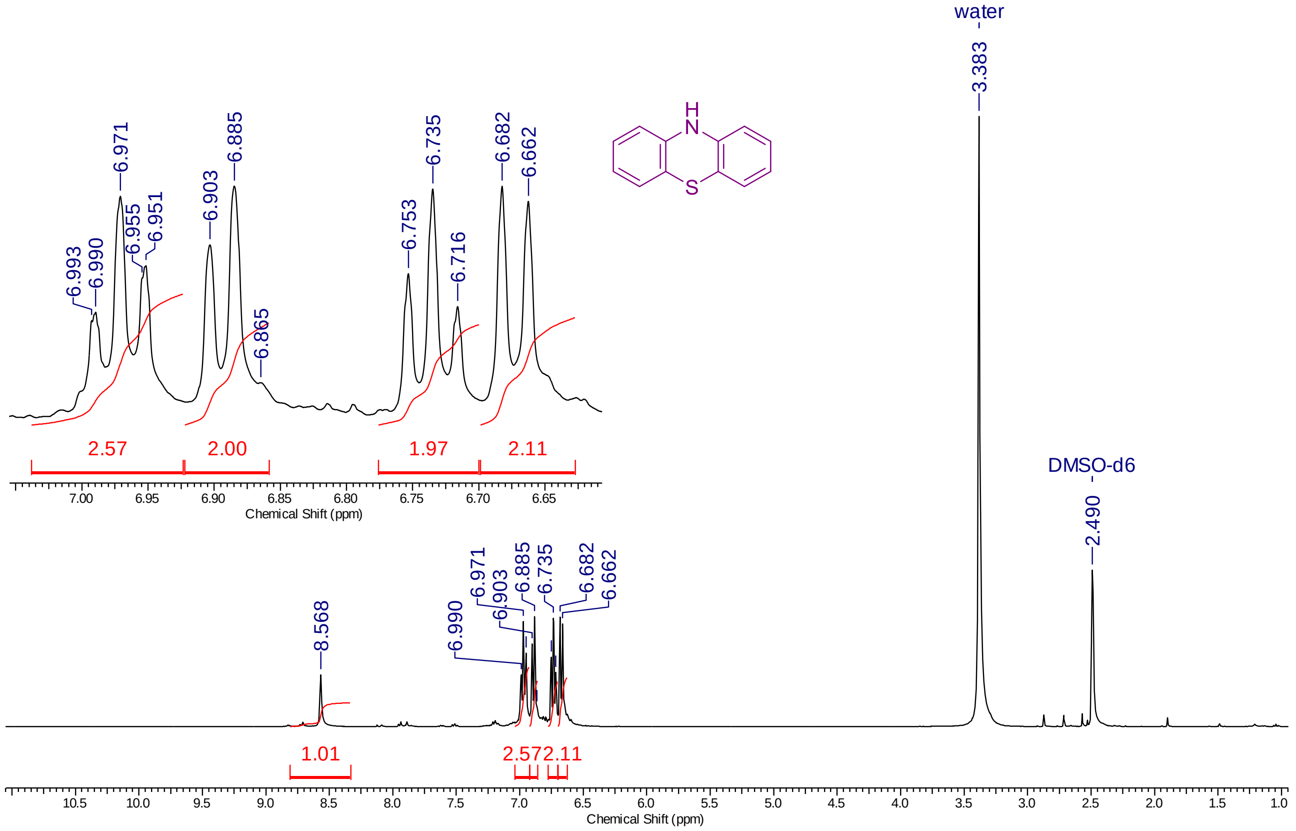

Figure S43. [13]**C DEPTQ NMR spectrum (101 MHz, DMSO-d$_6$) of phenothiazine(PhTz)**

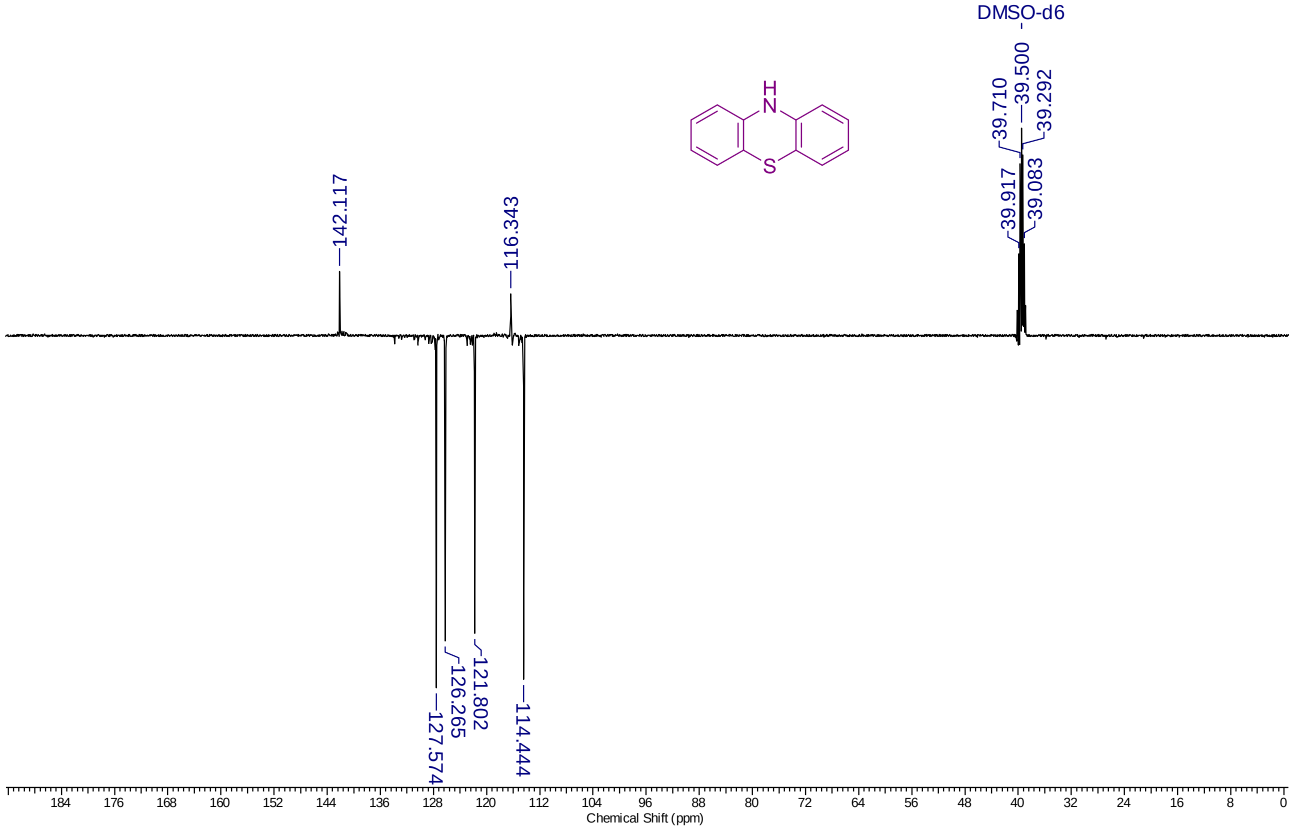

Figure S44. FTIR spectrum of (3-amino-4-(4-chlorophenyl)-5,6,7,8-tetrahydrothieno[2,3-b]quinolin-2-yl)(10H-phenothiazin-10-yl)methanone 12a

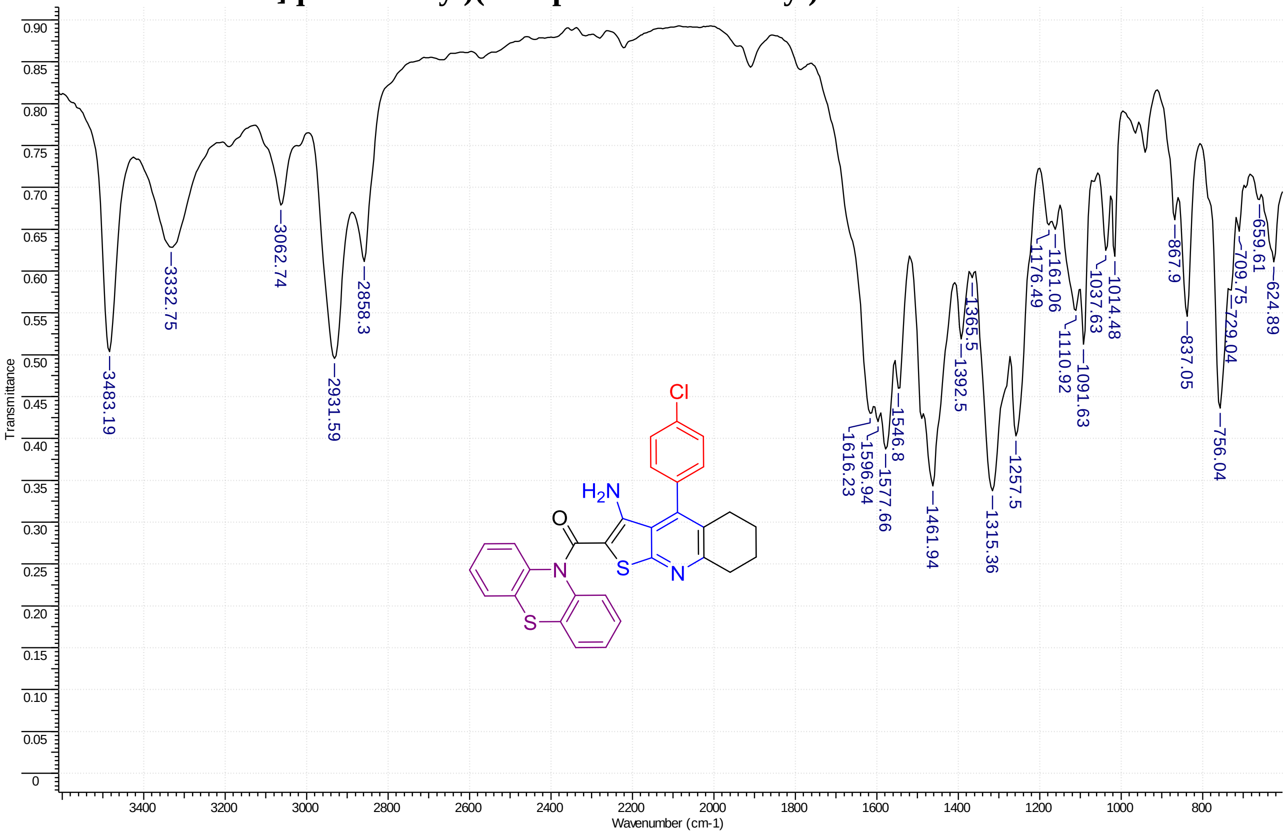

Figure S45. ^{1}H NMR spectrum (400 MHz, DMSO-d$_6$) of crude (3-amino-4-(4-chlorophenyl)-5,6,7,8-tetrahydrothieno[2,3-b]quinolin-2-yl)(10H-phenothiazin-10-yl)methanone 12a

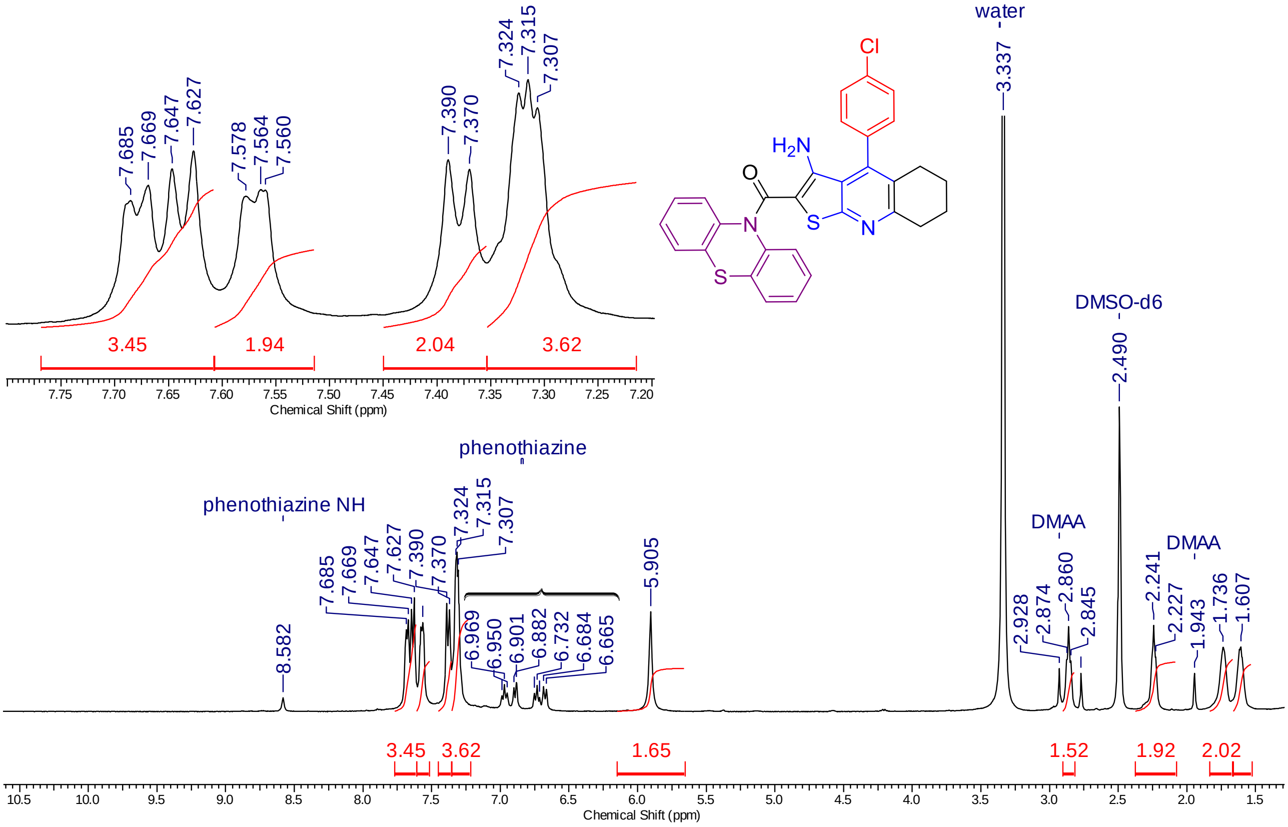

Figure S46. ^{13}C DEPTQ NMR spectrum (101 MHz, DMSO-d_6) of crude (3-amino-4-(4-chlorophenyl)-5,6,7,8-tetrahydrothieno[2,3-b]quinolin-2-yl)(10H-phenothiazin-10-yl)methanone 12a

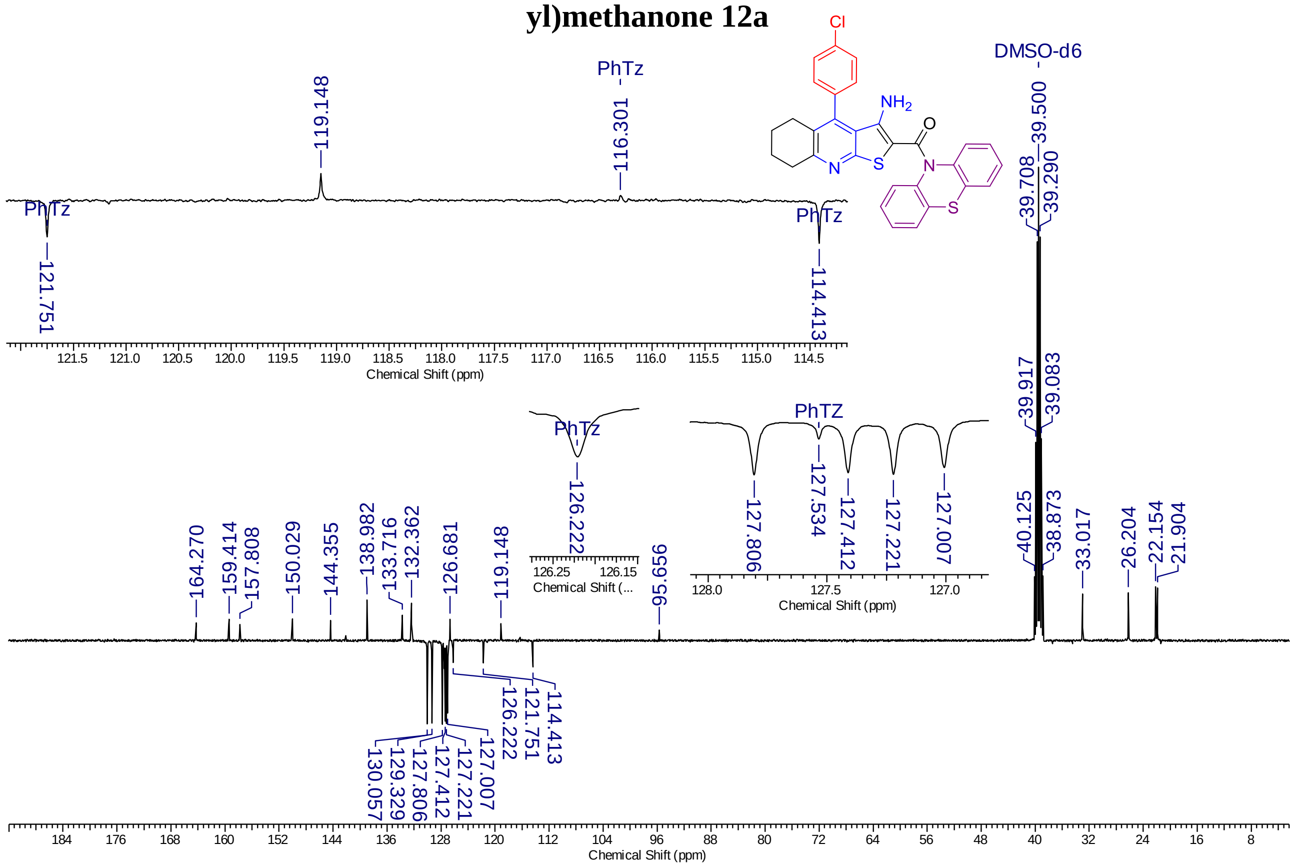

Figure S47. FTIR spectrum of (3-amino-4-(3-bromophenyl)-5,6,7,8-tetrahydrothieno[2,3-b]quinolin-2-yl)(10H-phenothiazin-10-yl)methanone 12b

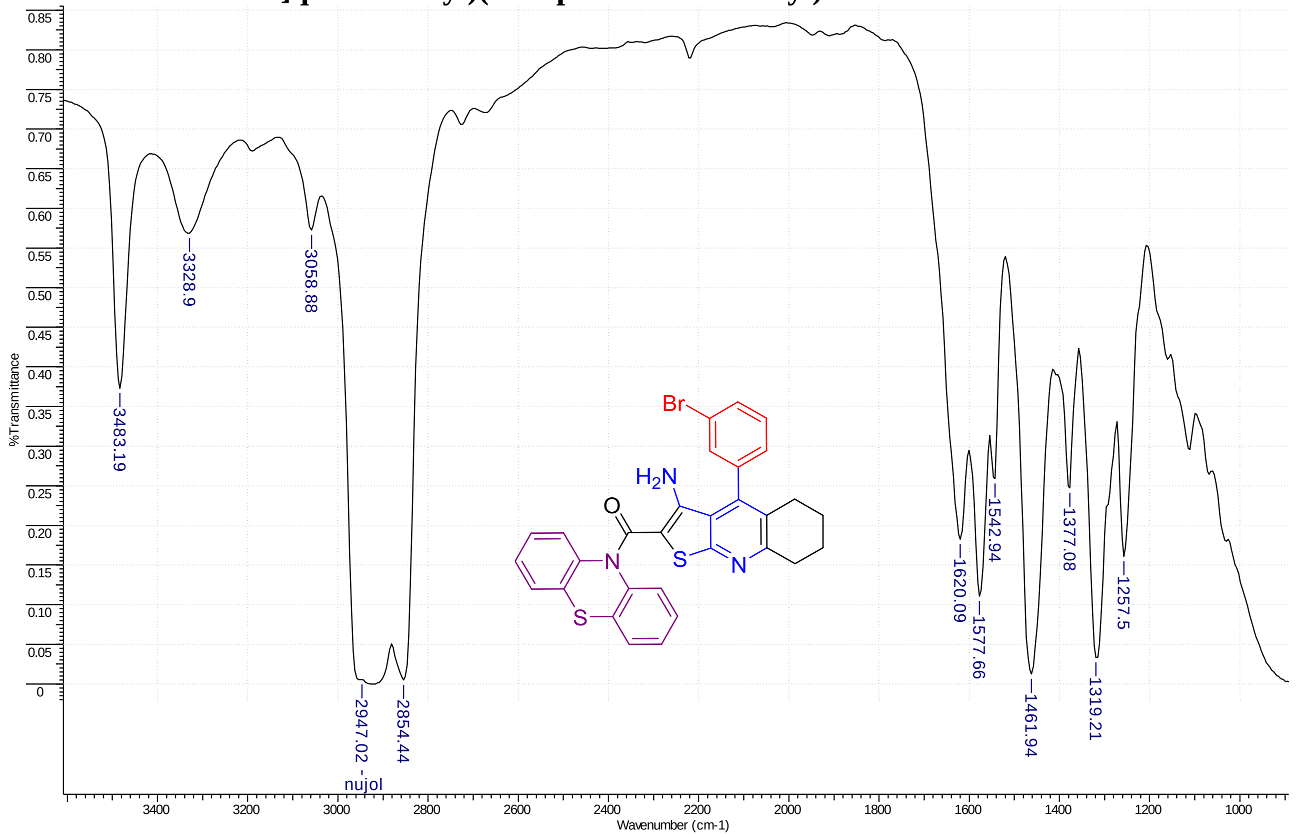

Figure S48. ^{1}**H NMR spectrum (400 MHz, DMSO-d$_6$) of crude (3-amino-4-(3-bromophenyl)-5,6,7,8-tetrahydrothieno[2,3-b]quinolin-2-yl)(10H-phenothiazin-10-yl)methanone 12b**

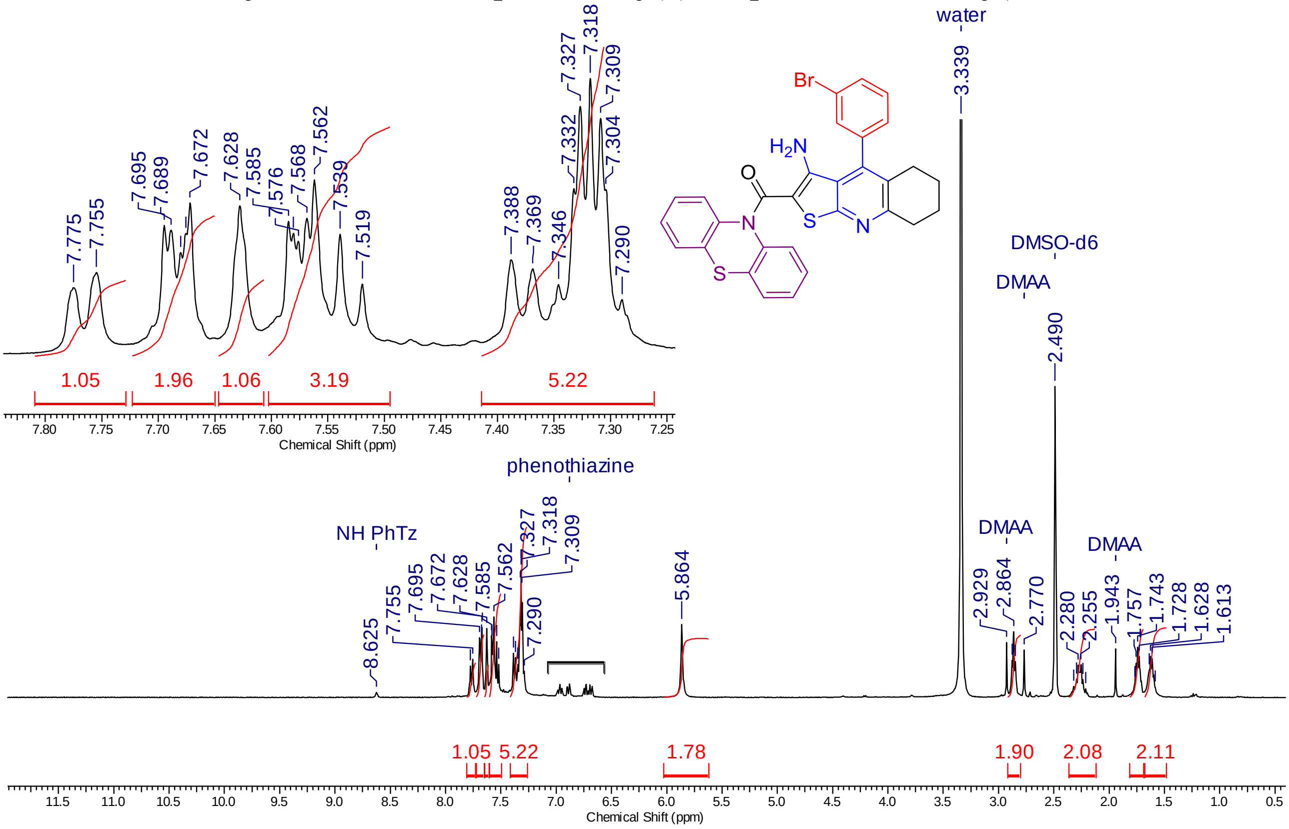

Figure S49. ^{13}**C DEPTQ NMR spectrum (101 MHz, DMSO-d$_6$) of crude (3-amino-4-(3-bromophenyl)-5,6,7,8-tetrahydrothieno[2,3-b]quinolin-2-yl)(10H-phenothiazin-10-yl)methanone 12b**

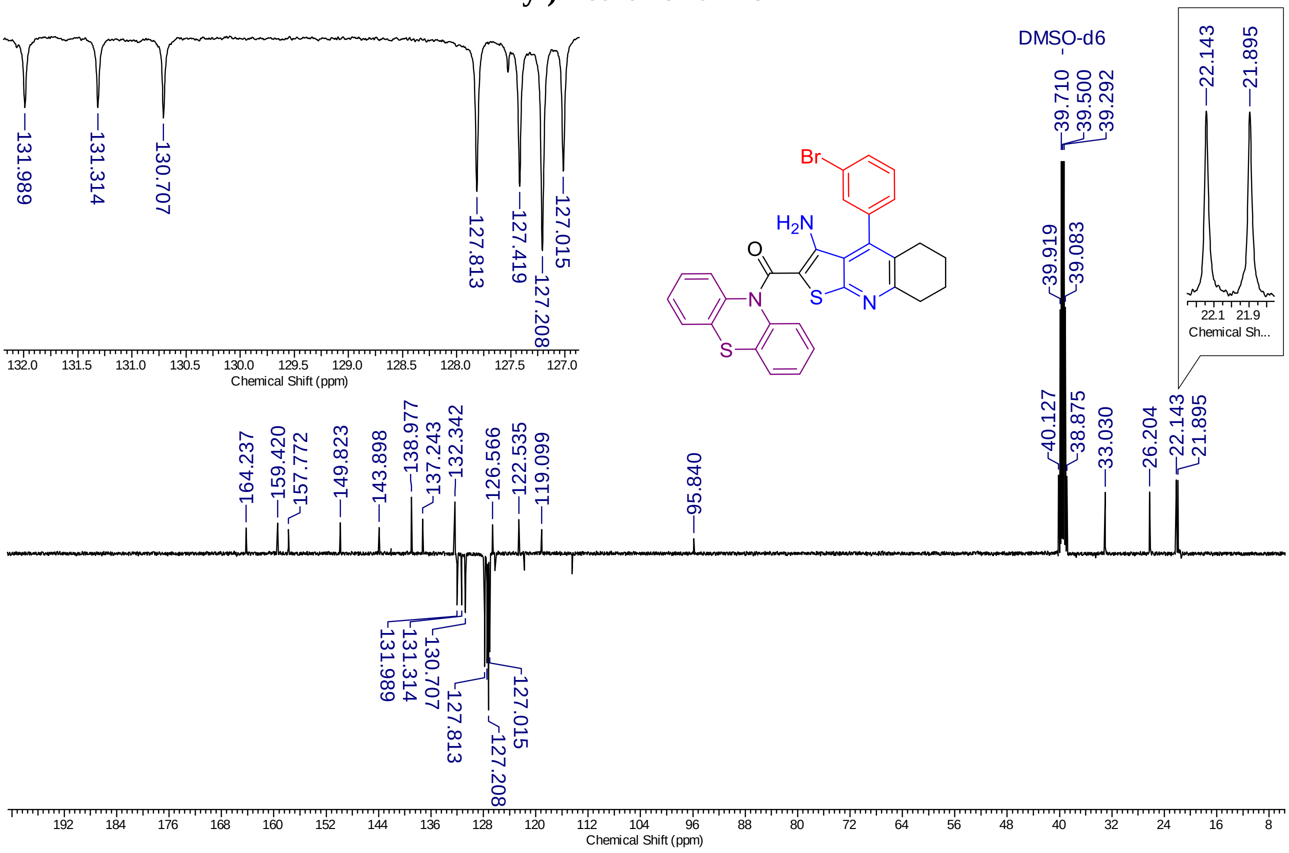

Figure S50. FTIR spectrum of (3-amino-4-(2-thienyl)-5,6,7,8-tetrahydrothieno[2,3-b]quinolin-2-yl)(10H-phenothiazin-10-yl)methanone 12d

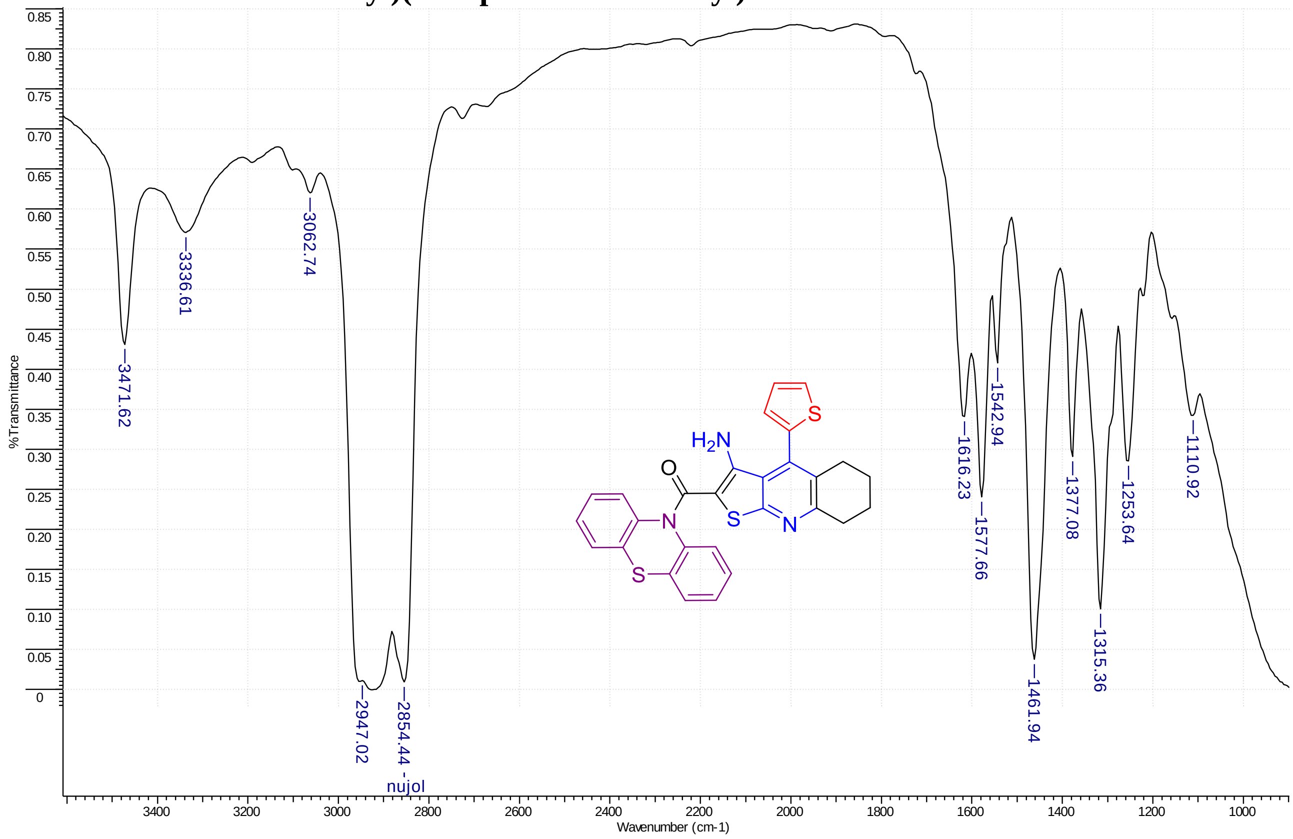

Figure S51. ^{1}H NMR spectrum (400 MHz, DMSO-d_6) of crude (3-amino-4-(2-thienyl)-5,6,7,8-tetrahydrothieno[2,3-b]quinolin-2-yl)(10H-phenothiazin-10-yl)methanone 12d

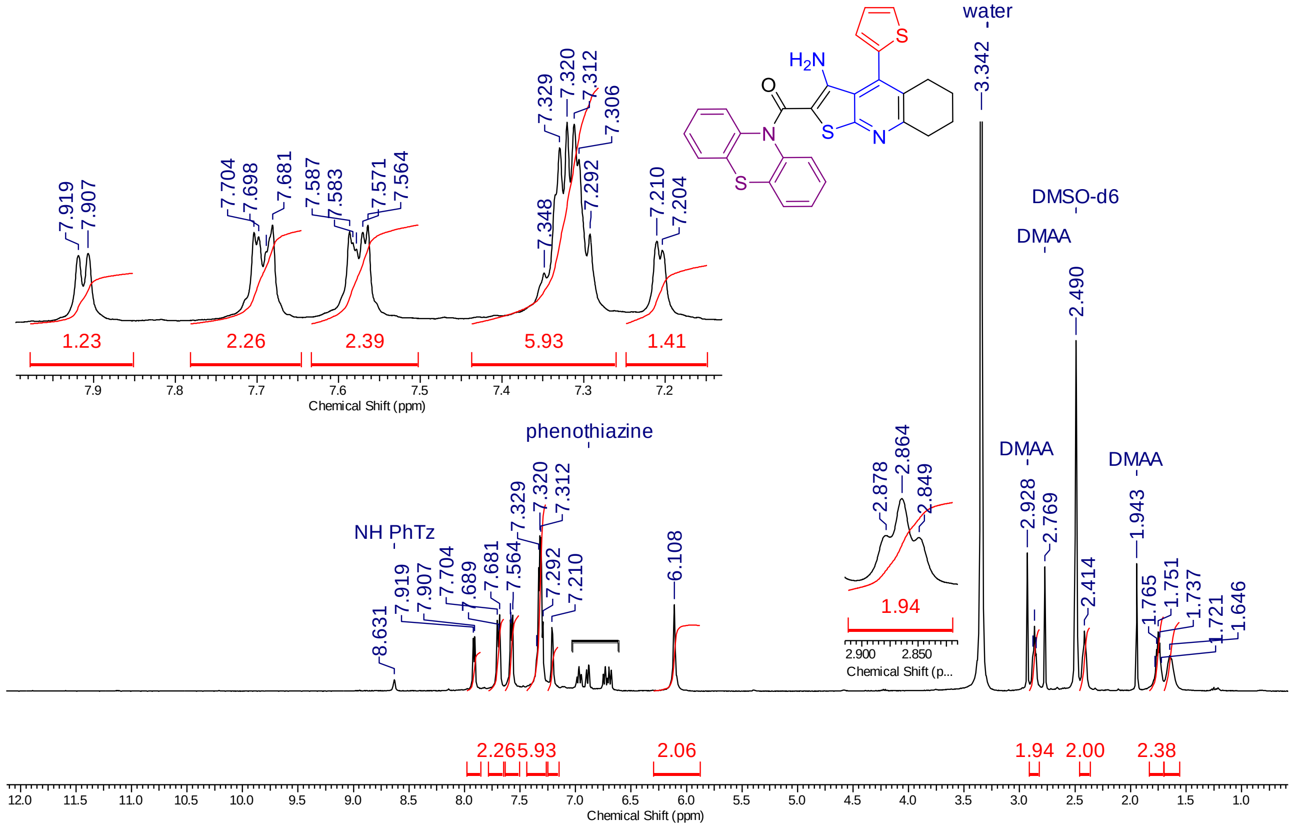

Figure S52. ^{13}C **DEPTQ NMR spectrum (101 MHz, DMSO-d$_6$) of crude (3-amino-4-(2-thienyl)-5,6,7,8-tetrahydrothieno[2,3-b]quinolin-2-yl)(10H-phenothiazin-10-yl)methanone 12d**

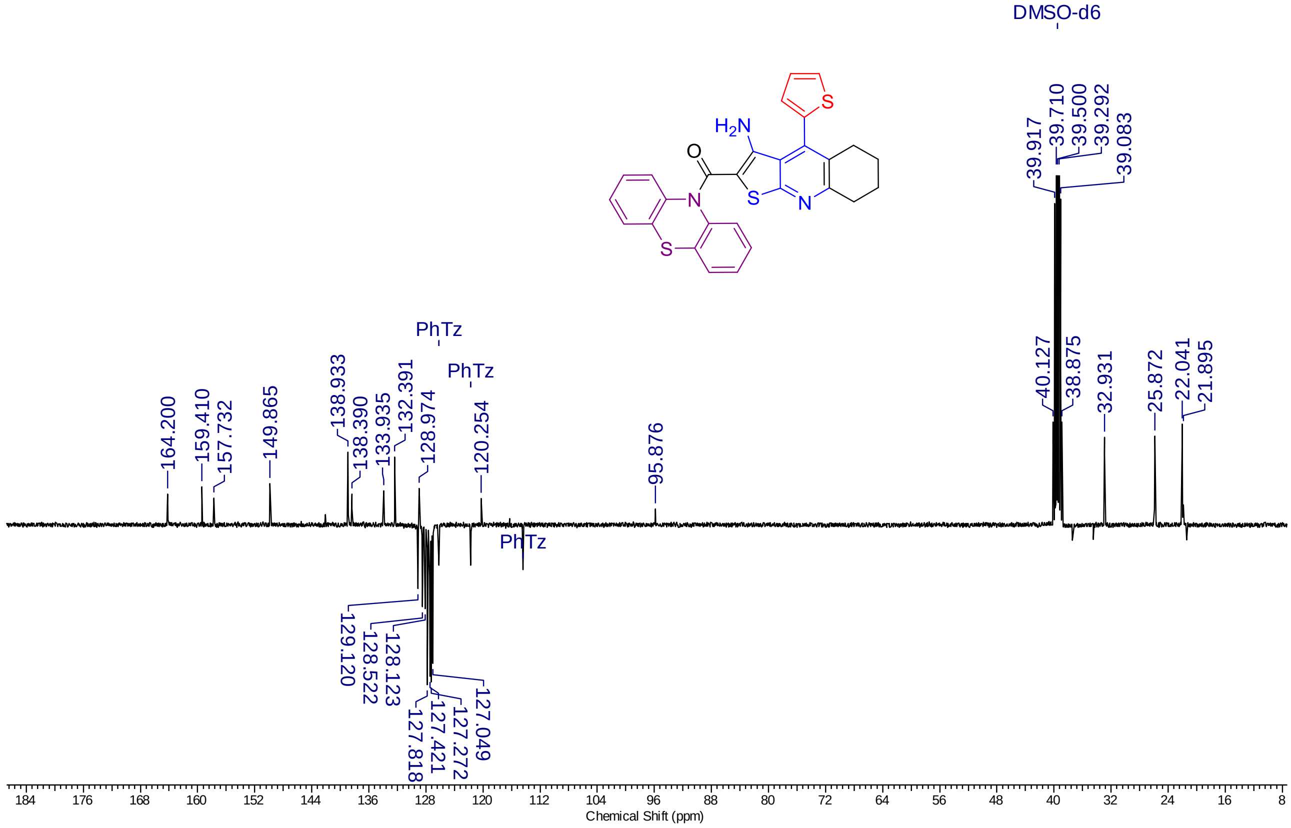

Figure S53. FTIR spectrum of (3-amino-4,6-dimethylthieno[2,3-b]pyridin-2-yl)(10H-phenothiazin-10-yl)methanone 12i.

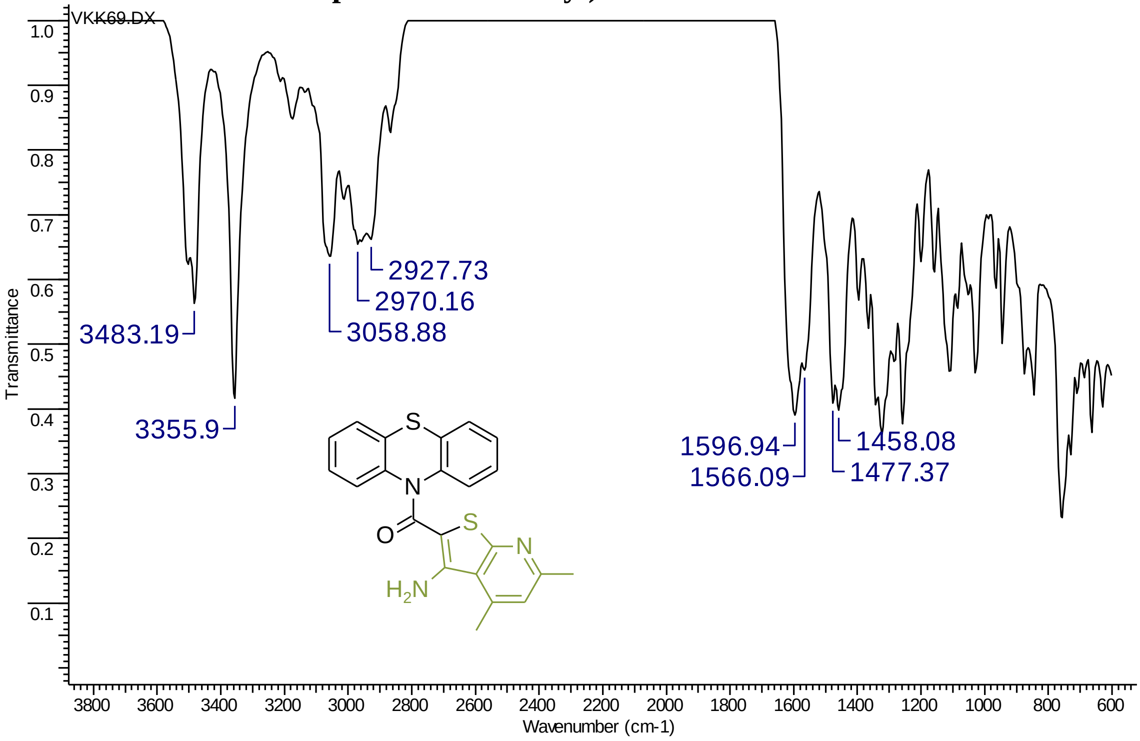

Figure S54. ^{1}H NMR spectrum (400 MHz, DMSO-d_6) of (3-amino-4,6-dimethylthieno[2,3-b]pyridin-2-yl)(10H-phenothiazin-10-yl)methanone 12i

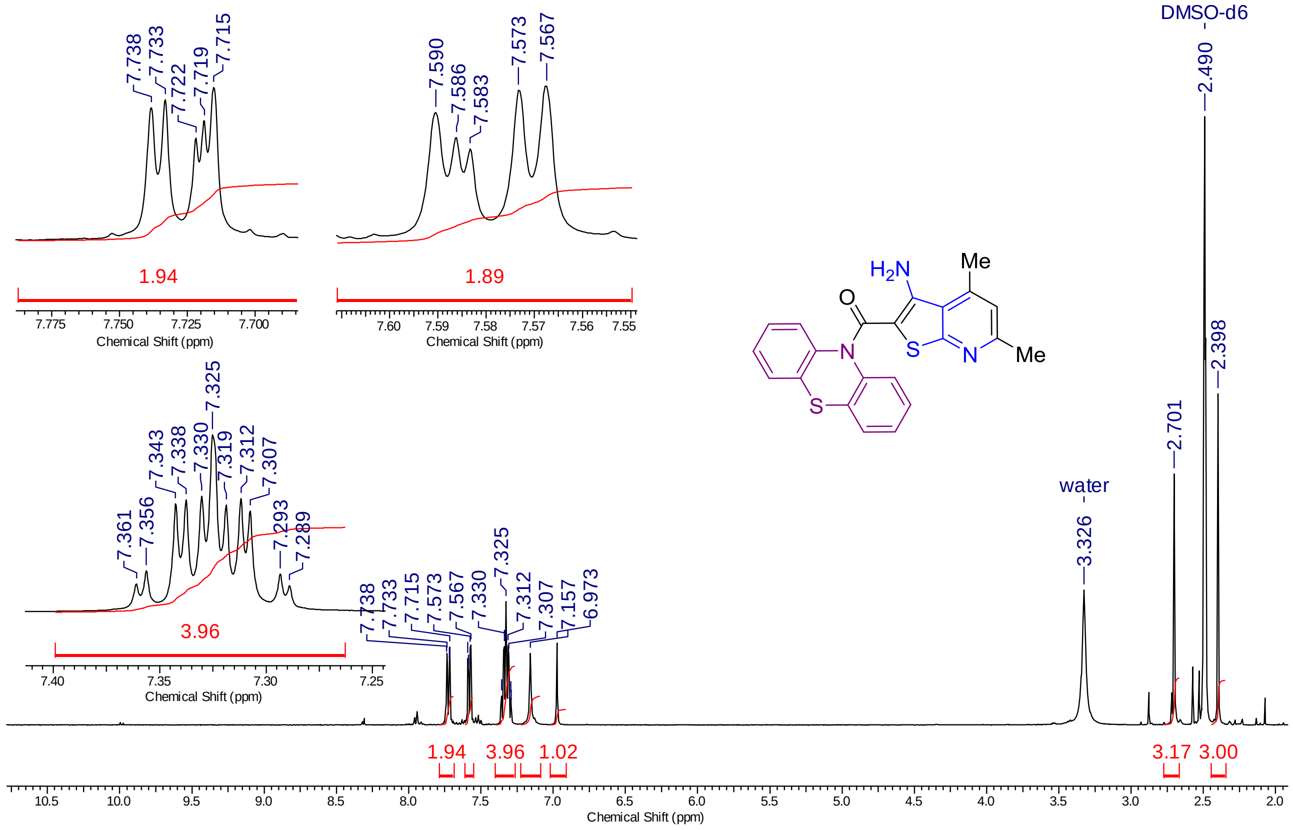

Figure S55. ^{13}C DEPTQ NMR spectrum (101 MHz, DMSO-d_6) of (3-amino-4,6-dimethylthieno[2,3-b]pyridin-2-yl)(10H-phenothiazin-10-yl)methanone 12i

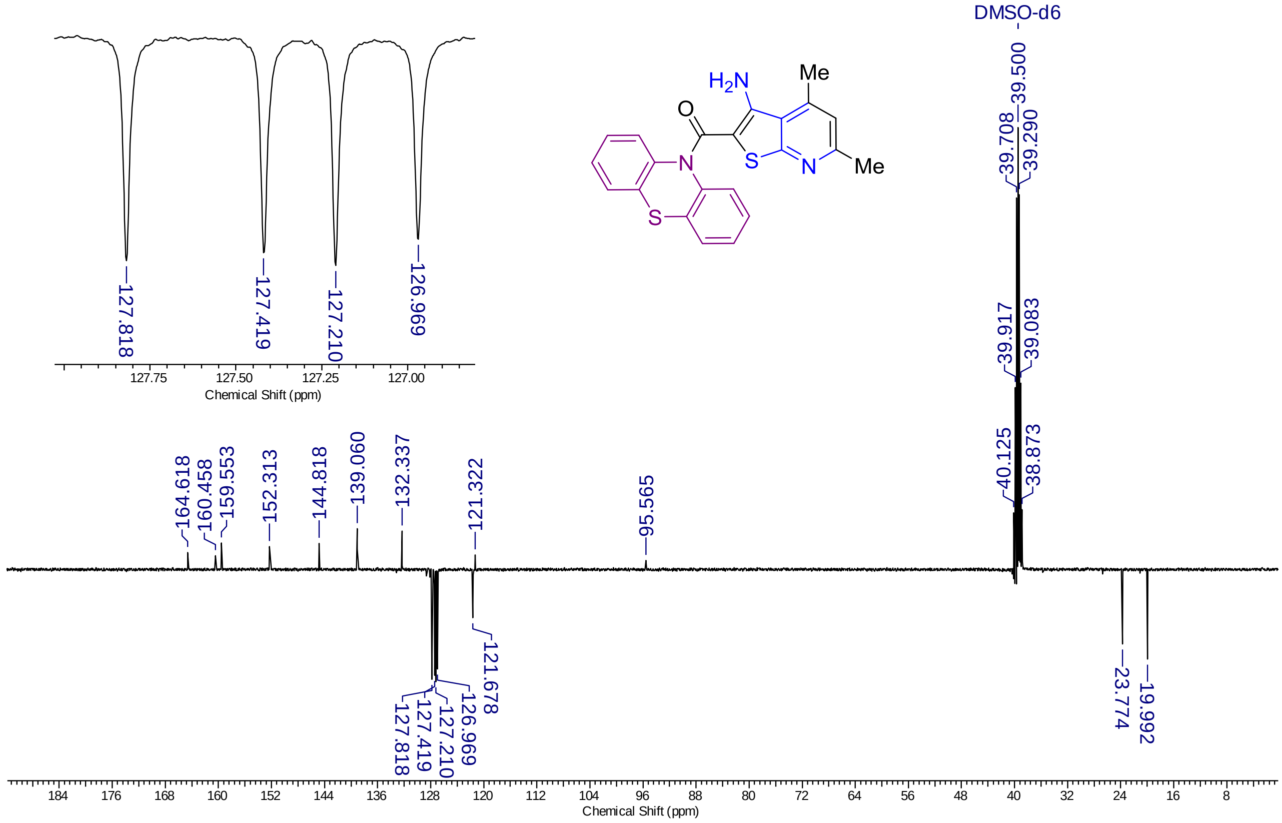

Figure S56. FTIR spectrum (nujol) of (3-amino-6-methylthieno[2,3-b]pyridin-2-yl)(10H-phenothiazin-10-yl)methanone 12k

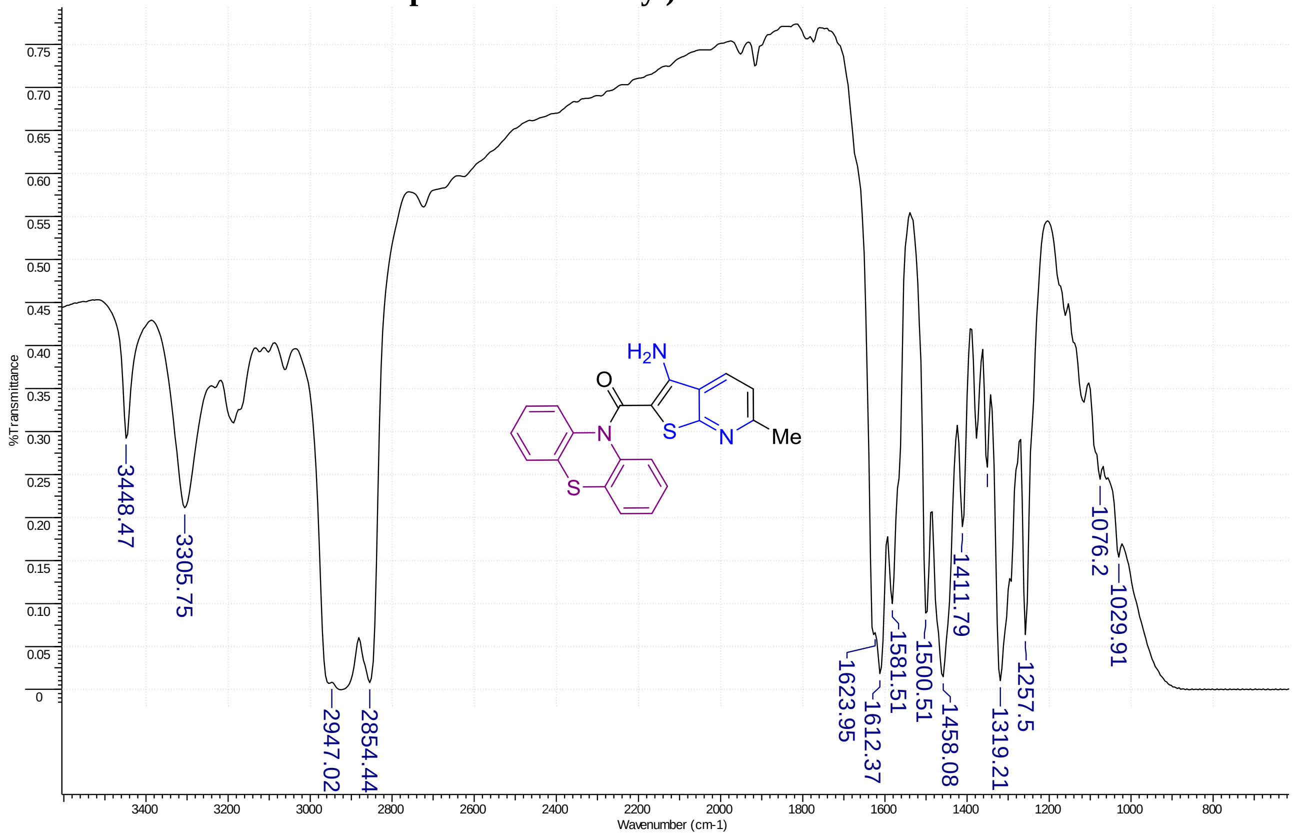

Figure S57. ^{1}H NMR spectrum (400 MHz, DMSO-d_6) of (3-amino-6-methylthieno[2,3-b]pyridin-2-yl)(10H-phenothiazin-10-yl)methanone 12k

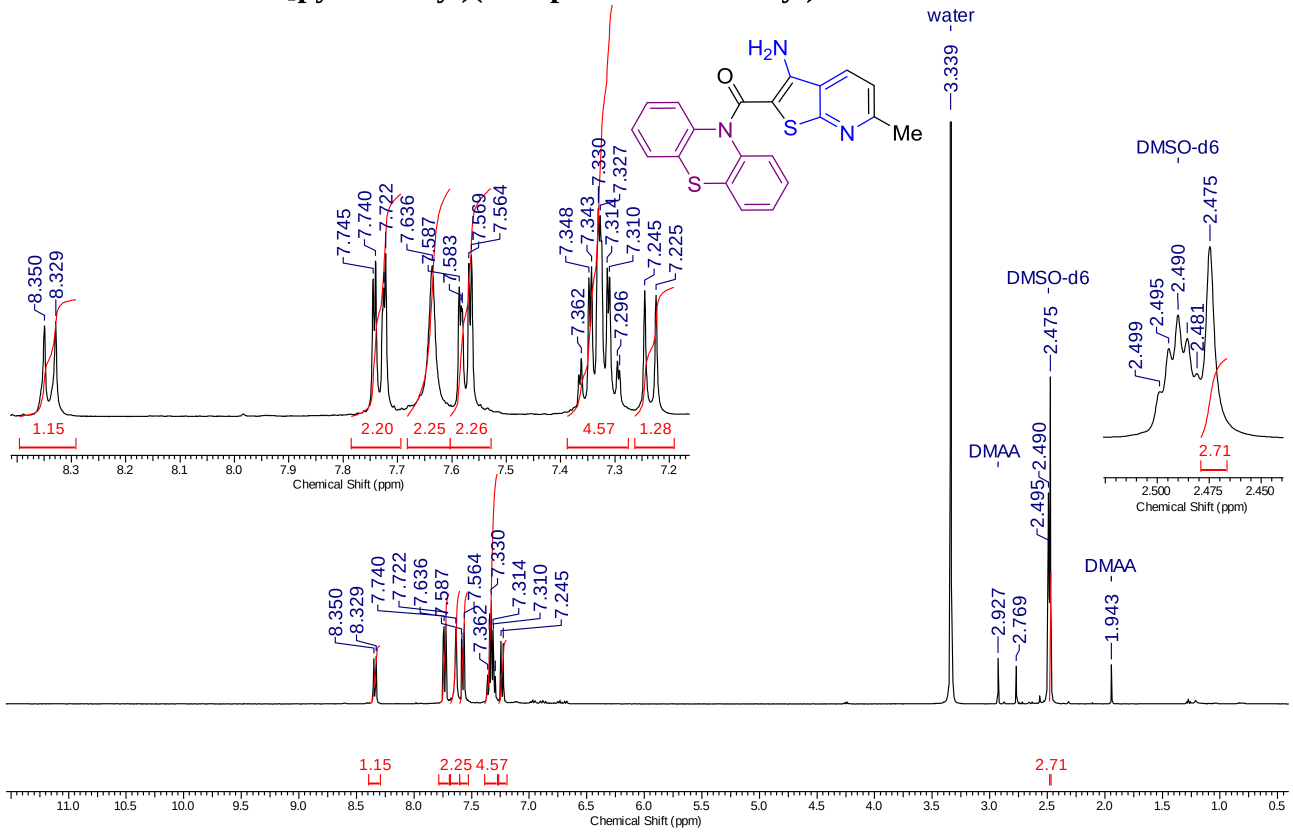

Figure S58. ^{13}C DEPTQ NMR spectrum (101 MHz, DMSO-d_6) of (3-amino-6-methylthieno[2,3-b]pyridin-2-yl)(10H-phenothiazin-10-yl)methanone 12k

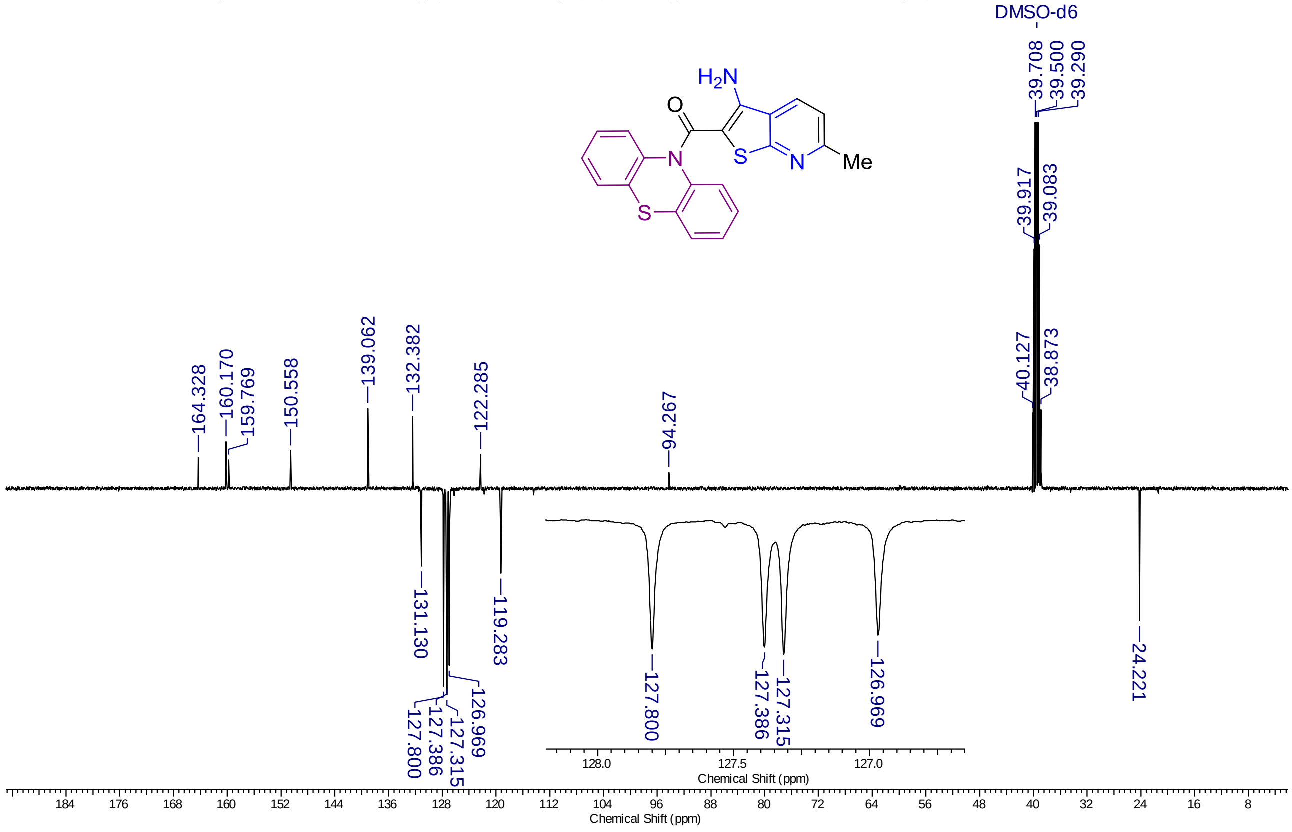

Figure S59. FTIR spectrum (nujol) of (3-amino-6-methylthieno[2,3-b]pyridin-2-yl)(10H-phenothiazin-10-yl)methanone 12m

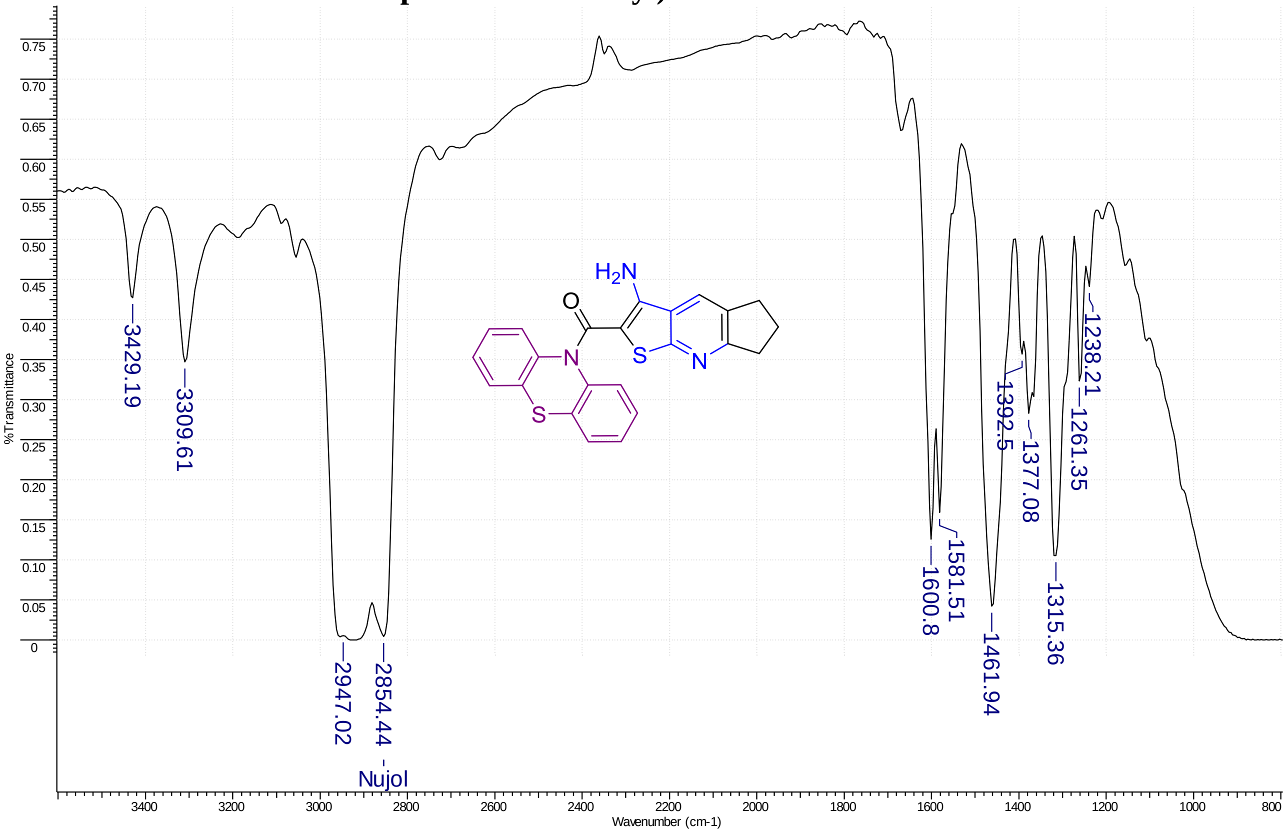

Figure S60. ^{1}H NMR spectrum (400 MHz, DMSO-d_6) of (3-amino-6-methylthieno[2,3-b]pyridin-2-yl)(10H-phenothiazin-10-yl)methanone 12m

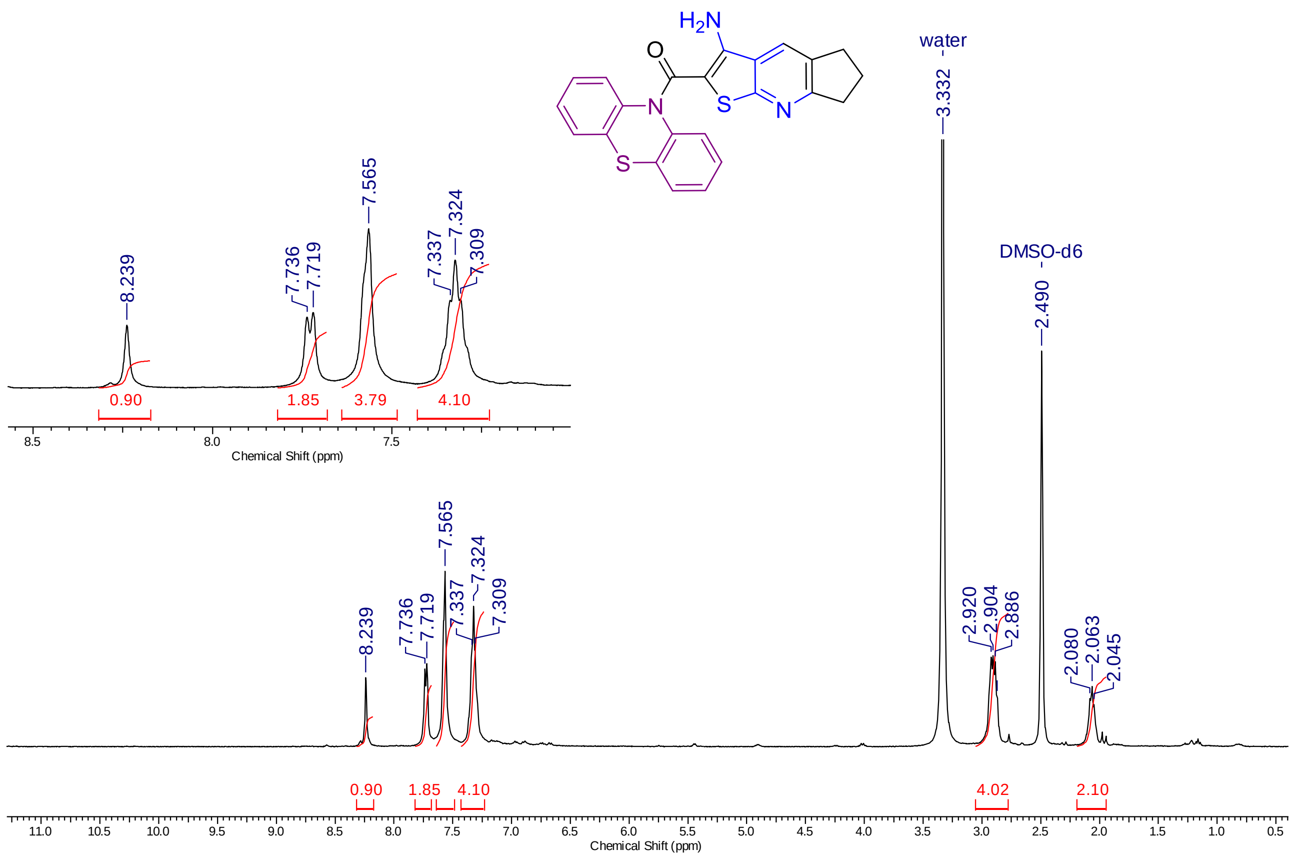

Figure S61. ^{13}C DEPTQ NMR spectrum (101 MHz, DMSO-d_6) of (3-amino-6-methylthieno[2,3-b]pyridin-2-yl)(10H-phenothiazin-10-yl)methanone 12m

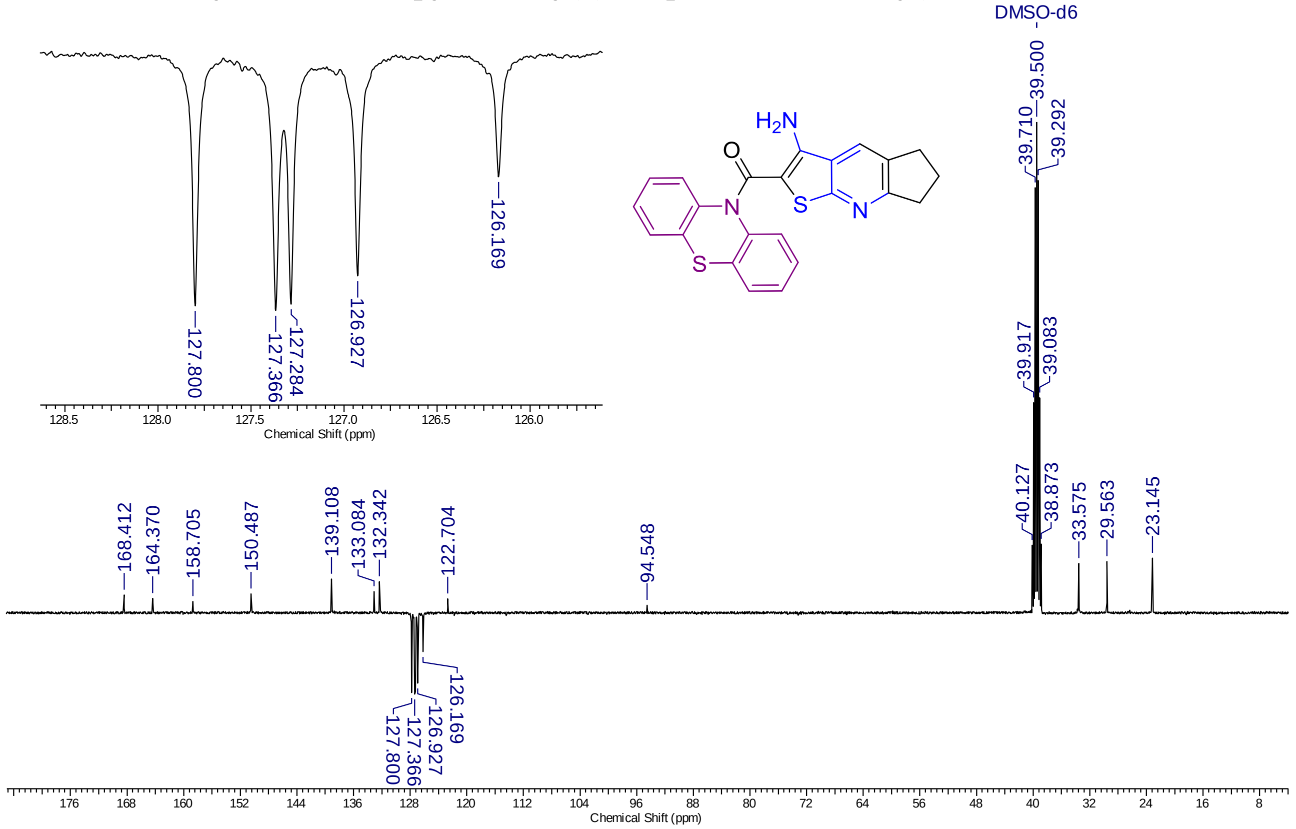

Figure S62. FTIR spectrum (nujol) of (3-amino-4,6-dimethylthieno[2,3-b]pyridin-2-yl)(3,7-dibromo-10H-phenothiazin-10-yl)methanone 12n

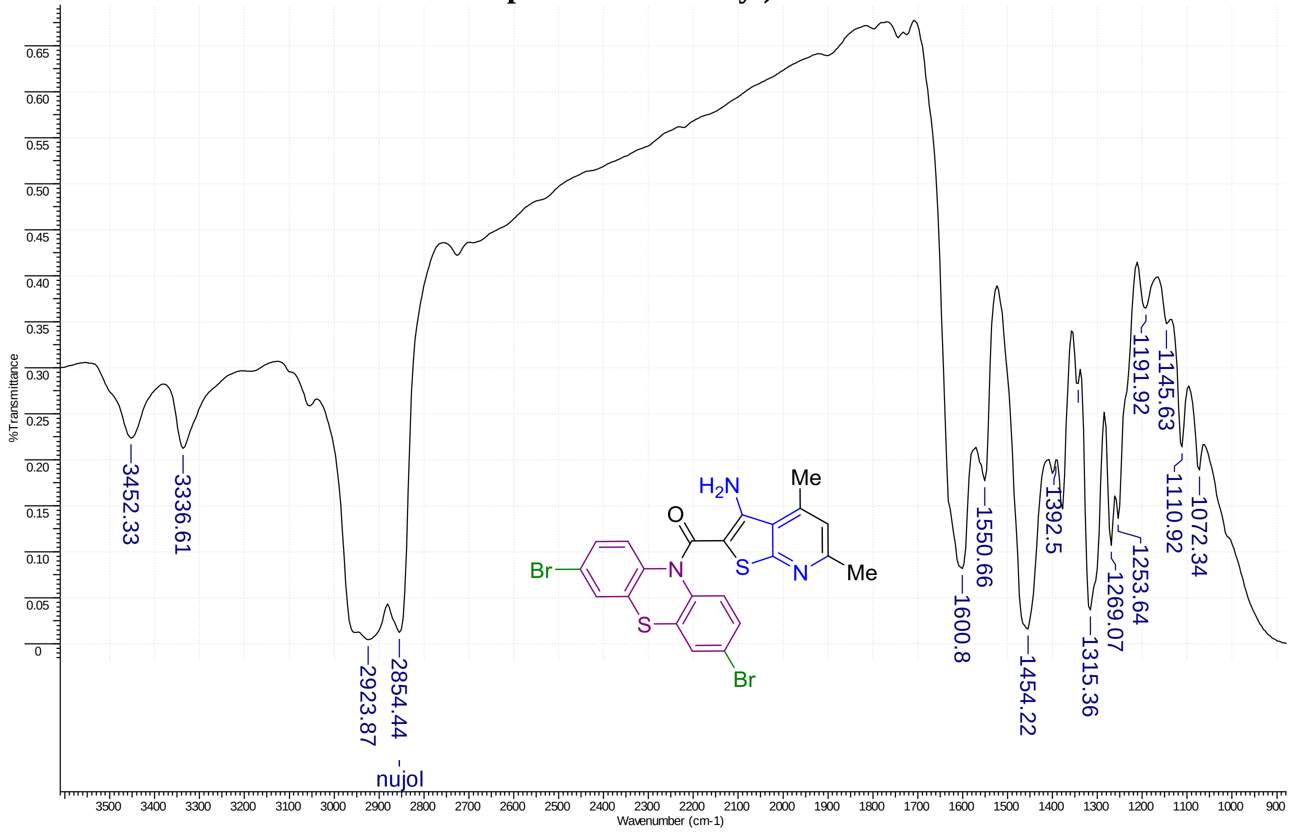

Figure S63. ^{1}H NMR spectrum (400 MHz, DMSO-d_6) of (3-amino-4,6-dimethylthieno[2,3-b]pyridin-2-yl)(3,7-dibromo-10H-phenothiazin-10-yl)methanone 12n

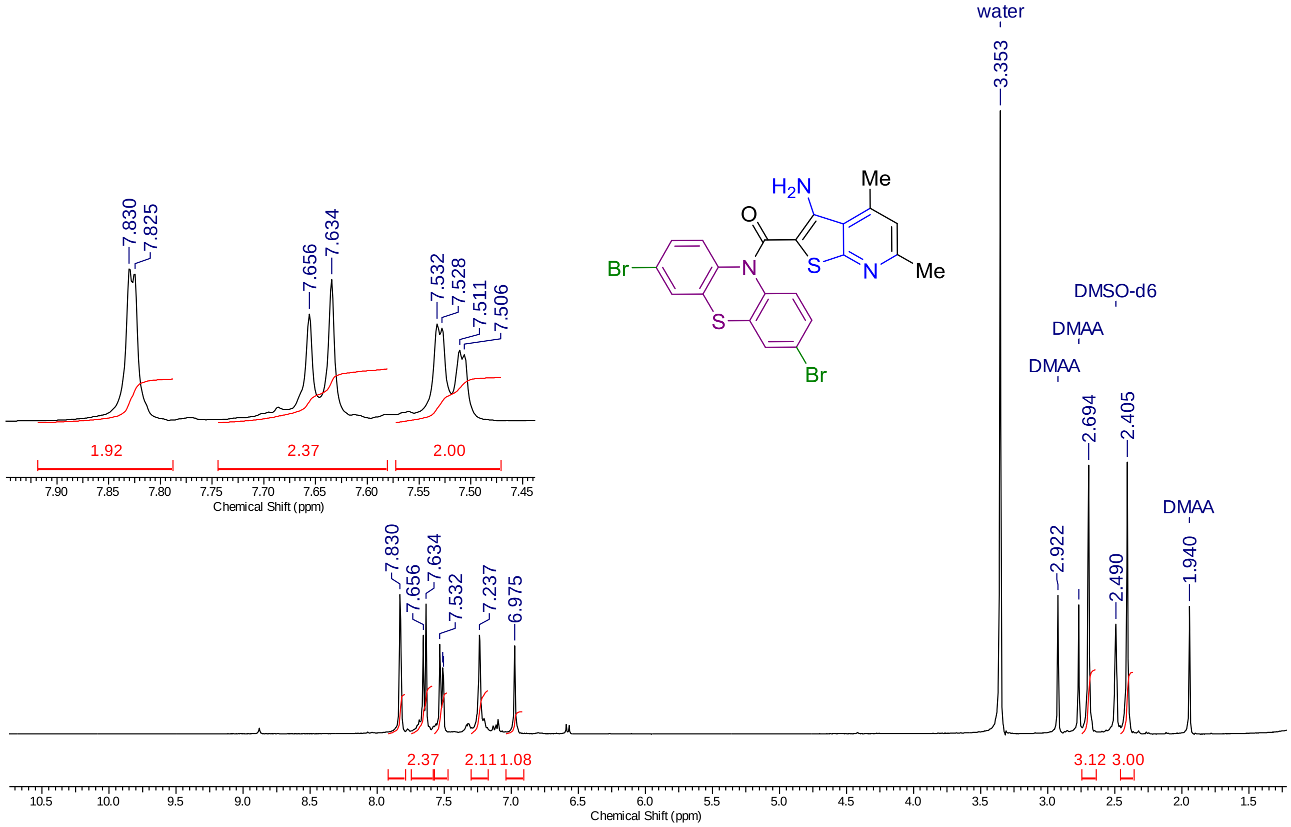

Figure S64. ^{13}**C NMR spectrum (101 MHz, DMSO-d$_6$) of (3-amino-4,6-dimethylthieno[2,3-b]pyridin-2-yl)(3,7-dibromo-10H-phenothiazin-10-yl)methanone 12n**

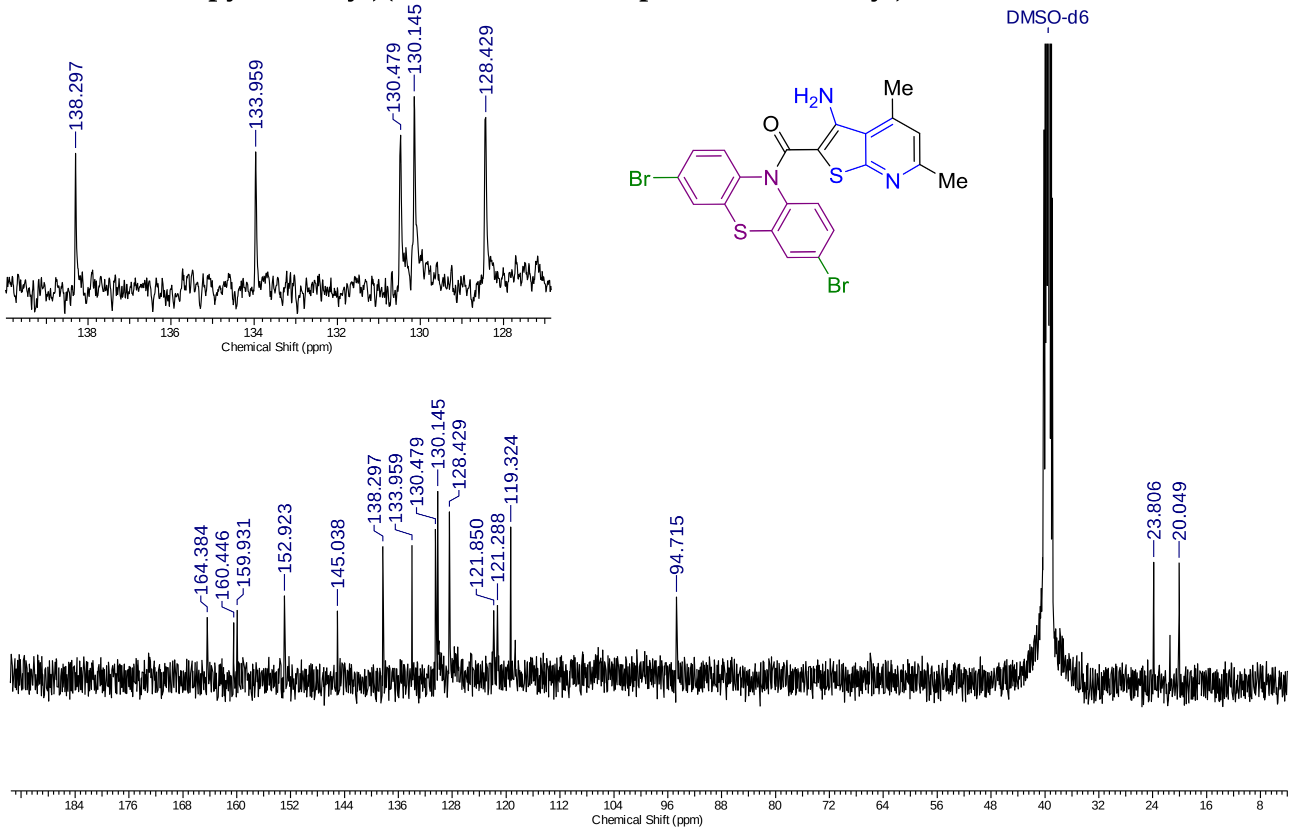

Figure S65. ^{1}**H NMR spectrum (400 MHz, DMSO-d$_6$) of 2-chloro-N-[4,6-dimethyl-2-(10H-phenothiazine-10-carbonyl)thieno[2,3-b]pyridin-3-yl]acetamide 18**

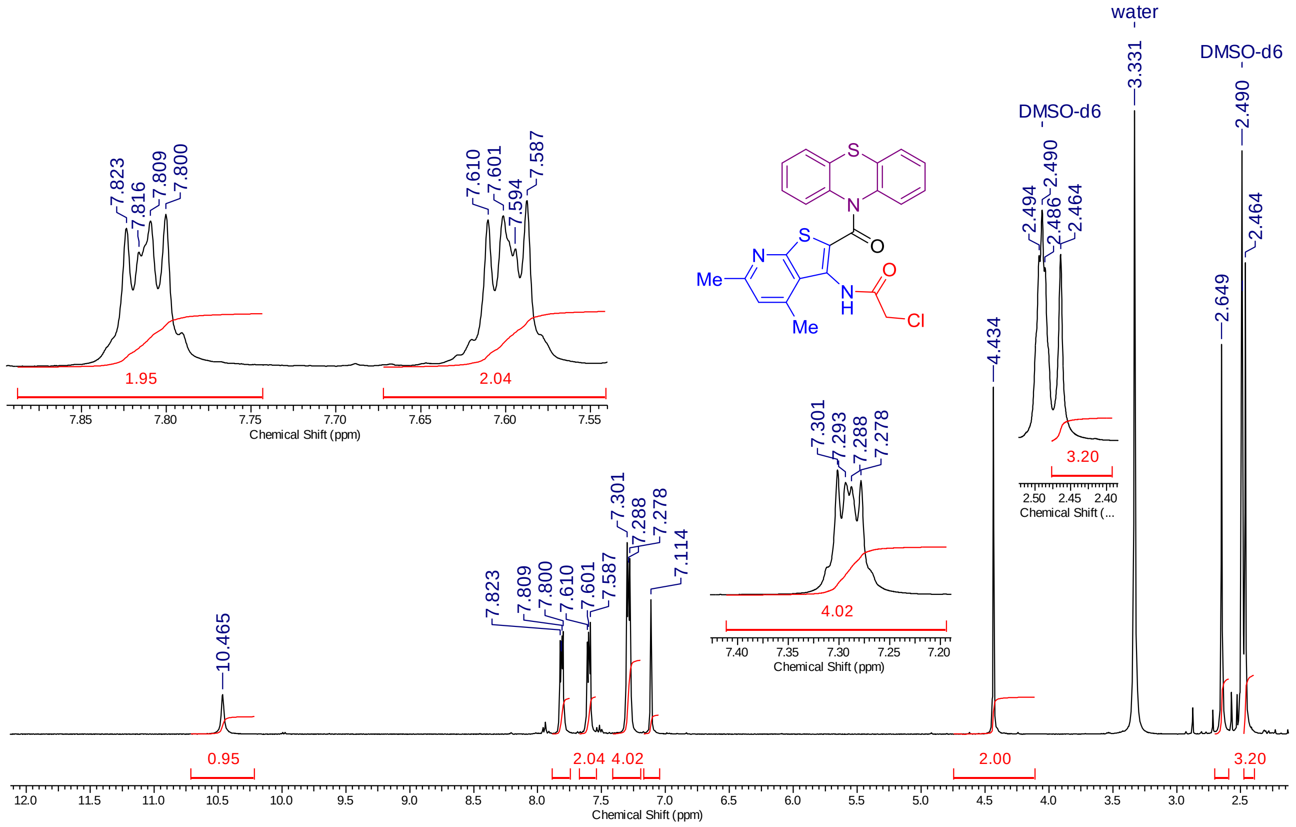

Figure S66. ^{13}C DEPTQ NMR spectrum (101 MHz, DMSO-d_6) of 2-chloro-N-[4,6-dimethyl-2-(10H-phenothiazine-10-carbonyl)thieno[2,3-b]pyridin-3-yl]acetamide 18

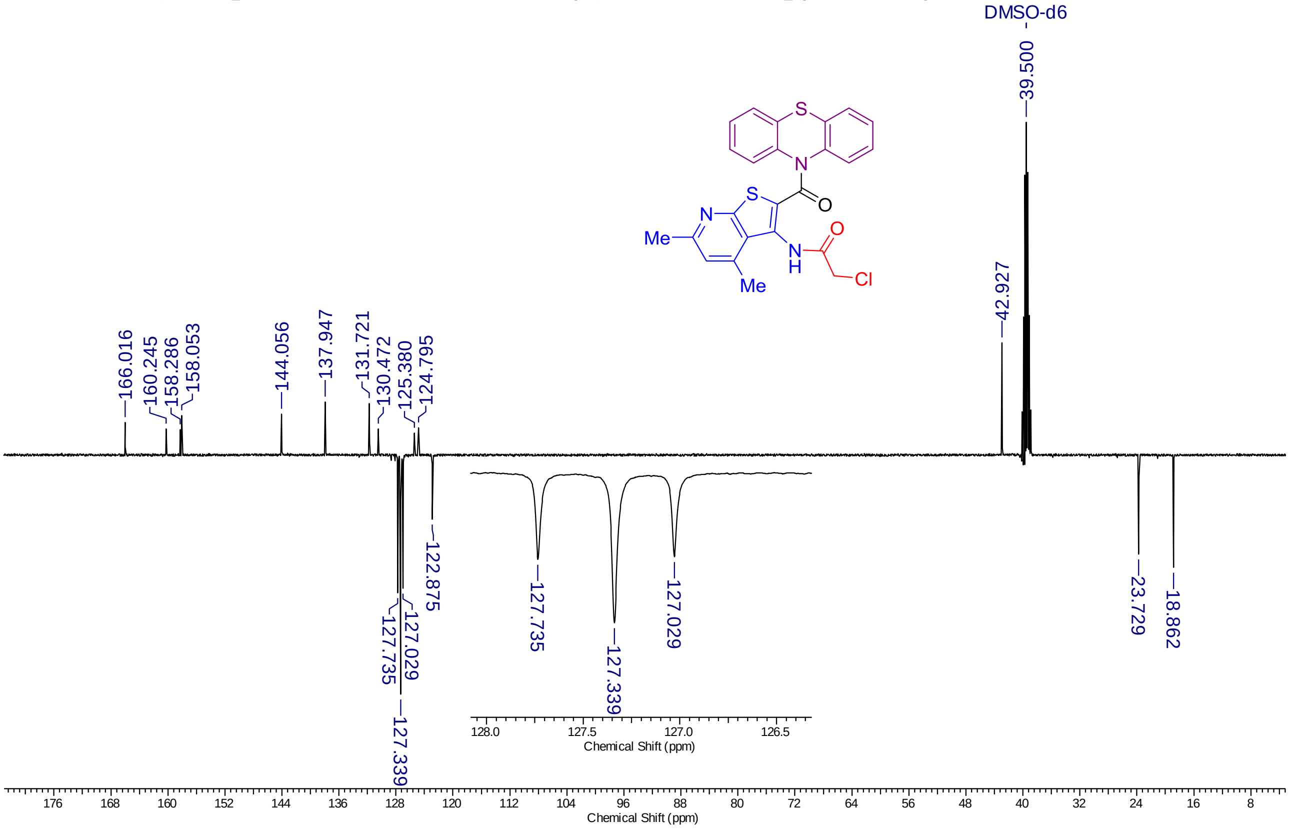

Figure S67. FTIR spectrum (nujol) of 3-{4,6-dimethyl-2-(10H-phenothiazine-10-carbonyl)thieno[2,3-b]pyridin-3-yl}-2-iminothiazolidin-4-one 30

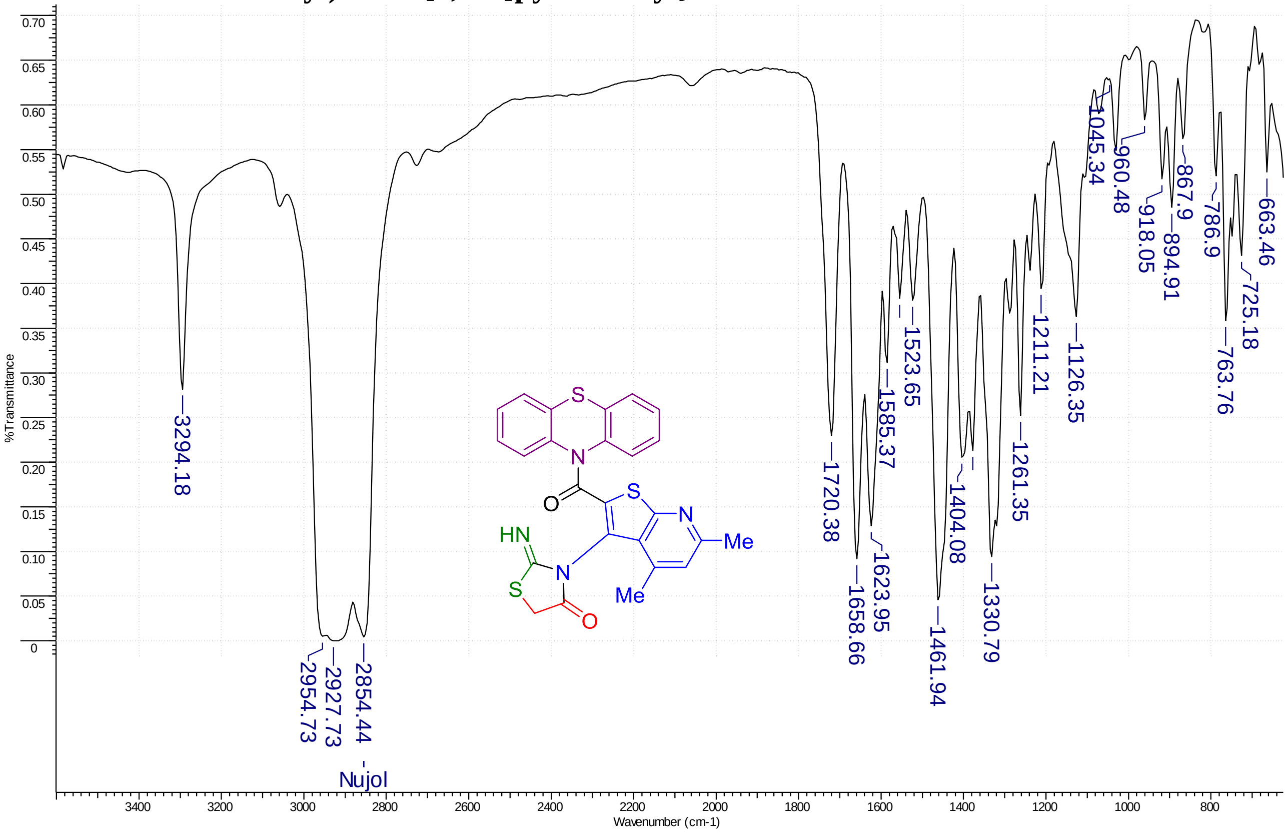

Figure S68. ^{1}H NMR spectrum (400 MHz, DMSO-d_6) of 3-{4,6-dimethyl-2-(10H-phenothiazine-10-carbonyl)thieno[2,3-b]pyridin-3-yl}-2-iminothiazolidin-4-one 30

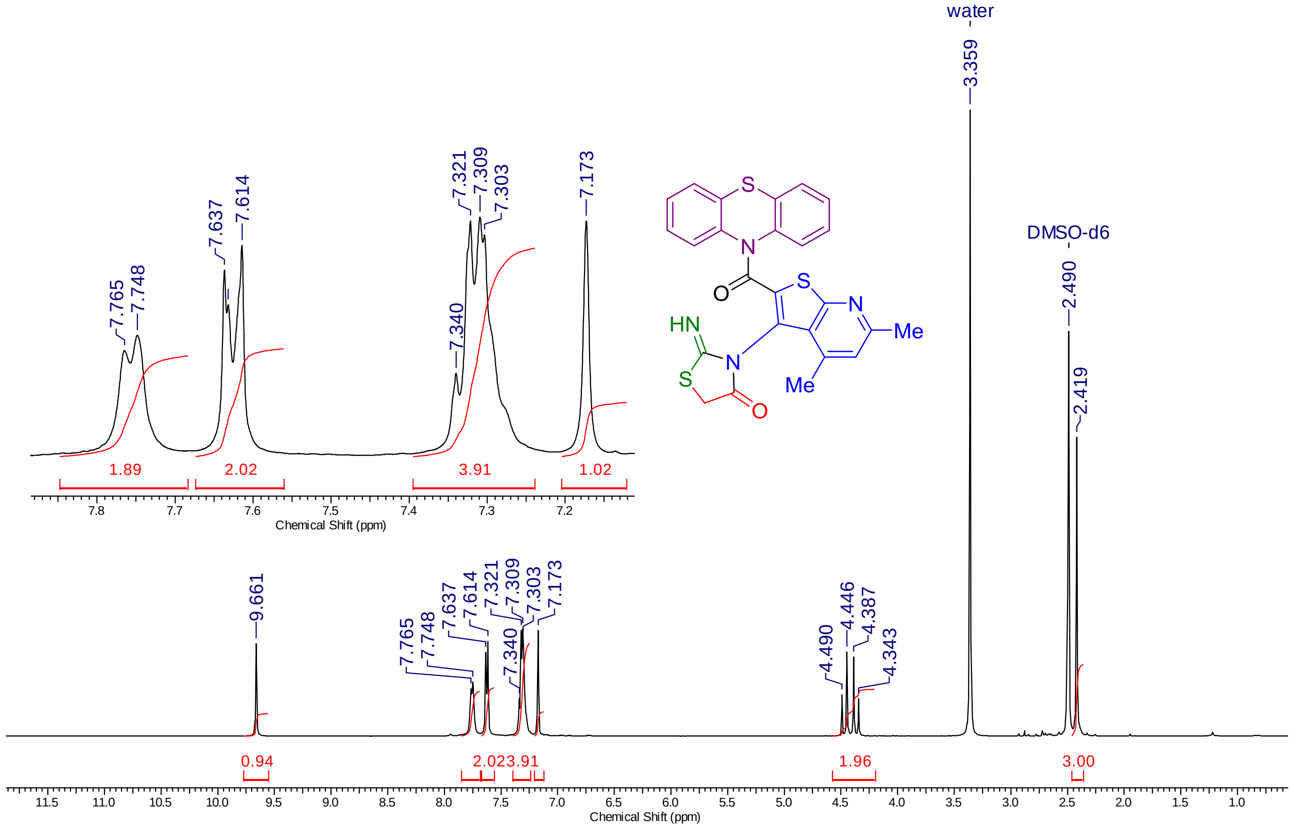

Figure S69. ^{13}C DEPTQ NMR spectrum (101 MHz, DMSO-d_6) of 3-{4,6-dimethyl-2-(10H-phenothiazine-10-carbonyl)thieno[2,3-b]pyridin-3-yl}-2-iminothiazolidin-4-one 30

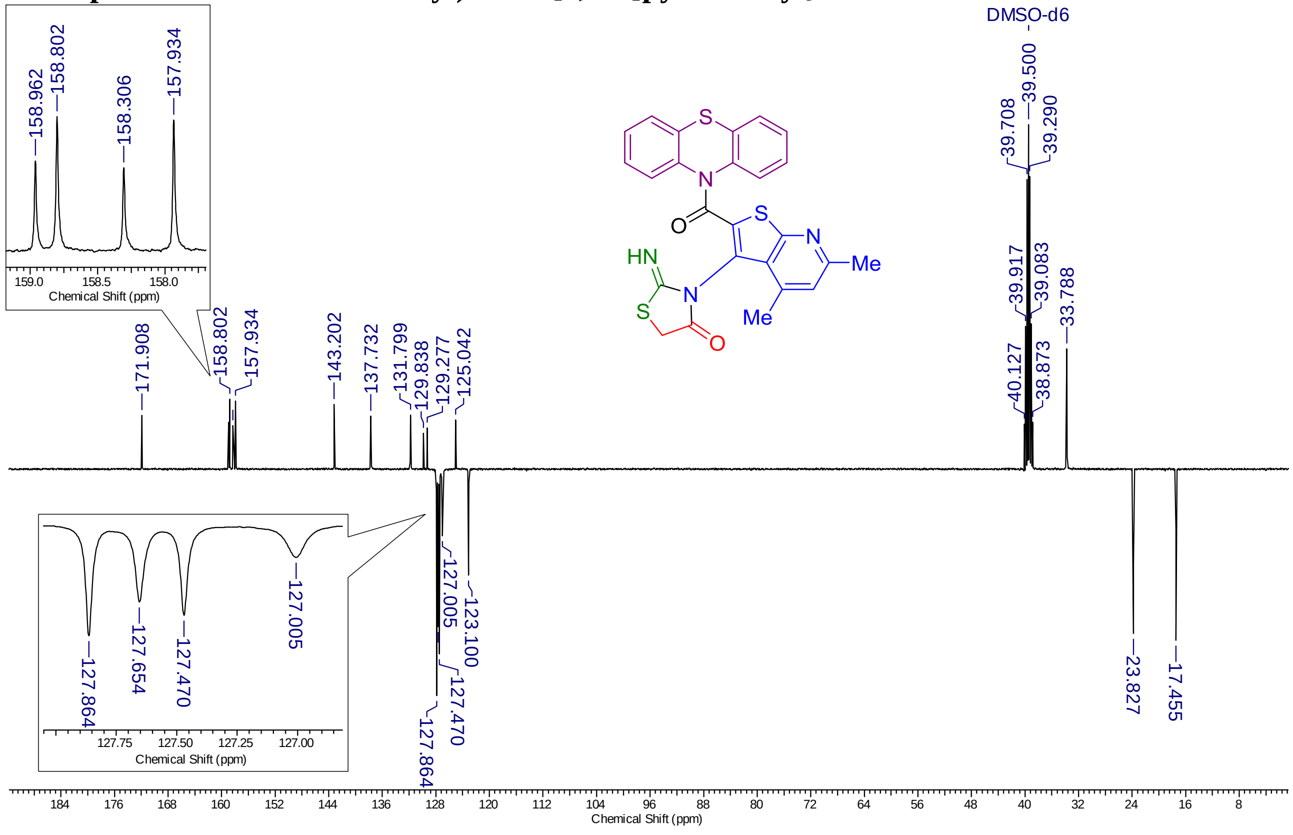

Figure S70. Comparison of the IR spectra of compound 30: experimental (KBr pellets) (top) and the IR spectrum calculated at the B3LYP-D4/def2-TZVP level (bottom).

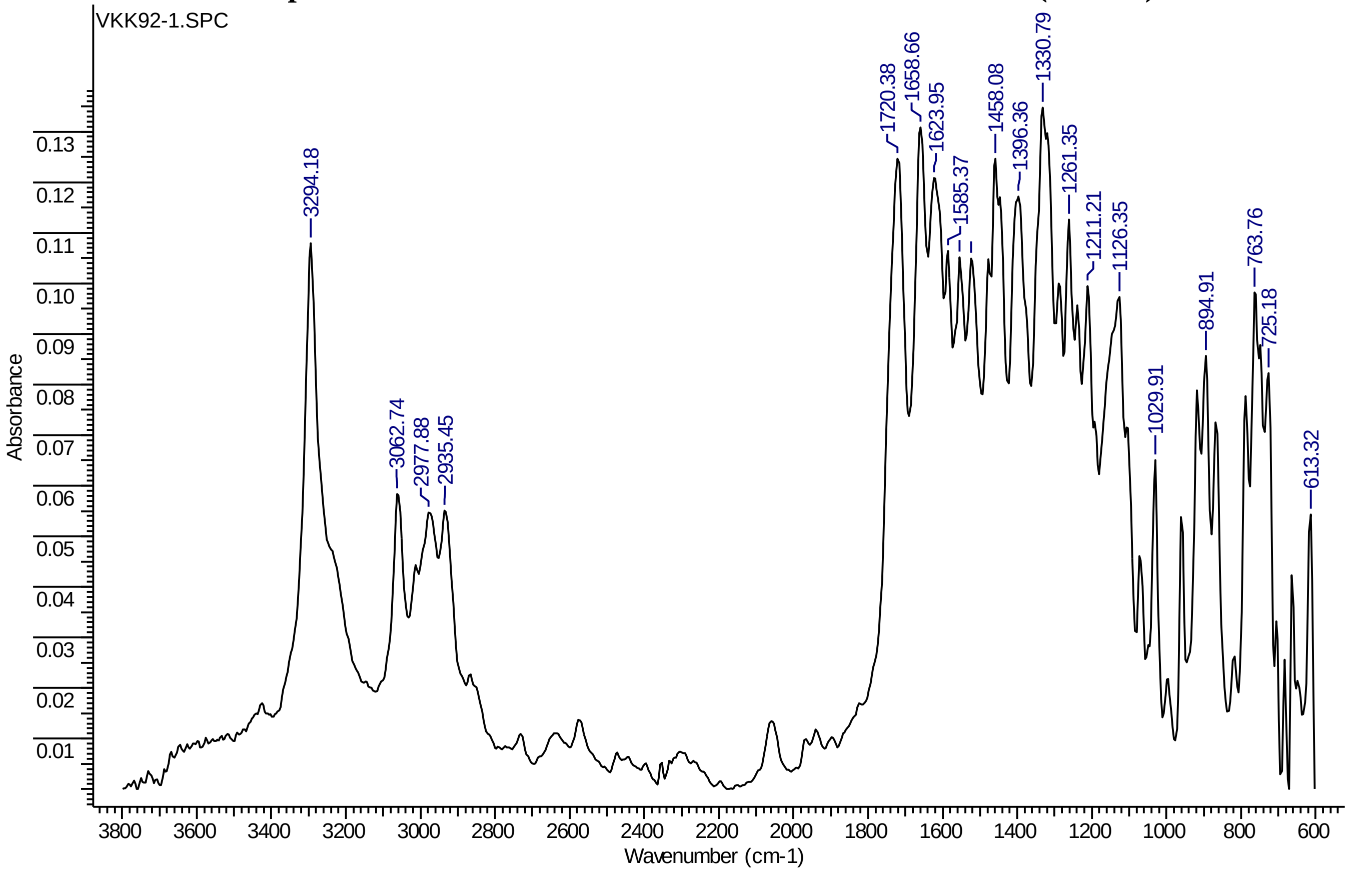

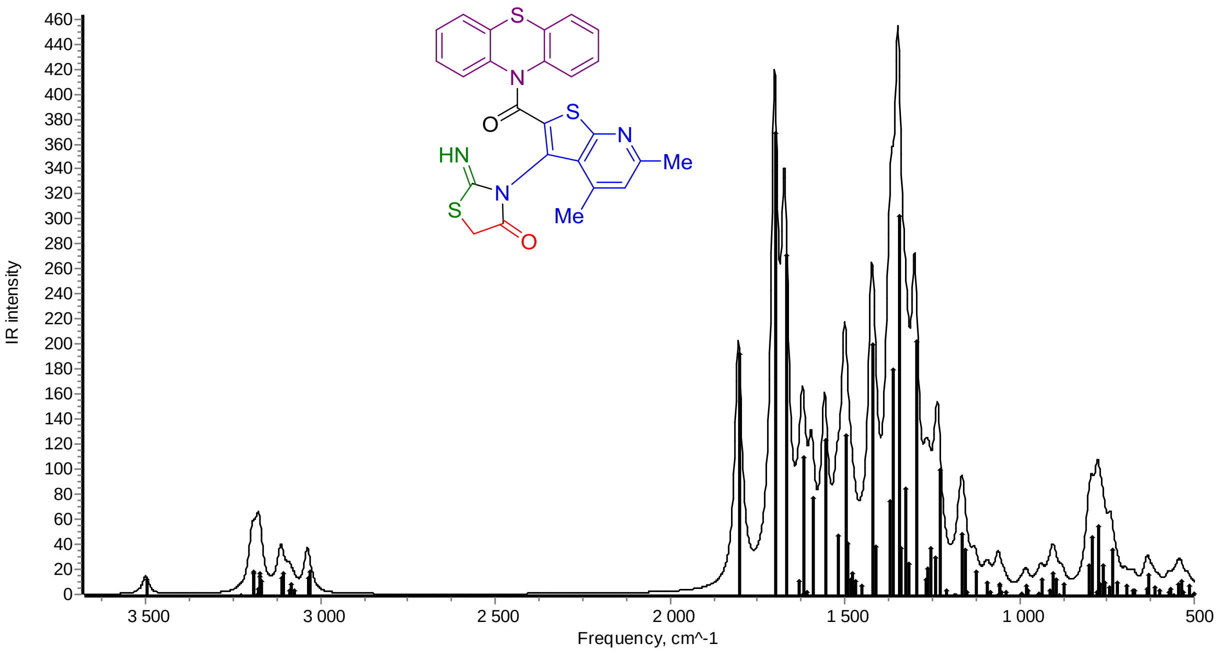

Table S8. Toxicity risks and physicochemical parameters of molecules 11a-h, 12a-n, 18 and 30 predicted using OSIRIS Property Explorer

Molecule	Toxicity risks*				Physico-chemical parameters					
	**A**	**B**	**C**	**D**	*cLogP*	*logS*	MW	TPSA	Drug likeness	Drug Score
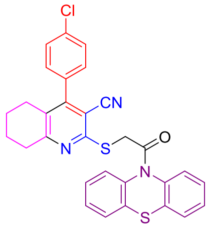 **11a**	-	-	-	+	7.97	-10.04	540	107.6	-4.67	0.11
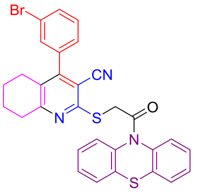 **11b**	-	-	-	+	8.09	-10.14	585	107.6	-8.77	0.10
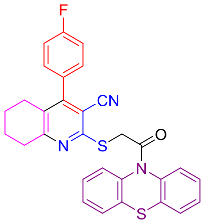 **11c**	-	-	-	+	7.46	-9.62	524	107.6	-6.37	0.12
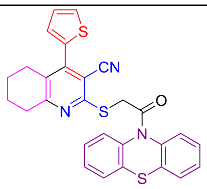 **11d**	-	-	-	+	7.39	-9.23	512	135.8	-4.13	0.12
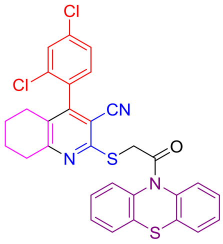 **11e**	-	-	-	+	8.57	-10.78	575	107.6	-4.47	0.10
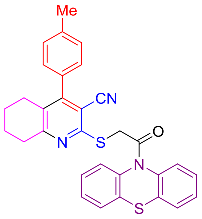 **11f**	-	-	-	+	7.71	-9.65	520	107.6	-6.94	0.11

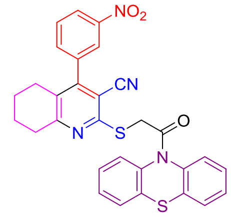 **11g**	-	-	-	+	6.44	-9.77	551	153.4	-10.74	0.12
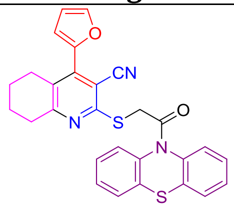 **11h**	-	-	-	+	6.59	-8.66	496	120.7	-5.61	0.13
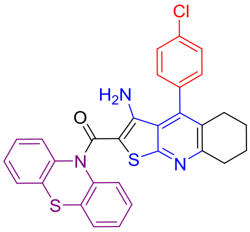 **12a**	-	-	-	+	8.26	-11.12	540	112.8	0.36	0.17
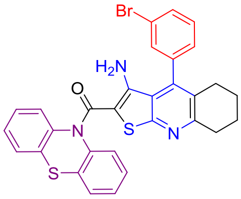 **12b**	-	-	-	+	8.38	-11.22	585	112.8	-3.67	0.10
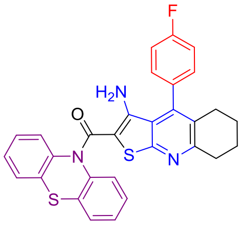 **12c**	-	-	-	+	7.76	-10.70	524	112.8	-1.31	0.14
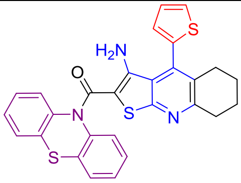 **12d**	-	-	-	+	7.68	-10.30	512	141.0	0.16	0.18
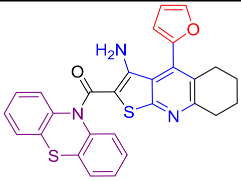 **12h**	-	-	-	+	6.88	-9.73	496	125.9	-0.64	0.17
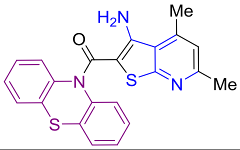	-	-	-	+	5.53	-7.89	404	112.8	4.81	0.36

**12i**										
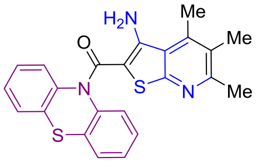 **12j**	-	-	-	+	5.87	-8.24	418	112.8	4.81	0.33
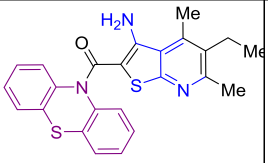 **12k**	-	-	-	+	6.29	-8.40	432	112.8	4.62	0.31
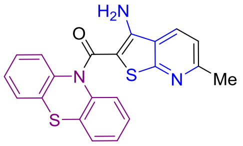 **12l**	-	-	-	+	5.18	-7.55	390	112.8	4.81	0.39
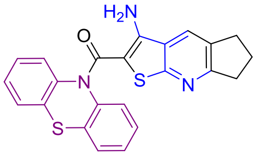 **12m**	-	-	-	+	5.66	-8.03	416	112.8	1.85	0.33
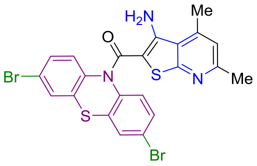 **12n**	-	-	-	+	6.98	-9.56	561	112.8	1.37	0.20
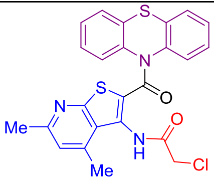 **18**	+	+	-	+	6.14	-8.56	480	115.8	5.77	0.10
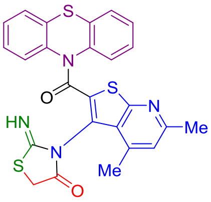 **30**	-	-	-	+	5.27	-8.18	503	156.2	5.47	0.32

*A high risk of toxicity is indicated by a plus sign (+), and no toxicity is indicated by a minus sign (–). A – Mutagenicity, B – Tumorogenicity, C – Irritant effects, D – Reproductive effects.

Table S9. Predicted ADMET parameters for molecules 11a-h, 12a-n, 18 and 30

No	BBB permeability*	Gastrointestinal absorption (HIA)*	Cytochrome P450 inhibition*					Ames test*	Acute toxicity (rats) pLD_{50}, lg [1/(mol/kg)]
			CYP1A2	CYP2C19	CYP2C9	CYP2D6	CYP3A4		
**11a**	+ 0.9689	+ 0.8975	+ 0.7873	+ 0.9252	+ 0.8836	– 0.8922	– 0.6254	– 0.7152	2.4093
**11b**	+ 0.9638	+ 0.8576	+ 0.7941	+ 0.8897	+ 0.8962	– 0.8851	+ 0.6800	– 0.7173	2.4990
**11c**	+ 0.9746	+ 0.8968	+ 0.7724	+ 0.8747	+ 0.8723	– 0.9188	– 0.6004	– 0.7185	2.5471
**11d**	+ 0.9753	+ 0.9412	+ 0.7953	+ 0.8781	+ 0.8989	– 0.9525	– 0.7260	– 0.7521	2.4281
**11e**	+ 0.9689	+ 0.8975	+ 0.7873	+ 0.9252	+ 0.8836	– 0.8922	– 0.6683	– 0.7152	2.4093
**11f**	+ 0.9695	+ 0.9265	+ 0.8232	+ 0.8733	+ 0.9284	– 0.9414	– 0.7518	– 0.7476	2.5174
**11g**	+ 0.9376	+ 0.8532	- 0.5099	+ 0.7573	+ 0.6618	– 0.8729	+ 0.9086	+ 0.6984	2.6291
**11h**	+ 0.9804	+ 0.9689	+ 0.7375	+ 0.8594	+ 0.9026	– 0.9571	+ 0.6825	- 0.7278	2.3981
**12a**	+ 0.9597	+ 0.9891	+ 0.7297	+ 0.9248	+ 0.7951	– 0.8593	+ 0.5000	- 0.6070	2.3681
**12b**	+ 0.9545	+ 0.9842	+ 0.7977	+ 0.8793	+ 0.8073	– 0.8579	+ 0.5152	- 0.5989	2.3972
**12c**	+ 0.9655	+ 0.9890	+ 0.7284	+ 0.8571	+ 0.7718	– 0.8950	+ 0.5149	- 0.5808	2.4479
**12d**	+ 0.9716	+ 0.9867	+ 0.7048	+ 0.9066	+ 0.8113	– 0.8694	+ 0.6644	- 0.5765	2.3078
**12h**	+ 0.9775	+ 0.9931	+ 0.6827	+ 0.8850	+ 0.8361	– 0.8684	+ 0.7348	- 0.5926	2.3063
**12i**	+ 0.9611	+ 0.9662	+ 0.8144	+ 0.9347	+ 0.7212	– 0.8768	- 0.5192	+ 0.6132	2.1684
**12j**	+ 0.9584	+ 0.9629	+ 0.7668	+ 0.9396	+ 0.7916	– 0.8738	+ 0.5585	+ 0.5842	2.2266
**12k**	+ 0.9613	+ 0.9762	+ 0.5688	+ 0.8537	+ 0.8831	– 0.9020	+ 0.5650	- 0.5198	2.3889
**12l**	+ 0.9658	+ 0.9747	+ 0.8840	+ 0.9502	+ 0.7030	– 0.8285	+ 0.5739	+ 0.6505	2.1164
**12m**	+ 0.9707	+ 0.9475	+ 0.8396	+ 0.8485	+ 0.7157	– 0.8238	- 0.5152	- 0.5353	2.2746
**12n**	+ 0.9409	+ 0.9594	+ 0.8902	+ 0.9099	+ 0.7809	– 0.8008	+ 0.5223	+ 0.5362	2.2684
**18**	+ 0.9531	+ 0.9863	- 0.5117	+ 0.8803	+ 0.8435	– 0.9016	+ 0.7610	- 0.6118	2.2685
**30**	+ 0.9351	+ 0.9876	+ 0.5108	+ 0.6440	+ 0.6877	– 0.9326	- 0.6942	- 0.5827	2.3065

The sign (+) or (–) indicates the presence or absence of an effect, and the number represents the probability of the effect.

Table S10. Prediction of the protein-ligand interaction for molecules 11a-h, 12a-n, 18, and 30 using the GalaxyWeb Sagittarius molecular docking protocol

Molecule	PDB ID	UniProt ID	Predock score	Docking score ΔG_{bind}, kcal/mol
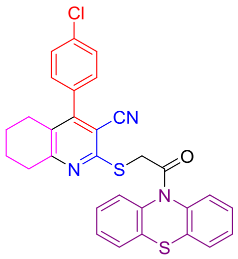 **11a**	**2zno**	P37231	0.124	-30.804
	**8hum**	P37231-2	0.090	-32.217
	7qp4	P51449	0.094	-31.405
	2o2n	Q07817	0.107	-27.448
	5oc8	Q00987	0.118	-25.433
	6p9f	Q15788	0.079	-30.321
	5vb7	Q15596	0.078	-30.260
	6l38	Q07869	0.072	-30.416
	4jv6	O43924	0.063	-31.092
	7wgl	Q03181	0.061	-30.773
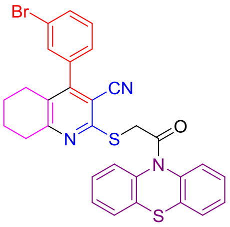 **11b**	**2zno**	P37231	0.124	-30.835
	2o2n	Q07817	0.119	-28.568
	7qp4	P51449	0.090	-31.922
	**8hum**	P37231-2	0.091	-31.167
	2q7m	P20292, P20292	0.060	-33.723
	6p9f	Q15788	0.075	-31.087
	5vb7	Q15596	0.070	-31.648
	4jvb	O43924	0.069	-31.420
	5mke	Q13563, Q13563	0.072	-30.701
	6s41	O75469	0.062	-31.992
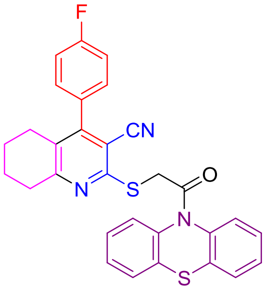 **11c**	**2zno**	P37231	0.125	-29.458
	2o2n	Q07817	0.118	-28.015
	7qp4	P51449	0.097	-30.231
	**8hum**	P37231-2	0.093	-30.464
	5x73	O43924	0.078	-30.445
	6p9f	Q15788	0.082	-29.511
	6a6p	Q03181	0.064	-31.846
	5vb5	Q15596	0.078	-29.588
	6s41	O75469	0.065	-31.246
	5myv	P78362	0.063	-31.134
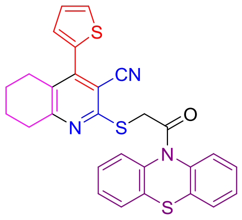 **11d**	**3zln**	Q07817	0.122	-25.818
	**2zno**	P37231	0.092	-29.378
	**8hum**	P37231-2	0.075	-30.139
	7qp4	P51449	0.067	-29.150
	3znr	Q8WUI4	0.074	-27.888
	6vxj	Q9UNQ0, Q9UNQ0	0.063	-28.621
	6k4j	P21926	0.065	-27.596
	5pay	P08709	0.078	-25.855
	1gzp	P29016	0.065	-27.509
	7w9l	Q15858	0.067	-27.270
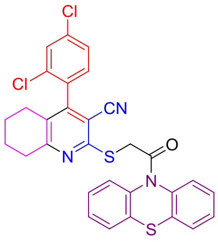 **11e**	5lay	Q00987	0.153	-25.664
	**2zno**	P37231	0.107	-31.447
	2o2n	Q07817	0.105	-28.865
	7qp4	P51449	0.081	-31.961
	**8hum**	P37231-2	0.073	-30.539
	7drt	P56704, Q5T9L3	0.067	-31.331
	3u15	O15151, O15151	0.069	-30.299
	4iph	P27694	0.135	-21.059

	5iez	Q07820, Q07820	0.080	-28.060
	7we4	Q9Y5Y9	0.074	-28.516
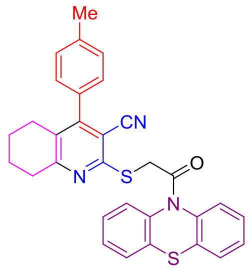 **11f**	2zno	P37231	0.134	-31.292
	8hup	P37231-2	0.105	-33.302
	7qp4	P51449	0.098	-31.471
	4jv6	O43924	0.080	-32.037
	2q7m	P20292, P20292	0.061	-34.170
	6a6p	Q03181	0.067	-33.217
	5vb5	Q15596	0.082	-31.056
	6p9f	Q15788	0.084	-30.486
	6uvc	Q07817	0.130	-24.251
	7wld	Q969N2, Q96S52, Q9H490	0.067	-32.475
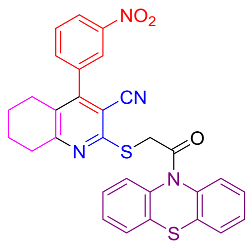 **11g**	6uvc	Q07817	0.185	-24.544
	2zno	P37231	0.097	-30.759
	8hup	P37231-2	0.070	-32.576
	1rd4	P20701, P20701	0.085	-29.805
	7tj9	Q01118	0.091	-28.741
	2q7r	P20292, P20292	0.071	-31.382
	7wld	Q969N2, Q96S52, Q9H490	0.067	-31.754
	2o2f	P10415	0.102	-27.049
	7dsx	P19634, P19634	0.068	-30.916
	1gzq	P29016	0.070	-30.419
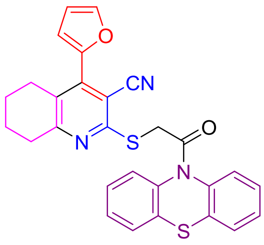 **11h**	2zno	P37231	0.118	-29.345
	3zln	Q07817	0.145	-25.022
	8hup	P37231-2	0.095	-30.703
	7qp4	P51449	0.081	-28.513
	1k7l	Q07869	0.061	-29.954
	5lay	Q00987	0.098	-24.766
	1rd4	P20701, P20701	0.085	-26.399
	6p9f	Q15788	0.069	-28.203
	3znr	Q8WUI4	0.075	-27.383
	7wld	Q969N2, Q96S52, Q9H490	0.056	-29.831
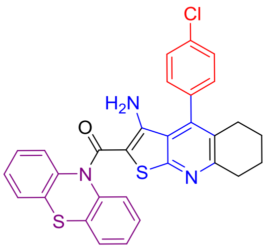 **12a**	2zno	P37231	0.116	-26.278
	7qp4	P51449	0.081	-28.976
	2o2n	Q07817	0.113	-24.529
	8hum	P37231-2	0.092	-26.229
	7wld	Q969N2, Q96S52, Q9H490	0.057	-30.272
	3zns	Q8WUI4	0.087	-26.146
	7jym	Q15788	0.069	-28.391
	1h69	P15559, P15559	0.057	-28.693
	6l38	Q07869	0.064	-27.609
	5oc8	Q00987	0.087	-24.446
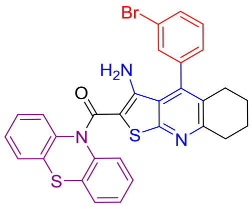 **12b**	2zno	P37231	0.114	-27.245
	2o2n	Q07817	0.124	-25.096
	7qp4	P51449	0.081	-28.829
	8hum	P37231-2	0.089	-27.286
	5oc8	Q00987	0.093	-25.504
	6kzp	O43497	0.071	-27.307
	6l38	Q07869	0.061	-28.370
	6wvg	P19397, P19397	0.059	-28.189
	6vxj	Q9UNQ0, Q9UNQ0	0.073	-26.200
	7nfc	P13010	0.064	-27.335
	2zno	P37231	0.114	-26.995

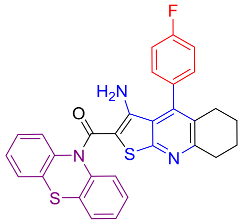 **12c**	7qp4	P51449	0.083	-29.909
	5oc8	Q00987	0.109	-24.487
	**8hum**	P37231-2	0.087	-26.812
	6uvc	Q07817	0.115	-22.307
	4b4o	Q9NRG7	0.064	-27.810
	3zns	Q8WUI4	0.083	-25.184
	1k7l	Q07869	0.066	-26.697
	6wvg	P19397, P19397	0.062	-27.058
	3u15	O15151, O15151	0.065	-26.684
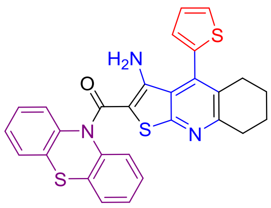 **12d**	8b8x	P37231, Q9Y618	0.064	-29.158
	4c52	Q07817	0.122	-20.117
	7qp4	P51449	0.067	-26.280
	**8hum**	P37231-2	0.074	-24.656
	7n6q	O75908	0.058	-26.570
	5ho8	O15530	0.112	-19.375
	3znr	Q8WUI4	0.069	-24.993
	7dtc	Q14524	0.069	-24.736
	7lhb	P10415, P10415	0.085	-22.276
	2g3t	P21673	0.069	-24.284
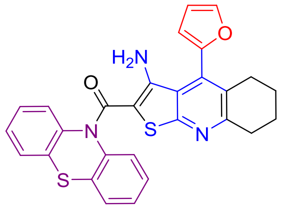 **12h**	**2zno**	P37231	0.116	-23.945
	8hup	P37231-2	0.093	-26.681
	**3zln**	Q07817	0.137	-19.683
	7qp4	P51449	0.078	-25.781
	2j14	Q03181	0.058	-28.255
	5vb5	Q15596	0.062	-27.697
	6p9f	Q15788	0.069	-26.499
	7wld	Q969N2, Q96S52, Q9H490	0.055	-28.140
	5pay	P08709	0.090	-22.943
	3znr	Q8WUI4	0.064	-26.194
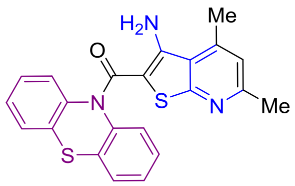 **12i**	**3zln**	Q07817	0.157	-17.454
	3vo3	P35968	0.096	-20.368
	3nnv	Q16539	0.078	-22.685
	**2zno**	P37231	0.072	-23.262
	5ho7	O15530	0.122	-15.840
	4trz	P56817	0.077	-21.300
	2byi	P07900	0.081	-20.324
	4uja	P17612	0.092	-18.859
	Q14568	Q14568	0.055	-23.393
	8hup	P37231-2	0.060	-22.570
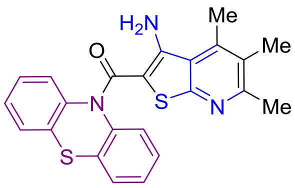 **12j**	**2zno**	P37231	0.096	-23.618
	**3zln**	Q07817	0.136	-18.022
	2byi	P07900	0.091	-21.018
	3vo3	P35968	0.095	-20.216
	7ruh	O60885	0.090	-20.472
	**8hum**	P37231-2	0.075	-22.264
	7qp4	P51449	0.070	-22.656
	5ho7	O15530	0.116	-16.360
	5tzr	O14842	0.057	-24.105
	4gu6	Q05397	0.067	-22.220
	**2zno**	P37231	0.110	-22.684
	**3zln**	Q07817	0.127	-18.461
	4hw3	Q07820, Q07820	0.061	-26.777
	**8hum**	P37231-2	0.088	-23.036

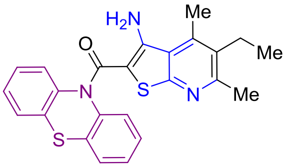 **12k**	7qp4	P51449	0.080	-23.653
	5ho8	O15530	0.118	-17.840
	3cs9	P00519	0.086	-21.848
	6a6p	Q03181	0.056	-25.541
	6lx4	Q07869	0.063	-24.406
	4uja	P17612	0.077	-22.454
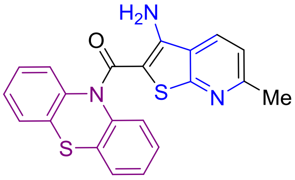 **12l**	5ho7	O15530	0.145	-15.708
	**3zln**	Q07817	0.134	-16.589
	3cs9	P00519	0.101	-20.066
	4trz	P56817	0.075	-21.848
	6w3a	O75460	0.069	-22.291
	3vo3	P35968	0.094	-18.759
	4uw1	O95271	0.057	-23.512
	7rhk	P29973, P29973, P29973, Q14028	0.056	-23.454
	5bui	P28482	0.081	-20.179
	5uch	P08238	0.069	-21.422
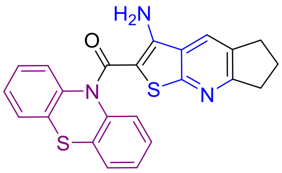 **12m**	**3zln**	Q07817	0.151	-17.768
	4trz	P56817	0.079	-24.320
	**2zno**	P37231	0.084	-23.092
	5ho7	O15530	0.127	-17.135
	3ue4	P00519	0.093	-21.400
	3vo3	P35968	0.093	-20.544
	8hup	P37231-2	0.066	-23.739
	2gu8	P17612	0.085	-20.329
	6xag	P15056	0.071	-21.979
	7fzf	P15090	0.080	-20.640
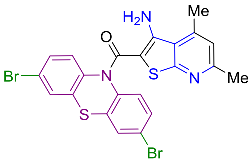 **12n**	**2zno**	P37231	0.095	-25.516
	**3zln**	Q07817	0.132	-18.658
	5ho7	O15530	0.130	-17.164
	**8hum**	P37231-2	0.074	-24.474
	1rd4	P20701, P20701	0.071	-22.759
	7qp4	P51449	0.062	-23.906
	6vxj	Q9UNQ0, Q9UNQ0	0.061	-23.715
	7tj9	Q01118	0.068	-22.204
	Q8WXI7	Q8WXI7	0.075	-21.227
	3vo3	P35968	0.079	-20.542
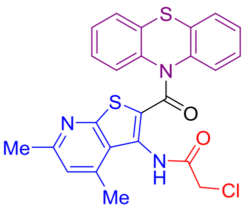 **18**	**3zln**	Q07817	0.155	-18.658
	5ho8	O15530	0.153	-17.939
	7ahu	P00742	0.132	-19.286
	5lay	Q00987	0.118	-19.824
	5ia0	P29317	0.116	-19.911
	**2zno**	P37231	0.104	-20.994
	4rak	P55055	0.062	-25.612
	1rd4	P20701	0.091	-21.139
	7wld	Q969N2, Q96S52, Q9H490	0.056	-25.449
	8ev9	Q16281	0.087	-20.826
	**3zln**	Q07817	0.189	-17.688
	5ho8	O15530	0.148	-17.886
	4lxd	P10415	0.100	-19.396
	8eob	P08238	0.054	-23.917
	3znr	Q8WUI4	0.083	-20.000
	Q86U	Q86UL3	0.050	-24.424

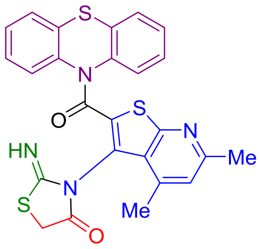<br>**30**	4r0i	Q9Y5Y6	0.062	-22.552
	3ff6	O00763, O00763	0.049	-24.271
	5dvx	Q16790	0.067	-21.941
	4crg	P03951	0.060	-22.457
